# Supplementary material for: Implementation of e-mental health interventions for informal caregivers of adults with chronic diseases: a protocol for a mixed-methods systematic review with a qualitative comparative analysis
Source: BMJ Open. 2020 Jun 21;10(6):e035406. doi: 10.1136/bmjopen-2019-035406 (PMC7307546; doi:10.1136/bmjopen-2019-035406)
Supplement: Supplementary data [file bmjopen-2019-035406supp002.pdf]

PRESS Guideline — Search Submission & Peer Review Assessment

SEARCH SUBMISSION: THIS SECTION TO BE FILLED IN BY THE SEARCHER

Searcher: Chelsea Coumoundouros	Email: chelsea.coumoundouros@kbh.uu.se
Date Submitted: 2019/09/18	Date requested by: 2019/10/09

Systematic Review Title:

Implementation of e-Mental Health interventions for informal caregivers of adults with chronic diseases: a protocol for a mixed methods systematic review with a qualitative comparative analysis

This search strategy is …

X	My PRIMARY (core) database strategy — First time submitting a strategy for search question and database
	My PRIMARY (core) strategy — Follow-up review NOT the first time submitting a strategy for search question and database. If this is a response to peer review, itemize the changes made to the review suggestions
	SECONDARY search strategy— First time submitting a strategy for search question and database
	SECONDARY search strategy — NOT the first time submitting a strategy for search question and database. If this is a response to peer review, itemize the changes made to the review suggestions

Database

(i.e., MEDLINE,CINAHL…): *[mandatory]*

MEDLINE

Interface

(i.e., Ovid, EBSCO…): *[mandatory]*

PubMed

Research Question

(Describe the purpose of the search) *[mandatory]*

The aim of this review is to determine factors related to the successful implementation of e-mental health interventions for caregivers of adults with chronic diseases. Two approaches will be used to investigate this. First, studies with more pragmatic designs will be used exclusively to determine which combinations of intervention or implementation characteristics are associated with effective interventions using a qualitative comparative analysis. Second, reports regarding the implementation of e-mental health interventions will be thematically synthesized to establish the common barriers and facilitators to e-mental health implementation.

PICO Format

(Outline the PICOs for your question — i.e., Patient, Intervention, Comparison, Outcome, and Study Design — as applicable)

**P**	Unpaid adult caregivers of adults with either heart disease, stroke, cancer, diabetes, dementia or chronic obstructive pulmonary disease.
**I**	Any e-mental health intervention targeting the treatment of common mental health difficulties (e.g. depression, anxiety) in caregivers
**C**	**For the qualitative comparative analysis:** Studies must use a non-active control (no treatment, wait-list control, treatment as usual, non-specific treatment component control or education on the care recipient's condition). **For the thematic synthesis:** No restriction based on presence/absence of control group or control type.
**O**	**For the qualitative comparative analysis:** Depression, anxiety, stress or distress measured using an instrument with at least acceptable reliability (Cronbach's alpha ≥ 0.7). **For the thematic synthesis:** Barriers and/or facilitators to implementation.
**S**	**For the qualitative comparative analysis:** Studies must be randomized controlled effectiveness studies. **For the thematic synthesis:** No restriction based on study design.

Inclusion Criteria *[optional]*

P: Caregivers and care recipients must be aged 18 years or older. Chronic health conditions eligible for inclusion are listed in the PICOS above.

I: Interventions must be primarily delivered using Internet technology and target the caregiver's mental health. Interventions may be supplemented by additional forms of support (e.g. telephone call, face-to-face session). Any type of therapy will be included.

O: For the thematic synthesis: Barriers and facilitators can include factors related to any aspect of the Consolidated Framework for Implementation Research or the implementation outcome framework developed by Proctor and colleagues.

S: For the qualitative comparative analysis: Effectiveness will be defined as any study with a mean score of 3 or more using the PRECIS-2 tool.

Exclusion Criteria *[optional]*

P: Studies that focus on caregivers with severe mental health conditions, caregivers providing care to non-community dwelling care recipients or care recipients at the palliative phase of disease will be excluded.

I: Interventions delivered using the telephone, CD-ROM or video (including Skype) alone will be excluded.

C: For the qualitative comparative analysis: Studies using psychoeducation, education on mental health or active controls will be excluded.

Date limit: Studies published prior to 2007 will be excluded. Technologies from work published prior to 2007 may be outdated and other reviews have shown that production of publications involving eHealth began to rise from 2007 onwards

Language restriction: Studies written in languages other than English or Swedish will be excluded.

Was a search filter applied?

Yes ■ No ☐

If YES, which one(s) (e.g., Cochrane RCT filter, PubMed Clinical Queries filter)? Provide the source if this is a published filter. *[mandatory if YES to previous question — textbox]*

Date limit was applied as part of the search.

Other notes or comments you feel would be useful for the peer reviewer? ***[optional]***

There are no terms related to implementation as (1) this is not the only area we are interested in (also need effectiveness studies) and (2) implementation can be referred to in many different ways and is sometimes not mentioned at all in the title or abstract of studies, so trying to create terms about this topic may lead to poor retrieval of relevant work

Please copy and paste your search strategy here, exactly as run, including the number of hits per line. ***[mandatory]***

**Database: PubMed. Final number of results from the full search 3265<br>Date Restriction: January 1, 2007 to present**				
**1: Caregivers**	**2: Chronic Health Conditions**	**3: eHealth/ Technology**	**4: Mental Health**	**5: Therapy**
**Title/Abstract**	**Title/Abstract**	**Title/Abstract**	**Title/Abstract**	**Title/Abstract**
Caregiver 19465	Cancer 1019721	eHealth 2990	Mental health 92203	Mental health service 3222
Caregivers 35586	Tumor 615354	e-health 2140	Mood 45028	Mental health services 10159
Care-giver 301	Tumors 279910	e-mental health 213	Mood disorder 3595	Mental healthcare 1741
Care-givers 1034	Tumours 49415	emental health 30	Depression 185606	Therapy 923016
Carer 3436	Tumour 95450	mhealth 3596	Depressive 72042	Therapies 201143
Carers 8055	Neoplasm 34474	m-health 462	Depressed 30180	Treatment 2180941
Informal carer 94	Neoplasms 51500	Smartphone 7429	Affective disorder 2287	Treatments 283397
Informal carers 575	Oncology 65177	Cell phone 1702	Affect 366439	Interventions 307575
Informal caregiver 460	Carcinoma 275810	Cellular phone 362	Negative affect 7192	Intervention 397118
Informal caregivers 1917	Malignan* 271316	Mobile phone 5430	Dysthymia 886	Program 229608
Family 412900	Melanoma 56126	Mobile app 1046	Dysphoria 1871	Programs 158202
Families 124835	Metastasis 147467	Mobile apps 991	Melancholic 594	Programmes 36810
Spouse 4404	Lymphoma 76891	Mobile application 1252	Anxiety 117199	Programme 52551
Spousal 2026	Leukemia 86466	Mobile applications 1283	Burden 141973	Psychological 117677
Spouses 4578	Diabetes 309446	App 16442	Distress 63488	Psychologist 2715
Sibling 10277	Diabetes mellitus 108204	Apps 4615	Stress 465139	Psychoeducation 2202
Siblings 15168	Type 1 diabetes 27354	Application 442621	Well being 49008	Psycho-education 518
Husband 2380	Type 2 diabetes 95548	Applications 318200	Emotion 29552	CBT 8475
Husbands 2371	COPD 32037	iPad 1114	Emotional 95270	Cognitive behavioural therapy 3103
Wife 1919	Chronic obstructive pulmonary disease 32548	Computer 94973		Cognitive therapy 1960
Wives 1829		Tablet 14085	**MeSH**	Health education 13665
Partner 47483	COAD 147	Computer based 6611	Mood disorders 55994	Problem solving 9444
Partners 51514	Chronic obstructive airway disease 80	Computer assistive technology 421	Depression 108610	Problem solving therapy 335
Parents 92138			Anxiety 42892	PST 1976
Parent 69723	Chronic obstructive lung disease 1842	Technology 205306	Anxiety disorders 28551	ACT 140661
Friend 6741		Technologies 99160	Stress, psychological 66704	Commitment therapy 809
Friends 16333	Chronic airflow obstruction 102	Electronic communication 757	Emotions 119893	Behaviour 108763
Relatives 28499	Stroke 150135		Mental health 21517	Behavioural 45351
Relative 436237	Cerebrovascular accident 2137	Email 4949		Therapist 9305
Couple 18462	CVA 1540	E-mail 4654		Support 563829
Couples 20787	Cerebral stroke 676	Text messaging 1952		Mindful 2490
Mother 51594	Acute stroke 9906	Text message 1338		Mindfulness 6250
Mothers 64116	Acute cerebrovascular accident 58	Internet 36172		Dialectic behaviour therapy 5
Fathers 9825	Cerebrovascular stroke 133	Internet based 6623		Psychotherapy 13408
Father 10914	Brain vascular accident 2	Wireless 10928		Relaxation 53119
Support person 216	Cerebrovascular apoplexy 2	Digital 71968		Meditation 3440

Support persons 226<br>Next of kin 770<br>Significant other 583<br>Significant others 1502<br><br>**MeSH**<br>Caregivers 23052<br>Family 134295<br>Spouses 5686<br>Siblings 7235<br>Parents 57525<br>Friends 3754<br>Mothers 23221<br>Fathers 4249	Apoplexy 938<br>Heart disease 71238<br>Cardiovascular disease 89039<br>CVD 27460<br>Ischemic heart disease 10129<br>IHD 2703<br>Coronary artery disease 45519<br>CAD 25678<br>Angina 16189<br>Myocardial infarction 85190<br>MI 28920<br>Heart attack 2262<br>Cardiac event 2336<br>Cardiac disease 7437<br>Dementia 64525<br>Alzheimer* 90032<br>Alzheimer's disease 73741<br>Vascular dementia 3528<br>Frontotemporal dementia 4920<br>FTD 2656<br>Lewy bodies 4361<br>Lewy body 2324<br>Neurocognitive disorder 739<br>Neurocognitive disorders 1551<br>Mild cognitive impairment 13740<br>Memory impair* 8743<br>Cognitive decline 16542<br>Chronic illness 7067<br>Chronic illnesses 2903<br>Chronic diseases 23041<br>Chronic disease 23460<br>Chronic condition 3482<br>Chronic conditions 10263<br>Multi-morbidity 476<br>Multimorbidity 3160<br>Multimorbidities 165<br>Multi-morbidities 66<br>Long-term health condition 39	Online 91273<br>On-line 10976<br>Virtual 42066<br>ICT 3885<br>Communication technology 1639<br>Web 82786<br>Web based 23890<br>Website 13756<br>Web page 528<br>Multimedia 2881<br>e-learning 2196<br>Online social network 154<br>e-therapy 146<br>e-therapies 23<br>etherapies 1<br>etherapy 10<br>iCBT 589<br>cCBT 158<br><br>**MeSH**<br>Cell phone 8834<br>Mobile application 4432<br>Computers 14176<br>Therapy, computer assisted 44315<br>Wireless technology 3136<br>Information technology 237<br>Technology 210207<br>Electronic mail 1850<br>Internet 51939<br>Online social networking 57<br>Virtual reality 1079<br>Web browser 962<br>Multimedia 951		Behavior 363942<br>Behavioral 167313<br>Cognitive behavioral therapy 7242<br>Dialectic behavior therapy 10<br>Behavioural activation 328<br>Behavioral activation 862<br>Counseling 38031<br>Counselling 14505<br>Cognitive reframing 59<br>Cognitive restructuring 506<br>Self-help 3173<br>Self-management 14367<br><br>**MeSH**<br>Mental health services 37274<br>Health education 103830<br>Problem solving 9350<br>Psychotherapy 69239<br>Relaxation 8308<br>Self-management 1348<br>Self-help groups 3265

	**MeSH**<br>Neoplasms 1299462<br>Diabetes mellitus 192881<br>Pulmonary disease, chronic obstructive 31584<br>Stroke 83194<br>Cardiovascular diseases 805860<br>Dementia 81697<br>Cognition disorders 56940<br>Chronic disease 85925<br>Multimorbidity 503			
**Total for concept 1: 1 278 504**	**Total for concept 2: 3 399 238**	**Total for concept 3: 1 504 756**	**Total for concept 4: 1 400 395**	**Total for concept 5: 4 478 921**

Note: All MeSH terms are exploded

Total for complete search (includes date restriction): 3265

Date of search: September 4, 2019

Raw PubMed Search

(((((caregiver[Title/Abstract] OR caregivers[Title/Abstract] OR care-giver[Title/Abstract] OR care-givers[Title/Abstract] OR carer[Title/Abstract] OR carers[Title/Abstract] OR informal carer[Title/Abstract] OR informal carers[Title/Abstract] OR informal caregiver[Title/Abstract] OR informal caregivers[Title/Abstract] OR family[Title/Abstract] OR families[Title/Abstract] OR spouse[Title/Abstract] OR spousal[Title/Abstract] OR spouses[Title/Abstract] OR sibling[Title/Abstract] OR siblings[Title/Abstract] OR husband[Title/Abstract] OR husbands[Title/Abstract] OR wife[Title/Abstract] OR wives[Title/Abstract] OR partner[Title/Abstract] OR partners[Title/Abstract] OR parents[Title/Abstract] OR parent[Title/Abstract] OR friend[Title/Abstract] OR friends[Title/Abstract] OR relatives[Title/Abstract] OR relative[Title/Abstract] OR couple[Title/Abstract] OR couples[Title/Abstract] OR mother[Title/Abstract] OR mothers[Title/Abstract] OR fathers[Title/Abstract] OR father[Title/Abstract] OR support person[Title/Abstract] OR support persons[Title/Abstract] OR next of kin[Title/Abstract] OR significant other[Title/Abstract] OR significant others[Title/Abstract] OR caregivers[MeSH terms] OR family[MeSH terms] OR spouses[MeSH terms] OR siblings[MeSH terms] OR parents[MeSH terms] OR friends[MeSH terms] OR mothers[MeSH terms] OR fathers[MeSH terms]) AND (cancer[Title/Abstract] OR tumor[Title/Abstract] OR tumors[Title/Abstract] OR tumours[Title/Abstract] OR tumour[Title/Abstract] OR neoplasm[Title/Abstract] OR neoplasms[Title/Abstract] OR oncology[Title/Abstract] OR carcinoma[Title/Abstract] OR malignan*[Title/Abstract] OR melanoma[Title/Abstract] OR metastasis[Title/Abstract] OR lymphoma[Title/Abstract] OR leukemia[Title/Abstract] OR diabetes[Title/Abstract] OR diabetes mellitus[Title/Abstract] OR Type 1 diabetes[Title/Abstract] OR Type 2 diabetes[Title/Abstract] OR COPD[Title/Abstract] OR chronic obstructive pulmonary disease[Title/Abstract] OR COAD[Title/Abstract] OR chronic obstructive airway disease[Title/Abstract] OR chronic obstructive lung disease[Title/Abstract] OR chronic airflow obstruction[Title/Abstract] OR stroke[Title/Abstract] OR cerebrovascular accident[Title/Abstract] OR CVA[Title/Abstract] OR cerebral stroke[Title/Abstract] OR acute stroke[Title/Abstract] OR acute cerebrovascular accident[Title/Abstract] OR cerebrovascular stroke[Title/Abstract] OR brain vascular accident[Title/Abstract] OR cerebrovascular apoplexy[Title/Abstract] OR apoplexy[Title/Abstract] OR heart disease[Title/Abstract] OR cardiovascular disease[Title/Abstract] OR CVD[Title/Abstract] OR ischemic heart disease[Title/Abstract] OR IHD[Title/Abstract] OR coronary artery disease[Title/Abstract] OR CAD[Title/Abstract] OR angina[Title/Abstract] OR myocardial infarction[Title/Abstract] OR MI[Title/Abstract] OR heart attack[Title/Abstract] OR cardiac event[Title/Abstract] OR cardiac disease[Title/Abstract] OR dementia[Title/Abstract] OR alzheimer*[Title/Abstract] OR alzheimer's disease[Title/Abstract] OR vascular dementia[Title/Abstract] OR frontotemporal dementia[Title/Abstract] OR FTD[Title/Abstract] OR Lewy body[Title/Abstract] OR Lewy bodies[Title/Abstract] OR neurocognitive disorder[Title/Abstract] OR neurocognitive disorders[Title/Abstract] OR mild cognitive impairment[Title/Abstract] OR memory impair*[Title/Abstract] OR cognitive decline[Title/Abstract] OR chronic illness[Title/Abstract] OR chronic illnesses[Title/Abstract] OR chronic diseases[Title/Abstract] OR chronic disease[Title/Abstract] OR chronic condition[Title/Abstract] OR chronic conditions[Title/Abstract] OR multi-morbidity[Title/Abstract] OR multimorbidity[Title/Abstract] OR multimorbidities[Title/Abstract] OR multi-morbidities[Title/Abstract] OR long-term health condition[Title/Abstract] OR neoplasms[MeSH

terms] OR diabetes mellitus[MeSH terms] OR pulmonary disease, chronic obstructive[MeSH terms] OR stroke[MeSH terms] OR cardiovascular diseases[MeSH terms] OR dementia[MeSH terms] OR Cognition disorders[MeSH terms] OR chronic disease[MeSH terms] OR multimorbidity[MeSH terms]) AND (eHealth[Title/Abstract] OR e-health[Title/Abstract] OR e-mental health[Title/Abstract] OR emental health[Title/Abstract] OR mhealth[Title/Abstract] OR m-health[Title/Abstract] OR smartphone[Title/Abstract] OR cell phone[Title/Abstract] OR cellular phone[Title/Abstract] OR mobile phone[Title/Abstract] OR mobile app[Title/Abstract] OR mobile apps[Title/Abstract] OR mobile applications[Title/Abstract] OR mobile application[Title/Abstract] OR app[Title/Abstract] OR application[Title/Abstract] OR applications[Title/Abstract] OR apps[Title/Abstract] OR iPad[Title/Abstract] OR computer[Title/Abstract] OR tablet[Title/Abstract] OR computer based[Title/Abstract] OR computer assistive technology[Title/Abstract] OR technology[Title/Abstract] OR technologies[Title/Abstract] OR electronic communication[Title/Abstract] OR email[Title/Abstract] OR e-mail[Title/Abstract] OR text messaging[Title/Abstract] OR text message[Title/Abstract] OR internet[Title/Abstract] OR internet based[Title/Abstract] OR wireless[Title/Abstract] OR online[Title/Abstract] OR digital[Title/Abstract] OR on-line[Title/Abstract] OR virtual[Title/Abstract] OR ICT[Title/Abstract] OR communication technology[Title/Abstract] OR web[Title/Abstract] OR web based[Title/Abstract] OR website[Title/Abstract] OR web page[Title/Abstract] OR multimedia[Title/Abstract] OR e-learning[Title/Abstract] OR online social network[Title/Abstract] OR iCBT[Title/Abstract] OR cCBT[Title/Abstract] OR e-therapy[Title/Abstract] OR etherapy[Title/Abstract] OR etherapies[Title/Abstract] OR e-therapies[Title/Abstract] OR cell phone[MeSH terms] OR mobile application[MeSH terms] OR computers[MeSH terms] OR therapy, computer assisted[MeSH terms] OR wireless technology[MeSH terms] OR information technology[MeSH terms] OR technology[MeSH terms] OR electronic mail[MeSH terms] OR internet[MeSH terms] OR online social networking[MeSH terms] OR virtual reality[MeSH terms] OR web browser[MeSH terms] OR multimedia[MeSH terms]) AND (mental health[Title/Abstract] OR mood[Title/Abstract] OR mood disorder[Title/Abstract] OR depression[Title/Abstract] OR depressive[Title/Abstract] OR depressed[Title/Abstract] OR affective disorder[Title/Abstract] OR affect[Title/Abstract] OR negative affect[Title/Abstract] OR dysthymia[Title/Abstract] OR dysphoria[Title/Abstract] OR melancholic[Title/Abstract] OR anxiety[Title/Abstract] OR burden[Title/Abstract] OR distress[Title/Abstract] OR stress[Title/Abstract] OR well being[Title/Abstract] OR emotion[Title/Abstract] OR emotional[Title/Abstract] OR mood disorders[MeSH terms] OR depression[MeSH terms] OR anxiety[MeSH terms] OR anxiety disorders[MeSH terms] OR stress, psychological[MeSH terms] OR emotions[MeSH terms] OR mental health[MeSH terms]) AND (mental health service[Title/Abstract] OR mental health services[Title/Abstract] OR mental healthcare[Title/Abstract] OR therapy[Title/Abstract] OR therapies[Title/Abstract] OR treatment[Title/Abstract] OR treatments[Title/Abstract] OR interventions[Title/Abstract] OR intervention[Title/Abstract] OR program[Title/Abstract] OR programs[Title/Abstract] OR programmes[Title/Abstract] OR programme[Title/Abstract] OR psychological[Title/Abstract] OR psychologist[Title/Abstract] OR psychoeducation[Title/Abstract] OR psycho-education[Title/Abstract] OR CBT[Title/Abstract] OR cognitive behavioural therapy[Title/Abstract] OR cognitive therapy[Title/Abstract] OR health education[Title/Abstract] OR problem solving[Title/Abstract] OR problem solving therapy[Title/Abstract] OR PST[Title/Abstract] OR ACT[Title/Abstract] OR commitment therapy[Title/Abstract] OR behaviour[Title/Abstract] OR

behavioural[Title/Abstract] OR therapist[Title/Abstract] OR support[Title/Abstract] OR mindful[Title/Abstract] OR mindfulness[Title/Abstract] OR dialectic behaviour therapy[Title/Abstract] OR psychotherapy[Title/Abstract] OR relaxation[Title/Abstract] OR meditation[Title/Abstract] OR behavior[Title/Abstract] OR behavioral[Title/Abstract] OR cognitive behavioral therapy[Title/Abstract] OR behavioural activation[Title/Abstract] OR behavioral activation[Title/Abstract] OR dialectic behavior therapy[Title/Abstract] OR counseling[Title/Abstract] OR counselling[Title/Abstract] OR cognitive reframing[Title/Abstract] OR cognitive restructuring[Title/Abstract] OR self-help[Title/Abstract] OR self-management[Title/Abstract] OR mental health services[MeSH terms] OR health education[MeSH terms] OR problem solving[MeSH terms] OR psychotherapy[MeSH terms] OR relaxation[MeSH terms] OR self-management[MeSH terms] OR self-help groups[MeSH terms]))))) AND ("2007/01/01"[Date - Publication] : "3000"[Date - Publication])

PEER REVIEW ASSESSMENT #1: THIS SECTION TO BE FILLED IN BY THE REVIEWER

Reviewer:	Email:	Date completed: 2019/10/03
Professor Mariët Hagedoorn & Truus van Ittersum	mariet.hagedoorn@umcg.nl	

1. TRANSLATION

A ---No revisions	☐
B --- Revision(s) suggested	☐
C --- Revision(s) required	x

If "B" or "C," please provide an explanation or example:

Does the search strategy match the research question/PICO?
I'm not familiar with the PRECIS-2 tool. Do you use it to rate the effectiveness of an internet technology intervention in reducing distress in caregivers as compared to a non-active control group?
Considering your overall question, I would expect that you would assess the effectiveness/successfulness of the implementation?
Are the search concepts clear?
Are there too many or too few PICO elements included?
You could consider not applying the fifth concept, but I can see the point why you do.
Are the search concepts too broad or too narrow?
Does the search retrieve too many or too few records?
Are unconventional or complex strategies explained?
The main focus is on successful implementation, which I find a relevant and timely issue to address in a review. However, the two approaches are not clear to me. For the first approach (which combination of intervention and implementation characteristics are associated with effective interventions), you want to include RCTs. To my understanding RCTs test the effectiveness of an intervention and usually does not include the aim of implementation. Will these studies be relevant to answer your overall question "which factors are related to successful implementation"? It would be helpful to clarify this.
On a practical note, the combination of two approaches appears to make the coding rather complex. Wouldn't it be better to do this in phases? First RCTs, than the remaining literature for approach 2?

I would advise to clarify the two approaches. As I commented above, it is not clear to me how approach one answers your overall research question. I also advise you to reconsider whether you really need two approaches, with specific inclusion criteria. In case you do need both approaches, would it be possible to use a two-step coding process and first search/code RCTs and than apply the second strategy?

Author response:
Thank you for your feedback. We believe that approach one (the qualitative comparative analysis) can produce results that relate to the overall research question regarding implementation of e-mental health interventions for caregivers. Approach one will only utilize pragmatic randomized controlled trials in the analysis. Pragmatic trials use conditions (e.g. the setting, follow up etc.) that more closely reflect how the interventions would be used in real-world settings. Therefore, pragmatic trials are more likely than explanatory trials to include details concerning implementation, which can be included in the qualitative comparative analysis to assess whether certain implementation factors are important for interventions effectiveness. Further, pragmatic trials examine effectiveness in near to real-world settings, and as such, provide estimates of intervention effectiveness that may better reflect estimates of effectiveness that may be found if the intervention was implemented in real-world practices. The PRECIS-2 tool evaluates how pragmatic the design of a randomized controlled trial is. By using this tool in the screening process, only trials with more pragmatic features will be included in the qualitative comparative analysis. The results of the qualitative comparative analysis can also be contrasted to the barriers and

facilitators identified in the thematic synthesis to determine if, for example, a factor identified as a facilitator to implementation in the thematic synthesis was also identified as important for intervention effectiveness in the qualitative comparative analysis.

We agree, the two approaches adds complexity to the literature screening process, however, if we were to only search for RCTs as a first step, we may miss many reports on implementation of interventions relevant to our research question.

2. BOOLEAN AND PROXIMITY OPERATORS

A ---No revisions	X
B --- Revision(s) suggested	☐
C --- Revision(s) required	☐

If "B" or "C," please provide an explanation or example:

Are Boolean or proximity operators used correctly?
Is the use of nesting with brackets appropriate and effective for the search?
If NOT is used, is this likely to result in any unintended exclusions?
Could precision be improved by using proximity operators (e.g. adjacent, near, within) or phrase searching instead of AND?
Is the width of proximity operators suitable (e.g. might adj5 pick up more variants than adj2)?

3. SUBJECT HEADINGS

A ---No revisions	X
B --- Revision(s) suggested	☐
C --- Revision(s) required	☐

If "B" or "C," please provide an explanation or example:

Are the subject headings relevant?
Are any relevant subject headings missing (e.g. previous index terms)?
Are any subject headings too broad or too narrow?
Are subject headings exploded where necessary and vice versa?
Are major headings ("starring" or restrict to focus) used? If so, if there adequate justification?
Are subheadings missing?
Are subheadings attached to subject headings? (Floating subheadings may be preferred)
Are floating subheadings relevant and used appropriately?
Are both subject headings and terms in free text used for each concept?

4. TEXT WORD SEARCHING

A ---No revisions	☐
B --- Revision(s)suggested	X
C --- Revision(s) required	☐

If "B" or "C," please provide an explanation or example:

General: there is an overload of terms. Some specific terms are not necessary as publications are already found by more general terms (<diabetes> also finds <diabetes mellitus>, <affect> also covers <negative affect>.

11

Does the search include all spelling variants in free text (e.g. UK versus US spelling)?
Does the search include all synonyms or antonyms (e.g. opposites)?
Does the search capture relevant truncation (e.g. is truncation at the correct place)?
Using the <*> helps narrowing down the number of terms, without loosing results. <Husband*> will also find <husbands>, <caregiver*> will also find <caregivers>. There are a lot of these terms in the list.
Are acronyms or abbreviations used appropriately? Do they capture irrelevant material? Are the full terms also included?
Are the keywords specific enough or too broad? Are too many or too few keywords used? Are stop words used?
Term “Technology”: Yields many results. Does not seem very relevant. Maybe skip this term?
Term “Web”: Yields mostly “Web of Science” OR “Web of Knowledge” hits. Maybe exclude? Reconsider?
Term “Multimorbidity”: Why multimorbidity? Populations should have a chronic illness and they are specified.
Term “long-term health condition”: To vague.
Have the appropriate fields been searched (e.g. is the choice of text world fields (.tw.) or all fields (.af.) appropriate? Are there any other fields to be included or excluded (database-specific)?
Should any long strings be broken into several shorter search statements?

Author response:
Thank you for these comments. Your feedback has been incorporated into a revised search strategy which has eliminated unnecessary terms and used more term truncations.

Although the terms “technology” and “web” are very broad, we feel they should remain in the primary search strategy as they may yield relevant results pertaining to technological or web based interventions. The terms “multimorbidity” and “long-term health condition” are included as we have come across publications which refer to the population under investigation using more generic terms, such as having multimorbidities or chronic diseases, with the specific health conditions only specified in the main text. Therefore, to ensure the search captures all publications that include caregivers of individuals with the health conditions of interest to this review, we included these more general terms.

5. SPELLING, SYNTAX, AND LINE NUMBERS

A ---No revisions	X
B --- Revision(s)suggested	☐
C --- Revision(s) required	☐

If “B” or “C,” please provide an explanation or example:

Are there any spelling errors
Are there errors in system syntax (e.g. the use of a truncation symbol from a different search interface)?
Are there incorrect line combinations or orphan lines (e.g. lines that are not referred to in the final summation that could indicate an error in an AND or OR statement)?

6. LIMITS AND FILTERS

A ---No revisions	X
B --- Revision(s) suggested	☐
C --- Revision(s) required	☐

If “B” or “C,” please provide an explanation or example:

Are all limits and filters used appropriately and are they relevant given the research question?
Are all limits and filters used appropriately and are they relevant for the database?
Are any potentially helpful limits or filters missing? Are the limits or filters too broad or too narrow? Can any limits or filters be added or removed?
Are sources cited for the filters used?

OVERALL EVALUATION (Note: If one or more "revision required" is noted above, the response below must be "revisions required".)

A ---No revisions	☐
B --- Revision(s) suggested	☐
C --- Revision(s) required	X

Additional comments: see above

PEER REVIEW ASSESSMENT #2: THIS SECTION TO BE FILLED IN BY THE REVIEWER

Reviewer:<br>Dr. Nathan Davies	Email:<br>n.m.davies@ucl.ac.uk	Date completed: 2019/10/07

1. TRANSLATION

A ---No revisions	X
B --- Revision(s) suggested	☐
C --- Revision(s) required	☐

If "B" or "C," please provide an explanation or example:

Does the search strategy match the research question/PICO?
Yes
Are the search concepts clear?
Yes
Are there too many or too few PICO elements included?
This is a very broad search and large area to study, it is doable but you may consider splitting the review into two.
Are the search concepts too broad or too narrow?
No, they are broad but needed for the question
Does the search retrieve too many or too few records?
My only concern is that this is going to be a very large review and managing such a large review
Are unconventional or complex strategies explained?
N/a

2. BOOLEAN AND PROXIMITY OPERATORS

A ---No revisions	X
B --- Revision(s) suggested	☐
C --- Revision(s) required	☐

If "B" or "C," please provide an explanation or example:

Are Boolean or proximity operators used correctly?
Yes
Is the use of nesting with brackets appropriate and effective for the search?
Yes
If NOT is used, is this likely to result in any unintended exclusions?
Could precision be improved by using proximity operators (e.g. adjacent, near, within) or phrase searching instead of AND?
No
Is the width of proximity operators suitable (e.g. might adj5 pick up more variants than adj2)?
n/a

3. SUBJECT HEADINGS

A ---No revisions	X
B --- Revision(s) suggested	☐
C --- Revision(s) required	☐

If "B" or "C," please provide an explanation or example:

Are the subject headings relevant?
Yes
Are any relevant subject headings missing (e.g. previous index terms)?
None obvious that I could see
Are any subject headings too broad or too narrow?
No
Are subject headings exploded where necessary and vice versa?
Yes
Are major headings ("starring" or restrict to focus) used? If so, if there adequate justification?
No
Are subheadings missing?
No
Are subheadings attached to subject headings? (Floating subheadings may be preferred)
n/a
Are floating subheadings relevant and used appropriately?
n/a
Are both subject headings and terms in free text used for each concept?
Yes

4. TEXT WORD SEARCHING

A ---No revisions	☐
B --- Revision(s)suggested	X
C --- Revision(s) required	☐

If "B" or "C," please provide an explanation or example:

Does the search include all spelling variants in free text (e.g. UK versus US spelling)?
Add MCI for mild cognitive impairment
Look at searching the term digital health.
Look at cognitive stimulation therapy which can be used with people with dementia
Does the search include all synonyms or antonyms (e.g. opposites)?
See above
Does the search capture relevant truncation (e.g. is truncation at the correct place)?
You can add * to some words which may want to be searched with and without an s at the end for example husband and husbands could just be searched as husband*. But this would not effect the numbers you have already retrieved but may make the search easier to follow with less terms.
Are acronyms or abbreviations used appropriately? Do they capture irrelevant material? Are the full terms also included?
See above
Are the keywords specific enough or too broad? Are too many or too few keywords used? Are stop words used?
See above

Have the appropriate fields been searched (e.g. is the choice of text world fields (.tw.) or all fields (.af.) appropriate? Are there any other fields to be included or excluded (database-specific)?
Yes
Should any long strings be broken into several shorter search statements?
No

Author response:
Thank you for these suggestions. MCI has been added to the search strategy and more terms have been truncated using the *. Digital health and cognitive stimulation therapy are very relevant concepts, however, the were not added to the search strategy as they should be captured by the terms “digital” and “therapy”, respectively, in the existing search strategy.

5. SPELLING, SYNTAX, AND LINE NUMBERS

A ---No revisions	X
B --- Revision(s)suggested	☐
C --- Revision(s) required	☐

If “B” or “C,” please provide an explanation or example:

Are there any spelling errors
no
Are there errors in system syntax (e.g. the use of a truncation symbol from a different search interface)?
no
Are there incorrect line combinations or orphan lines (e.g. lines that are not referred to in the final summation that could indicate an error in an AND or OR statement)?
no

6. LIMITS AND FILTERS

A ---No revisions	X
B --- Revision(s) suggested	☐
C --- Revision(s) required	☐

If “B” or “C,” please provide an explanation or example:

Are all limits and filters used appropriately and are they relevant given the research question?
The year limit seems appropriate you may even be able to change this to 2009 as last 10 years is a long time in technology so anything older than 10 years is most likely to be irrelevant now.
Are all limits and filters used appropriately and are they relevant for the database?
Yes – although for non-english/sweedish language you could rapidly appraise the English abstract if available and if seems very relevant seek translation of the remainder of the article.
Are any potentially helpful limits or filters missing? Are the limits or filters too broad or too narrow? Can any limits or filters be added or removed?
no
Are sources cited for the filters used?
Don’t understand this question

OVERALL EVALUATION (Note: If one or more "revision required" is noted above, the response below must be "revisions required".)

A ---No revisions	☐
B --- Revision(s) suggested	X
C --- Revision(s) required	☐

Additional comments:

Coumoundouros C, *et al. BMJ Open* 2020; 10:e035406. doi: 10.1136/bmjopen-2019-035406
