# Supplementary material for: Implementation of e-mental health interventions for informal caregivers of adults with chronic diseases: a protocol for a mixed-methods systematic review with a qualitative comparative analysis
Source: BMJ Open. 2020 Jun 21;10(6):e035406. doi: 10.1136/bmjopen-2019-035406 (PMC7307546; doi:10.1136/bmjopen-2019-035406)
Supplement: Supplementary data [file bmjopen-2019-035406supp003.pdf]

Implementation of e-mental health interventions for informal caregivers of adults with chronic diseases: a protocol for a mixed methods systematic review with a qualitative comparative analysis

Supplementary File 3: Search Strategy

PubMed

#	
**1**	caregiver*[Title/Abstract] OR<br>care-giver*[Title/Abstract] OR<br>carer*[Title/Abstract] OR<br>family[Title/Abstract] OR<br>families[Title/Abstract] OR<br>spous*[Title/Abstract] OR<br>sibling*[Title/Abstract] OR<br>husband*[Title/Abstract] OR<br>wife[Title/Abstract] OR<br>wives[Title/Abstract] OR<br>partner[Title/Abstract] OR<br>partners[Title/Abstract] OR<br>parents[Title/Abstract] OR<br>parent[Title/Abstract] OR<br>friend[Title/Abstract] OR<br>friends[Title/Abstract] OR<br>relative[Title/Abstract] OR<br>relatives[Title/Abstract] OR<br>couple[Title/Abstract] OR<br>couples[Title/Abstract] OR<br>mother*[Title/Abstract] OR<br>father*[Title/Abstract] OR<br>support person*[Title/Abstract] OR<br>next of kin[Title/Abstract] OR<br>significant other*[Title/Abstract] OR<br>caregivers[MeSH terms] OR<br>family[MeSH terms] OR<br>spouses[MeSH terms] OR<br>siblings[MeSH terms] OR<br>parents[MeSH terms] OR<br>friends[MeSH terms] OR<br>mothers[MeSH terms] OR<br>fathers[MeSH terms]
**2**	cancer[Title/Abstract] OR<br>tumor[Title/Abstract] OR<br>tumors[Title/Abstract] OR<br>tumours[Title/Abstract] OR<br>tumour[Title/Abstract] OR<br>neoplasm*[Title/Abstract] OR<br>oncology[Title/Abstract] OR<br>carcinoma[Title/Abstract] OR<br>malignan*[Title/Abstract] OR

	melanoma[Title/Abstract] OR<br>metastasis[Title/Abstract] OR<br>lymphoma[Title/Abstract] OR<br>leukemia[Title/Abstract] OR<br>diabetes[Title/Abstract] OR<br>COPD[Title/Abstract] OR<br>chronic obstructive pulmonary disease[Title/Abstract] OR<br>COAD[Title/Abstract] OR<br>chronic obstructive airway disease[Title/Abstract] OR<br>chronic obstructive lung disease[Title/Abstract] OR<br>chronic airflow obstruction[Title/Abstract] OR<br>stroke[Title/Abstract] OR<br>cerebrovascular accident[Title/Abstract] OR<br>CVA[Title/Abstract] OR<br>acute cerebrovascular accident[Title/Abstract] OR<br>brain vascular accident[Title/Abstract] OR<br>apoplexy[Title/Abstract] OR<br>heart disease[Title/Abstract] OR<br>cardiovascular disease[Title/Abstract] OR<br>CVD[Title/Abstract] OR<br>ischemic heart disease[Title/Abstract] OR<br>IHD[Title/Abstract] OR<br>coronary artery disease[Title/Abstract] OR<br>CAD[Title/Abstract] OR<br>angina[Title/Abstract] OR<br>myocardial infarction[Title/Abstract] OR<br>MI[Title/Abstract] OR<br>heart attack[Title/Abstract] OR<br>cardiac event[Title/Abstract] OR<br>cardiac disease[Title/Abstract] OR<br>dementia[Title/Abstract] OR<br>alzheimer*[Title/Abstract] OR<br>FTD[Title/Abstract] OR<br>Lewy body[Title/Abstract] OR<br>Lewy bodies[Title/Abstract] OR<br>neurocognitive disorder*[Title/Abstract] OR<br>MCI[Title/Abstract] OR<br>mild cognitive impairment[Title/Abstract] OR<br>memory impair*[Title/Abstract] OR<br>cognitive decline[Title/Abstract] OR<br>chronic illness*[Title/Abstract] OR<br>chronic disease*[Title/Abstract] OR<br>chronic condition*[Title/Abstract] OR<br>multi-morbidit*[Title/Abstract] OR<br>multimorbidit*[Title/Abstract] OR<br>long-term health condition*[Title/Abstract] OR<br>neoplasms[MeSH terms] OR<br>diabetes mellitus[MeSH terms] OR<br>pulmonary disease, chronic obstructive[MeSH terms] OR<br>stroke[MeSH terms] OR<br>cardiovascular diseases[MeSH terms] OR

	dementia[MeSH terms] OR<br>cognition disorders[MeSH terms] OR<br>chronic disease[MeSH terms] OR<br>multimorbidity[MeSH terms]
**3**	eHealth[Title/Abstract] OR<br>e-health[Title/Abstract] OR<br>e-mental health[Title/Abstract] OR<br>emental health[Title/Abstract] OR<br>mhealth[Title/Abstract] OR<br>m-health[Title/Abstract] OR<br>smartphone[Title/Abstract] OR<br>cell phone[Title/Abstract] OR<br>cellular phone[Title/Abstract] OR<br>mobile phone[Title/Abstract] OR<br>app[Title/Abstract] OR<br>apps[Title/Abstract] OR<br>application[Title/Abstract] OR<br>applications[Title/Abstract] OR<br>iPad[Title/Abstract] OR<br>computer[Title/Abstract] OR<br>tablet[Title/Abstract] OR<br>technology[Title/Abstract] OR<br>technologies[Title/Abstract] OR<br>electronic communication[Title/Abstract] OR<br>email[Title/Abstract] OR<br>e-mail[Title/Abstract] OR<br>text messag*[Title/Abstract] OR<br>internet[Title/Abstract] OR<br>wireless[Title/Abstract] OR<br>online[Title/Abstract] OR<br>digital[Title/Abstract] OR<br>on-line[Title/Abstract] OR<br>virtual[Title/Abstract] OR<br>ICT[Title/Abstract] OR<br>web[Title/Abstract] OR<br>website[Title/Abstract] OR<br>multimedia[Title/Abstract] OR<br>e-learning[Title/Abstract] OR<br>online social network[Title/Abstract] OR<br>iCBT[Title/Abstract] OR<br>cCBT[Title/Abstract] OR<br>e-therap*[Title/Abstract] OR<br>etherap*[Title/Abstract] OR<br>cell phone[MeSH terms] OR<br>mobile application[MeSH terms] OR<br>computers[MeSH terms] OR<br>therapy, computer assisted[MeSH terms] OR<br>wireless technology[MeSH terms] OR<br>information technology[MeSH terms] OR<br>technology[MeSH terms] OR<br>electronic mail[MeSH terms] OR

	internet[MeSH terms] OR<br>online social networking[MeSH terms] OR<br>virtual reality[MeSH terms] OR<br>web browser[MeSH terms] OR<br>multimedia[MeSH terms]
**4**	mental health[Title/Abstract] OR<br>mood[Title/Abstract] OR<br>depress*[Title/Abstract] OR<br>affective disorder[Title/Abstract] OR<br>negative affect[Title/Abstract] OR<br>dysthymia[Title/Abstract] OR<br>dysphoria[Title/Abstract] OR<br>melancholic[Title/Abstract] OR<br>anxiety[Title/Abstract] OR<br>burden[Title/Abstract] OR<br>distress[Title/Abstract] OR<br>stress[Title/Abstract] OR<br>well being[Title/Abstract] OR<br>emotion[Title/Abstract] OR<br>emotional[Title/Abstract] OR<br>mood disorders[MeSH terms] OR<br>depression[MeSH terms] OR<br>anxiety[MeSH terms] OR<br>anxiety disorders[MeSH terms] OR<br>stress, psychological[MeSH terms] OR<br>emotions[MeSH terms] OR<br>mental health[MeSH terms]
**5**	mental health service*[Title/Abstract] OR<br>mental healthcare[Title/Abstract] OR<br>therapy[Title/Abstract] OR<br>therapies[Title/Abstract] OR<br>treatment*[Title/Abstract] OR<br>intervention*[Title/Abstract] OR<br>program*[Title/Abstract] OR<br>psychological[Title/Abstract] OR<br>psychologist[Title/Abstract] OR<br>psychoeducation[Title/Abstract] OR<br>psycho-education[Title/Abstract] OR<br>CBT[Title/Abstract] OR<br>health education[Title/Abstract] OR<br>problem solving[Title/Abstract] OR<br>PST[Title/Abstract] OR<br>ACT[Title/Abstract] OR<br>behaviour*[Title/Abstract] OR<br>therapist[Title/Abstract] OR<br>support[Title/Abstract] OR<br>mindful*[Title/Abstract] OR<br>psychotherapy[Title/Abstract] OR<br>relaxation[Title/Abstract] OR<br>meditation[Title/Abstract] OR<br>behavior*[Title/Abstract] OR

	counseling[Title/Abstract] OR<br>counselling[Title/Abstract] OR<br>cognitive reframing[Title/Abstract] OR<br>cognitive restructuring[Title/Abstract] OR<br>self-help[Title/Abstract] OR<br>self-management[Title/Abstract] OR<br>blended[Title/Abstract] OR<br>mental health services[MeSH terms] OR<br>health education[MeSH terms] OR<br>problem solving[MeSH terms] OR<br>psychotherapy[MeSH terms] OR<br>relaxation[MeSH terms] OR<br>self-management[MeSH terms] OR<br>self-help groups[MeSH terms]
**6**	"2007/01/01"[Date - Publication] : "3000"[Date - Publication]
**7**	#1 AND #2 AND #3 AND #4 AND #5 AND #6

CINAHL Plus with Full Text

	((TI (caregiver* OR care-giver* OR carer* OR family OR families OR spous* OR sibling* OR husband* OR wife OR wives OR partner OR partners OR parents OR parent OR friend OR friends OR relative OR relatives OR couple OR couples OR mother* OR father* OR support person* OR next of kin OR significant other*) OR AB (caregiver* OR care-giver* OR carer* OR family OR families OR spous* OR sibling* OR husband* OR wife OR wives OR partner OR partners OR parents OR parent OR friend OR friends OR relative OR relatives OR couple OR couples OR mother* OR father* OR support person* OR next of kin OR significant other*) OR MH (caregivers+ OR family+ OR spouses+ OR siblings+ OR parents+ OR friends+ OR mothers+ OR fathers+)) AND (TI (cancer OR tumor OR tumors OR tumours OR tumour OR neoplasm* OR oncology OR carcinoma OR malignan* OR melanoma OR metastasis OR lymphoma OR leukemia OR diabetes OR COPD OR chronic obstructive pulmonary disease OR COAD OR chronic obstructive airway disease OR chronic obstructive lung disease OR chronic airflow obstruction OR stroke OR cerebrovascular accident OR CVA OR acute cerebrovascular accident OR brain vascular accident OR apoplexy OR heart disease OR cardiovascular disease OR CVD OR ischemic heart disease OR IHD OR coronary artery disease OR CAD OR angina OR myocardial infarction OR MI OR heart attack OR cardiac event OR cardiac disease OR dementia OR alzheimer* OR FTD OR Lewy body OR Lewy bodies OR neurocognitive disorder* OR MCI OR mild cognitive impairment OR memory impair* OR cognitive decline OR chronic illness* OR chronic disease* OR chronic condition* OR multi-morbidit* OR multimorbidit* OR long-term health condition*) OR AB (cancer OR tumor OR tumors OR tumours OR tumour OR neoplasm* OR oncology OR carcinoma OR malignan* OR melanoma OR metastasis OR lymphoma OR leukemia OR diabetes OR COPD OR chronic obstructive pulmonary disease OR COAD OR chronic obstructive airway disease OR chronic obstructive lung disease OR chronic airflow obstruction OR stroke OR cerebrovascular accident OR CVA OR acute cerebrovascular accident OR brain vascular accident OR apoplexy OR heart disease OR cardiovascular disease OR CVD OR ischemic heart disease OR IHD OR coronary artery disease OR CAD OR angina OR myocardial infarction OR MI OR heart attack OR cardiac event OR cardiac disease OR dementia OR alzheimer* OR FTD OR Lewy body OR Lewy bodies OR neurocognitive disorder* OR MCI OR mild cognitive impairment OR memory impair* OR cognitive decline OR chronic illness* OR chronic disease* OR chronic condition* OR multi-morbidit* OR multimorbidit* OR long-term health condition*) OR MH (neoplasms+ OR diabetes mellitus+ OR pulmonary disease, chronic obstructive+ OR stroke+ OR

	cardiovascular diseases+ OR dementia+ OR Cognition disorders+ OR Chronic disease+ OR multimorbidity+)) AND (TI (eHealth OR e-health OR e-mental health OR emental health OR mhealth OR m-health OR smartphone OR cell phone OR cellular phone OR mobile phone OR app OR application OR applications OR apps OR iPad OR computer OR tablet OR technology OR technologies OR electronic communication OR email OR e-mail OR text messag* OR internet OR wireless OR online OR digital OR on-line OR virtual OR ICT OR web OR website OR multimedia OR e-learning OR online social network OR iCBT OR cCBT OR e-therap* OR etherap*) OR AB (eHealth OR e-health OR e-mental health OR emental health OR mhealth OR m-health OR smartphone OR cell phone OR cellular phone OR mobile phone OR app OR application OR applications OR apps OR iPad OR computer OR tablet OR technology OR technologies OR electronic communication OR email OR e-mail OR text messag* OR internet OR wireless OR online OR digital OR on-line OR virtual OR ICT OR web OR website OR multimedia OR e-learning OR online social network OR iCBT OR cCBT OR e-therap* OR etherap*) OR MH (cell phone+ OR mobile application+ OR computers+ OR therapy, computer assisted+ OR wireless technology+ OR information technology+ OR technology+ OR electronic mail+ OR internet+ OR online social networking+ OR virtual reality+ OR web browser+ OR multimedia+)) AND (TI (mental health OR mood OR depress* OR affective disorder OR negative affect OR dysthymia OR dysphoria OR melancholic OR anxiety OR burden OR distress OR stress OR well being OR emotion OR emotional) OR AB (mental health OR mood OR depress* OR affective disorder OR negative affect OR dysthymia OR dysphoria OR melancholic OR anxiety OR burden OR distress OR stress OR well being OR emotion OR emotional) OR MH (mood disorders+ OR depression+ OR anxiety+ OR anxiety disorders+ OR stress, psychological+ OR emotions+ OR mental health+)) AND (TI (mental health service* OR mental healthcare OR therapy OR therapies OR treatment* OR intervention* OR program* OR psychological OR psychologist OR psychoeducation OR psycho-education OR CBT OR health education OR problem solving OR PST OR ACT OR behaviour* OR therapist OR support OR mindful* OR psychotherapy OR relaxation OR meditation OR behavior* OR counseling OR counselling OR cognitive reframing OR cognitive restructuring OR self-help OR self-management OR blended) OR AB (mental health service* OR mental healthcare OR therapy OR therapies OR treatment* OR intervention* OR program* OR psychological OR psychologist OR psychoeducation OR psycho-education OR CBT OR health education OR problem solving OR PST OR ACT OR behaviour* OR therapist OR support OR mindful* OR psychotherapy OR relaxation OR meditation OR behavior* OR counseling OR counselling OR cognitive reframing OR cognitive restructuring OR self-help OR self-management OR blended) OR MH (mental health services+ OR health education+ OR problem solving+ OR psychotherapy+ OR relaxation+ OR self-management+ OR self-help groups+)))

The Cochrane Library

	(((caregiver*):ti,ab,kw OR (care-giver*):ti,ab,kw OR (carer*):ti,ab,kw OR (family):ti,ab,kw OR (families):ti,ab,kw OR (spous*):ti,ab,kw OR (sibling*):ti,ab,kw OR (husband*):ti,ab,kw OR (wife):ti,ab,kw OR (wives):ti,ab,kw OR (partner):ti,ab,kw OR (partners):ti,ab,kw OR (parents):ti,ab,kw OR (parent):ti,ab,kw OR (friend):ti,ab,kw OR (friends):ti,ab,kw OR (relatives):ti,ab,kw OR (relative):ti,ab,kw OR (couples):ti,ab,kw OR (couple):ti,ab,kw OR (mother*):ti,ab,kw OR (father*):ti,ab,kw OR (support NEXT person*):ti,ab,kw OR ("next of kin"):ti,ab,kw OR (significant NEXT other*):ti,ab,kw OR [mh Caregivers] OR [mh Family] OR [mh Spouses] OR [mh Siblings] OR [mh Parents] OR [mh Friends] OR [mh Mothers] OR [mh Fathers]) AND ((cancer):ti,ab,kw OR (tumor):ti,ab,kw OR (tumors):ti,ab,kw OR (tumours):ti,ab,kw OR (tumour):ti,ab,kw OR (neoplasm*):ti,ab,kw OR (oncology):ti,ab,kw OR (carcinoma):ti,ab,kw OR (malignan*):ti,ab,kw OR (melanoma):ti,ab,kw OR (metastasis):ti,ab,kw OR (lymphoma):ti,ab,kw

	OR (leukemia):ti,ab,kw OR (diabetes):ti,ab,kw OR (COPD):ti,ab,kw OR ("chronic obstructive pulmonary disease"):ti,ab,kw OR (COAD):ti,ab,kw OR ("chronic obstructive airway disease"):ti,ab,kw OR ("chronic obstructive lung disease"):ti,ab,kw OR ("chronic airflow obstruction"):ti,ab,kw OR (stroke):ti,ab,kw OR ("cerebrovascular accident"):ti,ab,kw OR (CVA):ti,ab,kw OR ("acute cerebrovascular accident"):ti,ab,kw OR ("brain vascular accident"):ti,ab,kw OR (apoplexy):ti,ab,kw OR ("heart disease"):ti,ab,kw OR ("cardiovascular disease"):ti,ab,kw OR (CVD):ti,ab,kw OR ("ischemic heart disease"):ti,ab,kw OR (IHD):ti,ab,kw OR ("coronary artery disease"):ti,ab,kw OR (CAD):ti,ab,kw OR (angina):ti,ab,kw OR ("myocardial infarction"):ti,ab,kw OR (MI):ti,ab,kw OR ("heart attack"):ti,ab,kw OR ("cardiac event"):ti,ab,kw OR ("cardiac disease"):ti,ab,kw OR (dementia):ti,ab,kw OR (Alzheimer*):ti,ab,kw OR (FTD):ti,ab,kw OR ("Lewy body"):ti,ab,kw OR ("Lewy bodies"):ti,ab,kw OR (neurocognitive NEXT disorder*):ti,ab,kw OR ("mild cognitive impairment"):ti,ab,kw OR (MCI):ti,ab,kw OR (memory NEXT impair*):ti,ab,kw OR ("cognitive decline"):ti,ab,kw OR (chronic NEXT illness*):ti,ab,kw OR (chronic NEXT disease*):ti,ab,kw OR (chronic NEXT condition*):ti,ab,kw OR (multi-morbidit*):ti,ab,kw OR (multimorbidit*):ti,ab,kw OR (long-term NEXT health NEXT condition*):ti,ab,kw OR [mh Neoplasms] OR [mh "Diabetes mellitus"] OR [mh "pulmonary disease, chronic obstructive"] OR [mh stroke] OR [mh "cardiovascular diseases"] OR [mh dementia] OR [mh "cognition disorders"] OR [mh "chronic disease"] OR [mh Multimorbidity]) AND ((eHealth):ti,ab,kw OR (e-health):ti,ab,kw OR ("e-mental health"):ti,ab,kw OR ("emental health"):ti,ab,kw OR (mhealth):ti,ab,kw OR (m-health):ti,ab,kw OR (smartphone):ti,ab,kw OR ("cell phone"):ti,ab,kw OR ("cellular phone"):ti,ab,kw OR ("mobile phone"):ti,ab,kw OR (app):ti,ab,kw OR (application):ti,ab,kw OR (applications):ti,ab,kw OR (apps):ti,ab,kw OR (iPad):ti,ab,kw OR (computer):ti,ab,kw OR (tablet):ti,ab,kw OR (technology):ti,ab,kw OR (technologies):ti,ab,kw OR ("electronic communication"):ti,ab,kw OR (email):ti,ab,kw OR (e-mail):ti,ab,kw OR (text NEXT messag*):ti,ab,kw OR (internet):ti,ab,kw OR (wireless):ti,ab,kw OR (online):ti,ab,kw OR (digital):ti,ab,kw OR (on-line):ti,ab,kw OR (virtual):ti,ab,kw OR (ICT):ti,ab,kw OR (web):ti,ab,kw OR (website):ti,ab,kw OR (multimedia):ti,ab,kw OR (e-learning):ti,ab,kw OR ("online social network"):ti,ab,kw OR (iCBT):ti,ab,kw OR (cCBT):ti,ab,kw OR (e-therap*):ti,ab,kw OR (etherap*):ti,ab,kw OR [mh "cell phone"] OR [mh "mobile application"] OR [mh computers] OR [mh "therapy, computer assisted"] OR [mh "wireless technology"] OR [mh "information technology"] OR [mh technology] OR [mh "electronic mail"] OR [mh internet] OR [mh "online social networking"] OR [mh "virtual reality"] OR [mh "web browser"] OR [mh multimedia]) AND (("mental health"):ti,ab,kw OR (mood):ti,ab,kw OR (depress*):ti,ab,kw OR ("affective disorder"):ti,ab,kw OR ("negative affect"):ti,ab,kw OR (dysthymia):ti,ab,kw OR (dysphoria):ti,ab,kw OR (melancholic):ti,ab,kw OR (anxiety):ti,ab,kw OR (burden):ti,ab,kw OR (distress):ti,ab,kw OR (stress):ti,ab,kw OR ("well being"):ti,ab,kw OR (emotion):ti,ab,kw OR (emotional):ti,ab,kw OR [mh "mood disorders"] OR [mh depression] OR [mh anxiety] OR [mh "anxiety disorders"] OR [mh "stress, psychological"] OR [mh emotions] OR [mh "mental health"]) AND ((mental NEXT health NEXT service*):ti,ab,kw OR ("mental healthcare"):ti,ab,kw OR (therapy):ti,ab,kw OR (therapies):ti,ab,kw OR (treatment*):ti,ab,kw OR (intervention*):ti,ab,kw OR (program*):ti,ab,kw OR (psychological):ti,ab,kw OR (psychologist):ti,ab,kw OR (psychoeducation):ti,ab,kw OR (psycho-education):ti,ab,kw OR (CBT):ti,ab,kw OR ("health education"):ti,ab,kw OR ("problem solving"):ti,ab,kw OR (PST):ti,ab,kw OR (ACT):ti,ab,kw OR (behaviour*):ti,ab,kw OR (therapist):ti,ab,kw OR (support):ti,ab,kw OR (mindful*):ti,ab,kw OR (psychotherapy):ti,ab,kw OR (relaxation):ti,ab,kw OR (meditation):ti,ab,kw OR (behavior*):ti,ab,kw OR (counseling):ti,ab,kw OR (counselling):ti,ab,kw OR ("cognitive reframing"):ti,ab,kw OR ("cognitive restructuring"):ti,ab,kw OR (self-help):ti,ab,kw OR (self-management):ti,ab,kw OR (blended):ti,ab,kw OR [mh "mental health services"] OR [mh "health education"] OR [mh "problem solving"] OR [mh psychotherapy] OR [mh relaxation] OR [mh self-management] OR [mh "self-help groups"]))

EMBASE

Note: The broad search terms used in the search strategy, resulted in the retrieval of a significant number of irrelevant publications in the EMBASE database. Therefore, the search strategy for EMBASE was modified to eliminate key broad terms and when appropriate, replace them with phrases.

#	Searches
1	(caregiver* or care-giver* or carer* or family or families or spous* or sibling* or husband* or wife or wives or partner or partners or parents or parent or friend or friends or relatives or couple or couples or mother* or father* or support person* or next of kin or significant other*).ab,ti.
2	exp caregiver/
3	exp family/
4	exp spouse/
5	exp sibling/
6	exp parent/
7	exp friend/
8	exp mother/
9	exp father/
10	1 or 2 or 3 or 4 or 5 or 6 or 7 or 8 or 9
11	(cancer or tumor or tumors or tumours or tumour or neoplasm* or oncology or carcinoma or malignan* or melanoma or metastasis or lymphoma or leukemia or diabetes or COPD or chronic obstructive pulmonary disease or COAD or chronic obstructive airway disease or chronic obstructive lung disease or chronic airflow obstruction or stroke or cerebrovascular accident or CVA or acute cerebrovascular accident or brain vascular accident or apoplexy or heart disease or cardiovascular disease or CVD or ischemic heart disease or IHD or coronary artery disease or CAD or angina or myocardial infarction or MI or heart attack or cardiac event or cardiac disease or dementia or alzheimer* or FTD or Lewy body or Lewy bodies or neurocognitive disorder* or MCI or mild cognitive impairment or memory impair* or cognitive decline or chronic illness* or chronic disease* or chronic condition* or multi-morbidit* or multimorbidit* or long-term health condition*).ab,ti.
12	exp neoplasm/
13	exp diabetes mellitus/
14	exp chronic obstructive lung disease/
15	exp cerebrovascular accident/
16	exp cardiovascular disease/
17	exp dementia/

18	exp chronic disease/
19	exp multiple chronic conditions/
20	(eHealth or e-health or e-mental health or emental health or mhealth or m-health or smartphone or cell phone or cellular phone or mobile phone or app or apps or iPad or computer or tablet or electronic communication or email or e-mail or text messag* or internet or wireless or online or digital or on-line or virtual or ICT or website or multimedia or e-learning or online social network or iCBT or cCBT or e-therap* or etherap* or web-based or (mobile adj2 technolog*) or (technolog* adj2 solution*) or (technology adj2 based)).ab,ti.
21	exp mobile phone/
22	exp mobile application/
23	exp computer/
24	exp computer assisted therapy/
25	exp wireless communication/
26	exp information technology/
27	exp technology/
28	exp e-mail/
29	exp Internet/
30	exp virtual reality/
31	exp web browser/
32	exp multimedia/
33	20 or 21 or 22 or 23 or 24 or 25 or 26 or 27 or 28 or 29 or 30 or 31 or 32
34	(mental health or mood or depress* or affective disorder or negative affect or dysthymia or dysphoria or melancholic or anxiety or burden or distress or stress or well being or emotion or emotional).ab,ti.
35	exp mood disorder/
36	exp depression/
37	exp anxiety/ or exp anxiety disorder/
38	exp mental stress/
39	exp mental health/
40	(mental health service* or mental healthcare or intervention* or program* or psychological or psychologist or psychoeducation or psycho-education or CBT or health education or problem solving or PST or ACT or behaviour* or therapist or support or mindful* or psychotherapy or relaxation or meditation or behavior* or counseling or counselling or cognitive reframing or cognitive restructuring or self-help or self-management or (group adj therapy) or (psycho* adj therapy) or (cognitive adj2 therapy) or (behavio* adj2 therapy) or (problem adj2 therapy) or (acceptance adj2 therapy) or blended).ab,ti.

41	exp mental health service/
42	exp health education/
43	exp problem solving/
44	exp psychotherapy/
45	exp self help/
46	34 or 35 or 36 or 37 or 38 or 39
47	40 or 41 or 42 or 43 or 44 or 45
48	11 or 12 or 13 or 14 or 15 or 16 or 17 or 18 or 19
49	10 and 33 and 46 and 47 and 48
50	limit 49 to yr="2007 -Current"

PsychINFO

#	Searches
1	(caregiver* or care-giver* or carer* or family or families or spous* or sibling* or husband* or wife or wives or partner or partners or parents or parent or friend or friends or relative or relatives or couple or couples or mother* or father* or support person* or next of kin or significant other*).ab,ti.
2	exp Caregivers/
3	exp Family/
4	exp Spouses/
5	exp Siblings/
6	exp Parents/
7	exp Mothers/
8	exp Fathers/
9	exp Friends/
10	(cancer or tumor or tumors or tumours or tumour or neoplasm* or oncology or carcinoma or malignan* or melanoma or metastasis or lymphoma or leukemia or diabetes or COPD or chronic obstructive pulmonary disease or COAD or chronic obstructive airway disease or chronic obstructive lung disease or chronic airflow obstruction or stroke or cerebrovascular accident or CVA or acute cerebrovascular accident or brain vascular accident or apoplexy or heart disease or cardiovascular disease or CVD or ischemic heart disease or IHD or coronary artery disease or CAD or angina or myocardial infarction or MI or heart attack or cardiac event or cardiac disease or dementia or alzheimer* or FTD or Lewy body or Lewy bodies or neurocognitive disorder* or MCI or mild cognitive impairment or memory impair* or cognitive

	decline or chronic illness* or chronic disease* or chronic condition* or multi-morbidit* or multimorbidit* or long-term health condition*).ab,ti.
11	exp Neoplasms/
12	exp Diabetes Mellitus/
13	exp Chronic Obstructive Pulmonary Disease/
14	exp Stroke/
15	exp Dementia/
16	exp Cognitive Impairment/
17	exp Chronic Illness/
18	exp Comorbidity/
19	exp Cardiovascular Disorders/
20	(eHealth or e-health or e-mental health or emental health or mhealth or m-health or smartphone or cell phone or cellular phone or mobile phone or app or application or applications or apps or iPad or computer or tablet or technology or technologies or electronic communication or email or e-mail or text messag* or internet or wireless or online or digital or on-line or virtual or ICT or web or website or multimedia or e-learning or online social network or iCBT or cCBT or e-therap* or etherap*).ab,ti.
21	exp Mobile Phones/
22	exp Mobile Applications/
23	exp Computers/
24	exp Computer Assisted Therapy/
25	exp Wireless Technologies/
26	exp "Information and Communication Technology"/
27	exp Technology/
28	exp Computer Mediated Communication/
29	exp Internet/
30	exp Online Social Networks/
31	exp Virtual Reality/
32	exp Websites/
33	exp Multimedia/
34	(mental health or mood or depress* or affective disorder or negative affect or dysthymia or dysphoria or melancholic or anxiety or burden or distress or stress or well being or emotion or emotional).ab,ti.
35	exp "Depression (Emotion)"/ or exp Major Depression/

36	exp Anxiety Disorders/ or exp Anxiety/
37	exp Physiological Stress/
38	exp Emotions/
39	exp Mental Health/
40	exp Mood Disorders/
41	(mental health service* or mental healthcare or therapy or therapies or treatment* or intervention* or program* or psychological or psychologist or psychoeducation or psycho-education or CBT or health education or problem solving or PST or ACT or behaviour* or therapist or support or mindful* or psychotherapy or relaxation or meditation or behavior* or counseling or counselling or cognitive reframing or cognitive restructuring or self-help or self-management or blended).ab,ti.
42	exp Mental Health Services/
43	exp Health Education/
44	exp Problem Solving/
45	exp Psychotherapy/
46	exp Relaxation/
47	exp Self-Management/
48	exp Support Groups/
49	1 or 2 or 3 or 4 or 5 or 6 or 7 or 8 or 9
50	10 or 11 or 12 or 13 or 14 or 15 or 16 or 17 or 18 or 19
51	20 or 21 or 22 or 23 or 24 or 25 or 26 or 27 or 28 or 29 or 30 or 31 or 32 or 33
52	34 or 35 or 36 or 37 or 38 or 39 or 40
53	41 or 42 or 43 or 44 or 45 or 46 or 47 or 48
54	49 and 50 and 51 and 52 and 53
55	limit 54 to yr="2007 -Current"

Web of Science

#	
**1**	TI=(caregiver* OR care-giver* OR carer* OR family OR families OR spous* OR sibling* OR husband* OR wife OR wives OR partner OR partners OR parents OR parent OR friend OR friends OR relative OR relatives OR couple OR couples OR mother* OR father* OR support person* OR next of kin OR significant other*)
**2**	AB=(caregiver* OR care-giver* OR carer* OR family OR families OR spous* OR sibling* OR husband* OR wife OR wives OR partner OR partners OR parents OR parent OR friend OR friends OR relative OR relatives OR couple OR couples OR mother* OR father* OR support person* OR next of kin OR significant other*)
**3**	**#1 OR #2**
**4**	TI=(cancer OR tumor OR tumors OR tumours OR tumour OR neoplasm* OR oncology OR carcinoma OR malignan* OR melanoma OR metastasis OR lymphoma OR leukemia OR diabetes OR COPD OR chronic obstructive pulmonary disease OR COAD OR chronic obstructive airway disease OR chronic obstructive lung disease OR chronic airflow obstruction OR stroke OR cerebrovascular accident OR CVA OR acute cerebrovascular accident OR brain vascular accident OR apoplexy OR heart disease OR cardiovascular disease OR CVD OR ischemic heart disease OR IHD OR coronary artery disease OR CAD OR angina OR myocardial infarction OR MI OR heart attack OR cardiac event OR cardiac disease OR dementia OR alzheimer* OR FTD OR Lewy body OR Lewy bodies OR neurocognitive disorder* OR MCI OR mild cognitive impairment OR memory impair* OR cognitive decline OR chronic illness* OR chronic disease* OR chronic condition* OR multi-morbidit* OR multimorbidit* OR long-term health condition*)
**5**	AB=(cancer OR tumor OR tumors OR tumours OR tumour OR neoplasm* OR oncology OR carcinoma OR malignan* OR melanoma OR metastasis OR lymphoma OR leukemia OR diabetes OR COPD OR chronic obstructive pulmonary disease OR COAD OR chronic obstructive airway disease OR chronic obstructive lung disease OR chronic airflow obstruction OR stroke OR cerebrovascular accident OR CVA OR acute cerebrovascular accident OR brain vascular accident OR apoplexy OR heart disease OR cardiovascular disease OR CVD OR ischemic heart disease OR IHD OR coronary artery disease OR CAD OR angina OR myocardial infarction OR MI OR heart attack OR cardiac event OR cardiac disease OR dementia OR alzheimer* OR FTD OR Lewy body OR Lewy bodies OR neurocognitive disorder* OR MCI OR mild cognitive impairment OR memory impair* OR cognitive decline OR chronic illness* OR chronic disease* OR chronic condition* OR multi-morbidit* OR multimorbidit* OR long-term health condition*)
**6**	**#4 OR #5**
**7**	TI=(eHealth OR e-health OR e-mental health OR emental health OR mhealth OR m-health OR smartphone OR cell phone OR cellular phone OR mobile phone OR app OR application OR applications OR apps OR iPad OR computer OR tablet OR technology OR technologies OR electronic communication OR email OR e-mail OR text messag* OR internet OR wireless OR online OR digital OR on-line OR virtual OR ICT OR web OR website OR multimedia OR e-learning OR online social network OR iCBT OR cCBT OR e-therap* OR etherap*)
**8**	AB=(eHealth OR e-health OR e-mental health OR emental health OR mhealth OR m-health OR smartphone OR cell phone OR cellular phone OR mobile phone OR app OR application OR applications OR apps OR iPad OR computer OR tablet OR technology OR technologies OR electronic communication OR email OR e-mail OR text messag* OR internet OR wireless OR online OR digital OR on-line OR virtual OR ICT OR web OR website OR multimedia OR e-learning OR online social network OR iCBT OR cCBT OR e-therap* OR etherap*)
**9**	**#7 OR #8**

**10**	TI=(mental health OR mood OR depress* OR affective disorder OR negative affect OR dysthymia OR dysphoria OR melancholic OR anxiety OR burden OR distress OR stress OR well being OR emotion OR emotional)
**11**	AB=(mental health OR mood OR depress* OR affective disorder OR negative affect OR dysthymia OR dysphoria OR melancholic OR anxiety OR burden OR distress OR stress OR well being OR emotion OR emotional)
**12**	**#10 OR #11**
**13**	TI=(mental health service* OR mental healthcare OR therapy OR therapies OR treatment* OR intervention* OR program* OR psychological OR psychologist OR psychoeducation OR psycho-education OR CBT OR health education OR problem solving OR PST OR ACT OR behaviour* OR therapist OR support OR mindful* OR psychotherapy OR relaxation OR meditation OR behavior* OR counseling OR counselling OR cognitive reframing OR cognitive restructuring OR self-help OR self-management OR blended)
**14**	AB=(mental health service* OR mental healthcare OR therapy OR therapies OR treatment* OR intervention* OR program* OR psychological OR psychologist OR psychoeducation OR psycho-education OR CBT OR health education OR problem solving OR PST OR ACT OR behaviour* OR therapist OR support OR mindful* OR psychotherapy OR relaxation OR meditation OR behavior* OR counseling OR counselling OR cognitive reframing OR cognitive restructuring OR self-help OR self-management OR blended)
**15**	**#13 OR #14**
**16**	**#3 AND #6 AND # 9 AND #12 AND #15**
