# Supplementary material for: Implementation of e-mental health interventions for informal caregivers of adults with chronic diseases: a protocol for a mixed-methods systematic review with a qualitative comparative analysis
Source: BMJ Open. 2020 Jun 21;10(6):e035406. doi: 10.1136/bmjopen-2019-035406 (PMC7307546; doi:10.1136/bmjopen-2019-035406)
Supplement: Supplementary data [file bmjopen-2019-035406supp004.pdf]

Implementation of e-mental health interventions for informal caregivers of adults with chronic diseases: a protocol for a mixed methods systematic review with a qualitative comparative analysis

Supplementary File 4: Data Extraction Form

<table>
<tr><th colspan="3">Study Identification Features</th></tr>
<tr><td>Unique Study Identifier</td><td colspan="2"></td></tr>
<tr><td>Title</td><td colspan="2"></td></tr>
<tr><td>First Author</td><td colspan="2"></td></tr>
<tr><td>Year of Publication</td><td colspan="2"></td></tr>
<tr><td>Country of Study</td><td colspan="2"></td></tr>
<tr><td>Funding Source</td><td colspan="2"></td></tr>
<tr><td>Protocol Available?</td><td colspan="2"></td></tr>
<tr><th colspan="3">Study Characteristics</th></tr>
<tr><td>Aims and Objectives</td><td colspan="2"></td></tr>
<tr><td>Study Design (e.g. RCT, qualitative)</td><td colspan="2"></td></tr>
<tr><td>Inclusion/Exclusion Criteria</td><td colspan="2"></td></tr>
<tr><td>Recruitment Setting and Method</td><td colspan="2"></td></tr>
<tr><td rowspan="3">Randomization</td><td>Sequence Generation</td><td></td></tr>
<tr><td>Type</td><td></td></tr>
<tr><td>Allocation Concealment</td><td></td></tr>
<tr><td rowspan="2">Blinding</td><td>Data Collectors</td><td></td></tr>
<tr><td>Data Analysts</td><td></td></tr>
<tr><th colspan="3">Caregiver Characteristics</th></tr>
<tr><td>Caregiver Sample Size (total and per arm)</td><td colspan="2"></td></tr>
<tr><td>Age</td><td colspan="2"></td></tr>
<tr><td>Gender</td><td colspan="2"></td></tr>
<tr><td>Severity of Anxiety at Baseline</td><td colspan="2"></td></tr>
<tr><td>Severity of Depression at Baseline</td><td colspan="2"></td></tr>
<tr><td>Severity of Distress at Baseline</td><td colspan="2"></td></tr>
<tr><td>Severity of Stress at Baseline</td><td colspan="2"></td></tr>
<tr><td>Relationship to Care Recipient</td><td colspan="2"></td></tr>
<tr><td>Amount of Care Provided</td><td colspan="2"></td></tr>
<tr><td>Length of Time as a Caregiver</td><td colspan="2"></td></tr>
<tr><td>Education Level</td><td colspan="2"></td></tr>
<tr><td>Employment Status</td><td colspan="2"></td></tr>
<tr><th colspan="3">Care Recipient Characteristics</th></tr>
<tr><td>Sample Size (total and per arm)</td><td colspan="2"></td></tr>
<tr><td>Diagnosis</td><td colspan="2"></td></tr>
<tr><td>Age</td><td colspan="2"></td></tr>
<tr><td>Gender</td><td colspan="2"></td></tr>
<tr><td>Severity Measure of Physical Health Condition of Care Recipient</td><td colspan="2"></td></tr>
<tr><th colspan="3">Intervention</th></tr>
</table>

Summary of Intervention	
Summary of Control Condition	
Type of Therapy (e.g. psychoeducation, CBT)	
Format of Intervention (e.g. web, mobile app)	
Individual or Dyadic Intervention	
Other Support Provided (e.g. some face to face contact)	
Provider (e.g. lay worker, psychologist)	
Training provided to intervention provider (yes/no, describe if yes)	
Total Length of Treatment	
Number of Sessions	
Length of Sessions	
Adherence (did participants receive intended intervention, did they complete entire intervention)	
**Implementation Outcomes**	
Implementation Outcomes Measured or Described	
Measurement Tools Used	
Timing of Measurements	
**Mental Health Outcome Measurements**	
Mental Health Outcome Measurements (primary and secondary)	
Quality of Outcome Measurements	
Timing of Measurements	
**Statistical Techniques**	
Power Calculation	
Target Sample Size	
Method of Dealing with Missing Data	
Baseline Comparability	
**Participant Flow**	
Eligible Participants	
Randomised to Intervention	
Randomised to Control	
Lost to Follow-Up Intervention[1]	
Lost to Follow-Up Control[1]	
Analysed Intervention[2]	
Analysed Control[2]	
**Results Summary**	
Summary of Mental Health Outcome Results	
Summary of Implementation Results	
**Quantitative Results[3]**	

Intervention	Outcome Measurement	
	Measurement Scale Used	
	Analysis Method (ITT, per protocol)	
	Pre-Treatment Means	
	Pre-Treatment Standard Deviation	
	Pre-Treatment Number Analysed	
	Post-Treatment Means	
	Post-Treatment Standard Deviation	
	Post-Treatment Number Analysed	
Control	Outcome Measurement	
	Measurement Scale Used	
	Analysis Method (ITT, per protocol)	
	Pre-Treatment Means	
	Pre-Treatment Standard Deviation	
	Pre-Treatment Number Analysed	
	Post-Treatment Means	
	Post-Treatment Standard Deviation	
	Post-Treatment Number Analysed	
**Additional Comments**		

[1]Numbers lost to follow-up to be reported at each time point measured; [2]Numbers analysed to be reported for each time point measured; [3]Results to be extracted for outcomes of anxiety, depression, distress and/or stress at all follow-up time points
