# Supplementary material for: Implementation of e-mental health interventions for informal caregivers of adults with chronic diseases: a protocol for a mixed-methods systematic review with a qualitative comparative analysis
Source: BMJ Open. 2020 Jun 21;10(6):e035406. doi: 10.1136/bmjopen-2019-035406 (PMC7307546; doi:10.1136/bmjopen-2019-035406)
Supplement: Supplementary data [file bmjopen-2019-035406supp005.pdf]

Implementation of e-mental health interventions for informal caregivers of adults with chronic diseases: a protocol for a mixed methods systematic review with a qualitative comparative analysis

Supplementary File 5: Sample data table

Study	Conditions[a]				Outcome	
	Lay-led	Mobile app	Supplemented with personal contact	Implementing organization involved in project	Effect size (raw Hedges' g)	Effective intervention[b]
Study A	0	0	1	1	0.25	0
Study B	1	0	1	0	0.5	1

[a]The conditions shown here are examples of conditions that could be explored in the qualitative comparative analysis. These are subject to change as the conditions selected depend on the features and heterogeneity among studies included in the review. The number 1 signifies that the study belongs to the indicated set. For example Study A was not lay led or a mobile app, but it did contain personal contact and the implementing organization was involved in the project.

[b]Assuming an effective intervention is defined as having a Hedges' g effect size ≥ 0.3. In this example Study A has an effect size below this cut-off, therefore, is considered not effective.
