# Supplementary material for: Implementation of e-mental health interventions for informal caregivers of adults with chronic diseases: a protocol for a mixed-methods systematic review with a qualitative comparative analysis
Source: BMJ Open. 2020 Jun 21;10(6):e035406. doi: 10.1136/bmjopen-2019-035406 (PMC7307546; doi:10.1136/bmjopen-2019-035406)
Supplement: Author's manuscript [file bmjopen-2019-035406.draft_revisions.pdf]

BMJ Open is committed to open peer review. As part of this commitment we make the peer review history of every article we publish publicly available.

When an article is published we post the peer reviewers' comments and the authors' responses online. We also post the versions of the paper that were used during peer review. These are the versions that the peer review comments apply to.

The versions of the paper that follow are the versions that were submitted during the peer review process. They are not the versions of record or the final published versions. They should not be cited or distributed as the published version of this manuscript.

BMJ Open is an open access journal and the full, final, typeset and author-corrected version of record of the manuscript is available on our site with no access controls, subscription charges or pay-per-view fees (http://bmjopen.bmj.com).

If you have any questions on BMJ Open's open peer review process please email info.bmjopen@bmj.com

BMJ Open

Implementation of e-mental health interventions for informal caregivers of adults with chronic diseases: a protocol for a mixed methods systematic review with a qualitative comparative analysis

Journal:	*BMJ Open*
Manuscript ID	bmjopen-2019-035406
Article Type:	Protocol
Date Submitted by the Author:	30-Oct-2019
Complete List of Authors:	Coumoundouros, Chelsea; Uppsala University, Clinical Psychology in Healthcare, Department of Women's and Children's Health<br>von Essen, Louise; Uppsala University, Clinical Psychology in Healthcare, Department of Women's and Children's Health<br>Sanderman, Robbert; University of Groningen, University Medical Center Groningen, Department of Health Psychology; University of Twente, Department of Psychology, Health and Technology<br>Woodford, Joanne; Uppsala University, Clinical Psychology in Healthcare, Department of Women's and Children's Health
Keywords:	MENTAL HEALTH, World Wide Web technology < BIOTECHNOLOGY & BIOINFORMATICS, PRIMARY CARE, Depression & mood disorders < PSYCHIATRY, Anxiety disorders < PSYCHIATRY

SCHOLARONE™
Manuscripts

I, the Submitting Author has the right to grant and does grant on behalf of all authors of the Work (as defined in the below author licence), an exclusive licence and/or a non-exclusive licence for contributions from authors who are: i) UK Crown employees; ii) where BMJ has agreed a CC-BY licence shall apply, and/or iii) in accordance with the terms applicable for US Federal Government officers or employees acting as part of their official duties; on a worldwide, perpetual, irrevocable, royalty-free basis to BMJ Publishing Group Ltd ("BMJ") its licensees and where the relevant Journal is co-owned by BMJ to the co-owners of the Journal, to publish the Work in this journal and any other BMJ products and to exploit all rights, as set out in our licence.

The Submitting Author accepts and understands that any supply made under these terms is made by BMJ to the Submitting Author unless you are acting as an employee on behalf of your employer or a postgraduate student of an affiliated institution which is paying any applicable article publishing charge ("APC") for Open Access articles. Where the Submitting Author wishes to make the Work available on an Open Access basis (and intends to pay the relevant APC), the terms of reuse of such Open Access shall be governed by a Creative Commons licence – details of these licences and which Creative Commons licence will apply to this Work are set out in our licence referred to above.

Other than as permitted in any relevant BMJ Author's Self Archiving Policies, I confirm this Work has not been accepted for publication elsewhere, is not being considered for publication elsewhere and does not duplicate material already published. I confirm all authors consent to publication of this Work and authorise the granting of this licence.

For peer review only

Implementation of e-mental health interventions for informal caregivers of adults with chronic diseases: a protocol for a mixed methods systematic review with a qualitative comparative analysis

Chelsea COUMOUNDOUROS[1] (chelsea.coumoundouros@kbh.uu.se), Louise VON ESSEN[1] (louise-von.essen@kbh.uu.se), Robbert SANDERMAN[2, 3] (r.sanderman@umcg.nl), Joanne WOODFORD[1] (joanne.woodford@kbh.uu.se)

Corresponding author

Chelsea Coumoundouros, Clinical Psychology in Healthcare, Department of Women's and Children's Health, Uppsala University, 751 23 Uppsala, Sweden. E-mail: chelsea.coumoundouros@kbh.uu.se Telephone: 0046 729 999 211

Affiliations

[1]Clinical Psychology in Healthcare, Department of Women's and Children's Health, Uppsala University, 751 23 Uppsala, Sweden.

[2]Department of Health Psychology, University Medical Center Groningen, University of Groningen, 9713 AV, Groningen, the Netherlands.

[3]Department of Psychology, Health and Technology, University of Twente, Enschede, the Netherlands

Word count: 3691

For peer review only

ABSTRACT

Introduction. Informal caregivers provide the majority of care to individuals with chronic health conditions, benefiting the care recipient and reducing use of formal care services. However, providing informal care negatively impacts the mental health of many caregivers. E-mental health interventions have emerged as a way to provide accessible mental healthcare to caregivers. Much attention has been given to reviewing the effectiveness and efficacy of such interventions, however, factors related to implementation have received less consideration. Therefore, this mixed methods systematic review will aim to examine factors associated with the effectiveness and implementation of e-mental health interventions for caregivers.

Methods and analysis. Eligible studies published since 2007 will be searched for in several electronic databases (CINAHL Plus with Full Text, the Cochrane Library, EMBASE, PsychINFO, PubMed, and Web of Science), clinical trial registries and OpenGrey, with all screening steps conducted by two independent reviewers. Studies will be included if they focus on the implementation or effectiveness of e-mental health interventions designed for informal adult caregivers of adults with cancer, heart disease, stroke, diabetes, dementia or chronic obstructive pulmonary disease. Pragmatic randomized controlled trials quantitatively reporting on caregiver anxiety, depression, distress or stress will be used for a qualitative comparative analysis to identify combinations of conditions that result in effective interventions. Qualitative and quantitative data on implementation of e-mental health interventions for caregivers will be integrated in a thematic synthesis to identify barriers and facilitators to implementation. These results will inform future development and implementation planning of e-mental health interventions for caregivers.

Ethics and dissemination. Ethical approval is not required for this study as no primary data will be collected. Results will be disseminated in the form of a scientific publication and presentations at academic conferences and plain language summaries for various stakeholders.

PROSPERO registration number: CRDXXX

For peer review only

STRENGTHS AND LIMITATIONS OF THIS STUDY

- The mixed method design of this review will ensure a wide variety of data on implementation is captured
- The peer-reviewed, comprehensive search strategy with all selection steps completed by two independent reviewers will ensure a thorough search of the literature and reduce bias in study selection
- High heterogeneity across studies in terms of implementation or intervention features is easily accommodated in a qualitative comparative analysis
- Crisp set qualitative comparative analysis will make results of this review more concrete and usable for healthcare professionals and decision-makers
- However, crisp set qualitative comparative analysis dichotomizes all variables including the outcome, therefore, a more detailed understanding of the strength of the effect size may be lost in this process

INTRODUCTION

Informal caregivers (hereafter referred to as caregivers) are family members or friends who provide unpaid support and care to individuals with healthcare needs. Caregivers play a vital societal role in healthcare systems worldwide, providing up to 80% of care to individuals with long-term care needs.[1] Informal care provision can include emotional support, assistance with household tasks (e.g. cooking, cleaning), medical care, transportation, managing finances and advocacy on behalf of the care recipient.[2,3] Demand for caregivers is expected to increase in the future as the proportion of older adults in populations around the world increases and healthcare policies favour deinstitutionalization and outpatient care.[4,5] As societal dependence on informal care continues to grow, it is becoming increasingly important to implement programs and policies to support individuals who become caregivers.[2,6]

Caregivers can experience both positive and negative outcomes over the course of their time as a caregiver.[3,7,8] Caregiving can lead to an improved relationship between caregiver and care recipient, feelings of personal development and a sense of accomplishment related to obtaining skills and recognizing the impact of the care they provide.[8] However, caregivers may also experience negative outcomes related to the caregiving role, such as financial strain and poor physical and mental health.[3,7,9] Indeed, the rate of depression and anxiety among caregivers exceeds that of the general population.[10] The prevalence of depressive symptoms in cancer and stroke caregivers is often above 40% and the prevalence of anxiety ranges from 21% to over 40%.[11,12] Mental health problems can result in large personal and societal costs related to increased morbidity and reduced productivity.[13–15] Additionally, poor caregiver mental health negatively impacts distress levels in the care recipient[16,17] and the quality of care provided by the caregiver.[18–21]

There is a clear need to develop effective interventions and resources to prevent or reduce the mental health burden experienced by caregivers. However, caregivers have reported various barriers to accessing mental health services such as lack of knowledge regarding available services, financial barriers, stigma and prioritization of the caregiving role over self-care.[22] E-

For peer review only

health technologies have emerged as an accessible way to provide support and information to caregivers[23–25] and can be designed to achieve various goals such as, improving communication, teaching skills or reducing depression.[26,27] Numerous systematic reviews and meta-analyses have examined e-health interventions for caregivers of adults with chronic health conditions, showing the potential for e-health interventions to improve caregiver well-being.[23,25,28–40]

E-mental health interventions, that is, mental health interventions delivered via the internet or using mobile technologies,[27,41,42] represent a subset of e-health interventions. E-mental health solutions offer a means to improve mental health service access globally[43,44] by eliminating many barriers to mental health service access (e.g. transportation, stigma, time)[25,42,45] and are often more cost-effective than traditional therapies.[42,45] Meta-analyses show reductions in caregiver's depression and anxiety in response to e-mental health interventions.[37,39] However, as many reviews focus on intervention efficacy and effectiveness,[23,28–32,34,36–40,46] gaps remain in our understanding of factors related to the intervention and the implementation context that make e-mental health interventions effective among caregivers.

Wider literature suggests that the implementation of e-health programs in real-world settings often encounters many barriers,[24,33,47,48] preventing effective interventions from being made available to those who need them. Few reviews have examined factors related to implementation of e-health interventions for caregivers,[33,35] with no current reviews, to the best of our knowledge, focusing on implementation of e-mental health interventions for caregivers exclusively. Evaluating the implementation of an intervention is essential to gain insights into why interventions succeed or fail when put into practice. Factors influencing implementation can be related to the intervention itself, the participants, the implementation setting and wider societal factors (e.g. regional policies).[49] Additionally, although efficacy (also referred to as explanatory) trials are a useful tool to establish the beneficial effects of an intervention under ideal settings, trials with a more pragmatic design may be better suited to investigating factors potentially associated with implementation given real-world conditions are more closely reflected in pragmatic trials.[50,51] Commonly, systematic reviews and meta-analyses do not distinguish between pragmatic and explanatory trials despite the different conditions (e.g. setting, recruitment methods, eligibility criteria, control of adherence to and delivery of the intervention) under which interventions are evaluated.[50,52]

The aim of this review is to examine factors related to the effectiveness and implementation of e-mental health interventions for caregivers of adults with chronic diseases. Two approaches will be used to investigate this. First, studies with more pragmatic designs will be used exclusively to determine which combinations of intervention or implementation characteristics are associated with effectiveness using a qualitative comparative analysis. Second, reports regarding the implementation of e-mental health interventions will be thematically synthesized to establish the common barriers and facilitators to e-mental health implementation. Findings from this review can be used to guide the development of effective e-mental health interventions to support caregivers and ensure the successful implementation of these interventions within real-world healthcare settings.

For peer review only - http://bmjopen.bmj.com/site/about/guidelines.xhtml

METHODS AND ANALYSIS

This mixed method review protocol has been registered with PROSPERO with registration number CRDXXX and any protocol amendments will be recorded in PROSPERO. The Preferred Reporting Items for Systematic Reviews and Meta-Analysis Protocols (PRISMA-P) checklist[53] (online supplementary appendix 1) and the Joanna Briggs methodology for mixed methods systematic reviews[54] were used to guide the development of this protocol.

Study eligibility criteria

The eligibility criteria used to inform study inclusion and exclusion are outlined using PICOS (population, interventions, comparators, outcomes and study design).[55,56]

Population

Unpaid adult caregivers (aged 18 years or older) of adults with either cancer, chronic obstructive pulmonary disease (COPD), dementia, diabetes, heart disease or stroke. Care recipient's chronic health conditions eligible for inclusion were selected as, globally, they are the largest sources of disability adjusted life years due to physical chronic diseases in adults[57] and often require informal care.[58] No restrictions will be placed on the frequency or amount of care provided for someone to be considered a caregiver. Studies focusing on caregivers with severe mental health conditions (e.g. psychosis or bipolar disorder), caregivers providing care to non-community dwelling care recipients or care recipients at the palliative phase of disease will be excluded.

Interventions

Interventions will utilise internet technology, which can include mobile applications, to deliver a mental health intervention to caregivers.[27,41] E-mental health interventions can encompass many types of mental health support such as screening, prevention, treatment or service delivery.[41] This review will focus on interventions targeting the treatment of common caregiver mental health difficulties such as depression and anxiety. This can include any type of mental health treatment, including psychoeducation. The e-mental health intervention may be supplemented with additional forms of support (such as telephone contact, face-to-face support or video-conferencing), however, the majority of the intervention must be internet based. Interventions delivered via telephone, CD-ROM or video (including Skype) alone will be excluded.

Comparators

As it is necessary to determine effect sizes for the qualitative comparative analysis,[59] only studies with non-active controls will be included in this analysis. Non-active controls include: no treatment, wait-list control, treatment as usual, non-specific treatment component control (e.g. control for attention) or education on the care recipient's condition.[60] Studies using psychoeducation or active controls (e.g. controls using specific treatment components or studies comparing two therapies) will be excluded.

For thematic synthesis of barriers and facilitators to implementation, uncontrolled or controlled studies, with no restrictions based on control type, will be included in the analysis.

For peer review only - http://bmjopen.bmj.com/site/about/guidelines.xhtml

Outcomes

For the qualitative comparative analysis, studies must report on caregiver mental health outcomes, specifically anxiety, depression, psychological distress or stress, measured using an instrument with at least acceptable reliability (Cronbach's alpha ≥ 0.7).[61] Examples of eligible measurement instruments include the Center for Epidemiologic Studies – Depression Scale,[62] the Hospital Anxiety and Depression Scale[63] or the Perceived Stress Scale.[64]

For the thematic synthesis, studies will report on barriers and/or facilitators to intervention implementation. This may include qualitative (e.g. interviews or focus groups) or quantitative (e.g. Normalization Measure Development questionnaire[65]) data. Barriers or facilitators can include factors related to any aspect of the Consolidated Framework for Implementation Research[49] or the implementation outcome framework developed by Proctor and colleagues.[66] The Consolidated Framework for Implementation Research consists of 5 domains related to implementation, namely (1) intervention characteristics (e.g. adaptability, complexity); (2) outer setting (e.g. external policies, patient needs and resources); (3) inner/implementation setting (e.g. culture within the organization, readiness for implementation); (4) characteristics of individuals (e.g. self-efficacy, individual stage of change); and (5) process (e.g. planning, engaging).[49] The implementation outcome framework broadly classifies measurable implementation outcomes which includes acceptability, adoption, feasibility, fidelity, reach, appropriateness, implementation cost and sustainability.[66]

Study designs

Studies included for the qualitative comparative analysis must be pragmatic randomized controlled trials (also referred to as effectiveness trials). Pragmatic trials will be identified using the validated PRagmatic Explanatory Continuum Indicator Summary 2 (PRECIS-2) tool.[50] PRECIS-2 was developed with input from clinicians, researchers and policymakers to allow trialists to assess how pragmatic or explanatory their trial design is across nine domains: eligibility criteria, recruitment, setting, organization, flexibility (delivery), flexibility (adherence), follow-up, primary outcome and primary analysis.[50] Trials with a pragmatic design will be defined as any trial with a mean score of 3 or higher using the PRECIS-2 tool.[50] PRECIS-2 has been used with this cut-off score to categorize studies in another systematic review,[67] although to our knowledge it has not previously been used to exclude studies from a systematic review. Using a cut-off score of 3 should ensure generous inclusion of trials containing at least a mixture of pragmatic and explanatory design features.[50]

To assess barriers and facilitators to implementation, any study type with quantitative and/or qualitative data will be eligible for inclusion.

Search strategy

Comprehensive literature searches will be conducted in multiple electronic databases (CINAHL Plus with Full Text, the Cochrane Library, EMBASE, PsychINFO, PubMed and Web of Science). Clinical trial registries (www.clinicaltrials.gov and www.who.int/trialsearch/) will be searched for relevant completed clinical trials and the resulting publications will be found and

For peer review only

screened for inclusion. Searches for grey literature will be performed using OpenGrey (http://www.opengrey.eu/), a database of grey literature in Europe such as research reports and conference papers.

The search strategy was developed in consultation with Agnes Kotka, a librarian at Uppsala University and was reviewed by Professor Mariët Hagedoorn and Truus van Ittersum (University Medical Centre Groningen, University of Groningen) and Dr. Nathan Davies (University College London) following the PRESS peer review guidelines[68] (online supplementary appendix 2). The search was constructed using terms related to (1) caregivers; (2) the chronic health conditions of interest (cancer, COPD, dementia, diabetes, heart disease and stroke); (3) e-health/information and communication technology; (4) mental health; and (5) psychological therapies (see online supplementary appendix 3). Included terms were informed by existing reviews focusing on the population and/or intervention of interest to this review.[9,33,39,69–74] Search terms were refined based on feedback from the peer-review process, resulting in the addition of more truncations to search terms, elimination of repetitive search terms that did not retrieve additional records and the addition of an abbreviation missed prior to the peer-review process. The search will include relevant Medical Subject Headings (MeSH) when possible and terms will be searched for in the title/abstract of publications. Included studies will be restricted to those published in English, Dutch, German or Swedish. Literature produced from January 2007 onwards will be eligible for inclusion. Technologies from work published prior to 2007 may be outdated and other reviews have shown that production of publications involving e-health began to rise from 2007 onwards.[33,35]

Upon final inclusion of any studies, their references, results from forward citation searches and from the first three pages of the "find similar" search function in PubMed will be used to check for any additional studies of interest. Experts in the field will be contacted to identify further studies for inclusion.

Study selection

Results of database searches will be imported into EndNote for deduplication following the procedures outlined by Bramer et al.[75] Remaining records will be imported into the online screening software Rayyan.[76] Titles, abstracts and full-texts will be screened independently by two reviewers. Conflicts will be discussed and a third reviewer will be consulted if consensus cannot be reached. Study selection will be based on the criteria outlined by the PICOS, with reasons for study exclusion being recorded at the full-text screening stage. Full-texts will be checked against each sub-section of the PICOS, recording which sub-sections are or are not met by each study, with an overall reason for exclusion being reported in the PRISMA flow-diagram. This will facilitate detailed discussions regarding study exclusion when conflicts arise. If studies do not contain enough information to decide upon inclusion, the original authors will be contacted at most twice over a one month period to obtain information to determine study eligibility. If the original authors do not respond, the study will be excluded. Abstracts, theses, books, commentaries, editorials and letters to the editor will be excluded. Reviews and study protocols will also be excluded, however, the references of related reviews will be checked for additional studies of interest, published results of relevant study protocols will be obtained and if

For peer review only

protocol results are unpublished, authors will be contacted to determine whether access to unpublished results is possible.

Exclusion of studies on the basis of adopting a more explanatory, as opposed to pragmatic, trial design will be conducted as a final step during the full text screening process. This screening step will only be applied to trials eligible for the qualitative comparative analysis. Studies will be scored using the PRECIS-2 tool by two independent reviewers and studies with a mean score below 3 will be excluded.[50,67]

Assessment of methodological quality

Methodological quality of studies included in the qualitative comparative analysis will be evaluated using the Cochrane Risk of Bias 2.0 tool for randomized controlled trials[77,78]. This evaluation will facilitate the identification of selection, performance, measurement, attrition and reporting bias.[78] Authors will be contacted if more information is required to complete the quality assessment. Reporting bias will be explored by comparing outcomes measures described in study protocols to the outcome measures reported in the methods and results sections of the corresponding completed trial. In response to any identified inconsistencies, authors will be contacted to determine potential causes of this. Study assessment will be conducted by two independent reviewers, followed by discussion of any discrepancies, consulting a third reviewer as needed. Studies will not be excluded based on methodological quality, however the results of the Cochrane Risk of Bias 2.0 evaluation will be reported descriptively.

Data extraction

Data from included full-texts will be extracted into Microsoft Excel (2016), using a data extraction form developed for this review based on the Centre for Reviews and Dissemination guidelines.[56] Extracted information will include data pertaining to study participants, study design, the intervention and relevant outcomes (full details in online supplementary appendix 4). Data will be extracted independently by two reviewers, with resulting extractions compared for accuracy and completion. If conflicts arise, the original publication will be referred to in order to resolve misunderstandings and a third reviewer will be consulted if necessary. Authors will be contacted at most twice to obtain additional data and/or clarification as needed. Qualitative results pertaining to implementation will be transferred into NVivo 10 software[79] for thematic synthesis.

Data synthesis

Two analysis methods will be used. Pragmatic randomized controlled trials with quantitative mental health outcome data will be included in the qualitative comparative analysis. Publications of any study design reporting on implementation will be included in the thematic synthesis, taking an integrative approach to synthesize both qualitative and quantitative findings.

Qualitative comparative analysis

A crisp set qualitative comparative analysis will be conducted to determine sets of conditions that result in effective e-mental health programs for caregivers.[59] Crisp set qualitative comparative

For peer review only

analysis involves dichotomizing outcome data (e.g. effective or not effective) and conditions (e.g. present or absent) selected for inclusion in the analysis into distinct categories.[59] A crisp set analysis approach was selected over a fuzzy set analysis as the results will be more clearly interpretable and easier for decision-makers to use.[80]

The first step of a qualitative comparative analysis is to build a data table containing information regarding the effectiveness of each study and conditions related to the intervention and its implementation (see online supplementary appendix 5).[59] Conditions to include in the data table will be based on important factors related to intervention components (e.g. uses goal-setting, homework), intervention delivery methods (e.g. mobile app, computer), and implementation (e.g. acceptability, feasibility). By restricting this analysis to pragmatic trials, which are designed to more closely reflect real-world settings, implementation conditions are more likely to be reported. Conditions selected will be adjusted given the need to ensure adequate heterogeneity is present.[59] Qualitative comparative analysis requires diversity among studies in terms of conditions present and intervention effectiveness in order to determine the combination of factors sufficient for interventions to be effective.[59] Therefore, adjustments to outcome classification and conditions selected for analysis will be needed after data collection is completed.

Intervention effectiveness will be measured as the standardized mean effect size between control and comparator groups' mental health outcomes, calculated using Hedges' g and the Comprehensive Meta-Analysis (version 3) software. Effect sizes will be calculated for all mental health outcomes of interest for this review (anxiety, depression, distress and stress) and will be based on data collected immediately after intervention completion. If enough studies report subsequent post-intervention follow-ups, these effect sizes will be calculated to explore whether different factors contribute to sustained intervention success. Effect sizes will be used to create crisp sets to categorize studies as effective (Hedges' $g \geq 0.3$) or not effective.[81] If most interventions are effective (or not effective), a different classification system will be created to ensure adequate heterogeneity for analysis,[59] for example categorizing studies as highly effective (Hedges' $g \geq 0.5$) or not highly effective.[81] Proposed cut-offs were developed based on existing meta-analyses of e-mental health interventions.[82–85]

The main data table will use general effectiveness as the outcome measure, meaning the primary mental health outcome as identified in each study will be used to represent the effectiveness of that intervention. If studies include multiple outcomes of interest, but do not identify a primary outcome measure, the outcome most frequently measured in included studies will be used to evaluate intervention effectiveness. Secondary analyses may be conducted for anxiety, depression, distress and stress separately, to explore whether different conditions are more important for different outcome measures, however, this is dependent upon identification of an adequate number of studies for each outcome of interest. After completion of the data table, truth tables will be constructed and the software fs/QCA (version 3.1b) will be used to determine the sufficient conditions for effective e-mental health interventions.[86,87]

For peer review only

Thematic synthesis

Data from studies addressing implementation of e-mental health interventions for caregivers will be thematically synthesized using a deductive coding approach, to identify barriers and facilitators experienced during implementation.[88,89] It will likely be necessary to integrate qualitative and quantitative data as many aspects of implementation such as acceptability, feasibility and usability, may be measured using quantitative tools.[90] First, qualitative data will be thematically analyzed using the Consolidated Framework for Implementation Research to guide coding.[49] This framework was selected a priori as it was developed by combining multiple implementation theories into a single, comprehensive theory covering all aspects related to implementation[49] and it has been used as a coding guide in other reviews on implementation.[33,91] Qualitative data will be coded based on the 39 pre-defined constructs within the Consolidated Framework for Implementation Research.[49]

Quantitative data will be narratively summarized to facilitate subsequent integration of qualitative and quantitative findings. Creating narrative summaries will involve approaches such as textually describing study findings and grouping findings based on the constructs and domains of the Consolidated Framework for Implementation Research.[89] Initially, 10% of full-texts included in the thematic synthesis will be coded independently by two reviewers, followed by discussion of the coding process in consultation with a third reviewer. The remaining coding will be conducted by one reviewer with regular discussions with a second reviewer, involving a third reviewer as needed. Results of the qualitative and quantitative analyses will then be integrated to synthesize higher level themes representing the key barriers and facilitators to the implementation of e-mental health interventions within each domain of the Consolidated Framework for Implementation Research. This final synthesis step will involve at least two reviewers to ensure themes accurately reflect the results obtained from the individual studies.

Patient and public involvement

There was no patient or public involvement in the development of this protocol. However, caregivers and healthcare professionals will be involved in the interpretation of data for the thematic synthesis.[92,93] Results of the thematic synthesis will be presented to a multidisciplinary group of stakeholders (e.g. caregivers, mental health service providers) to explore whether the identified themes resonate with their experiences or if they feel important aspects related to implementation have not been captured by the synthesis. Their perspectives will be incorporated into the interpretation of the results of the thematic synthesis.

DISCUSSION

Despite the importance of implementation planning and need for effective e-mental health interventions for caregivers in real world practice, there have been no reviews focusing on this area. Using pragmatic trials and implementation research, this review will identify both the key characteristics of effective interventions and barriers and facilitators to implementation. A qualitative comparative analysis will be employed to identify combinations of conditions resulting in effective e-mental health interventions for caregivers, a method which, to the best of our knowledge, has not yet been used in this field. The results of the qualitative comparative

For peer review only

analysis can be used to improve the design of future e-mental health interventions by identifying intervention components and implementation factors important to intervention effectiveness in real-world settings.

Additionally, common barriers and facilitators to implementation of e-mental health interventions for caregivers identified in this review can be used to inform implementation planning for similar interventions designed to reduce the mental health burden experienced by caregivers. For example, results may highlight the importance of providing training to individuals delivering the intervention or involvement of management staff in implementation activities. Improving our understanding of factors associated with implementation will allow implementers to both account for and avoid common implementation challenges, thereby potentially increasing subsequent uptake and effectiveness of e-mental health programs developed to support caregivers.

ETHICS AND DISSEMINATION

Ethical approval is not required for this review, as no raw primary level data will be collected. The results of this work will be disseminated in the form of a scientific publication in a peer-reviewed journal and as presentations at conferences. Plain language summaries will be prepared and provided to groups working with or supporting caregivers and healthcare organizations. Results will also be disseminated throughout the Marie Sklodowska-Curie Innovation Training Network, ENTWINE, which conducts research related to informal care and technological interventions to support caregivers.

AUTHORS' CONTRIBUTIONS

CC contributed to the design of the study and wrote the manuscript. JW, who acts as the reviews guarantor, conceived the study, contributed to the study design and critically revised the manuscript draft. LvE and RS critically revised the study design and manuscript. All authors approved of the final manuscript.

ACKNOWLEDGEMENTS

We would like to thank Agnes Kotka, librarian at Uppsala University, for assisting in development of the search strategy. We are also grateful to Professor Mariët Hagedoorn, Truus van Ittersum (University Medical Centre Groningen, University of Groningen) and Dr. Nathan Davies (University College London), for providing valuable feedback as the peer-reviewers of the search strategy.

FUNDING

This work was supported by the European Union's Horizon 2020 research and innovation program under the Marie-Sklodowska Curie grant agreement No 814072. Funders were not involved in the creation, development or publication of this protocol, nor will they be involved in the conduct, analysis or reporting of the resulting systematic review.

COMPETING INTERESTS

The authors declare they have no conflict of interests.

For peer review only

ABBREVIATIONS

CINAHL: Cumulative Index to Nursing and Allied Health Literature

COPD: Chronic Obstructive Pulmonary Disease

EMBASE: Excerpta Medica dataBASE

PICOS: Population, Interventions, Comparators, Outcomes and Study design

PRECIS-2: PRagmatic Explanatory Continuum Indicator Summary 2

PRISMA-P: Preferred Reporting Items for Systematic Reviews and Meta-Analysis Protocols

PROSPERO: International prospective register of systematic reviews

REFERENCES

1 Hoffmann F, Rodrigues R. *Informal Carers: Who Takes Care of Them? Policy Brief*. Vienna: : European Centre for Social Welfare Policy and Research 2010. https://www.euro.centre.org/publications/detail/387 (accessed 8 Jul 2019).

2 Committee on Family Caregiving for Older Adults, Board on Health Care Services, Health and Medicine Division, *et al.*, editors. Family Caregiving Roles and Impacts. In: *Families Caring for an Aging America*. Washington, DC: : The National Academies Press 2016. doi:10.17226/23606

3 Girgis A, Lambert S. Cost of informal caregiving in cancer care. *Cancer Forum* 2017;**41**:16–22.https://search.informit.com.au/documentSummary;dn=112312308779892;res=IELHEA (accessed 9 Jul 2019).

4 Spasova S, Baeten R, Coster S, *et al. Challenges in Long-term Care in Europe: A Study of National Policies*. Brussels: European Comission: : European Social Policy Network (ESPN) 2018.

5 Colombo F, Llena-Nozal A, Mercier J, *et al. Help Wanted? Providing and Paying for Long-Term Care*. OECD Publishing 2011. doi:10.1787/9789264097759-en

6 Schulz R, Czaja SJ. Family Caregiving: A Vision for the Future. *Am J Geriatr Psychiatry* 2018;**26**:358–63. doi:10.1016/J.JAGP.2017.06.023

7 Lambert SD, Levesque J V., Girgis A. The Impact of Cancer and Chronic Conditions on Caregivers and Family Members. In: Koczwara B, ed. *Cancer and Chronic Conditions*. Singapore: : Springer 2016. 159–202. doi:10.1007/978-981-10-1844-2_6

8 Li Q, Loke AY. The positive aspects of caregiving for cancer patients: a critical review of the literature and directions for future research. *Psychooncology* 2013;**22**:2399–407. doi:10.1002/pon.3311

9 Coumoundouros C, Ould Brahim L, Lambert SD, *et al.* The direct and indirect financial

For peer review only

costs of informal cancer care: A scoping review. *Health Soc Care Community* 2019;**27**:e622–36. doi:10.1111/hsc.12808

10 Pinquart M, Sörensen S. Differences between caregivers and noncaregivers in psychological health and physical health: a meta-analysis. *Psychol Aging* 2003;**18**:250–67.https://psycnet-apa-org.proxy3.library.mcgill.ca/fulltext/2003-05349-011.pdf (accessed 9 Jul 2019).

11 Loh AZ, Tan JS, Zhang MW, *et al.* The Global Prevalence of Anxiety and Depressive Symptoms Among Caregivers of Stroke Survivors. *J Am Med Dir Assoc* 2017;**18**:111–6. doi:10.1016/j.jamda.2016.08.014

12 Geng H, Chuang D, Yang F, *et al.* Prevalence and determinants of depression in caregivers of cancer patients: A systematic review and meta-analysis. *Medicine (Baltimore)* 2018;**97**:e11863. doi:10.1097/MD.0000000000011863

13 Birnbaum HG, Kessler RC, Kelley D, *et al.* Employer burden of mild, moderate, and severe major depressive disorder: mental health services utilization and costs, and work performance. *Depress Anxiety* 2010;**27**:78–89. doi:10.1002/da.20580

14 Kessler RC. The costs of depression. *Psychiatr Clin North Am* 2012;**35**:1–14. doi:10.1016/j.psc.2011.11.005

15 Doran CM, Kinchin I. A review of the economic impact of mental illness. *Aust Heal Rev* 2019;**43**:43–8. doi:10.1071/AH16115

16 Hagedoorn M, Sanderman R, Bolks H, *et al.* Distress in couples coping with cancer: a meta-analysis and critical review of role and gender effects. *Psychol Bull* 2008;**134**:1–30.https://psycnet-apa-org.proxy3.library.mcgill.ca/fulltext/2007-19419-001.pdf (accessed 11 Jul 2019).

17 Hodges LJ, Humphris GM, Macfarlane G. A meta-analytic investigation of the relationship between the psychological distress of cancer patients and their carers. *Soc Sci Med* 2005;**60**:1–12. doi:10.1016/J.SOCSCIMED.2004.04.018

18 Smith GR, Williamson GM, Miller LS, *et al.* Depression and quality of informal care: a longitudinal investigation of caregiving stressors. *Psychol Aging* 2011;**26**:584–91. doi:10.1037/a0022263

19 Christie J, Smith GR, Williamson GM, *et al.* Quality of informal care is multidimensional. *Rehabil Psychol* 2009;**54**:173–81. doi:10.1037/a0015705

20 Cooney C, Howard R, Lawlor B. Abuse of vulnerable people with dementia by their carers: can we identify those most at risk? *Int J Geriatr Psychiatry* 2006;**21**:564–71. doi:10.1002/gps.1525

21 Beach SR, Schulz R, Williamson GM, *et al.* Risk Factors for Potentially Harmful Informal Caregiver Behavior. *J Am Geriatr Soc* 2005;**53**:255–61. doi:10.1111/j.1532-5415.2005.53111.x

22 Mosher CE, Given BA, Ostroff JS. Barriers to mental health service use among distressed family caregivers of lung cancer patients. *Eur J Cancer Care (Engl)* 2015;**24**:50–9.

For peer review only

doi:10.1111/ecc.12203

23 Ploeg J, Ali MU, Markle-Reid M, *et al.* Caregiver-Focused, Web-Based Interventions: Systematic Review and Meta-Analysis (Part 2). *J Med Internet Res* 2018;**20**:e11247. doi:10.2196/11247

24 Christie HL, Martin JL, Connor J, *et al.* eHealth interventions to support caregivers of people with dementia may be proven effective, but are they implementation-ready? *Internet Interv* 2019;:100260. doi:10.1016/j.invent.2019.100260

25 Sin J, Henderson C, Spain D, *et al.* eHealth interventions for family carers of people with long term illness: A promising approach? *Clin Psychol Rev* 2018;**60**:109–25. doi:10.1016/J.CPR.2018.01.008

26 Pagliari C, Sloan D, Gregor P, *et al.* What is eHealth (4): a scoping exercise to map the field. *J Med Internet Res* 2005;**7**:e9. doi:10.2196/jmir.7.1.e9

27 Eysenbach G. What is e-health? *J Med Internet Res* 2001;**3**:e20. doi:10.2196/jmir.3.2.e20

28 Aldehaim AY, Alotaibi FF, Uphold CR, *et al.* The Impact of Technology-Based Interventions on Informal Caregivers of Stroke Survivors: A Systematic Review. *Telemed J e-health* 2016;**22**:223–31. doi:10.1089/tmj.2015.0062

29 Tang WP, Chan CW, So WK, *et al.* Web-based interventions for caregivers of cancer patients: A review of literatures. *Asia-Pacific J Oncol Nurs* 2014;**1**:9–15. doi:10.4103/2347-5625.135811

30 Kaltenbaugh D, Klem M, Hu L, *et al.* Using Web-Based Interventions to Support Caregivers of Patients With Cancer: A Systematic Review. *Oncol Nurs Forum* 2015;**42**:156–64. doi:10.1188/15.ONF.156-164

31 McKechnie V, Barker C, Stott J. Effectiveness of computer-mediated interventions for informal carers of people with dementia—a systematic review. *Int Psychogeriatrics* 2014;**26**:1619–37. doi:10.1017/S1041610214001045

32 Wasilewski MB, Stinson JN, Cameron JI. Web-based health interventions for family caregivers of elderly individuals: A scoping review. *Int J Med Inform* 2017;**103**:109–38. doi:10.1016/J.IJMEDINF.2017.04.009

33 Christie HL, Bartels SL, Boots LMM, *et al.* A systematic review on the implementation of eHealth interventions for informal caregivers of people with dementia. *Internet Interv* 2018;**13**:51–9. doi:10.1016/J.INVENT.2018.07.002

34 Guay C, Auger C, Demers L, *et al.* Components and Outcomes of Internet-Based Interventions for Caregivers of Older Adults: Systematic Review. *J Med Internet Res* 2017;**19**:e313. doi:10.2196/jmir.7896

35 Heynsbergh N, Heckel L, Botti M, *et al.* Feasibility, useability and acceptability of technology-based interventions for informal cancer carers: a systematic review. *BMC Cancer* 2018;**18**. doi:10.1186/S12885-018-4160-9

36 Hopwood J, Walker N, McDonagh L, *et al.* Internet-Based Interventions Aimed at Supporting Family Caregivers of People With Dementia: Systematic Review. *J Med*

Internet Res 2018;**20**:e216. doi:10.2196/jmir.9548

37 Sherifali D, Ali MU, Ploeg J, *et al.* Impact of Internet-Based Interventions on Caregiver Mental Health: Systematic Review and Meta-Analysis. *J Med Internet Res* 2018;**20**:e10668. doi:10.2196/10668

38 Ploeg J, Markle-Reid M, Valaitis R, *et al.* Web-Based Interventions to Improve Mental Health, General Caregiving Outcomes, and General Health for Informal Caregivers of Adults With Chronic Conditions Living in the Community: Rapid Evidence Review. *J Med Internet Res* 2017;**19**:e263. doi:10.2196/jmir.7564

39 Zhao Y, Feng H, Hu M, *et al.* Web-Based Interventions to Improve Mental Health in Home Caregivers of People With Dementia: Meta-Analysis. *J Med Internet Res* 2019;**21**:e13415. doi:10.2196/13415

40 Boots LMM, de Vugt ME, van Knippenberg RJM, *et al.* A systematic review of Internet-based supportive interventions for caregivers of patients with dementia. *Int J Geriatr Psychiatry* 2014;**29**:331–44. doi:10.1002/gps.4016

41 Riper H, Andersson G, Christensen H, *et al.* Theme issue on e-mental health: a growing field in internet research. *J Med Internet Res* 2010;**12**:e74. doi:10.2196/jmir.1713

42 Lal S. E-mental health: Promising advancements in policy, research, and practice. *Healthc Manag Forum* 2019;**32**:56–62. doi:10.1177/0840470418818583

43 Rebello TJ, Marques A, Gureje O, *et al.* Innovative strategies for closing the mental health treatment gap globally. *Curr Opin Psychiatry* 2014;**27**:308–14. doi:10.1097/YCO.0000000000000068

44 Naslund JA, Aschbrenner KA, Araya R, *et al.* Digital technology for treating and preventing mental disorders in low-income and middle-income countries: a narrative review of the literature. *The Lancet Psychiatry* 2017;**4**:486–500. doi:10.1016/S2215-0366(17)30096-2

45 Health Quality Ontario. Internet-Delivered Cognitive Behavioural Therapy for Major Depression and Anxiety Disorders: A Health Technology Assessment. *Ont Health Technol Assess Ser* 2019;**19**:1–199.http://www.ncbi.nlm.nih.gov/pubmed/30873251 (accessed 11 Jul 2019).

46 Sin J, Norman I. Psychoeducational interventions for family members of people with schizophrenia: a mixed-method systematic review. *J Clin Psychiatry* 2013;**74**:e1145–62. doi:10.4088/JCP.12r08308

47 Folker AP, Mathiasen K, Lauridsen SM, *et al.* Implementing internet-delivered cognitive behavior therapy for common mental health disorders: A comparative case study of implementation challenges perceived by therapists and managers in five European internet services. *Internet Interv* 2018;**11**:60–70. doi:10.1016/J.INVENT.2018.02.001

48 Gitlin LN, Marx K, Stanley IH, *et al.* Translating evidence-based dementia caregiving interventions into practice: State-of-the-science and next steps. *Gerontologist* 2015;**55**:210–26. doi:10.1093/geront/gnu123

For peer review only

49 Damschroder LJ, Aron DC, Keith RE, *et al.* Fostering implementation of health services research findings into practice: a consolidated framework for advancing implementation science. *Implement Sci* 2009;**4**:50. doi:10.1186/1748-5908-4-50

50 Loudon K, Treweek S, Sullivan F, *et al.* The PRECIS-2 tool: designing trials that are fit for purpose. *BMJ* 2015;**350**:h2147. doi:10.1136/bmj.h2147

51 Rothwell PM. External validity of randomised controlled trials: "To whom do the results of this trial apply?" *Lancet* 2005;**365**:82–93. doi:10.1016/S0140-6736(04)17670-8

52 Aves T, Allan KS, Lawson D, *et al.* The role of pragmatism in explaining heterogeneity in meta-analyses of randomised trials: a protocol for a cross-sectional methodological review. *BMJ Open* 2017;**7**:e017887. doi:10.1136/BMJOPEN-2017-017887

53 Shamseer L, Moher D, Clarke M, *et al.* Preferred reporting items for systematic review and meta-analysis protocols (PRISMA-P) 2015: elaboration and explanation. *BMJ* 2015;**350**:g7647. doi:10.1136/bmj.g7647

54 Lizarondo L, Stern C, Carrier J, *et al.* Chapter 8: Mixed methods systematic reviews. In: Aromataris E, Munn Z, eds. *Joanna Briggs Institute Reviewer's Manual*. The Joanna Briggs Institute 2017. https://wiki.joannabriggs.org/display/MANUAL/Chapter+8%3A+Mixed+methods+systematic+reviews (accessed 1 Jul 2019).

55 Costantino G, Montano N, Casazza G. When should we change our clinical practice based on the results of a clinical study? Searching for evidence: PICOS and PubMed. *Intern Emerg Med* 2015;**10**:525–7. doi:10.1007/s11739-015-1225-5

56 Centre for Reviews and Dissemination. *Systematic Reviews: CRD's guidance for undertaking reviews in health care*. 3rd ed. CRD, University of York 2009. www.york.ac.uk/inst/crd (accessed 10 Jul 2019).

57 Institute for Health Metrics and Evaluation. *GBD Compare Data Visualization*. Seattle, WA: : IHME, University of Washington 2018. http://www.healthdata.org/data-visualization/gbd-compare (accessed 10 Jul 2019).

58 Sinha M. *Portrait of caregivers, 2012*. 2013. https://www150.statcan.gc.ca/n1/pub/89-652-x/89-652-x2013001-eng.htm (accessed 11 Jul 2019).

59 Thomas J, O'Mara-Eves A, Brunton G. Using qualitative comparative analysis (QCA) in systematic reviews of complex interventions: a worked example. *Syst Rev* 2014;**3**:67. doi:10.1186/2046-4053-3-67

60 Mohr DC, Spring B, Freedland KE, *et al.* The selection and design of control conditions for randomized controlled trials of psychological interventions. *Psychother Psychosom* 2009;**78**:275–84. doi:10.1159/000228248

61 Fitzpatrick R, Davey C, Buxton M, *et al.* Evaluating patient-based outcome measures for use in clinical trials: a review. *Health Technol Assess (Rockv)* 1998;**2**. doi:10.3310/hta2140

62 Radloff LS. The CES-D Scale: A Self-Report Depression Scale for Research in the General Population. *Appl Psychol Meas* 1977;**1**:385–401.

For peer review only

doi:10.1177/014662167700100306

63 Zigmond AS, Snaith RP. The Hospital Anxiety and Depression Scale. *Acta Psychiatr Scand* 1983;**67**:361–70. doi:10.1111/j.1600-0447.1983.tb09716.x

64 Cohen S, Kamarck T, Mermelstein R. A Global Measure of Perceived Stress. *J Health Soc Behav* 1983;**24**:385–96. doi:10.2307/2136404

65 Vis C, Ruwaard J, Finch T, *et al.* Toward an Objective Assessment of Implementation Processes for Innovations in Health Care: Psychometric Evaluation of the Normalization Measure Development (NoMAD) Questionnaire Among Mental Health Care Professionals. *J Med Internet Res* 2019;**21**:e12376. doi:10.2196/12376

66 Proctor E, Silmere H, Raghavan R, *et al.* Outcomes for implementation research: conceptual distinctions, measurement challenges, and research agenda. *Adm Policy Ment Health* 2011;**38**:65–76. doi:10.1007/s10488-010-0319-7

67 Sajobi TT, Li G, Awosoga O, *et al.* A comparison of meta-analytic methods for synthesizing evidence from explanatory and pragmatic trials. *Syst Rev* 2018;**7**:19. doi:10.1186/s13643-017-0668-3

68 McGowan J, Sampson M, Salzwedel DM, *et al.* PRESS Peer Review of Electronic Search Strategies: 2015 Guideline Statement. *J Clin Epidemiol* 2016;**75**:40–6. doi:10.1016/J.JCLINEPI.2016.01.021

69 Lambert SD, Duncan LR, Kapellas S, *et al.* A Descriptive Systematic Review of Physical Activity Interventions for Caregivers: Effects on Caregivers' and Care Recipients' Psychosocial Outcomes, Physical Activity Levels, and Physical Health. *Ann Behav Med* 2016;**50**:907–19. doi:10.1007/s12160-016-9819-3

70 Woodford J, Farrand P, Richards D, *et al.* Psychological treatments for common mental health problems experienced by informal carers of adults with chronic physical health conditions (Protocol). *Syst Rev* 2013;**2**:9. doi:10.1186/2046-4053-2-9

71 Corry M, While A, Neenan K, *et al.* A systematic review of systematic reviews on interventions for caregivers of people with chronic conditions. *J Adv Nurs* 2015;**71**:718–34. doi:10.1111/jan.12523

72 Vis C, Mol M, Kleiboer A, *et al.* Improving Implementation of eMental Health for Mood Disorders in Routine Practice: Systematic Review of Barriers and Facilitating Factors. *JMIR Ment Heal* 2018;**5**:e20. doi:10.2196/mental.9769

73 Wozney L, Mcgrath PJ, Gehring ND, *et al.* eMental Healthcare Technologies for Anxiety and Depression in Childhood and Adolescence: Systematic Review of Studies Reporting Implementation Outcomes. *JMIR Ment Heal* 2018;**5**:e48. doi:10.2196/mental.9655

74 Farrand P, Matthews J, Dickens C, *et al.* Psychological interventions to improve psychological well-being in people with dementia or mild cognitive impairment: systematic review and meta-analysis protocol. *BMJ Open* 2016;**6**:e009713. doi:10.1136/bmjopen-2015-009713

75 Bramer WM, Giustini D, de Jonge GB, *et al.* De-duplication of database search results for

For peer review only

systematic reviews in EndNote. *J Med Libr Assoc* 2016;**104**:240–3. doi:10.3163/1536-5050.104.3.014

76 Ouzzani M, Hammady H, Fedorowicz Z, *et al.* Rayyan—a web and mobile app for systematic reviews. *Syst Rev* 2016;**5**:210. doi:10.1186/s13643-016-0384-4

77 Higgins JP, Savovic J, Page M, *et al.*, editors. *Revised Cochrane risk-of-bias tool for randomized trials (RoB 2)*. 2019. https://drive.google.com/file/d/1xBzD1BeRrXH2DHg58fnTNKinTkX5SLEt/view (accessed 22 Jul 2019).

78 Higgins JP, Altman D, Gotzsche P, *et al.* The Cochrane Collaboration's tool for assessing risk of bias in randomised trials. *BMJ* 2011;**343**:d5928. doi:10.1136/bmj.d5928

79 QSR International Pty Ltd. NVivo qualitative data analysis software. 2014.

80 Hill LG, Cooper BR, Parker LA. Qualitative Comparative Analysis: A Mixed-Method Tool for Complex Implementation Questions. *J Prim Prev* 2019;**40**:69–87. doi:10.1007/s10935-019-00536-5

81 Sullivan GM, Feinn R. Using Effect Size-or Why the P Value Is Not Enough. *J Grad Med Educ* 2012;**4**:279–82. doi:10.4300/JGME-D-12-00156.1

82 Davies EB, Morriss R, Glazebrook C. Computer-delivered and web-based interventions to improve depression, anxiety, and psychological well-being of university students: a systematic review and meta-analysis. *J Med Internet Res* 2014;**16**:e130. doi:10.2196/jmir.3142

83 van Beugen S, Ferwerda M, Hoeve D, *et al.* Internet-based cognitive behavioral therapy for patients with chronic somatic conditions: a meta-analytic review. *J Med Internet Res* 2014;**16**:e88. doi:10.2196/jmir.2777

84 Lorenzo-Luaces L, Johns E, Keefe JR. The Generalizability of Randomized Controlled Trials of Self-Guided Internet-Based Cognitive Behavioral Therapy for Depressive Symptoms: Systematic Review and Meta-Regression Analysis. *J Med Internet Res* 2018;**20**:e10113. doi:10.2196/10113

85 Linde K, Sigterman K, Kriston L, *et al.* Effectiveness of psychological treatments for depressive disorders in primary care: systematic review and meta-analysis. *Ann Fam Med* 2015;**13**:56–68. doi:10.1370/afm.1719

86 Ragin C, Davey S. *Fuzzy-Set/Qualitative Comparative Analysis 3.0*. Irvine, Californai: : Department of Sociology, University of California 2016. http://www.socsci.uci.edu/~cragin/fsQCA/software.shtml (accessed 12 Jul 2019).

87 Ragin CC. User's Guide to Fuzzy-Set/Qualitative Comparative Analysis. Irvine, California: 2017. www.fsqca.com. (accessed 12 Jul 2019).

88 Thomas J, Harden A. Methods for the thematic synthesis of qualitative research in systematic reviews. *BMC Med Res Methodol* 2008;**8**:45. doi:10.1186/1471-2288-8-45

89 Popay J, Roberts H, Sowden A, *et al.* Guidance on the Conduct of Narrative Synthesis in Systematic Reviews. 2006.

For peer review only

http://citeseerx.ist.psu.edu/viewdoc/download?doi=10.1.1.178.3100&rep=rep1&type=pdf (accessed 31 Jul 2019).

90 Lewis CC, Fischer S, Weiner BJ, *et al.* Outcomes for implementation science: an enhanced systematic review of instruments using evidence-based rating criteria. *Implement Sci* 2015;**10**:155. doi:10.1186/s13012-015-0342-x

91 Kerins C, McSharry J, Hayes C, *et al.* Barriers and facilitators to implementation of menu labelling interventions to support healthy food choices: a mixed methods systematic review protocol. *Syst Rev* 2018;**7**:88. doi:10.1186/s13643-018-0752-3

92 Pollock A, Campbell P, Struthers C, *et al.* Stakeholder involvement in systematic reviews: A scoping review. *Syst Rev* 2018;**7**:208. doi:10.1186/s13643-018-0852-0

93 Boote J, Baird W, Sutton A. Public involvement in the systematic review process in health and social care: A narrative review of case examples. *Health Policy (New York)* 2011;**102**:105–16. doi:10.1016/J.HEALTHPOL.2011.05.002

For peer review only

Implementation of e-mental health interventions for informal caregivers of adults with chronic diseases: a protocol for a mixed methods systematic review with a qualitative comparative analysis

PRISMA-P (Preferred Reporting Items for Systematic review and Meta-Analysis Protocols) 2015 checklist: recommended items to address in a systematic review protocol*

Section and topic	Item No	Checklist item	Location in text (page number)
**ADMINISTRATIVE INFORMATION**			
Title:			
Identification	1a	Identify the report as a protocol of a systematic review	1
Update	1b	If the protocol is for an update of a previous systematic review, identify as such	N/A
Registration	2	If registered, provide the name of the registry (such as PROSPERO) and registration number	2 & 5
Authors:			
Contact	3a	Provide name, institutional affiliation, e-mail address of all protocol authors; provide physical mailing address of corresponding author	1
Contributions	3b	Describe contributions of protocol authors and identify the guarantor of the review	11
Amendments	4	If the protocol represents an amendment of a previously completed or published protocol, identify as such and list changes; otherwise, state plan for documenting important protocol amendments	5
Support:			
Sources	5a	Indicate sources of financial or other support for the review	11
Sponsor	5b	Provide name for the review funder and/or sponsor	11
Role of sponsor or funder	5c	Describe roles of funder(s), sponsor(s), and/or institution(s), if any, in developing the protocol	11
**INTRODUCTION**			
Rationale	6	Describe the rationale for the review in the context of what is already known	3-4
Objectives	7	Provide an explicit statement of the question(s) the review will address with reference to participants, interventions, comparators, and outcomes (PICO)	4
**METHODS**			
Eligibility criteria	8	Specify the study characteristics (such as PICO, study design, setting, time frame) and report characteristics (such as years considered, language, publication status) to be used as criteria for eligibility for the review	5-7

For peer review only

Information sources	9	Describe all intended information sources (such as electronic databases, contact with study authors, trial registers or other grey literature sources) with planned dates of coverage	6-7
Search strategy	10	Present draft of search strategy to be used for at least one electronic database, including planned limits, such that it could be repeated	7 & supplementary appendix 3
Study records:			
Data management	11a	Describe the mechanism(s) that will be used to manage records and data throughout the review	7-8
Selection process	11b	State the process that will be used for selecting studies (such as two independent reviewers) through each phase of the review (that is, screening, eligibility and inclusion in meta-analysis)	7-8
Data collection process	11c	Describe planned method of extracting data from reports (such as piloting forms, done independently, in duplicate), any processes for obtaining and confirming data from investigators	8
Data items	12	List and define all variables for which data will be sought (such as PICO items, funding sources), any pre-planned data assumptions and simplifications	8 & supplementary appendix 4
Outcomes and prioritization	13	List and define all outcomes for which data will be sought, including prioritization of main and additional outcomes, with rationale	6
Risk of bias in individual studies	14	Describe anticipated methods for assessing risk of bias of individual studies, including whether this will be done at the outcome or study level, or both; state how this information will be used in data synthesis	8
Data synthesis	15a	Describe criteria under which study data will be quantitatively synthesised	5-6 & 8
	15b	If data are appropriate for quantitative synthesis, describe planned summary measures, methods of handling data and methods of combining data from studies, including any planned exploration of consistency (such as I^2, Kendall's τ)	9
	15c	Describe any proposed additional analyses (such as sensitivity or subgroup analyses, meta-regression)	9
	15d	If quantitative synthesis is not appropriate, describe the type of summary planned	10
Meta-bias(es)	16	Specify any planned assessment of meta-bias(es) (such as publication bias across studies, selective reporting within studies)	8
Confidence in cumulative evidence	17	Describe how the strength of the body of evidence will be assessed (such as GRADE)	N/A

N/A not applicable

*** It is strongly recommended that this checklist be read in conjunction with the PRISMA-P Explanation and Elaboration (cite when available) for important clarification on the items. Amendments to a review protocol should be tracked and dated. The copyright for PRISMA-P (including checklist) is held by the PRISMA-P Group and is distributed under a Creative Commons Attribution Licence 4.0.**

From: Shamseer L, Moher D, Clarke M, Ghersi D, Liberati A, Petticrew M, Shekelle P, Stewart L, PRISMA-P Group. Preferred reporting items for systematic review and meta-analysis protocols (PRISMA-P) 2015: elaboration and explanation. BMJ. 2015 Jan 2;349(jan02 1):g7647.

For peer review only

For peer review only

PRESS Guideline — Search Submission & Peer Review Assessment

SEARCH SUBMISSION: THIS SECTION TO BE FILLED IN BY THE SEARCHER

Searcher: Chelsea Coumoundouros	Email: chelsea.coumoundouros@kbh.uu.se
Date Submitted: 2019/09/18	Date requested by: 2019/10/09

Systematic Review Title:

Implementation of e-Mental Health interventions for informal caregivers of adults with chronic diseases: a protocol for a mixed methods systematic review with a qualitative comparative analysis

This search strategy is …

X	My PRIMARY (core) database strategy — First time submitting a strategy for search question and database
	My PRIMARY (core) strategy — Follow-up review NOT the first time submitting a strategy for search question and database. If this is a response to peer review, itemize the changes made to the review suggestions
	SECONDARY search strategy— First time submitting a strategy for search question and database
	SECONDARY search strategy — NOT the first time submitting a strategy for search question and database. If this is a response to peer review, itemize the changes made to the review suggestions

Database

(i.e., MEDLINE,CINAHL…): *[mandatory]*

MEDLINE

Interface

(i.e., Ovid, EBSCO…): *[mandatory]*

PubMed

Research Question

(Describe the purpose of the search) *[mandatory]*

The aim of this review is to determine factors related to the successful implementation of e-mental health interventions for caregivers of adults with chronic diseases. Two approaches will be used to investigate this. First, studies with more pragmatic designs will be used exclusively to determine which combinations of intervention or implementation characteristics are associated with effective interventions using a qualitative comparative analysis. Second, reports regarding the implementation of e-mental health interventions will be thematically synthesized to establish the common barriers and facilitators to e-mental health implementation.

For peer review only - http://bmjopen.bmj.com/site/about/guidelines.xhtml

PICO Format

(Outline the PICOs for your question — i.e., Patient, Intervention, Comparison, Outcome, and Study Design — as applicable)

**P**	Unpaid adult caregivers of adults with either heart disease, stroke, cancer, diabetes, dementia or chronic obstructive pulmonary disease.
**I**	Any e-mental health intervention targeting the treatment of common mental health difficulties (e.g. depression, anxiety) in caregivers
**C**	**For the qualitative comparative analysis:** Studies must use a non-active control (no treatment, wait-list control, treatment as usual, non-specific treatment component control or education on the care recipient's condition). **For the thematic synthesis:** No restriction based on presence/absence of control group or control type.
**O**	**For the qualitative comparative analysis:** Depression, anxiety, stress or distress measured using an instrument with at least acceptable reliability (Cronbach's alpha ≥ 0.7). **For the thematic synthesis:** Barriers and/or facilitators to implementation.
**S**	**For the qualitative comparative analysis:** Studies must be randomized controlled effectiveness studies. **For the thematic synthesis:** No restriction based on study design.

Inclusion Criteria *[optional]*

P: Caregivers and care recipients must be aged 18 years or older. Chronic health conditions eligible for inclusion are listed in the PICOS above.

I: Interventions must be primarily delivered using Internet technology and target the caregiver's mental health. Interventions may be supplemented by additional forms of support (e.g. telephone call, face-to-face session). Any type of therapy will be included.

O: For the thematic synthesis: Barriers and facilitators can include factors related to any aspect of the Consolidated Framework for Implementation Research or the implementation outcome framework developed by Proctor and colleagues.

S: For the qualitative comparative analysis: Effectiveness will be defined as any study with a mean score of 3 or more using the PRECIS-2 tool.

For peer review only - http://bmjopen.bmj.com/site/about/guidelines.xhtml

Exclusion Criteria *[optional]*

P: Studies that focus on caregivers with severe mental health conditions, caregivers providing care to non-community dwelling care recipients or care recipients at the palliative phase of disease will be excluded.

I: Interventions delivered using the telephone, CD-ROM or video (including Skype) alone will be excluded.

C: For the qualitative comparative analysis: Studies using psychoeducation, education on mental health or active controls will be excluded.

Date limit: Studies published prior to 2007 will be excluded. Technologies from work published prior to 2007 may be outdated and other reviews have shown that production of publications involving eHealth began to rise from 2007 onwards

Language restriction: Studies written in languages other than English or Swedish will be excluded.

Was a search filter applied?

Yes ■ No ☐

If YES, which one(s) (e.g., Cochrane RCT filter, PubMed Clinical Queries filter)? Provide the source if this is a published filter. *[mandatory if YES to previous question — textbox]*

Date limit was applied as part of the search.

Other notes or comments you feel would be useful for the peer reviewer? ***[optional]***

There are no terms related to implementation as (1) this is not the only area we are interested in (also need effectiveness studies) and (2) implementation can be referred to in many different ways and is sometimes not mentioned at all in the title or abstract of studies, so trying to create terms about this topic may lead to poor retrieval of relevant work

Please copy and paste your search strategy here, exactly as run, including the number of hits per line. ***[mandatory]***

For peer review only - http://bmjopen.bmj.com/site/about/guidelines.xhtml

**Database: PubMed. Final number of results from the full search 3265<br>Date Restriction: January 1, 2007 to present**				
**1: Caregivers**	**2: Chronic Health Conditions**	**3: eHealth/ Technology**	**4: Mental Health**	**5: Therapy**
**Title/Abstract**	**Title/Abstract**	**Title/Abstract**	**Title/Abstract**	**Title/Abstract**
Caregiver 19465	Cancer 1019721	eHealth 2990	Mental health 92203	Mental health service 3222
Caregivers 35586	Tumor 615354	e-health 2140	Mood 45028	Mental health services 10159
Care-giver 301	Tumors 279910	e-mental health 213	Mood disorder 3595	Mental healthcare 1741
Care-givers 1034	Tumours 49415	emental health 30	Depression 185606	Therapy 923016
Carer 3436	Tumour 95450	mhealth 3596	Depressive 72042	Therapies 201143
Carers 8055	Neoplasm 34474	m-health 462	Depressed 30180	Treatment 2180941
Informal carer 94	Neoplasms 51500	Smartphone 7429	Affective disorder 2287	Treatments 283397
Informal carers 575	Oncology 65177	Cell phone 1702	Affect 366439	Interventions 307575
Informal caregiver 460	Carcinoma 275810	Cellular phone 362	Negative affect 7192	Intervention 397118
Informal caregivers 1917	Malignan* 271316	Mobile phone 5430	Dysthymia 886	Program 229608
Family 412900	Melanoma 56126	Mobile app 1046	Dysphoria 1871	Programs 158202
Families 124835	Metastasis 147467	Mobile apps 991	Melancholic 594	Programmes 36810
Spouse 4404	Lymphoma 76891	Mobile application 1252	Anxiety 117199	Programme 52551
Spousal 2026	Leukemia 86466	Mobile applications 1283	Burden 141973	Psychological 117677
Spouses 4578	Diabetes 309446	App 16442	Distress 63488	Psychologist 2715
Sibling 10277	Diabetes mellitus 108204	Apps 4615	Stress 465139	Psychoeducation 2202
Siblings 15168	Type 1 diabetes 27354	Application 442621	Well being 49008	Psycho-education 518
Husband 2380	Type 2 diabetes 95548	Applications 318200	Emotion 29552	CBT 8475
Husbands 2371	COPD 32037	iPad 1114	Emotional 95270	Cognitive behavioural therapy 3103
Wife 1919	Chronic obstructive pulmonary	Computer 94973		Cognitive therapy 1960
Wives 1829	disease 32548	Tablet 14085	**MeSH**	Health education 13665
Partner 47483	COAD 147	Computer based 6611	Mood disorders 55994	Problem solving 9444
Partners 51514	Chronic obstructive airway disease	Computer assistive	Depression 108610	Problem solving therapy 335
Parents 92138	80	technology 421	Anxiety 42892	PST 1976
Parent 69723	Chronic obstructive lung disease	Technology 205306	Anxiety disorders 28551	ACT 140661
Friend 6741	1842	Technologies 99160	Stress, psychological 66704	Commitment therapy 809
Friends 16333	Chronic airflow obstruction 102	Electronic communication	Emotions 119893	Behaviour 108763
Relatives 28499	Stroke 150135	757	Mental health 21517	Behavioural 45351
Relative 436237	Cerebrovascular accident 2137	Email 4949		Therapist 9305
Couple 18462	CVA 1540	E-mail 4654		Support 563829
Couples 20787	Cerebral stroke 676	Text messaging 1952		Mindful 2490
Mother 51594	Acute stroke 9906	Text message 1338		Mindfulness 6250
Mothers 64116	Acute cerebrovascular accident 58	Internet 36172		Dialectic behaviour therapy 5
Fathers 9825	Cerebrovascular stroke 133	Internet based 6623		Psychotherapy 13408
Father 10914	Brain vascular accident 2	Wireless 10928		Relaxation 53119
Support person 216	Cerebrovascular apoplexy 2	Digital 71968		Meditation 3440

For peer review only

For peer review only - http://bmjopen.bmj.com/site/about/guidelines.xhtml

Support persons 226<br>Next of kin 770<br>Significant other 583<br>Significant others 1502<br><br>**MeSH**<br>Caregivers 23052<br>Family 134295<br>Spouses 5686<br>Siblings 7235<br>Parents 57525<br>Friends 3754<br>Mothers 23221<br>Fathers 4249	Apoplexy 938<br>Heart disease 71238<br>Cardiovascular disease 89039<br>CVD 27460<br>Ischemic heart disease 10129<br>IHD 2703<br>Coronary artery disease 45519<br>CAD 25678<br>Angina 16189<br>Myocardial infarction 85190<br>MI 28920<br>Heart attack 2262<br>Cardiac event 2336<br>Cardiac disease 7437<br>Dementia 64525<br>Alzheimer* 90032<br>Alzheimer's disease 73741<br>Vascular dementia 3528<br>Frontotemporal dementia 4920<br>FTD 2656<br>Lewy bodies 4361<br>Lewy body 2324<br>Neurocognitive disorder 739<br>Neurocognitive disorders 1551<br>Mild cognitive impairment 13740<br>Memory impair* 8743<br>Cognitive decline 16542<br>Chronic illness 7067<br>Chronic illnesses 2903<br>Chronic diseases 23041<br>Chronic disease 23460<br>Chronic condition 3482<br>Chronic conditions 10263<br>Multi-morbidity 476<br>Multimorbidity 3160<br>Multimorbidities 165<br>Multi-morbidities 66<br>Long-term health condition 39	Online 91273<br>On-line 10976<br>Virtual 42066<br>ICT 3885<br>Communication technology 1639<br>Web 82786<br>Web based 23890<br>Website 13756<br>Web page 528<br>Multimedia 2881<br>e-learning 2196<br>Online social network 154<br>e-therapy 146<br>e-therapies 23<br>etherapies 1<br>etherapy 10<br>iCBT 589<br>cCBT 158<br><br>**MeSH**<br>Cell phone 8834<br>Mobile application 4432<br>Computers 14176<br>Therapy, computer assisted 44315<br>Wireless technology 3136<br>Information technology 237<br>Technology 210207<br>Electronic mail 1850<br>Internet 51939<br>Online social networking 57<br>Virtual reality 1079<br>Web browser 962<br>Multimedia 951		Behavior 363942<br>Behavioral 167313<br>Cognitive behavioral therapy 7242<br>Dialectic behavior therapy 10<br>Behavioural activation 328<br>Behavioral activation 862<br>Counseling 38031<br>Counselling 14505<br>Cognitive reframing 59<br>Cognitive restructuring 506<br>Self-help 3173<br>Self-management 14367<br><br>**MeSH**<br>Mental health services 37274<br>Health education 103830<br>Problem solving 9350<br>Psychotherapy 69239<br>Relaxation 8308<br>Self-management 1348<br>Self-help groups 3265

For peer review only

For peer review only - http://bmjopen.bmj.com/site/about/guidelines.xhtml

	**MeSH**<br>Neoplasms 1299462<br>Diabetes mellitus 192881<br>Pulmonary disease, chronic obstructive 31584<br>Stroke 83194<br>Cardiovascular diseases 805860<br>Dementia 81697<br>Cognition disorders 56940<br>Chronic disease 85925<br>Multimorbidity 503			
**Total for concept 1: 1 278 504**	**Total for concept 2: 3 399 238**	**Total for concept 3: 1 504 756**	**Total for concept 4: 1 400 395**	**Total for concept 5: 4 478 921**

Note: All MeSH terms are exploded

Total for complete search (includes date restriction): 3265

Date of search: September 4, 2019

For peer review only - http://bmjopen.bmj.com/site/about/guidelines.xhtml

Raw PubMed Search

(((((caregiver[Title/Abstract] OR caregivers[Title/Abstract] OR care-giver[Title/Abstract] OR care-givers[Title/Abstract] OR carer[Title/Abstract] OR carers[Title/Abstract] OR informal carer[Title/Abstract] OR informal carers[Title/Abstract] OR informal caregiver[Title/Abstract] OR informal caregivers[Title/Abstract] OR family[Title/Abstract] OR families[Title/Abstract] OR spouse[Title/Abstract] OR spousal[Title/Abstract] OR spouses[Title/Abstract] OR sibling[Title/Abstract] OR siblings[Title/Abstract] OR husband[Title/Abstract] OR husbands[Title/Abstract] OR wife[Title/Abstract] OR wives[Title/Abstract] OR partner[Title/Abstract] OR partners[Title/Abstract] OR parents[Title/Abstract] OR parent[Title/Abstract] OR friend[Title/Abstract] OR friends[Title/Abstract] OR relatives[Title/Abstract] OR relative[Title/Abstract] OR couple[Title/Abstract] OR couples[Title/Abstract] OR mother[Title/Abstract] OR mothers[Title/Abstract] OR fathers[Title/Abstract] OR father[Title/Abstract] OR support person[Title/Abstract] OR support persons[Title/Abstract] OR next of kin[Title/Abstract] OR significant other[Title/Abstract] OR significant others[Title/Abstract] OR caregivers[MeSH terms] OR family[MeSH terms] OR spouses[MeSH terms] OR siblings[MeSH terms] OR parents[MeSH terms] OR friends[MeSH terms] OR mothers[MeSH terms] OR fathers[MeSH terms]) AND (cancer[Title/Abstract] OR tumor[Title/Abstract] OR tumors[Title/Abstract] OR tumours[Title/Abstract] OR tumour[Title/Abstract] OR neoplasm[Title/Abstract] OR neoplasms[Title/Abstract] OR oncology[Title/Abstract] OR carcinoma[Title/Abstract] OR malignan*[Title/Abstract] OR melanoma[Title/Abstract] OR metastasis[Title/Abstract] OR lymphoma[Title/Abstract] OR leukemia[Title/Abstract] OR diabetes[Title/Abstract] OR diabetes mellitus[Title/Abstract] OR Type 1 diabetes[Title/Abstract] OR Type 2 diabetes[Title/Abstract] OR COPD[Title/Abstract] OR chronic obstructive pulmonary disease[Title/Abstract] OR COAD[Title/Abstract] OR chronic obstructive airway disease[Title/Abstract] OR chronic obstructive lung disease[Title/Abstract] OR chronic airflow obstruction[Title/Abstract] OR stroke[Title/Abstract] OR cerebrovascular accident[Title/Abstract] OR CVA[Title/Abstract] OR cerebral stroke[Title/Abstract] OR acute stroke[Title/Abstract] OR acute cerebrovascular accident[Title/Abstract] OR cerebrovascular stroke[Title/Abstract] OR brain vascular accident[Title/Abstract] OR cerebrovascular apoplexy[Title/Abstract] OR apoplexy[Title/Abstract] OR heart disease[Title/Abstract] OR cardiovascular disease[Title/Abstract] OR CVD[Title/Abstract] OR ischemic heart disease[Title/Abstract] OR IHD[Title/Abstract] OR coronary artery disease[Title/Abstract] OR CAD[Title/Abstract] OR angina[Title/Abstract] OR myocardial infarction[Title/Abstract] OR MI[Title/Abstract] OR heart attack[Title/Abstract] OR cardiac event[Title/Abstract] OR cardiac disease[Title/Abstract] OR dementia[Title/Abstract] OR alzheimer*[Title/Abstract] OR alzheimer's disease[Title/Abstract] OR vascular dementia[Title/Abstract] OR frontotemporal dementia[Title/Abstract] OR FTD[Title/Abstract] OR Lewy body[Title/Abstract] OR Lewy bodies[Title/Abstract] OR neurocognitive disorder[Title/Abstract] OR neurocognitive disorders[Title/Abstract] OR mild cognitive impairment[Title/Abstract] OR memory impair*[Title/Abstract] OR cognitive decline[Title/Abstract] OR chronic illness[Title/Abstract] OR chronic illnesses[Title/Abstract] OR chronic diseases[Title/Abstract] OR chronic disease[Title/Abstract] OR chronic condition[Title/Abstract] OR chronic conditions[Title/Abstract] OR multi-morbidity[Title/Abstract] OR multimorbidity[Title/Abstract] OR multimorbidities[Title/Abstract] OR multi-morbidities[Title/Abstract] OR long-term health condition[Title/Abstract] OR neoplasms[MeSH

For peer review only

terms] OR diabetes mellitus[MeSH terms] OR pulmonary disease, chronic obstructive[MeSH terms] OR stroke[MeSH terms] OR cardiovascular diseases[MeSH terms] OR dementia[MeSH terms] OR Cognition disorders[MeSH terms] OR chronic disease[MeSH terms] OR multimorbidity[MeSH terms]) AND (eHealth[Title/Abstract] OR e-health[Title/Abstract] OR e-mental health[Title/Abstract] OR emental health[Title/Abstract] OR mhealth[Title/Abstract] OR m-health[Title/Abstract] OR smartphone[Title/Abstract] OR cell phone[Title/Abstract] OR cellular phone[Title/Abstract] OR mobile phone[Title/Abstract] OR mobile app[Title/Abstract] OR mobile apps[Title/Abstract] OR mobile applications[Title/Abstract] OR mobile application[Title/Abstract] OR app[Title/Abstract] OR application[Title/Abstract] OR applications[Title/Abstract] OR apps[Title/Abstract] OR iPad[Title/Abstract] OR computer[Title/Abstract] OR tablet[Title/Abstract] OR computer based[Title/Abstract] OR computer assistive technology[Title/Abstract] OR technology[Title/Abstract] OR technologies[Title/Abstract] OR electronic communication[Title/Abstract] OR email[Title/Abstract] OR e-mail[Title/Abstract] OR text messaging[Title/Abstract] OR text message[Title/Abstract] OR internet[Title/Abstract] OR internet based[Title/Abstract] OR wireless[Title/Abstract] OR online[Title/Abstract] OR digital[Title/Abstract] OR on-line[Title/Abstract] OR virtual[Title/Abstract] OR ICT[Title/Abstract] OR communication technology[Title/Abstract] OR web[Title/Abstract] OR web based[Title/Abstract] OR website[Title/Abstract] OR web page[Title/Abstract] OR multimedia[Title/Abstract] OR e-learning[Title/Abstract] OR online social network[Title/Abstract] OR iCBT[Title/Abstract] OR cCBT[Title/Abstract] OR e-therapy[Title/Abstract] OR etherapy[Title/Abstract] OR etherapies[Title/Abstract] OR e-therapies[Title/Abstract] OR cell phone[MeSH terms] OR mobile application[MeSH terms] OR computers[MeSH terms] OR therapy, computer assisted[MeSH terms] OR wireless technology[MeSH terms] OR information technology[MeSH terms] OR technology[MeSH terms] OR electronic mail[MeSH terms] OR internet[MeSH terms] OR online social networking[MeSH terms] OR virtual reality[MeSH terms] OR web browser[MeSH terms] OR multimedia[MeSH terms]) AND (mental health[Title/Abstract] OR mood[Title/Abstract] OR mood disorder[Title/Abstract] OR depression[Title/Abstract] OR depressive[Title/Abstract] OR depressed[Title/Abstract] OR affective disorder[Title/Abstract] OR affect[Title/Abstract] OR negative affect[Title/Abstract] OR dysthymia[Title/Abstract] OR dysphoria[Title/Abstract] OR melancholic[Title/Abstract] OR anxiety[Title/Abstract] OR burden[Title/Abstract] OR distress[Title/Abstract] OR stress[Title/Abstract] OR well being[Title/Abstract] OR emotion[Title/Abstract] OR emotional[Title/Abstract] OR mood disorders[MeSH terms] OR depression[MeSH terms] OR anxiety[MeSH terms] OR anxiety disorders[MeSH terms] OR stress, psychological[MeSH terms] OR emotions[MeSH terms] OR mental health[MeSH terms]) AND (mental health service[Title/Abstract] OR mental health services[Title/Abstract] OR mental healthcare[Title/Abstract] OR therapy[Title/Abstract] OR therapies[Title/Abstract] OR treatment[Title/Abstract] OR treatments[Title/Abstract] OR interventions[Title/Abstract] OR intervention[Title/Abstract] OR program[Title/Abstract] OR programs[Title/Abstract] OR programmes[Title/Abstract] OR programme[Title/Abstract] OR psychological[Title/Abstract] OR psychologist[Title/Abstract] OR psychoeducation[Title/Abstract] OR psycho-education[Title/Abstract] OR CBT[Title/Abstract] OR cognitive behavioural therapy[Title/Abstract] OR cognitive therapy[Title/Abstract] OR health education[Title/Abstract] OR problem solving[Title/Abstract] OR problem solving therapy[Title/Abstract] OR PST[Title/Abstract] OR ACT[Title/Abstract] OR commitment therapy[Title/Abstract] OR behaviour[Title/Abstract] OR

For peer review only - http://bmjopen.bmj.com/site/about/guidelines.xhtml

behavioural[Title/Abstract] OR therapist[Title/Abstract] OR support[Title/Abstract] OR mindful[Title/Abstract] OR mindfulness[Title/Abstract] OR dialectic behaviour therapy[Title/Abstract] OR psychotherapy[Title/Abstract] OR relaxation[Title/Abstract] OR meditation[Title/Abstract] OR behavior[Title/Abstract] OR behavioral[Title/Abstract] OR cognitive behavioral therapy[Title/Abstract] OR behavioural activation[Title/Abstract] OR behavioral activation[Title/Abstract] OR dialectic behavior therapy[Title/Abstract] OR counseling[Title/Abstract] OR counselling[Title/Abstract] OR cognitive reframing[Title/Abstract] OR cognitive restructuring[Title/Abstract] OR self-help[Title/Abstract] OR self-management[Title/Abstract] OR mental health services[MeSH terms] OR health education[MeSH terms] OR problem solving[MeSH terms] OR psychotherapy[MeSH terms] OR relaxation[MeSH terms] OR self-management[MeSH terms] OR self-help groups[MeSH terms]))))) AND ("2007/01/01"[Date - Publication] : "3000"[Date - Publication])

For peer review only

PEER REVIEW ASSESSMENT #1: THIS SECTION TO BE FILLED IN BY THE REVIEWER

Reviewer: Professor Mariët Hagedoorn & Truus van Ittersum	Email: mariet.hagedoorn@umcg.nl	Date completed: 2019/10/03

1. TRANSLATION

A ---No revisions	☐
B --- Revision(s) suggested	☐
C --- Revision(s) required	X

If "B" or "C," please provide an explanation or example:

Does the search strategy match the research question/PICO?
I'm not familiar with the PRECIS-2 tool. Do you use it to rate the effectiveness of an internet technology intervention in reducing distress in caregivers as compared to a non-active control group?
Considering your overall question, I would expect that you would assess the effectiveness/successfulness of the implementation?
Are the search concepts clear?
Are there too many or too few PICO elements included?
You could consider not applying the fifth concept, but I can see the point why you do.
Are the search concepts too broad or too narrow?
Does the search retrieve too many or too few records?
Are unconventional or complex strategies explained?
The main focus is on successful implementation, which I find a relevant and timely issue to address in a review. However, the two approaches are not clear to me. For the first approach (which combination of intervention and implementation characteristics are associated with effective interventions), you want to include RCTs. To my understanding RCTs test the effectiveness of an intervention and usually does not include the aim of implementation. Will these studies be relevant to answer your overall question "which factors are related to successful implementation"? It would be helpful to clarify this.
On a practical note, the combination of two approaches appears to make the coding rather complex. Wouldn't it be better to do this in phases? First RCTs, than the remaining literature for approach 2?

I would advise to clarify the two approaches. As I commented above, it is not clear to me how approach one answers your overall research question. I also advise you to reconsider whether you really need two approaches, with specific inclusion criteria. In case you do need both approaches, would it be possible to use a two-step coding process and first search/code RCTs and than apply the second strategy?

Author response:
Thank you for your feedback. We believe that approach one (the qualitative comparative analysis) can produce results that relate to the overall research question regarding implementation of e-mental health interventions for caregivers. Approach one will only utilize pragmatic randomized controlled trials in the analysis. Pragmatic trials use conditions (e.g. the setting, follow up etc.) that more closely reflect how the interventions would be used in real-world settings. Therefore, pragmatic trials are more likely than explanatory trials to include details concerning implementation, which can be included in the qualitative comparative analysis to assess whether certain implementation factors are important for interventions effectiveness. Further, pragmatic trials examine effectiveness in near to real-world settings, and as such, provide estimates of intervention effectiveness that may better reflect estimates of effectiveness that may be found if the intervention was implemented in real-world practices. The PRECIS-2 tool evaluates how pragmatic the design of a randomized controlled trial is. By using this tool in the screening process, only trials with more pragmatic features will be included in the qualitative comparative analysis. The results of the qualitative comparative analysis can also be contrasted to the barriers and

For peer review only - http://bmjopen.bmj.com/site/about/guidelines.xhtml

facilitators identified in the thematic synthesis to determine if, for example, a factor identified as a facilitator to implementation in the thematic synthesis was also identified as important for intervention effectiveness in the qualitative comparative analysis.

We agree, the two approaches adds complexity to the literature screening process, however, if we were to only search for RCTs as a first step, we may miss many reports on implementation of interventions relevant to our research question.

2. BOOLEAN AND PROXIMITY OPERATORS

A ---No revisions	X
B --- Revision(s) suggested	☐
C --- Revision(s) required	☐

For peer review only

If “B” or “C,” please provide an explanation or example:

Are Boolean or proximity operators used correctly?
Is the use of nesting with brackets appropriate and effective for the search?
If NOT is used, is this likely to result in any unintended exclusions?
Could precision be improved by using proximity operators (e.g. adjacent, near, within) or phrase searching instead of AND?
Is the width of proximity operators suitable (e.g. might adj5 pick up more variants than adj2)?

3. SUBJECT HEADINGS

A ---No revisions	X
B --- Revision(s) suggested	☐
C --- Revision(s) required	☐

If “B” or “C,” please provide an explanation or example:

Are the subject headings relevant?
Are any relevant subject headings missing (e.g. previous index terms)?
Are any subject headings too broad or too narrow?
Are subject headings exploded where necessary and vice versa?
Are major headings (“starring” or restrict to focus) used? If so, if there adequate justification?
Are subheadings missing?
Are subheadings attached to subject headings? (Floating subheadings may be preferred)
Are floating subheadings relevant and used appropriately?
Are both subject headings and terms in free text used for each concept?

4. TEXT WORD SEARCHING

A ---No revisions	☐
B --- Revision(s)suggested	X
C --- Revision(s) required	☐

If “B” or “C,” please provide an explanation or example:

General: there is an overload of terms. Some specific terms are not necessary as publications are already found by more general terms (<diabetes> also finds <diabetes mellitus>, <affect> also covers <negative affect>.

For peer review only - http://bmjopen.bmj.com/site/about/guidelines.xhtml

Does the search include all spelling variants in free text (e.g. UK versus US spelling)?
Does the search include all synonyms or antonyms (e.g. opposites)?
Does the search capture relevant truncation (e.g. is truncation at the correct place)?
Using the <*> helps narrowing down the number of terms, without loosing results. <Husband*> will also find <husbands>, <caregiver*> will also find <caregivers>. There are a lot of these terms in the list.
Are acronyms or abbreviations used appropriately? Do they capture irrelevant material? Are the full terms also included?
Are the keywords specific enough or too broad? Are too many or too few keywords used? Are stop words used?
Term “Technology”: Yields many results. Does not seem very relevant. Maybe skip this term?
Term “Web”: Yields mostly “Web of Science” OR “Web of Knowledge” hits. Maybe exclude? Reconsider?
Term “Multimorbidity”: Why multimorbidity? Populations should have a chronic illness and they are specified.
Term “long-term health condition”: To vague.
Have the appropriate fields been searched (e.g. is the choice of text world fields (.tw.) or all fields (.af.) appropriate? Are there any other fields to be included or excluded (database-specific)?
Should any long strings be broken into several shorter search statements?

Author response:
Thank you for these comments. Your feedback has been incorporated into a revised search strategy which has eliminated unnecessary terms and used more term truncations.

Although the terms “technology” and “web” are very broad, we feel they should remain in the primary search strategy as they may yield relevant results pertaining to technological or web based interventions. The terms “multimorbidity” and “long-term health condition” are included as we have come across publications which refer to the population under investigation using more generic terms, such as having multimorbidities or chronic diseases, with the specific health conditions only specified in the main text. Therefore, to ensure the search captures all publications that include caregivers of individuals with the health conditions of interest to this review, we included these more general terms.

5. SPELLING, SYNTAX, AND LINE NUMBERS

A ---No revisions	X
B --- Revision(s)suggested	☐
C --- Revision(s) required	☐

If “B” or “C,” please provide an explanation or example:

Are there any spelling errors
Are there errors in system syntax (e.g. the use of a truncation symbol from a different search interface)?
Are there incorrect line combinations or orphan lines (e.g. lines that are not referred to in the final summation that could indicate an error in an AND or OR statement)?

6. LIMITS AND FILTERS

A ---No revisions	X
B --- Revision(s) suggested	☐
C --- Revision(s) required	☐

If “B” or “C,” please provide an explanation or example:

12

For peer review only

Are all limits and filters used appropriately and are they relevant given the research question?
Are all limits and filters used appropriately and are they relevant for the database?
Are any potentially helpful limits or filters missing? Are the limits or filters too broad or too narrow? Can any limits or filters be added or removed?
Are sources cited for the filters used?

OVERALL EVALUATION (Note: If one or more "revision required" is noted above, the response below must be "revisions required".)

A ---No revisions	☐
B --- Revision(s) suggested	☐
C --- Revision(s) required	χ

For peer review only

Additional comments: see above

For peer review only - http://bmjopen.bmj.com/site/about/guidelines.xhtml

PEER REVIEW ASSESSMENT #2: THIS SECTION TO BE FILLED IN BY THE REVIEWER

Reviewer: Dr. Nathan Davies	Email: n.m.davies@ucl.ac.uk	Date completed: 2019/10/07

1. TRANSLATION

A ---No revisions	X
B --- Revision(s) suggested	☐
C --- Revision(s) required	☐

If "B" or "C," please provide an explanation or example:

Does the search strategy match the research question/PICO?
Yes
Are the search concepts clear?
Yes
Are there too many or too few PICO elements included?
This is a very broad search and large area to study, it is doable but you may consider splitting the review into two.
Are the search concepts too broad or too narrow?
No, they are broad but needed for the question
Does the search retrieve too many or too few records?
My only concern is that this is going to be a very large review and managing such a large review
Are unconventional or complex strategies explained?
N/a

2. BOOLEAN AND PROXIMITY OPERATORS

A ---No revisions	X
B --- Revision(s) suggested	☐
C --- Revision(s) required	☐

If "B" or "C," please provide an explanation or example:

Are Boolean or proximity operators used correctly?
Yes
Is the use of nesting with brackets appropriate and effective for the search?
Yes
If NOT is used, is this likely to result in any unintended exclusions?
Could precision be improved by using proximity operators (e.g. adjacent, near, within) or phrase searching instead of AND?
No
Is the width of proximity operators suitable (e.g. might adj5 pick up more variants than adj2)?
n/a

For peer review only

For peer review only - http://bmjopen.bmj.com/site/about/guidelines.xhtml

3. SUBJECT HEADINGS

A ---No revisions	x
B --- Revision(s) suggested	☐
C --- Revision(s) required	☐

If "B" or "C," please provide an explanation or example:

Are the subject headings relevant?
Yes
Are any relevant subject headings missing (e.g. previous index terms)?
None obvious that I could see
Are any subject headings too broad or too narrow?
No
Are subject headings exploded where necessary and vice versa?
Yes
Are major headings ("starring" or restrict to focus) used? If so, if there adequate justification?
No
Are subheadings missing?
No
Are subheadings attached to subject headings? (Floating subheadings may be preferred)
n/a
Are floating subheadings relevant and used appropriately?
n/a
Are both subject headings and terms in free text used for each concept?
Yes

4. TEXT WORD SEARCHING

A ---No revisions	☐
B --- Revision(s)suggested	x
C --- Revision(s) required	☐

If "B" or "C," please provide an explanation or example:

Does the search include all spelling variants in free text (e.g. UK versus US spelling)?
Add MCI for mild cognitive impairment
Look at searching the term digital health.
Look at cognitive stimulation therapy which can be used with people with dementia
Does the search include all synonyms or antonyms (e.g. opposites)?
See above
Does the search capture relevant truncation (e.g. is truncation at the correct place)?
You can add * to some words which may want to be searched with and without an s at the end for example husband and husbands could just be searched as husband*. But this would not effect the numbers you have already retrieved but may make the search easier to follow with less terms.
Are acronyms or abbreviations used appropriately? Do they capture irrelevant material? Are the full terms also included?
See above
Are the keywords specific enough or too broad? Are too many or too few keywords used? Are stop words used?
See above

For peer review only - http://bmjopen.bmj.com/site/about/guidelines.xhtml

Have the appropriate fields been searched (e.g. is the choice of text world fields (.tw.) or all fields (.af.) appropriate? Are there any other fields to be included or excluded (database-specific)?
Yes
Should any long strings be broken into several shorter search statements?
No

Author response:
Thank you for these suggestions. MCI has been added to the search strategy and more terms have been truncated using the *. Digital health and cognitive stimulation therapy are very relevant concepts, however, the were not added to the search strategy as they should be captured by the terms “digital” and “therapy”, respectively, in the existing search strategy.

5. SPELLING, SYNTAX, AND LINE NUMBERS

A ---No revisions	X
B --- Revision(s)suggested	☐
C --- Revision(s) required	☐

If “B” or “C,” please provide an explanation or example:

Are there any spelling errors
no
Are there errors in system syntax (e.g. the use of a truncation symbol from a different search interface)?
no
Are there incorrect line combinations or orphan lines (e.g. lines that are not referred to in the final summation that could indicate an error in an AND or OR statement)?
no

6. LIMITS AND FILTERS

A ---No revisions	X
B --- Revision(s) suggested	☐
C --- Revision(s) required	☐

If “B” or “C,” please provide an explanation or example:

Are all limits and filters used appropriately and are they relevant given the research question?
The year limit seems appropriate you may even be able to change this to 2009 as last 10 years is a long time in technology so anything older than 10 years is most likely to be irrelevant now.
Are all limits and filters used appropriately and are they relevant for the database?
Yes – although for non-english/sweedish language you could rapidly appraise the English abstract if available and if seems very relevant seek translation of the remainder of the article.
Are any potentially helpful limits or filters missing? Are the limits or filters too broad or too narrow? Can any limits or filters be added or removed?
no
Are sources cited for the filters used?
Don’t understand this question

For peer review only - http://bmjopen.bmj.com/site/about/guidelines.xhtml

OVERALL EVALUATION (Note: If one or more "revision required" is noted above, the response below must be "revisions required".)

A ---No revisions	
B --- Revision(s) suggested	x
C --- Revision(s) required	

Additional comments:

For peer review only

For peer review only - http://bmjopen.bmj.com/site/about/guidelines.xhtml

Implementation of e-mental health interventions for informal caregivers of adults with chronic diseases: a protocol for a mixed methods systematic review with a qualitative comparative analysis

Supplementary File 3: Search Strategy

PubMed

#	
**1**	caregiver*[Title/Abstract] OR<br>care-giver*[Title/Abstract] OR<br>carer*[Title/Abstract] OR<br>family[Title/Abstract] OR<br>families[Title/Abstract] OR<br>spous*[Title/Abstract] OR<br>sibling*[Title/Abstract] OR<br>husband*[Title/Abstract] OR<br>wife[Title/Abstract] OR<br>wives[Title/Abstract] OR<br>partner[Title/Abstract] OR<br>partners[Title/Abstract] OR<br>parents[Title/Abstract] OR<br>parent[Title/Abstract] OR<br>friend[Title/Abstract] OR<br>friends[Title/Abstract] OR<br>relative[Title/Abstract] OR<br>relatives[Title/Abstract] OR<br>couple[Title/Abstract] OR<br>couples[Title/Abstract] OR<br>mother*[Title/Abstract] OR<br>father*[Title/Abstract] OR<br>support person*[Title/Abstract] OR<br>next of kin[Title/Abstract] OR<br>significant other*[Title/Abstract] OR<br>caregivers[MeSH terms] OR<br>family[MeSH terms] OR<br>spouses[MeSH terms] OR<br>siblings[MeSH terms] OR<br>parents[MeSH terms] OR<br>friends[MeSH terms] OR<br>mothers[MeSH terms] OR<br>fathers[MeSH terms]
**2**	cancer[Title/Abstract] OR<br>tumor[Title/Abstract] OR<br>tumors[Title/Abstract] OR<br>tumours[Title/Abstract] OR<br>tumour[Title/Abstract] OR<br>neoplasm*[Title/Abstract] OR<br>oncology[Title/Abstract] OR<br>carcinoma[Title/Abstract] OR<br>malignan*[Title/Abstract] OR

For peer review only

	melanoma[Title/Abstract] OR<br>metastasis[Title/Abstract] OR<br>lymphoma[Title/Abstract] OR<br>leukemia[Title/Abstract] OR<br>diabetes[Title/Abstract] OR<br>COPD[Title/Abstract] OR<br>chronic obstructive pulmonary disease[Title/Abstract] OR<br>COAD[Title/Abstract] OR<br>chronic obstructive airway disease[Title/Abstract] OR<br>chronic obstructive lung disease[Title/Abstract] OR<br>chronic airflow obstruction[Title/Abstract] OR<br>stroke[Title/Abstract] OR<br>cerebrovascular accident[Title/Abstract] OR<br>CVA[Title/Abstract] OR<br>acute cerebrovascular accident[Title/Abstract] OR<br>brain vascular accident[Title/Abstract] OR<br>apoplexy[Title/Abstract] OR<br>heart disease[Title/Abstract] OR<br>cardiovascular disease[Title/Abstract] OR<br>CVD[Title/Abstract] OR<br>ischemic heart disease[Title/Abstract] OR<br>IHD[Title/Abstract] OR<br>coronary artery disease[Title/Abstract] OR<br>CAD[Title/Abstract] OR<br>angina[Title/Abstract] OR<br>myocardial infarction[Title/Abstract] OR<br>MI[Title/Abstract] OR<br>heart attack[Title/Abstract] OR<br>cardiac event[Title/Abstract] OR<br>cardiac disease[Title/Abstract] OR<br>dementia[Title/Abstract] OR<br>alzheimer*[Title/Abstract] OR<br>FTD[Title/Abstract] OR<br>Lewy body[Title/Abstract] OR<br>Lewy bodies[Title/Abstract] OR<br>neurocognitive disorder*[Title/Abstract] OR<br>MCI[Title/Abstract] OR<br>mild cognitive impairment[Title/Abstract] OR<br>memory impair*[Title/Abstract] OR<br>cognitive decline[Title/Abstract] OR<br>chronic illness*[Title/Abstract] OR<br>chronic disease*[Title/Abstract] OR<br>chronic condition*[Title/Abstract] OR<br>multi-morbidit*[Title/Abstract] OR<br>multimorbidit*[Title/Abstract] OR<br>long-term health condition*[Title/Abstract] OR<br>neoplasms[MeSH terms] OR<br>diabetes mellitus[MeSH terms] OR<br>pulmonary disease, chronic obstructive[MeSH terms] OR<br>stroke[MeSH terms] OR<br>cardiovascular diseases[MeSH terms] OR

For peer review only

	dementia[MeSH terms] OR<br>cognition disorders[MeSH terms] OR<br>chronic disease[MeSH terms] OR<br>multimorbidity[MeSH terms]
**3**	eHealth[Title/Abstract] OR<br>e-health[Title/Abstract] OR<br>e-mental health[Title/Abstract] OR<br>emental health[Title/Abstract] OR<br>mhealth[Title/Abstract] OR<br>m-health[Title/Abstract] OR<br>smartphone[Title/Abstract] OR<br>cell phone[Title/Abstract] OR<br>cellular phone[Title/Abstract] OR<br>mobile phone[Title/Abstract] OR<br>app[Title/Abstract] OR<br>apps[Title/Abstract] OR<br>application[Title/Abstract] OR<br>applications[Title/Abstract] OR<br>iPad[Title/Abstract] OR<br>computer[Title/Abstract] OR<br>tablet[Title/Abstract] OR<br>technology[Title/Abstract] OR<br>technologies[Title/Abstract] OR<br>electronic communication[Title/Abstract] OR<br>email[Title/Abstract] OR<br>e-mail[Title/Abstract] OR<br>text messag*[Title/Abstract] OR<br>internet[Title/Abstract] OR<br>wireless[Title/Abstract] OR<br>online[Title/Abstract] OR<br>digital[Title/Abstract] OR<br>on-line[Title/Abstract] OR<br>virtual[Title/Abstract] OR<br>ICT[Title/Abstract] OR<br>web[Title/Abstract] OR<br>website[Title/Abstract] OR<br>multimedia[Title/Abstract] OR<br>e-learning[Title/Abstract] OR<br>online social network[Title/Abstract] OR<br>iCBT[Title/Abstract] OR<br>cCBT[Title/Abstract] OR<br>e-therap*[Title/Abstract] OR<br>etherap*[Title/Abstract] OR<br>cell phone[MeSH terms] OR<br>mobile application[MeSH terms] OR<br>computers[MeSH terms] OR<br>therapy, computer assisted[MeSH terms] OR<br>wireless technology[MeSH terms] OR<br>information technology[MeSH terms] OR<br>technology[MeSH terms] OR<br>electronic mail[MeSH terms] OR

For peer review only

	internet[MeSH terms] OR<br>online social networking[MeSH terms] OR<br>virtual reality[MeSH terms] OR<br>web browser[MeSH terms] OR<br>multimedia[MeSH terms]
**4**	mental health[Title/Abstract] OR<br>mood[Title/Abstract] OR<br>depress*[Title/Abstract] OR<br>affective disorder[Title/Abstract] OR<br>negative affect[Title/Abstract] OR<br>dysthymia[Title/Abstract] OR<br>dysphoria[Title/Abstract] OR<br>melancholic[Title/Abstract] OR<br>anxiety[Title/Abstract] OR<br>burden[Title/Abstract] OR<br>distress[Title/Abstract] OR<br>stress[Title/Abstract] OR<br>well being[Title/Abstract] OR<br>emotion[Title/Abstract] OR<br>emotional[Title/Abstract] OR<br>mood disorders[MeSH terms] OR<br>depression[MeSH terms] OR<br>anxiety[MeSH terms] OR<br>anxiety disorders[MeSH terms] OR<br>stress, psychological[MeSH terms] OR<br>emotions[MeSH terms] OR<br>mental health[MeSH terms]
**5**	mental health service*[Title/Abstract] OR<br>mental healthcare[Title/Abstract] OR<br>therapy[Title/Abstract] OR<br>therapies[Title/Abstract] OR<br>treatment*[Title/Abstract] OR<br>intervention*[Title/Abstract] OR<br>program*[Title/Abstract] OR<br>psychological[Title/Abstract] OR<br>psychologist[Title/Abstract] OR<br>psychoeducation[Title/Abstract] OR<br>psycho-education[Title/Abstract] OR<br>CBT[Title/Abstract] OR<br>health education[Title/Abstract] OR<br>problem solving[Title/Abstract] OR<br>PST[Title/Abstract] OR<br>ACT[Title/Abstract] OR<br>behaviour*[Title/Abstract] OR<br>therapist[Title/Abstract] OR<br>support[Title/Abstract] OR<br>mindful*[Title/Abstract] OR<br>psychotherapy[Title/Abstract] OR<br>relaxation[Title/Abstract] OR<br>meditation[Title/Abstract] OR<br>behavior*[Title/Abstract] OR

For peer review only

	counseling[Title/Abstract] OR<br>counselling[Title/Abstract] OR<br>cognitive reframing[Title/Abstract] OR<br>cognitive restructuring[Title/Abstract] OR<br>self-help[Title/Abstract] OR<br>self-management[Title/Abstract] OR<br>blended[Title/Abstract] OR<br>mental health services[MeSH terms] OR<br>health education[MeSH terms] OR<br>problem solving[MeSH terms] OR<br>psychotherapy[MeSH terms] OR<br>relaxation[MeSH terms] OR<br>self-management[MeSH terms] OR<br>self-help groups[MeSH terms]
**6**	"2007/01/01"[Date - Publication] : "3000"[Date - Publication]
**7**	#1 AND #2 AND #3 AND #4 AND #5 AND #6

CINAHL Plus with Full Text

	((TI (caregiver* OR care-giver* OR carer* OR family OR families OR spous* OR sibling* OR husband* OR wife OR wives OR partner OR partners OR parents OR parent OR friend OR friends OR relative OR relatives OR couple OR couples OR mother* OR father* OR support person* OR next of kin OR significant other*) OR AB (caregiver* OR care-giver* OR carer* OR family OR families OR spous* OR sibling* OR husband* OR wife OR wives OR partner OR partners OR parents OR parent OR friend OR friends OR relative OR relatives OR couple OR couples OR mother* OR father* OR support person* OR next of kin OR significant other*) OR MH (caregivers+ OR family+ OR spouses+ OR siblings+ OR parents+ OR friends+ OR mothers+ OR fathers+)) AND (TI (cancer OR tumor OR tumors OR tumours OR tumour OR neoplasm* OR oncology OR carcinoma OR malignan* OR melanoma OR metastasis OR lymphoma OR leukemia OR diabetes OR COPD OR chronic obstructive pulmonary disease OR COAD OR chronic obstructive airway disease OR chronic obstructive lung disease OR chronic airflow obstruction OR stroke OR cerebrovascular accident OR CVA OR acute cerebrovascular accident OR brain vascular accident OR apoplexy OR heart disease OR cardiovascular disease OR CVD OR ischemic heart disease OR IHD OR coronary artery disease OR CAD OR angina OR myocardial infarction OR MI OR heart attack OR cardiac event OR cardiac disease OR dementia OR alzheimer* OR FTD OR Lewy body OR Lewy bodies OR neurocognitive disorder* OR MCI OR mild cognitive impairment OR memory impair* OR cognitive decline OR chronic illness* OR chronic disease* OR chronic condition* OR multi-morbidit* OR multimorbidit* OR long-term health condition*) OR AB (cancer OR tumor OR tumors OR tumours OR tumour OR neoplasm* OR oncology OR carcinoma OR malignan* OR melanoma OR metastasis OR lymphoma OR leukemia OR diabetes OR COPD OR chronic obstructive pulmonary disease OR COAD OR chronic obstructive airway disease OR chronic obstructive lung disease OR chronic airflow obstruction OR stroke OR cerebrovascular accident OR CVA OR acute cerebrovascular accident OR brain vascular accident OR apoplexy OR heart disease OR cardiovascular disease OR CVD OR ischemic heart disease OR IHD OR coronary artery disease OR CAD OR angina OR myocardial infarction OR MI OR heart attack OR cardiac event OR cardiac disease OR dementia OR alzheimer* OR FTD OR Lewy body OR Lewy bodies OR neurocognitive disorder* OR MCI OR mild cognitive impairment OR memory impair* OR cognitive decline OR chronic illness* OR chronic disease* OR chronic condition* OR multi-morbidit* OR multimorbidit* OR long-term health condition*) OR MH (neoplasms+ OR diabetes mellitus+ OR pulmonary disease, chronic obstructive+ OR stroke+ OR

For peer review only

	cardiovascular diseases+ OR dementia+ OR Cognition disorders+ OR Chronic disease+ OR multimorbidity+)) AND (TI (eHealth OR e-health OR e-mental health OR emental health OR mhealth OR m-health OR smartphone OR cell phone OR cellular phone OR mobile phone OR app OR application OR applications OR apps OR iPad OR computer OR tablet OR technology OR technologies OR electronic communication OR email OR e-mail OR text messag* OR internet OR wireless OR online OR digital OR on-line OR virtual OR ICT OR web OR website OR multimedia OR e-learning OR online social network OR iCBT OR cCBT OR e-therap* OR etherap*) OR AB (eHealth OR e-health OR e-mental health OR emental health OR mhealth OR m-health OR smartphone OR cell phone OR cellular phone OR mobile phone OR app OR application OR applications OR apps OR iPad OR computer OR tablet OR technology OR technologies OR electronic communication OR email OR e-mail OR text messag* OR internet OR wireless OR online OR digital OR on-line OR virtual OR ICT OR web OR website OR multimedia OR e-learning OR online social network OR iCBT OR cCBT OR e-therap* OR etherap*) OR MH (cell phone+ OR mobile application+ OR computers+ OR therapy, computer assisted+ OR wireless technology+ OR information technology+ OR technology+ OR electronic mail+ OR internet+ OR online social networking+ OR virtual reality+ OR web browser+ OR multimedia+)) AND (TI (mental health OR mood OR depress* OR affective disorder OR negative affect OR dysthymia OR dysphoria OR melancholic OR anxiety OR burden OR distress OR stress OR well being OR emotion OR emotional) OR AB (mental health OR mood OR depress* OR affective disorder OR negative affect OR dysthymia OR dysphoria OR melancholic OR anxiety OR burden OR distress OR stress OR well being OR emotion OR emotional) OR MH (mood disorders+ OR depression+ OR anxiety+ OR anxiety disorders+ OR stress, psychological+ OR emotions+ OR mental health+)) AND (TI (mental health service* OR mental healthcare OR therapy OR therapies OR treatment* OR intervention* OR program* OR psychological OR psychologist OR psychoeducation OR psycho-education OR CBT OR health education OR problem solving OR PST OR ACT OR behaviour* OR therapist OR support OR mindful* OR psychotherapy OR relaxation OR meditation OR behavior* OR counseling OR counselling OR cognitive reframing OR cognitive restructuring OR self-help OR self-management OR blended) OR AB (mental health service* OR mental healthcare OR therapy OR therapies OR treatment* OR intervention* OR program* OR psychological OR psychologist OR psychoeducation OR psycho-education OR CBT OR health education OR problem solving OR PST OR ACT OR behaviour* OR therapist OR support OR mindful* OR psychotherapy OR relaxation OR meditation OR behavior* OR counseling OR counselling OR cognitive reframing OR cognitive restructuring OR self-help OR self-management OR blended) OR MH (mental health services+ OR health education+ OR problem solving+ OR psychotherapy+ OR relaxation+ OR self-management+ OR self-help groups+)))

The Cochrane Library

	(((caregiver*):ti,ab,kw OR (care-giver*):ti,ab,kw OR (carer*):ti,ab,kw OR (family):ti,ab,kw OR (families):ti,ab,kw OR (spous*):ti,ab,kw OR (sibling*):ti,ab,kw OR (husband*):ti,ab,kw OR (wife):ti,ab,kw OR (wives):ti,ab,kw OR (partner):ti,ab,kw OR (partners):ti,ab,kw OR (parents):ti,ab,kw OR (parent):ti,ab,kw OR (friend):ti,ab,kw OR (friends):ti,ab,kw OR (relatives):ti,ab,kw OR (relative):ti,ab,kw OR (couples):ti,ab,kw OR (couple):ti,ab,kw OR (mother*):ti,ab,kw OR (father*):ti,ab,kw OR (support NEXT person*):ti,ab,kw OR ("next of kin"):ti,ab,kw OR (significant NEXT other*):ti,ab,kw OR [mh Caregivers] OR [mh Family] OR [mh Spouses] OR [mh Siblings] OR [mh Parents] OR [mh Friends] OR [mh Mothers] OR [mh Fathers]) AND ((cancer):ti,ab,kw OR (tumor):ti,ab,kw OR (tumors):ti,ab,kw OR (tumours):ti,ab,kw OR (tumour):ti,ab,kw OR (neoplasm*):ti,ab,kw OR (oncology):ti,ab,kw OR (carcinoma):ti,ab,kw OR (malignan*):ti,ab,kw OR (melanoma):ti,ab,kw OR (metastasis):ti,ab,kw OR (lymphoma):ti,ab,kw

For peer review only

	OR (leukemia):ti,ab,kw OR (diabetes):ti,ab,kw OR (COPD):ti,ab,kw OR ("chronic obstructive pulmonary disease"):ti,ab,kw OR (COAD):ti,ab,kw OR ("chronic obstructive airway disease"):ti,ab,kw OR ("chronic obstructive lung disease"):ti,ab,kw OR ("chronic airflow obstruction"):ti,ab,kw OR (stroke):ti,ab,kw OR ("cerebrovascular accident"):ti,ab,kw OR (CVA):ti,ab,kw OR ("acute cerebrovascular accident"):ti,ab,kw OR ("brain vascular accident"):ti,ab,kw OR (apoplexy):ti,ab,kw OR ("heart disease"):ti,ab,kw OR ("cardiovascular disease"):ti,ab,kw OR (CVD):ti,ab,kw OR ("ischemic heart disease"):ti,ab,kw OR (IHD):ti,ab,kw OR ("coronary artery disease"):ti,ab,kw OR (CAD):ti,ab,kw OR (angina):ti,ab,kw OR ("myocardial infarction"):ti,ab,kw OR (MI):ti,ab,kw OR ("heart attack"):ti,ab,kw OR ("cardiac event"):ti,ab,kw OR ("cardiac disease"):ti,ab,kw OR (dementia):ti,ab,kw OR (Alzheimer*):ti,ab,kw OR (FTD):ti,ab,kw OR ("Lewy body"):ti,ab,kw OR ("Lewy bodies"):ti,ab,kw OR (neurocognitive NEXT disorder*):ti,ab,kw OR ("mild cognitive impairment"):ti,ab,kw OR (MCI):ti,ab,kw OR (memory NEXT impair*):ti,ab,kw OR ("cognitive decline"):ti,ab,kw OR (chronic NEXT illness*):ti,ab,kw OR (chronic NEXT disease*):ti,ab,kw OR (chronic NEXT condition*):ti,ab,kw OR (multi-morbidit*):ti,ab,kw OR (multimorbidit*):ti,ab,kw OR (long-term NEXT health NEXT condition*):ti,ab,kw OR [mh Neoplasms] OR [mh "Diabetes mellitus"] OR [mh "pulmonary disease, chronic obstructive"] OR [mh stroke] OR [mh "cardiovascular diseases"] OR [mh dementia] OR [mh "cognition disorders"] OR [mh "chronic disease"] OR [mh Multimorbidity]) AND ((eHealth):ti,ab,kw OR (e-health):ti,ab,kw OR ("e-mental health"):ti,ab,kw OR ("emental health"):ti,ab,kw OR (mhealth):ti,ab,kw OR (m-health):ti,ab,kw OR (smartphone):ti,ab,kw OR ("cell phone"):ti,ab,kw OR ("cellular phone"):ti,ab,kw OR ("mobile phone"):ti,ab,kw OR (app):ti,ab,kw OR (application):ti,ab,kw OR (applications):ti,ab,kw OR (apps):ti,ab,kw OR (iPad):ti,ab,kw OR (computer):ti,ab,kw OR (tablet):ti,ab,kw OR (technology):ti,ab,kw OR (technologies):ti,ab,kw OR ("electronic communication"):ti,ab,kw OR (email):ti,ab,kw OR (e-mail):ti,ab,kw OR (text NEXT messag*):ti,ab,kw OR (internet):ti,ab,kw OR (wireless):ti,ab,kw OR (online):ti,ab,kw OR (digital):ti,ab,kw OR (on-line):ti,ab,kw OR (virtual):ti,ab,kw OR (ICT):ti,ab,kw OR (web):ti,ab,kw OR (website):ti,ab,kw OR (multimedia):ti,ab,kw OR (e-learning):ti,ab,kw OR ("online social network"):ti,ab,kw OR (iCBT):ti,ab,kw OR (cCBT):ti,ab,kw OR (e-therap*):ti,ab,kw OR (etherap*):ti,ab,kw OR [mh "cell phone"] OR [mh "mobile application"] OR [mh computers] OR [mh "therapy, computer assisted"] OR [mh "wireless technology"] OR [mh "information technology"] OR [mh technology] OR [mh "electronic mail"] OR [mh internet] OR [mh "online social networking"] OR [mh "virtual reality"] OR [mh "web browser"] OR [mh multimedia]) AND (("mental health"):ti,ab,kw OR (mood):ti,ab,kw OR (depress*):ti,ab,kw OR ("affective disorder"):ti,ab,kw OR ("negative affect"):ti,ab,kw OR (dysthymia):ti,ab,kw OR (dysphoria):ti,ab,kw OR (melancholic):ti,ab,kw OR (anxiety):ti,ab,kw OR (burden):ti,ab,kw OR (distress):ti,ab,kw OR (stress):ti,ab,kw OR ("well being"):ti,ab,kw OR (emotion):ti,ab,kw OR (emotional):ti,ab,kw OR [mh "mood disorders"] OR [mh depression] OR [mh anxiety] OR [mh "anxiety disorders"] OR [mh "stress, psychological"] OR [mh emotions] OR [mh "mental health"]) AND ((mental NEXT health NEXT service*):ti,ab,kw OR ("mental healthcare"):ti,ab,kw OR (therapy):ti,ab,kw OR (therapies):ti,ab,kw OR (treatment*):ti,ab,kw OR (intervention*):ti,ab,kw OR (program*):ti,ab,kw OR (psychological):ti,ab,kw OR (psychologist):ti,ab,kw OR (psychoeducation):ti,ab,kw OR (psycho-education):ti,ab,kw OR (CBT):ti,ab,kw OR ("health education"):ti,ab,kw OR ("problem solving"):ti,ab,kw OR (PST):ti,ab,kw OR (ACT):ti,ab,kw OR (behaviour*):ti,ab,kw OR (therapist):ti,ab,kw OR (support):ti,ab,kw OR (mindful*):ti,ab,kw OR (psychotherapy):ti,ab,kw OR (relaxation):ti,ab,kw OR (meditation):ti,ab,kw OR (behavior*):ti,ab,kw OR (counseling):ti,ab,kw OR (counselling):ti,ab,kw OR ("cognitive reframing"):ti,ab,kw OR ("cognitive restructuring"):ti,ab,kw OR (self-help):ti,ab,kw OR (self-management):ti,ab,kw OR (blended):ti,ab,kw OR [mh "mental health services"] OR [mh "health education"] OR [mh "problem solving"] OR [mh psychotherapy] OR [mh relaxation] OR [mh self-management] OR [mh "self-help groups"]))

For peer review only

EMBASE

Note: The broad search terms used in the search strategy, resulted in the retrieval of a significant number of irrelevant publications in the EMBASE database. Therefore, the search strategy for EMBASE was modified to eliminate key broad terms and when appropriate, replace them with phrases.

#	Searches
1	(caregiver* or care-giver* or carer* or family or families or spous* or sibling* or husband* or wife or wives or partner or partners or parents or parent or friend or friends or relatives or couple or couples or mother* or father* or support person* or next of kin or significant other*).ab,ti.
2	exp caregiver/
3	exp family/
4	exp spouse/
5	exp sibling/
6	exp parent/
7	exp friend/
8	exp mother/
9	exp father/
10	1 or 2 or 3 or 4 or 5 or 6 or 7 or 8 or 9
11	(cancer or tumor or tumors or tumours or tumour or neoplasm* or oncology or carcinoma or malignan* or melanoma or metastasis or lymphoma or leukemia or diabetes or COPD or chronic obstructive pulmonary disease or COAD or chronic obstructive airway disease or chronic obstructive lung disease or chronic airflow obstruction or stroke or cerebrovascular accident or CVA or acute cerebrovascular accident or brain vascular accident or apoplexy or heart disease or cardiovascular disease or CVD or ischemic heart disease or IHD or coronary artery disease or CAD or angina or myocardial infarction or MI or heart attack or cardiac event or cardiac disease or dementia or alzheimer* or FTD or Lewy body or Lewy bodies or neurocognitive disorder* or MCI or mild cognitive impairment or memory impair* or cognitive decline or chronic illness* or chronic disease* or chronic condition* or multi-morbidit* or multimorbidit* or long-term health condition*).ab,ti.
12	exp neoplasm/
13	exp diabetes mellitus/
14	exp chronic obstructive lung disease/
15	exp cerebrovascular accident/
16	exp cardiovascular disease/
17	exp dementia/

18	exp chronic disease/
19	exp multiple chronic conditions/
20	(eHealth or e-health or e-mental health or emental health or mhealth or m-health or smartphone or cell phone or cellular phone or mobile phone or app or apps or iPad or computer or tablet or electronic communication or email or e-mail or text messag* or internet or wireless or online or digital or on-line or virtual or ICT or website or multimedia or e-learning or online social network or iCBT or cCBT or e-therap* or etherap* or web-based or (mobile adj2 technolog*) or (technolog* adj2 solution*) or (technology adj2 based)).ab,ti.
21	exp mobile phone/
22	exp mobile application/
23	exp computer/
24	exp computer assisted therapy/
25	exp wireless communication/
26	exp information technology/
27	exp technology/
28	exp e-mail/
29	exp Internet/
30	exp virtual reality/
31	exp web browser/
32	exp multimedia/
33	20 or 21 or 22 or 23 or 24 or 25 or 26 or 27 or 28 or 29 or 30 or 31 or 32
34	(mental health or mood or depress* or affective disorder or negative affect or dysthymia or dysphoria or melancholic or anxiety or burden or distress or stress or well being or emotion or emotional).ab,ti.
35	exp mood disorder/
36	exp depression/
37	exp anxiety/ or exp anxiety disorder/
38	exp mental stress/
39	exp mental health/
40	(mental health service* or mental healthcare or intervention* or program* or psychological or psychologist or psychoeducation or psycho-education or CBT or health education or problem solving or PST or ACT or behaviour* or therapist or support or mindful* or psychotherapy or relaxation or meditation or behavior* or counseling or counselling or cognitive reframing or cognitive restructuring or self-help or self-management or (group adj therapy) or (psycho* adj therapy) or (cognitive adj2 therapy) or (behavio* adj2 therapy) or (problem adj2 therapy) or (acceptance adj2 therapy) or blended).ab,ti.

41	exp mental health service/
42	exp health education/
43	exp problem solving/
44	exp psychotherapy/
45	exp self help/
46	34 or 35 or 36 or 37 or 38 or 39
47	40 or 41 or 42 or 43 or 44 or 45
48	11 or 12 or 13 or 14 or 15 or 16 or 17 or 18 or 19
49	10 and 33 and 46 and 47 and 48
50	limit 49 to yr="2007 -Current"

PsychINFO

#	Searches
1	(caregiver* or care-giver* or carer* or family or families or spous* or sibling* or husband* or wife or wives or partner or partners or parents or parent or friend or friends or relative or relatives or couple or couples or mother* or father* or support person* or next of kin or significant other*).ab,ti.
2	exp Caregivers/
3	exp Family/
4	exp Spouses/
5	exp Siblings/
6	exp Parents/
7	exp Mothers/
8	exp Fathers/
9	exp Friends/
10	(cancer or tumor or tumors or tumours or tumour or neoplasm* or oncology or carcinoma or malignan* or melanoma or metastasis or lymphoma or leukemia or diabetes or COPD or chronic obstructive pulmonary disease or COAD or chronic obstructive airway disease or chronic obstructive lung disease or chronic airflow obstruction or stroke or cerebrovascular accident or CVA or acute cerebrovascular accident or brain vascular accident or apoplexy or heart disease or cardiovascular disease or CVD or ischemic heart disease or IHD or coronary artery disease or CAD or angina or myocardial infarction or MI or heart attack or cardiac event or cardiac disease or dementia or alzheimer* or FTD or Lewy body or Lewy bodies or neurocognitive disorder* or MCI or mild cognitive impairment or memory impair* or cognitive

	decline or chronic illness* or chronic disease* or chronic condition* or multi-morbidit* or multimorbidit* or long-term health condition*).ab,ti.
11	exp Neoplasms/
12	exp Diabetes Mellitus/
13	exp Chronic Obstructive Pulmonary Disease/
14	exp Stroke/
15	exp Dementia/
16	exp Cognitive Impairment/
17	exp Chronic Illness/
18	exp Comorbidity/
19	exp Cardiovascular Disorders/
20	(eHealth or e-health or e-mental health or emental health or mhealth or m-health or smartphone or cell phone or cellular phone or mobile phone or app or application or applications or apps or iPad or computer or tablet or technology or technologies or electronic communication or email or e-mail or text messag* or internet or wireless or online or digital or on-line or virtual or ICT or web or website or multimedia or e-learning or online social network or iCBT or cCBT or e-therap* or etherap*).ab,ti.
21	exp Mobile Phones/
22	exp Mobile Applications/
23	exp Computers/
24	exp Computer Assisted Therapy/
25	exp Wireless Technologies/
26	exp "Information and Communication Technology"/
27	exp Technology/
28	exp Computer Mediated Communication/
29	exp Internet/
30	exp Online Social Networks/
31	exp Virtual Reality/
32	exp Websites/
33	exp Multimedia/
34	(mental health or mood or depress* or affective disorder or negative affect or dysthymia or dysphoria or melancholic or anxiety or burden or distress or stress or well being or emotion or emotional).ab,ti.
35	exp "Depression (Emotion)"/ or exp Major Depression/

36	exp Anxiety Disorders/ or exp Anxiety/
37	exp Physiological Stress/
38	exp Emotions/
39	exp Mental Health/
40	exp Mood Disorders/
41	(mental health service* or mental healthcare or therapy or therapies or treatment* or intervention* or program* or psychological or psychologist or psychoeducation or psycho-education or CBT or health education or problem solving or PST or ACT or behaviour* or therapist or support or mindful* or psychotherapy or relaxation or meditation or behavior* or counseling or counselling or cognitive reframing or cognitive restructuring or self-help or self-management or blended).ab,ti.
42	exp Mental Health Services/
43	exp Health Education/
44	exp Problem Solving/
45	exp Psychotherapy/
46	exp Relaxation/
47	exp Self-Management/
48	exp Support Groups/
49	1 or 2 or 3 or 4 or 5 or 6 or 7 or 8 or 9
50	10 or 11 or 12 or 13 or 14 or 15 or 16 or 17 or 18 or 19
51	20 or 21 or 22 or 23 or 24 or 25 or 26 or 27 or 28 or 29 or 30 or 31 or 32 or 33
52	34 or 35 or 36 or 37 or 38 or 39 or 40
53	41 or 42 or 43 or 44 or 45 or 46 or 47 or 48
54	49 and 50 and 51 and 52 and 53
55	limit 54 to yr="2007 -Current"

Web of Science

#	
**1**	TI=(caregiver* OR care-giver* OR carer* OR family OR families OR spous* OR sibling* OR husband* OR wife OR wives OR partner OR partners OR parents OR parent OR friend OR friends OR relative OR relatives OR couple OR couples OR mother* OR father* OR support person* OR next of kin OR significant other*)
**2**	AB=(caregiver* OR care-giver* OR carer* OR family OR families OR spous* OR sibling* OR husband* OR wife OR wives OR partner OR partners OR parents OR parent OR friend OR friends OR relative OR relatives OR couple OR couples OR mother* OR father* OR support person* OR next of kin OR significant other*)
**3**	**#1 OR #2**
**4**	TI=(cancer OR tumor OR tumors OR tumours OR tumour OR neoplasm* OR oncology OR carcinoma OR malignan* OR melanoma OR metastasis OR lymphoma OR leukemia OR diabetes OR COPD OR chronic obstructive pulmonary disease OR COAD OR chronic obstructive airway disease OR chronic obstructive lung disease OR chronic airflow obstruction OR stroke OR cerebrovascular accident OR CVA OR acute cerebrovascular accident OR brain vascular accident OR apoplexy OR heart disease OR cardiovascular disease OR CVD OR ischemic heart disease OR IHD OR coronary artery disease OR CAD OR angina OR myocardial infarction OR MI OR heart attack OR cardiac event OR cardiac disease OR dementia OR alzheimer* OR FTD OR Lewy body OR Lewy bodies OR neurocognitive disorder* OR MCI OR mild cognitive impairment OR memory impair* OR cognitive decline OR chronic illness* OR chronic disease* OR chronic condition* OR multi-morbidit* OR multimorbidit* OR long-term health condition*)
**5**	AB=(cancer OR tumor OR tumors OR tumours OR tumour OR neoplasm* OR oncology OR carcinoma OR malignan* OR melanoma OR metastasis OR lymphoma OR leukemia OR diabetes OR COPD OR chronic obstructive pulmonary disease OR COAD OR chronic obstructive airway disease OR chronic obstructive lung disease OR chronic airflow obstruction OR stroke OR cerebrovascular accident OR CVA OR acute cerebrovascular accident OR brain vascular accident OR apoplexy OR heart disease OR cardiovascular disease OR CVD OR ischemic heart disease OR IHD OR coronary artery disease OR CAD OR angina OR myocardial infarction OR MI OR heart attack OR cardiac event OR cardiac disease OR dementia OR alzheimer* OR FTD OR Lewy body OR Lewy bodies OR neurocognitive disorder* OR MCI OR mild cognitive impairment OR memory impair* OR cognitive decline OR chronic illness* OR chronic disease* OR chronic condition* OR multi-morbidit* OR multimorbidit* OR long-term health condition*)
**6**	**#4 OR #5**
**7**	TI=(eHealth OR e-health OR e-mental health OR emental health OR mhealth OR m-health OR smartphone OR cell phone OR cellular phone OR mobile phone OR app OR application OR applications OR apps OR iPad OR computer OR tablet OR technology OR technologies OR electronic communication OR email OR e-mail OR text messag* OR internet OR wireless OR online OR digital OR on-line OR virtual OR ICT OR web OR website OR multimedia OR e-learning OR online social network OR iCBT OR cCBT OR e-therap* OR etherap*)
**8**	AB=(eHealth OR e-health OR e-mental health OR emental health OR mhealth OR m-health OR smartphone OR cell phone OR cellular phone OR mobile phone OR app OR application OR applications OR apps OR iPad OR computer OR tablet OR technology OR technologies OR electronic communication OR email OR e-mail OR text messag* OR internet OR wireless OR online OR digital OR on-line OR virtual OR ICT OR web OR website OR multimedia OR e-learning OR online social network OR iCBT OR cCBT OR e-therap* OR etherap*)
**9**	**#7 OR #8**

For peer review only

**10**	TI=(mental health OR mood OR depress* OR affective disorder OR negative affect OR dysthymia OR dysphoria OR melancholic OR anxiety OR burden OR distress OR stress OR well being OR emotion OR emotional)
**11**	AB=(mental health OR mood OR depress* OR affective disorder OR negative affect OR dysthymia OR dysphoria OR melancholic OR anxiety OR burden OR distress OR stress OR well being OR emotion OR emotional)
**12**	**#10 OR #11**
**13**	TI=(mental health service* OR mental healthcare OR therapy OR therapies OR treatment* OR intervention* OR program* OR psychological OR psychologist OR psychoeducation OR psycho-education OR CBT OR health education OR problem solving OR PST OR ACT OR behaviour* OR therapist OR support OR mindful* OR psychotherapy OR relaxation OR meditation OR behavior* OR counseling OR counselling OR cognitive reframing OR cognitive restructuring OR self-help OR self-management OR blended)
**14**	AB=(mental health service* OR mental healthcare OR therapy OR therapies OR treatment* OR intervention* OR program* OR psychological OR psychologist OR psychoeducation OR psycho-education OR CBT OR health education OR problem solving OR PST OR ACT OR behaviour* OR therapist OR support OR mindful* OR psychotherapy OR relaxation OR meditation OR behavior* OR counseling OR counselling OR cognitive reframing OR cognitive restructuring OR self-help OR self-management OR blended)
**15**	**#13 OR #14**
**16**	**#3 AND #6 AND # 9 AND #12 AND #15**

For peer review only

Implementation of e-mental health interventions for informal caregivers of adults with chronic diseases: a protocol for a mixed methods systematic review with a qualitative comparative analysis

Supplementary File 4: Data Extraction Form

**Study Identification Features**		
Unique Study Identifier		
Title		
First Author		
Year of Publication		
Country of Study		
Funding Source		
Protocol Available?		
**Study Characteristics**		
Aims and Objectives		
Study Design (e.g. RCT, qualitative)		
Inclusion/Exclusion Criteria		
Recruitment Setting and Method		
Randomization	Sequence Generation	
	Type	
	Allocation Concealment	
Blinding	Data Collectors	
	Data Analysts	
**Caregiver Characteristics**		
Caregiver Sample Size (total and per arm)		
Age		
Gender		
Severity of Anxiety at Baseline		
Severity of Depression at Baseline		
Severity of Distress at Baseline		
Severity of Stress at Baseline		
Relationship to Care Recipient		
Amount of Care Provided		
Length of Time as a Caregiver		
Education Level		
Employment Status		
**Care Recipient Characteristics**		
Sample Size (total and per arm)		
Diagnosis		
Age		
Gender		
Severity Measure of Physical Health Condition of Care Recipient		
**Intervention**		

For peer review only

Summary of Intervention	
Summary of Control Condition	
Type of Therapy (e.g. psychoeducation, CBT)	
Format of Intervention (e.g. web, mobile app)	
Individual or Dyadic Intervention	
Other Support Provided (e.g. some face to face contact)	
Provider (e.g. lay worker, psychologist)	
Training provided to intervention provider (yes/no, describe if yes)	
Total Length of Treatment	
Number of Sessions	
Length of Sessions	
Adherence (did participants receive intended intervention, did they complete entire intervention)	
**Implementation Outcomes**	
Implementation Outcomes Measured or Described	
Measurement Tools Used	
Timing of Measurements	
**Mental Health Outcome Measurements**	
Mental Health Outcome Measurements (primary and secondary)	
Quality of Outcome Measurements	
Timing of Measurements	
**Statistical Techniques**	
Power Calculation	
Target Sample Size	
Method of Dealing with Missing Data	
Baseline Comparability	
**Participant Flow**	
Eligible Participants	
Randomised to Intervention	
Randomised to Control	
Lost to Follow-Up Intervention[1]	
Lost to Follow-Up Control[1]	
Analysed Intervention[2]	
Analysed Control[2]	
**Results Summary**	
Summary of Mental Health Outcome Results	
Summary of Implementation Results	
**Quantitative Results[3]**	

For peer review only

Intervention	Outcome Measurement	
	Measurement Scale Used	
	Analysis Method (ITT, per protocol)	
	Pre-Treatment Means	
	Pre-Treatment Standard Deviation	
	Pre-Treatment Number Analysed	
	Post-Treatment Means	
	Post-Treatment Standard Deviation	
	Post-Treatment Number Analysed	
Control	Outcome Measurement	
	Measurement Scale Used	
	Analysis Method (ITT, per protocol)	
	Pre-Treatment Means	
	Pre-Treatment Standard Deviation	
	Pre-Treatment Number Analysed	
	Post-Treatment Means	
	Post-Treatment Standard Deviation	
	Post-Treatment Number Analysed	
**Additional Comments**		

[1]Numbers lost to follow-up to be reported at each time point measured; [2]Numbers analysed to be reported for each time point measured; [3]Results to be extracted for outcomes of anxiety, depression, distress and/or stress at all follow-up time points

For peer review only

Implementation of e-mental health interventions for informal caregivers of adults with chronic diseases: a protocol for a mixed methods systematic review with a qualitative comparative analysis

Supplementary File 5: Sample data table

Study	Conditions[a]				Outcome	
	Lay-led	Mobile app	Supplemented with personal contact	Implementing organization involved in project	Effect size (raw Hedges' g)	Effective intervention[b]
Study A	0	0	1	1	0.25	0
Study B	1	0	1	0	0.5	1

[a]The conditions shown here are examples of conditions that could be explored in the qualitative comparative analysis. These are subject to change as the conditions selected depend on the features and heterogeneity among studies included in the review. The number 1 signifies that the study belongs to the indicated set. For example Study A was not lay led or a mobile app, but it did contain personal contact and the implementing organization was involved in the project.

[b]Assuming an effective intervention is defined as having a Hedges' g effect size ≥ 0.3. In this example Study A has an effect size below this cut-off, therefore, is considered not effective.

For peer review only

BMJ Open

Implementation of e-mental health interventions for informal caregivers of adults with chronic diseases: a protocol for a mixed methods systematic review with a qualitative comparative analysis

Journal:	*BMJ Open*
Manuscript ID	bmjopen-2019-035406.R1
Article Type:	Protocol
Date Submitted by the Author:	31-Mar-2020
Complete List of Authors:	Coumoundouros, Chelsea; Uppsala University, Clinical Psychology in Healthcare, Department of Women's and Children's Health<br>von Essen, Louise; Uppsala University, Clinical Psychology in Healthcare, Department of Women's and Children's Health<br>Sanderman, Robbert; University of Groningen, University Medical Center Groningen, Department of Health Psychology; University of Twente, Department of Psychology, Health and Technology<br>Woodford, Joanne; Uppsala University, Clinical Psychology in Healthcare, Department of Women's and Children's Health
**Primary Subject Heading**:	Mental health
Secondary Subject Heading:	Evidence based practice, Health services research
Keywords:	MENTAL HEALTH, World Wide Web technology < BIOTECHNOLOGY & BIOINFORMATICS, PRIMARY CARE, Depression & mood disorders < PSYCHIATRY, Anxiety disorders < PSYCHIATRY

SCHOLARONE™ Manuscripts

I, the Submitting Author has the right to grant and does grant on behalf of all authors of the Work (as defined in the below author licence), an exclusive licence and/or a non-exclusive licence for contributions from authors who are: i) UK Crown employees; ii) where BMJ has agreed a CC-BY licence shall apply, and/or iii) in accordance with the terms applicable for US Federal Government officers or employees acting as part of their official duties; on a worldwide, perpetual, irrevocable, royalty-free basis to BMJ Publishing Group Ltd ("BMJ") its licensees and where the relevant Journal is co-owned by BMJ to the co-owners of the Journal, to publish the Work in this journal and any other BMJ products and to exploit all rights, as set out in our licence.

The Submitting Author accepts and understands that any supply made under these terms is made by BMJ to the Submitting Author unless you are acting as an employee on behalf of your employer or a postgraduate student of an affiliated institution which is paying any applicable article publishing charge ("APC") for Open Access articles. Where the Submitting Author wishes to make the Work available on an Open Access basis (and intends to pay the relevant APC), the terms of reuse of such Open Access shall be governed by a Creative Commons licence – details of these licences and which Creative Commons licence will apply to this Work are set out in our licence referred to above.

Other than as permitted in any relevant BMJ Author's Self Archiving Policies, I confirm this Work has not been accepted for publication elsewhere, is not being considered for publication elsewhere and does not duplicate material already published. I confirm all authors consent to publication of this Work and authorise the granting of this licence.

For peer review only

Implementation of e-mental health interventions for informal caregivers of adults with chronic diseases: a protocol for a mixed methods systematic review with a qualitative comparative analysis

Chelsea COUMOUNDOUROS[1] (chelsea.coumoundouros@kbh.uu.se), Louise VON ESSEN[1] (louise-von.essen@kbh.uu.se), Robbert SANDERMAN[2, 3] (r.sanderman@umcg.nl), Joanne WOODFORD[1] (joanne.woodford@kbh.uu.se)

Corresponding author

Chelsea Coumoundouros, Clinical Psychology in Healthcare, Department of Women's and Children's Health, Uppsala University, 751 23 Uppsala, Sweden. E-mail: chelsea.coumoundouros@kbh.uu.se Telephone: 0046 729 999 211

Affiliations

[1]Clinical Psychology in Healthcare, Department of Women's and Children's Health, Uppsala University, 751 23 Uppsala, Sweden.

[2]Department of Health Psychology, University Medical Center Groningen, University of Groningen, 9713 AV, Groningen, the Netherlands.

[3]Department of Psychology, Health and Technology, University of Twente, Enschede, the Netherlands

Word count: 4078

For peer review only

ABSTRACT

Introduction. Informal caregivers provide the majority of care to individuals with chronic health conditions, benefiting the care recipient and reducing use of formal care services. However, providing informal care negatively impacts the mental health of many caregivers. E-mental health interventions have emerged as a way to provide accessible mental healthcare to caregivers. Much attention has been given to reviewing the effectiveness and efficacy of such interventions, however, factors related to implementation have received less consideration. Therefore, this mixed methods systematic review will aim to examine factors associated with the effectiveness and implementation of e-mental health interventions for caregivers.

Methods and analysis. Eligible studies published since 2007 will be searched for in several electronic databases (CINAHL Plus with Full Text, the Cochrane Library, EMBASE, PsychINFO, PubMed, and Web of Science), clinical trial registries and OpenGrey, with all screening steps conducted by two independent reviewers. Studies will be included if they focus on the implementation or effectiveness of e-mental health interventions designed for informal adult caregivers of adults with cancer, heart disease, stroke, diabetes, dementia or chronic obstructive pulmonary disease. Pragmatic randomized controlled trials quantitatively reporting on caregiver anxiety, depression, psychological distress or stress will be used for a qualitative comparative analysis to identify combinations of conditions that result in effective interventions. Qualitative and quantitative data on implementation of e-mental health interventions for caregivers will be integrated in a thematic synthesis to identify barriers and facilitators to implementation. These results will inform future development and implementation planning of e-mental health interventions for caregivers.

Ethics and dissemination. Ethical approval is not required for this study as no primary data will be collected. Results will be disseminated in the form of a scientific publication and presentations at academic conferences and plain language summaries for various stakeholders.

For peer review only

STRENGTHS AND LIMITATIONS OF THIS STUDY

- The mixed method design of this review will ensure a wide variety of data on implementation is captured
- The peer-reviewed, comprehensive search strategy with all selection steps completed by two independent reviewers will ensure a thorough search of the literature and reduce bias in study selection
- High heterogeneity across studies in terms of implementation or intervention features is easily accommodated in a qualitative comparative analysis
- Crisp set qualitative comparative analysis will make results of this review more concrete and usable for healthcare professionals and decision-makers
- However, crisp set qualitative comparative analysis dichotomizes all variables including the outcome, therefore, a more detailed understanding of the strength of the effect size may be lost in this process

INTRODUCTION

Informal caregivers (hereafter referred to as caregivers) are family members or friends who provide unpaid support and care to individuals with healthcare needs. Caregivers play a vital societal role in healthcare systems worldwide, providing up to 80% of care to individuals with long-term care needs.[1] Informal care provision can include emotional support, assistance with household tasks (e.g. cooking, cleaning), medical care, transportation, managing finances and advocacy on behalf of the care recipient.[2,3] Demand for caregivers is expected to increase in the future as the proportion of older adults in populations around the world increases and healthcare policies favour deinstitutionalization and outpatient care.[4,5] As societal dependence on informal care continues to grow, it is becoming increasingly important to implement programs and policies to support individuals who become caregivers.[2,6]

Caregivers can experience both positive and negative outcomes over the course of their time as a caregiver.[3,7,8] Caregiving can lead to an improved relationship between caregiver and care recipient, feelings of personal development and a sense of accomplishment related to obtaining skills and recognizing the impact of the care they provide.[8] However, caregivers may also experience negative outcomes related to the caregiving role, such as financial strain and poor physical and mental health.[3,7,9] Indeed, the rate of depression and anxiety among caregivers exceeds that of the general population.[10] The prevalence of depressive symptoms in cancer and stroke caregivers is often above 40% and the prevalence of anxiety ranges from 21% to over 40%.[11,12] Mental health problems can result in large personal and societal costs related to increased morbidity and reduced productivity.[13–15] Additionally, poor caregiver mental health negatively impacts distress levels in the care recipient[16,17] and the quality of care provided by the caregiver.[18–21]

There is a clear need to develop effective interventions and resources to prevent or reduce the mental health burden experienced by caregivers. However, caregivers have reported various barriers to accessing mental health services such as lack of knowledge regarding available services, financial barriers, stigma and prioritization of the caregiving role over self-care.[22] E-

For peer review only

health technologies have emerged as an accessible way to provide support and information to caregivers[23–25] and can be designed to achieve various goals such as, improving communication, teaching skills or reducing depression.[26,27] Numerous systematic reviews and meta-analyses have examined e-health interventions for caregivers of adults with chronic health conditions, showing the potential for e-health interventions to improve caregiver well-being.[23,25,28–40]

E-mental health interventions, that is, mental health interventions delivered via the internet or using mobile technologies,[27,41,42] represent a subset of e-health interventions. E-mental health solutions offer a means to improve mental health service access globally[43,44] by eliminating many barriers to mental health service access (e.g. transportation, stigma, time)[25,42,45] and are often more cost-effective than traditional therapies.[42,45] Meta-analyses show reductions in caregiver's depression and anxiety in response to e-mental health interventions.[37,39] However, as many reviews focus on intervention efficacy and effectiveness,[23,28–32,34,36–40,46] gaps remain in our understanding of factors related to the intervention and the implementation context that make e-mental health interventions effective among caregivers.

Wider literature suggests that the implementation of e-health programs in real-world settings often encounters many barriers,[24,33,47,48] preventing effective interventions from being made available to those who need them. Few reviews have examined factors related to implementation of e-health interventions for caregivers,[33,35] with no current reviews, to the best of our knowledge, focusing on implementation of e-mental health interventions for caregivers exclusively. Evaluating the implementation of an intervention is essential to gain insights into why interventions succeed or fail when put into practice. Factors influencing implementation can be related to the intervention itself, the participants, the implementation setting and wider societal factors (e.g. regional policies).[49] Additionally, although efficacy (also referred to as explanatory) trials are a useful tool to establish the beneficial effects of an intervention under ideal settings, trials with a more pragmatic design may be better suited to investigating factors potentially associated with implementation given real-world conditions are more closely reflected in pragmatic trials.[50,51] Commonly, systematic reviews and meta-analyses do not distinguish between pragmatic and explanatory trials despite the different conditions (e.g. setting, recruitment methods, eligibility criteria, control of adherence to and delivery of the intervention) under which interventions are evaluated.[50,52]

The aim of this review is to examine factors related to the effectiveness and implementation of e-mental health interventions for caregivers of adults with chronic diseases. Two approaches will be used to investigate this. First, studies with more pragmatic designs will be used exclusively to determine which combinations of intervention or implementation characteristics are associated with effectiveness using a qualitative comparative analysis. Second, reports regarding the implementation of e-mental health interventions will be thematically synthesized to establish the common barriers and facilitators to e-mental health implementation. Findings from this review can be used to guide the development of effective e-mental health interventions to support caregivers and ensure the successful implementation of these interventions within real-world healthcare settings.

For peer review only

METHODS AND ANALYSIS

The Preferred Reporting Items for Systematic Reviews and Meta-Analysis Protocols (PRISMA-P) checklist[53] (online supplementary appendix 1) and the Joanna Briggs methodology for mixed methods systematic reviews[54] were used to guide the development of this protocol. Protocol changes will be recorded by study authors and reported in the publication of review results.

Study eligibility criteria

The eligibility criteria used to inform study inclusion and exclusion are outlined using PICOS (population, interventions, comparators, outcomes and study design).[55,56]

Population

Unpaid adult caregivers (aged 18 years or older) of adults with either cancer, chronic obstructive pulmonary disease (COPD), dementia, diabetes, heart disease or stroke. Care recipient's chronic health conditions eligible for inclusion were selected as, globally, they are the largest sources of disability adjusted life years due to physical chronic diseases in adults[57] and often require informal care.[58] No restrictions will be placed on the frequency or amount of care provided for someone to be considered a caregiver. Studies exclusively focusing on caregivers with severe mental health conditions (e.g. psychosis or bipolar disorder) will be excluded, as the focus of this review is on e-mental health interventions targeting psychological health difficulties associated with the provision of informal care, for example anxiety or depression, as opposed to targeting severe mental health conditions. Studies with interventions that solely focus on caregivers providing care to non-community dwelling care recipients will be excluded, given caregivers of individuals who do not live in the community may spend less time providing informal care[59] and generally experience lower levels of depression.[60,61] Additionally, studies of interventions designed specifically for caregivers of individuals at the end-of-life (e.g. within a few months of death) will be excluded, as end-of-life caregiving is associated with additional needs and burdens, for example difficulties related to grief and bereavement.[62]

Interventions

Interventions will utilise internet technology, such as web-based platforms or mobile-based applications, to deliver a mental health intervention to caregivers.[27,41] E-mental health interventions can encompass many types of mental health support such as screening, prevention, treatment or service delivery.[41] This review will focus on interventions targeting the treatment of common caregiver psychological health difficulties (anxiety, depression, psychological distress or stress). This can include any type of mental health treatment, including psychoeducation. Psychoeducation is defined as the provision of information regarding common psychological health difficulties and can be delivered passively (e.g. an information website) or actively (e.g. an information website with therapist support, homework or exercises).[63] The e-mental health intervention may be supplemented with additional forms of support (such as telephone contact, face-to-face support or video-conferencing), however, the majority of the

For peer review only

intervention must be internet based. Interventions delivered via telephone, CD-ROM or video (including Skype) alone will be excluded.

Comparators

As it is necessary to determine effect sizes for the qualitative comparative analysis,[64] only studies of pragmatic randomized controlled trials with non-active controls will be included in this analysis. Non-active controls include: no treatment, wait-list control, treatment as usual, non-specific treatment component control (e.g. control for attention) or education on the care recipient's condition.[65] Studies using psychoeducation or active controls (e.g. controls using specific treatment components or studies comparing two therapies) will be excluded.

For thematic synthesis of barriers and facilitators to implementation, studies of any design (e.g. randomized controlled trials, process evaluations, focus groups) will be included in the analysis, regardless of the presence or absence of a control.

Outcomes

For the qualitative comparative analysis, studies must report on caregiver mental health outcomes, specifically anxiety, depression, psychological distress or stress, measured using an instrument with at least acceptable reliability (Cronbach's alpha ≥ 0.7).[66] Reliability of outcome measures will be assessed based on the main validation paper of the relevant measurement instrument, as this review will likely include studies with different caregiver populations, ages, genders and languages, the combination of which may not have been validated. Examples of eligible measurement instruments include the Center for Epidemiologic Studies – Depression Scale,[67] the Hospital Anxiety and Depression Scale[68] or the Perceived Stress Scale.[69]

For the thematic synthesis, studies will report on barriers and/or facilitators to intervention implementation. This may include qualitative (e.g. interviews or focus groups) or quantitative (e.g. Normalization Measure Development questionnaire[70]) data. Barriers or facilitators can include factors related to any aspect of the Consolidated Framework for Implementation Research[49] or the implementation outcome framework developed by Proctor and colleagues.[71] The Consolidated Framework for Implementation Research consists of 5 domains related to implementation, namely (1) intervention characteristics (e.g. adaptability, complexity); (2) outer setting (e.g. external policies, patient needs and resources); (3) inner/implementation setting (e.g. culture within the organization, readiness for implementation); (4) characteristics of individuals (e.g. self-efficacy, individual stage of change); and (5) process (e.g. planning, engaging).[49] The implementation outcome framework broadly classifies measurable implementation outcomes which includes acceptability, adoption, feasibility, fidelity, reach, appropriateness, implementation cost and sustainability.[71]

Study designs

Studies included for the qualitative comparative analysis must be pragmatic randomized controlled trials (also referred to as effectiveness trials). Pragmatic trials will be identified using the validated PRagmatic Explanatory Continuum Indicator Summary 2 (PRECIS-2) tool.[50]

For peer review only

PRECIS-2 was developed with input from clinicians, researchers and policymakers to allow trialists to assess how pragmatic or explanatory their trial design is across nine domains: eligibility criteria, recruitment, setting, organization, flexibility (delivery), flexibility (adherence), follow-up, primary outcome and primary analysis.[50] Trials with a pragmatic design will be defined as any trial with a mean score of 3 or higher using the PRECIS-2 tool.[50] PRECIS-2 has been used with this cut-off score to categorize studies in another systematic review,[72] although to our knowledge it has not previously been used to exclude studies from a systematic review. Using a cut-off score of 3 should ensure generous inclusion of trials containing at least a mixture of pragmatic and explanatory design features.[50]

To assess barriers and facilitators to implementation, any study type with quantitative and/or qualitative data will be eligible for inclusion.

Search strategy

Comprehensive literature searches will be conducted in multiple electronic databases (CINAHL Plus with Full Text, the Cochrane Library, EMBASE, PsychINFO, PubMed and Web of Science). Clinical trial registries (www.clinicaltrials.gov and www.who.int/trialsearch/) will be searched for relevant completed clinical trials and the resulting publications will be found and screened for inclusion. Searches for grey literature will be performed using OpenGrey (http://www.opengrey.eu/), a database of grey literature in Europe such as research reports and conference papers.

The search strategy was developed in consultation with Agnes Kotka, a librarian at Uppsala University and was reviewed by Professor Mariët Hagedoorn and Truus van Ittersum (University Medical Centre Groningen, University of Groningen) and Dr. Nathan Davies (University College London) following the PRESS peer review guidelines[73] (online supplementary appendix 2). The search was constructed using terms related to (1) caregivers; (2) the chronic health conditions of interest (cancer, COPD, dementia, diabetes, heart disease and stroke); (3) e-health/information and communication technology; (4) mental health; and (5) psychological therapies (see online supplementary appendix 3). Included terms were informed by existing reviews focusing on the population and/or intervention of interest to this review.[9,33,39,74–79] Search terms were refined based on feedback from the peer-review process, resulting in the addition of more truncations to search terms, elimination of repetitive search terms that did not retrieve additional records and the addition of an abbreviation missed prior to the peer-review process. The search will include relevant Medical Subject Headings (MeSH) when possible and terms will be searched for in the title/abstract of publications. Included studies will be restricted to those published in English, Dutch, German or Swedish. Literature produced from January 2007 onwards will be eligible for inclusion. Technologies from work published prior to 2007 may be outdated and other reviews have shown that production of publications involving e-health began to rise from 2007 onwards.[33,35]

Upon final inclusion of any studies, their references, results from forward citation searches and from the first three pages of the “find similar” search function in PubMed will be used to check for any additional studies of interest. Experts in the field will be contacted to identify further studies for inclusion.

For peer review only

Study selection

Results of database searches will be imported into EndNote for deduplication following the procedures outlined by Bramer et al.[80] Remaining records will be imported into the online screening software Rayyan.[81] Titles, abstracts and full-texts will be screened independently by two reviewers. Conflicts will be discussed and a third reviewer will be consulted if consensus cannot be reached. Study selection will be based on the criteria outlined by the PICOS, with reasons for study exclusion being recorded at the full-text screening stage. Full-texts will be checked against each sub-section of the PICOS, recording which sub-sections are or are not met by each study, with an overall reason for exclusion being reported in the PRISMA flow-diagram. This will facilitate detailed discussions regarding study exclusion when conflicts arise. If studies do not contain enough information to decide upon inclusion, the original authors will be contacted at most twice over a one month period to obtain information to determine study eligibility. If the original authors do not respond, the study will be excluded. Abstracts, theses, books, commentaries, editorials and letters to the editor will be excluded. Reviews and study protocols will also be excluded, however, the references of related reviews will be checked for additional studies of interest, published results of relevant study protocols will be obtained and if protocol results are unpublished, authors will be contacted to determine whether access to unpublished results is possible.

Records retrieved from searches of clinical trial registries and OpenGrey will be screened for eligibility by one reviewer. When relevant clinical trial registries are identified, any resulting publications will be retrieved and screened for inclusion, unless already captured by the electronic database searches. If results from relevant trial registries are unpublished, authors will be contacted to determine if they are able to share details of any available results. Authors of grey literature records that do not contain enough information to assess eligibility will also be contacted for additional study details.

Exclusion of studies on the basis of adopting a more explanatory, as opposed to pragmatic, trial design will be conducted as a final step during the full text screening process. This screening step will only be applied to trials eligible for the qualitative comparative analysis. Studies will be scored using the PRECIS-2 tool by two independent reviewers and studies with a mean score below 3 will be excluded.[50,72]

Assessment of methodological quality

Methodological quality of studies included in the qualitative comparative analysis will be evaluated using the Cochrane Risk of Bias 2.0 tool for randomized controlled trials[82,83]. This evaluation will facilitate the identification of selection, performance, measurement, attrition and reporting bias.[83] Authors will be contacted if more information is required to complete the quality assessment. Reporting bias will be explored by comparing outcomes measures described in study protocols to the outcome measures reported in the methods and results sections of the corresponding completed trial. In response to any identified inconsistencies, authors will be contacted to determine potential causes of this. Study assessment will be conducted by two independent reviewers, followed by discussion of any discrepancies, consulting a third reviewer

For peer review only

as needed. Studies will not be excluded based on methodological quality, however the results of the Cochrane Risk of Bias 2.0 evaluation will be reported descriptively.

Data extraction

Data from included full-texts will be extracted into Microsoft Excel (2016), using a data extraction form developed for this review based on the Centre for Reviews and Dissemination guidelines.[56] Extracted information will include data pertaining to study participants, study design, the intervention and relevant outcomes (full details in online supplementary appendix 4). Data used in the qualitative comparative analysis and thematic synthesis will be extracted independently by two reviewers, with resulting extractions compared for accuracy and completion. All other data will be extracted by one reviewer and verified by a second reviewer. If conflicts arise, the original publication will be referred to in order to resolve misunderstandings and a third reviewer will be consulted if necessary. Authors will be contacted at most twice to obtain additional data and/or clarification as needed. Qualitative results pertaining to implementation will be transferred into NVivo 10 software[84] for thematic synthesis.

Data synthesis

Data related to the characteristics of each included study, such as the sample (e.g. sample size, participant demographics) or intervention (e.g. duration, type of support provided, delivery mode) characteristics, will be reported in summary tables. Further data synthesis will involve two analysis methods. Pragmatic randomized controlled trials with quantitative mental health outcome data will be included in the qualitative comparative analysis. Publications of any study design reporting on implementation will be included in the thematic synthesis, taking an integrative approach to synthesize both qualitative and quantitative findings.

Qualitative comparative analysis

A crisp set qualitative comparative analysis will be conducted to determine sets of conditions that result in effective e-mental health interventions for caregivers.[64] Crisp set qualitative comparative analysis involves dichotomizing outcome data (e.g. effective or not effective) and conditions (e.g. present or absent) selected for inclusion in the analysis into distinct categories.[64] A crisp set analysis approach was selected over a fuzzy set analysis as the results will be more clearly interpretable and easier for decision-makers to use.[85]

The first step of a qualitative comparative analysis is to build a data table containing information regarding the effectiveness of each study and conditions related to the intervention and its implementation (see online supplementary appendix 5).[64] Conditions to include in the data table will be based on important factors related to intervention components (e.g. uses goal-setting, homework), intervention delivery methods (e.g. mobile app, computer), and implementation (e.g. acceptability, feasibility). By restricting this analysis to pragmatic trials, which are designed to more closely reflect real-world settings, implementation conditions are more likely to be reported. Conditions selected will be adjusted given the need to ensure adequate heterogeneity is present.[64] Qualitative comparative analysis requires diversity among studies in terms of conditions present and intervention effectiveness in order to determine the combination

For peer review only

of factors sufficient for interventions to be effective.[64] Therefore, adjustments to outcome classification and conditions selected for analysis will be needed after data collection is completed.

Intervention effectiveness will be measured as the standardized mean effect size between control and comparator groups' mental health outcomes, calculated using Hedges' g and the Comprehensive Meta-Analysis (version 3) software. Effect sizes will be calculated for all mental health outcomes of interest for this review (anxiety, depression, psychological distress and stress) and will be based on data collected immediately after intervention completion. If enough studies report subsequent post-intervention follow-ups, these effect sizes will be calculated to explore whether different factors contribute to sustained intervention success. Effect sizes will be used to create crisp sets to categorize studies as effective (Hedges' g ≥ 0.3) or not effective.[86] If most interventions are effective (or not effective), a different classification system will be created to ensure adequate heterogeneity for analysis,[64] for example categorizing studies as highly effective (Hedges' g ≥ 0.5) or not highly effective.[86] Proposed cut-offs were developed based on existing meta-analyses of e-mental health interventions.[87–90]

The main data table will use general effectiveness as the outcome measure, meaning the primary mental health outcome as identified in each study will be used to represent the effectiveness of that intervention. If studies include multiple outcomes of interest, but do not identify a primary outcome measure, the outcome most frequently measured in included studies will be used to evaluate intervention effectiveness. Secondary analyses may be conducted for anxiety, depression, psychological distress and stress separately, to explore whether different conditions are more important for different outcome measures. However, this is dependent upon identification of an adequate number of studies for each outcome of interest. After completion of the data table, truth tables will be constructed and the software fs/QCA (version 3.1b) will be used to determine the sufficient conditions for effective e-mental health interventions.[91,92]

Thematic synthesis

Data from studies addressing implementation of e-mental health interventions for caregivers will be thematically synthesized using a deductive coding approach, to identify barriers and facilitators experienced during implementation.[93,94] It will likely be necessary to integrate qualitative and quantitative data as many aspects of implementation such as acceptability, feasibility and usability, may be measured using quantitative tools.[95] First, qualitative data will be thematically analyzed using the Consolidated Framework for Implementation Research to guide coding.[49] This framework was selected a priori as it was developed by combining multiple implementation theories into a single, comprehensive theory covering all aspects related to implementation[49] and it has been used as a coding guide in other reviews on implementation.[33,96] Qualitative data will be coded based on the 39 pre-defined constructs within the Consolidated Framework for Implementation Research,[49] with the creation of additional codes if needed.

Quantitative data will be narratively summarized to facilitate subsequent integration of qualitative and quantitative findings. Creating narrative summaries will involve approaches such as textually describing study findings and grouping findings based on the constructs and domains of the

For peer review only

Consolidated Framework for Implementation Research.[94] Initially, 10% of full-texts included in the thematic synthesis will be coded independently by two reviewers, followed by discussion of the coding process in consultation with a third reviewer. The remaining coding will be conducted by one reviewer with regular discussions with a second reviewer, involving a third reviewer as needed. Results of the initial coding of qualitative data and narrative summaries of quantitative data will be analyzed together to identify barriers and facilitators to implementation. Two reviewers will independently identify barriers and facilitators, followed by discussion involving a third reviewer as needed.[93] Through this discussion, more abstract, analytical themes will be developed that go beyond the initial codes and identified barriers and facilitators.[93] This process will be iterative, modifying barriers and facilitators after defining initial analytical themes, followed by further refinement of analytical themes until the analytical themes fully encompass all codes and identified barriers and facilitators.[93]

Patient and public involvement

There was no patient or public involvement in the development of this protocol. However, caregivers and healthcare professionals will be involved in the interpretation of data for the thematic synthesis.[97,98] Results of the thematic synthesis will be presented to a multidisciplinary group of stakeholders (e.g. caregivers, mental health service providers) to explore whether the identified themes resonate with their experiences or if they feel important aspects related to implementation have not been captured by the synthesis. Their perspectives will be incorporated into the interpretation of the results of the thematic synthesis.

DISCUSSION

Despite the importance of implementation planning and need for effective e-mental health interventions for caregivers in real world practice, there have been no reviews focusing on this area. Using pragmatic trials and implementation research, this review will identify both the key characteristics of effective interventions and barriers and facilitators to implementation. A qualitative comparative analysis will be employed to identify combinations of conditions resulting in effective e-mental health interventions for caregivers, a method which, to the best of our knowledge, has not yet been used in this field. The results of the qualitative comparative analysis can be used to improve the design of future e-mental health interventions by identifying intervention components and implementation factors important to intervention effectiveness in real-world settings.

Additionally, common barriers and facilitators to implementation of e-mental health interventions for caregivers identified in this review can be used to inform implementation planning for similar interventions designed to reduce the mental health burden experienced by caregivers. For example, results may highlight the importance of providing training to individuals delivering the intervention or involvement of management staff in implementation activities. Improving our understanding of factors associated with implementation will allow implementers to both account for and avoid common implementation challenges, thereby potentially increasing subsequent uptake and effectiveness of e-mental health programs developed to support caregivers.

For peer review only - http://bmjopen.bmj.com/site/about/guidelines.xhtml

ETHICS AND DISSEMINATION

Ethical approval is not required for this review, as no raw primary level data will be collected. The results of this work will be disseminated in the form of a scientific publication in a peer-reviewed journal and as presentations at conferences. Plain language summaries will be prepared and provided to groups working with or supporting caregivers and healthcare organizations. Results will also be disseminated throughout the Marie Sklodowska-Curie Innovation Training Network, ENTWINE, which conducts research related to informal care and technological interventions to support caregivers.

AUTHORS' CONTRIBUTIONS

CC contributed to the design of the study and wrote the manuscript. JW, who acts as the reviews guarantor, conceived the study, contributed to the study design and critically revised the manuscript draft. LvE and RS critically revised the study design and manuscript. All authors approved of the final manuscript.

ACKNOWLEDGEMENTS

We would like to thank Agnes Kotka, librarian at Uppsala University, for assisting in development of the search strategy. We are also grateful to Professor Mariët Hagedoorn, Truus van Ittersum (University Medical Centre Groningen, University of Groningen) and Dr. Nathan Davies (University College London), for providing valuable feedback as the peer-reviewers of the search strategy.

FUNDING

This work was supported by the European Union's Horizon 2020 research and innovation program under the Marie-Sklodowska Curie grant agreement No 814072. Funders were not involved in the creation, development or publication of this protocol, nor will they be involved in the conduct, analysis or reporting of the resulting systematic review.

COMPETING INTERESTS

The authors declare they have no conflict of interests.

ABBREVIATIONS

CINAHL: Cumulative Index to Nursing and Allied Health Literature

COPD: Chronic Obstructive Pulmonary Disease

EMBASE: Excerpta Medica dataBASE

PICOS: Population, Interventions, Comparators, Outcomes and Study design

PRECIS-2: PRagmatic Explanatory Continuum Indicator Summary 2

PRISMA-P: Preferred Reporting Items for Systematic Reviews and Meta-Analysis Protocols

For peer review only

REFERENCES

1 Hoffmann F, Rodrigues R. *Informal carers: who takes care of them? Policy brief*. Vienna: : European Centre for Social Welfare Policy and Research 2010. https://www.euro.centre.org/publications/detail/387 (accessed 8 Jul 2019).

2 National Academies of Sciences Engineering and Medicine. Family caregiving roles and impacts. In: Committee on Family Caregiving for Older Adults, Board on Health Care Services, Health and Medicine Division, *et al.*, eds. *Families Caring for an Aging America*. Washington, DC: : The National Academies Press 2016. doi:10.17226/23606

3 Girgis A, Lambert S. Cost of informal caregiving in cancer care. *Cancer Forum* 2017;**41**:16–22.https://search.informit.com.au/documentSummary;dn=112312308779892;res=IELHEA (accessed 9 Jul 2019).

4 Spasova S, Baeten R, Coster S, *et al. Challenges in long-term care in Europe: A study of national policies*. Brussels: European Comission: : European Social Policy Network (ESPN) 2018.

5 Colombo F, Llena-Nozal A, Mercier J, *et al. Help wanted? Providing and paying for long-term care*. OECD Publishing 2011. doi:10.1787/9789264097759-en

6 Schulz R, Czaja SJ. Family caregiving: A vision for the future. *Am J Geriatr Psychiatry* 2018;**26**:358–63. doi:10.1016/J.JAGP.2017.06.023

7 Lambert SD, Levesque J V., Girgis A. The impact of cancer and chronic conditions on caregivers and family members. In: Koczwara B, ed. *Cancer and Chronic Conditions*. Singapore: : Springer 2016. 159–202. doi:10.1007/978-981-10-1844-2_6

8 Li Q, Loke AY. The positive aspects of caregiving for cancer patients: a critical review of the literature and directions for future research. *Psychooncology* 2013;**22**:2399–407. doi:10.1002/pon.3311

9 Coumoundouros C, Ould Brahim L, Lambert SD, *et al.* The direct and indirect financial costs of informal cancer care: A scoping review. *Health Soc Care Community* 2019;**27**:e622–36. doi:10.1111/hsc.12808

10 Pinquart M, Sörensen S. Differences between caregivers and noncaregivers in psychological health and physical health: a meta-analysis. *Psychol Aging* 2003;**18**:250–67.https://psycnet-apa-org.proxy3.library.mcgill.ca/fulltext/2003-05349-011.pdf (accessed 9 Jul 2019).

11 Loh AZ, Tan JS, Zhang MW, *et al.* The global prevalence of anxiety and depressive symptoms among caregivers of stroke survivors. *J Am Med Dir Assoc* 2017;**18**:111–6. doi:10.1016/j.jamda.2016.08.014

12 Geng H, Chuang D, Yang F, *et al.* Prevalence and determinants of depression in caregivers of cancer patients: A systematic review and meta-analysis. *Medicine (Baltimore)* 2018;**97**:e11863. doi:10.1097/MD.0000000000011863

13 Birnbaum HG, Kessler RC, Kelley D, *et al.* Employer burden of mild, moderate, and

For peer review only

severe major depressive disorder: mental health services utilization and costs, and work performance. *Depress Anxiety* 2010;**27**:78–89. doi:10.1002/da.20580

14 Kessler RC. The costs of depression. *Psychiatr Clin North Am* 2012;**35**:1–14. doi:10.1016/j.psc.2011.11.005

15 Doran CM, Kinchin I. A review of the economic impact of mental illness. *Aust Heal Rev* 2019;**43**:43–8. doi:10.1071/AH16115

16 Hagedoorn M, Sanderman R, Bolks H, *et al.* Distress in couples coping with cancer: a meta-analysis and critical review of role and gender effects. *Psychol Bull* 2008;**134**:1–30.https://psycnet-apa-org.proxy3.library.mcgill.ca/fulltext/2007-19419-001.pdf (accessed 11 Jul 2019).

17 Hodges LJ, Humphris GM, Macfarlane G. A meta-analytic investigation of the relationship between the psychological distress of cancer patients and their carers. *Soc Sci Med* 2005;**60**:1–12. doi:10.1016/J.SOCSCIMED.2004.04.018

18 Smith GR, Williamson GM, Miller LS, *et al.* Depression and quality of informal care: a longitudinal investigation of caregiving stressors. *Psychol Aging* 2011;**26**:584–91. doi:10.1037/a0022263

19 Christie J, Smith GR, Williamson GM, *et al.* Quality of informal care is multidimensional. *Rehabil Psychol* 2009;**54**:173–81. doi:10.1037/a0015705

20 Cooney C, Howard R, Lawlor B. Abuse of vulnerable people with dementia by their carers: can we identify those most at risk? *Int J Geriatr Psychiatry* 2006;**21**:564–71. doi:10.1002/gps.1525

21 Beach SR, Schulz R, Williamson GM, *et al.* Risk Factors for Potentially Harmful Informal Caregiver Behavior. *J Am Geriatr Soc* 2005;**53**:255–61. doi:10.1111/j.1532-5415.2005.53111.x

22 Mosher CE, Given BA, Ostroff JS. Barriers to mental health service use among distressed family caregivers of lung cancer patients. *Eur J Cancer Care (Engl)* 2015;**24**:50–9. doi:10.1111/ecc.12203

23 Ploeg J, Ali MU, Markle-Reid M, *et al.* Caregiver-focused, web-based interventions: systematic review and meta-analysis (Part 2). *J Med Internet Res* 2018;**20**:e11247. doi:10.2196/11247

24 Christie HL, Martin JL, Connor J, *et al.* eHealth interventions to support caregivers of people with dementia may be proven effective, but are they implementation-ready? *Internet Interv* 2019;:100260. doi:10.1016/j.invent.2019.100260

25 Sin J, Henderson C, Spain D, *et al.* eHealth interventions for family carers of people with long term illness: A promising approach? *Clin Psychol Rev* 2018;**60**:109–25. doi:10.1016/J.CPR.2018.01.008

26 Pagliari C, Sloan D, Gregor P, *et al.* What is eHealth (4): a scoping exercise to map the field. *J Med Internet Res* 2005;**7**:e9. doi:10.2196/jmir.7.1.e9

27 Eysenbach G. What is e-health? *J Med Internet Res* 2001;**3**:e20. doi:10.2196/jmir.3.2.e20

For peer review only

28 Aldehaim AY, Alotaibi FF, Uphold CR, *et al.* The impact of technology-based interventions on informal caregivers of stroke survivors: a systematic review. *Telemed J e-health* 2016;**22**:223–31. doi:10.1089/tmj.2015.0062

29 Tang WP, Chan CW, So WK, *et al.* Web-based interventions for caregivers of cancer patients: A review of literatures. *Asia-Pacific J Oncol Nurs* 2014;**1**:9–15. doi:10.4103/2347-5625.135811

30 Kaltenbaugh D, Klem M, Hu L, *et al.* Using web-based interventions to support caregivers of patients with cancer: a systematic review. *Oncol Nurs Forum* 2015;**42**:156–64. doi:10.1188/15.ONF.156-164

31 McKechnie V, Barker C, Stott J. Effectiveness of computer-mediated interventions for informal carers of people with dementia—a systematic review. *Int Psychogeriatrics* 2014;**26**:1619–37. doi:10.1017/S1041610214001045

32 Wasilewski MB, Stinson JN, Cameron JI. Web-based health interventions for family caregivers of elderly individuals: A scoping review. *Int J Med Inform* 2017;**103**:109–38. doi:10.1016/J.IJMEDINF.2017.04.009

33 Christie HL, Bartels SL, Boots LMM, *et al.* A systematic review on the implementation of eHealth interventions for informal caregivers of people with dementia. *Internet Interv* 2018;**13**:51–9. doi:10.1016/J.INVENT.2018.07.002

34 Guay C, Auger C, Demers L, *et al.* Components and outcomes of Internet-based interventions for caregivers of older adults: systematic review. *J Med Internet Res* 2017;**19**:e313. doi:10.2196/jmir.7896

35 Heynsbergh N, Heckel L, Botti M, *et al.* Feasibility, useability and acceptability of technology-based interventions for informal cancer carers: a systematic review. *BMC Cancer* 2018;**18**. doi:10.1186/S12885-018-4160-9

36 Hopwood J, Walker N, McDonagh L, *et al.* Internet-based interventions aimed at supporting family caregivers of people with dementia: systematic review. *J Med Internet Res* 2018;**20**:e216. doi:10.2196/jmir.9548

37 Sherifali D, Ali MU, Ploeg J, *et al.* Impact of Internet-based interventions on caregiver mental health: systematic review and meta-analysis. *J Med Internet Res* 2018;**20**:e10668. doi:10.2196/10668

38 Ploeg J, Markle-Reid M, Valaitis R, *et al.* Web-based interventions to improve mental health, general caregiving outcomes, and general health for informal caregivers of adults with chronic conditions living in the community: rapid evidence review. *J Med Internet Res* 2017;**19**:e263. doi:10.2196/jmir.7564

39 Zhao Y, Feng H, Hu M, *et al.* Web-based interventions to improve mental health in home caregivers of people with dementia: meta-analysis. *J Med Internet Res* 2019;**21**:e13415. doi:10.2196/13415

40 Boots LMM, de Vugt ME, van Knippenberg RJM, *et al.* A systematic review of Internet-based supportive interventions for caregivers of patients with dementia. *Int J Geriatr Psychiatry* 2014;**29**:331–44. doi:10.1002/gps.4016

For peer review only

41 Riper H, Andersson G, Christensen H, *et al.* Theme issue on e-mental health: a growing field in internet research. *J Med Internet Res* 2010;**12**:e74. doi:10.2196/jmir.1713

42 Lal S. E-mental health: Promising advancements in policy, research, and practice. *Healthc Manag Forum* 2019;**32**:56–62. doi:10.1177/0840470418818583

43 Rebello TJ, Marques A, Gureje O, *et al.* Innovative strategies for closing the mental health treatment gap globally. *Curr Opin Psychiatry* 2014;**27**:308–14. doi:10.1097/YCO.0000000000000068

44 Naslund JA, Aschbrenner KA, Araya R, *et al.* Digital technology for treating and preventing mental disorders in low-income and middle-income countries: a narrative review of the literature. *The Lancet Psychiatry* 2017;**4**:486–500. doi:10.1016/S2215-0366(17)30096-2

45 Health Quality Ontario. Internet-delivered cognitive behavioural therapy for major depression and anxiety disorders: a health technology assessment. *Ont Health Technol Assess Ser* 2019;**19**:1–199.http://www.ncbi.nlm.nih.gov/pubmed/30873251 (accessed 11 Jul 2019).

46 Sin J, Norman I. Psychoeducational interventions for family members of people with schizophrenia: a mixed-method systematic review. *J Clin Psychiatry* 2013;**74**:e1145–62. doi:10.4088/JCP.12r08308

47 Folker AP, Mathiasen K, Lauridsen SM, *et al.* Implementing internet-delivered cognitive behavior therapy for common mental health disorders: A comparative case study of implementation challenges perceived by therapists and managers in five European internet services. *Internet Interv* 2018;**11**:60–70. doi:10.1016/J.INVENT.2018.02.001

48 Gitlin LN, Marx K, Stanley IH, *et al.* Translating evidence-based dementia caregiving interventions into practice: State-of-the-science and next steps. *Gerontologist* 2015;**55**:210–26. doi:10.1093/geront/gnu123

49 Damschroder LJ, Aron DC, Keith RE, *et al.* Fostering implementation of health services research findings into practice: a consolidated framework for advancing implementation science. *Implement Sci* 2009;**4**:50. doi:10.1186/1748-5908-4-50

50 Loudon K, Treweek S, Sullivan F, *et al.* The PRECIS-2 tool: designing trials that are fit for purpose. *BMJ* 2015;**350**:h2147. doi:10.1136/bmj.h2147

51 Rothwell PM. External validity of randomised controlled trials: "To whom do the results of this trial apply?" *Lancet* 2005;**365**:82–93. doi:10.1016/S0140-6736(04)17670-8

52 Aves T, Allan KS, Lawson D, *et al.* The role of pragmatism in explaining heterogeneity in meta-analyses of randomised trials: a protocol for a cross-sectional methodological review. *BMJ Open* 2017;**7**:e017887. doi:10.1136/BMJOPEN-2017-017887

53 Shamseer L, Moher D, Clarke M, *et al.* Preferred reporting items for systematic review and meta-analysis protocols (PRISMA-P) 2015: elaboration and explanation. *BMJ* 2015;**350**:g7647. doi:10.1136/bmj.g7647

54 Lizarondo L, Stern C, Carrier J, *et al.* Chapter 8: Mixed methods systematic reviews. In:

For peer review only

Aromataris E, Munn Z, eds. *Joanna Briggs Institute Reviewer's Manual*. The Joanna Briggs Institute 2017. https://wiki.joannabriggs.org/display/MANUAL/Chapter+8%3A+Mixed+methods+systematic+reviews (accessed 1 Jul 2019).

55 Costantino G, Montano N, Casazza G. When should we change our clinical practice based on the results of a clinical study? Searching for evidence: PICOS and PubMed. *Intern Emerg Med* 2015;**10**:525–7. doi:10.1007/s11739-015-1225-5

56 Centre for Reviews and Dissemination. *Systematic Reviews: CRD's guidance for undertaking reviews in health care*. 3rd ed. CRD, University of York 2009. www.york.ac.uk/inst/crd (accessed 10 Jul 2019).

57 Institute for Health Metrics and Evaluation. *GBD compare data visualization*. Seattle, WA: : IHME, University of Washington 2018. http://www.healthdata.org/data-visualization/gbd-compare (accessed 10 Jul 2019).

58 Sinha M. *Portrait of caregivers, 2012*. 2013. https://www150.statcan.gc.ca/n1/pub/89-652-x/89-652-x2013001-eng.htm (accessed 11 Jul 2019).

59 König HH, Leicht H, Brettschneider C, *et al.* The costs of dementia from the societal perspective: is care provided in the community really cheaper than nursing home care? *J Am Med Dir Assoc* 2014;**15**:117–26. doi:10.1016/j.jamda.2013.10.003

60 Bleijlevens MHC, Stolt M, Stephan A, *et al.* Changes in caregiver burden and health-related quality of life of informal caregivers of older people with Dementia: Evidence from the European RightTimePlaceCare prospective cohort study. *J Adv Nurs* 2015;**71**:1378–91. doi:10.1111/jan.12561

61 Gaugler JE, Mittelman MS, Hepburn K, *et al.* Clinically significant changes in burden and depression among dementia caregivers following nursing home admission. *BMC Med* 2010;**8**:85. doi:10.1186/1741-7015-8-85

62 Bernard-Dubenske LL, Wen K-Y, Gustafson DH, *et al.* Caregivers' differing needs across key experiences of the advanced cancer disease trajectory. *Palliat Support Care* 2008;**6**:265–72. doi:10.1017/S1478951508000400

63 Donker T, Griffiths KM, Cuijpers P, *et al.* Psychoeducation for depression, anxiety and psychological distress: A meta-analysis. *BMC Med* 2009;**7**:79. doi:10.1186/1741-7015-7-79

64 Thomas J, O'Mara-Eves A, Brunton G. Using qualitative comparative analysis (QCA) in systematic reviews of complex interventions: a worked example. *Syst Rev* 2014;**3**:67. doi:10.1186/2046-4053-3-67

65 Mohr DC, Spring B, Freedland KE, *et al.* The selection and design of control conditions for randomized controlled trials of psychological interventions. *Psychother Psychosom* 2009;**78**:275–84. doi:10.1159/000228248

66 Fitzpatrick R, Davey C, Buxton M, *et al.* Evaluating patient-based outcome measures for use in clinical trials: a review. *Health Technol Assess (Rockv)* 1998;**2**. doi:10.3310/hta2140

For peer review only

67 Radloff LS. The CES-D scale: a self-report depression scale for research in the general population. *Appl Psychol Meas* 1977;**1**:385–401. doi:10.1177/014662167700100306

68 Zigmond AS, Snaith RP. The hospital anxiety and depression scale. *Acta Psychiatr Scand* 1983;**67**:361–70. doi:10.1111/j.1600-0447.1983.tb09716.x

69 Cohen S, Kamarck T, Mermelstein R. A global measure of perceived stress. *J Health Soc Behav* 1983;**24**:385–96. doi:10.2307/2136404

70 Vis C, Ruwaard J, Finch T, *et al.* Toward an objective assessment of implementation processes for innovations in health care: psychometric evaluation of the Normalization Measure Development (NoMAD) questionnaire among mental health care professionals. *J Med Internet Res* 2019;**21**:e12376. doi:10.2196/12376

71 Proctor E, Silmere H, Raghavan R, *et al.* Outcomes for implementation research: conceptual distinctions, measurement challenges, and research agenda. *Adm Policy Ment Health* 2011;**38**:65–76. doi:10.1007/s10488-010-0319-7

72 Sajobi TT, Li G, Awosoga O, *et al.* A comparison of meta-analytic methods for synthesizing evidence from explanatory and pragmatic trials. *Syst Rev* 2018;**7**:19. doi:10.1186/s13643-017-0668-3

73 McGowan J, Sampson M, Salzwedel DM, *et al.* PRESS Peer Review of Electronic Search Strategies: 2015 guideline statement. *J Clin Epidemiol* 2016;**75**:40–6. doi:10.1016/J.JCLINEPI.2016.01.021

74 Lambert SD, Duncan LR, Kapellas S, *et al.* A descriptive systematic review of physical activity interventions for caregivers: effects on caregivers' and care recipients' psychosocial outcomes, physical activity levels, and physical health. *Ann Behav Med* 2016;**50**:907–19. doi:10.1007/s12160-016-9819-3

75 Woodford J, Farrand P, Richards D, *et al.* Psychological treatments for common mental health problems experienced by informal carers of adults with chronic physical health conditions (Protocol). *Syst Rev* 2013;**2**:9. doi:10.1186/2046-4053-2-9

76 Corry M, While A, Neenan K, *et al.* A systematic review of systematic reviews on interventions for caregivers of people with chronic conditions. *J Adv Nurs* 2015;**71**:718–34. doi:10.1111/jan.12523

77 Vis C, Mol M, Kleiboer A, *et al.* Improving implementation of eMental health for mood disorders in routine practice: systematic review of barriers and facilitating factors. *JMIR Ment Heal* 2018;**5**:e20. doi:10.2196/mental.9769

78 Wozney L, Mcgrath PJ, Gehring ND, *et al.* eMental healthcare technologies for anxiety and depression in childhood and adolescence: systematic review of studies reporting implementation outcomes. *JMIR Ment Heal* 2018;**5**:e48. doi:10.2196/mental.9655

79 Farrand P, Matthews J, Dickens C, *et al.* Psychological interventions to improve psychological well-being in people with dementia or mild cognitive impairment: systematic review and meta-analysis protocol. *BMJ Open* 2016;**6**:e009713. doi:10.1136/bmjopen-2015-009713

For peer review only

80 Bramer WM, Giustini D, de Jonge GB, *et al.* De-duplication of database search results for systematic reviews in EndNote. *J Med Libr Assoc* 2016;**104**:240–3. doi:10.3163/1536-5050.104.3.014

81 Ouzzani M, Hammady H, Fedorowicz Z, *et al.* Rayyan—a web and mobile app for systematic reviews. *Syst Rev* 2016;**5**:210. doi:10.1186/s13643-016-0384-4

82 Higgins JP, Savovic J, Page M, *et al.*, editors. *Revised Cochrane risk-of-bias tool for randomized trials (RoB 2)*. 2019. https://drive.google.com/file/d/1xBzD1BeRrXH2DHg58fnTNKinTkX5SLEt/view (accessed 22 Jul 2019).

83 Higgins JP, Altman D, Gotzsche P, *et al.* The Cochrane Collaboration's tool for assessing risk of bias in randomised trials. *BMJ* 2011;**343**:d5928. doi:10.1136/bmj.d5928

84 QSR International Pty Ltd. NVivo qualitative data analysis software. 2014.

85 Hill LG, Cooper BR, Parker LA. Qualitative comparative analysis: a mixed-method tool for complex implementation questions. *J Prim Prev* 2019;**40**:69–87. doi:10.1007/s10935-019-00536-5

86 Sullivan GM, Feinn R. Using effect size-or why the p value Is not enough. *J Grad Med Educ* 2012;**4**:279–82. doi:10.4300/JGME-D-12-00156.1

87 Davies EB, Morriss R, Glazebrook C. Computer-delivered and web-based interventions to improve depression, anxiety, and psychological well-being of university students: a systematic review and meta-analysis. *J Med Internet Res* 2014;**16**:e130. doi:10.2196/jmir.3142

88 van Beugen S, Ferwerda M, Hoeve D, *et al.* Internet-based cognitive behavioral therapy for patients with chronic somatic conditions: a meta-analytic review. *J Med Internet Res* 2014;**16**:e88. doi:10.2196/jmir.2777

89 Lorenzo-Luaces L, Johns E, Keefe JR. The generalizability of randomized controlled trials of self-guided Internet-based cognitive behavioral therapy for depressive symptoms: systematic review and meta-regression analysis. *J Med Internet Res* 2018;**20**:e10113. doi:10.2196/10113

90 Linde K, Sigterman K, Kriston L, *et al.* Effectiveness of psychological treatments for depressive disorders in primary care: systematic review and meta-analysis. *Ann Fam Med* 2015;**13**:56–68. doi:10.1370/afm.1719

91 Ragin C, Davey S. *Fuzzy-Set/Qualitative Comparative Analysis 3.0*. Irvine, Californai: : Department of Sociology, University of California 2016. http://www.socsci.uci.edu/~cragin/fsQCA/software.shtml (accessed 12 Jul 2019).

92 Ragin CC. User's guide to fuzzy-set/qualitative comparative analysis. Irvine, California: 2017. www.fsqca.com. (accessed 12 Jul 2019).

93 Thomas J, Harden A. Methods for the thematic synthesis of qualitative research in systematic reviews. *BMC Med Res Methodol* 2008;**8**:45. doi:10.1186/1471-2288-8-45

94 Popay J, Roberts H, Sowden A, *et al.* Guidance on the conduct of narrative synthesis in

For peer review only

systematic reviews. 2006. http://citeseerx.ist.psu.edu/viewdoc/download?doi=10.1.1.178.3100&rep=rep1&type=pdf (accessed 31 Jul 2019).

95 Lewis CC, Fischer S, Weiner BJ, *et al.* Outcomes for implementation science: an enhanced systematic review of instruments using evidence-based rating criteria. *Implement Sci* 2015;**10**:155. doi:10.1186/s13012-015-0342-x

96 Kerins C, McSharry J, Hayes C, *et al.* Barriers and facilitators to implementation of menu labelling interventions to support healthy food choices: a mixed methods systematic review protocol. *Syst Rev* 2018;**7**:88. doi:10.1186/s13643-018-0752-3

97 Pollock A, Campbell P, Struthers C, *et al.* Stakeholder involvement in systematic reviews: A scoping review. *Syst Rev* 2018;**7**:208. doi:10.1186/s13643-018-0852-0

98 Boote J, Baird W, Sutton A. Public involvement in the systematic review process in health and social care: A narrative review of case examples. *Health Policy (New York)* 2011;**102**:105–16. doi:10.1016/J.HEALTHPOL.2011.05.002

For peer review only

Implementation of e-mental health interventions for informal caregivers of adults with chronic diseases: a protocol for a mixed methods systematic review with a qualitative comparative analysis

PRISMA-P (Preferred Reporting Items for Systematic review and Meta-Analysis Protocols) 2015 checklist: recommended items to address in a systematic review protocol*

Section and topic	Item No	Checklist item	Location in text (page number)
**ADMINISTRATIVE INFORMATION**			
Title:			
Identification	1a	Identify the report as a protocol of a systematic review	1
Update	1b	If the protocol is for an update of a previous systematic review, identify as such	N/A
Registration	2	If registered, provide the name of the registry (such as PROSPERO) and registration number	N/A (submitted for registration, awaiting approval)
Authors:			
Contact	3a	Provide name, institutional affiliation, e-mail address of all protocol authors; provide physical mailing address of corresponding author	1
Contributions	3b	Describe contributions of protocol authors and identify the guarantor of the review	12
Amendments	4	If the protocol represents an amendment of a previously completed or published protocol, identify as such and list changes; otherwise, state plan for documenting important protocol amendments	5
Support:			
Sources	5a	Indicate sources of financial or other support for the review	12
Sponsor	5b	Provide name for the review funder and/or sponsor	12
Role of sponsor or funder	5c	Describe roles of funder(s), sponsor(s), and/or institution(s), if any, in developing the protocol	12
**INTRODUCTION**			
Rationale	6	Describe the rationale for the review in the context of what is already known	3-4
Objectives	7	Provide an explicit statement of the question(s) the review will address with reference to participants, interventions, comparators, and outcomes (PICO)	4
**METHODS**			

For peer review only

Eligibility criteria	8	Specify the study characteristics (such as PICO, study design, setting, time frame) and report characteristics (such as years considered, language, publication status) to be used as criteria for eligibility for the review	5-7
Information sources	9	Describe all intended information sources (such as electronic databases, contact with study authors, trial registers or other grey literature sources) with planned dates of coverage	7
Search strategy	10	Present draft of search strategy to be used for at least one electronic database, including planned limits, such that it could be repeated	7 & supplementary appendix 3
Study records:			
Data management	11a	Describe the mechanism(s) that will be used to manage records and data throughout the review	8
Selection process	11b	State the process that will be used for selecting studies (such as two independent reviewers) through each phase of the review (that is, screening, eligibility and inclusion in meta-analysis)	8
Data collection process	11c	Describe planned method of extracting data from reports (such as piloting forms, done independently, in duplicate), any processes for obtaining and confirming data from investigators	9
Data items	12	List and define all variables for which data will be sought (such as PICO items, funding sources), any pre-planned data assumptions and simplifications	9 & supplementary appendix 4
Outcomes and prioritization	13	List and define all outcomes for which data will be sought, including prioritization of main and additional outcomes, with rationale	6
Risk of bias in individual studies	14	Describe anticipated methods for assessing risk of bias of individual studies, including whether this will be done at the outcome or study level, or both; state how this information will be used in data synthesis	8-9
Data synthesis	15a	Describe criteria under which study data will be quantitatively synthesised	6-7 & 9
	15b	If data are appropriate for quantitative synthesis, describe planned summary measures, methods of handling data and methods of combining data from studies, including any planned exploration of consistency (such as I^2, Kendall's τ)	9-10
	15c	Describe any proposed additional analyses (such as sensitivity or subgroup analyses, meta-regression)	9-10
	15d	If quantitative synthesis is not appropriate, describe the type of summary planned	10-11
Meta-bias(es)	16	Specify any planned assessment of meta-bias(es) (such as publication bias across studies, selective reporting within studies)	8-9
Confidence in cumulative evidence	17	Describe how the strength of the body of evidence will be assessed (such as GRADE)	N/A

N/A not applicable

*** It is strongly recommended that this checklist be read in conjunction with the PRISMA-P Explanation and Elaboration (cite when available) for important clarification on the items. Amendments to a review protocol should be tracked and dated. The copyright for PRISMA-P (including checklist) is held by the PRISMA-P Group and is distributed under a Creative Commons Attribution Licence 4.0.**

From: Shamseer L, Moher D, Clarke M, Ghersi D, Liberati A, Petticrew M, Shekelle P, Stewart L, PRISMA-P Group. Preferred reporting items for systematic review and meta-analysis protocols (PRISMA-P) 2015: elaboration and explanation. BMJ. 2015 Jan 2;349(jan02 1):g7647.

For peer review only

PRESS Guideline — Search Submission & Peer Review Assessment

SEARCH SUBMISSION: THIS SECTION TO BE FILLED IN BY THE SEARCHER

Searcher: Chelsea Coumoundouros
Email: chelsea.coumoundouros@kbh.uu.se
Date Submitted: 2019/09/18
Date requested by: 2019/10/09

Systematic Review Title:

Implementation of e-Mental Health interventions for informal caregivers of adults with chronic diseases: a protocol for a mixed methods systematic review with a qualitative comparative analysis

This search strategy is …

X	My PRIMARY (core) database strategy — First time submitting a strategy for search question and database
	My PRIMARY (core) strategy — Follow-up review NOT the first time submitting a strategy for search question and database. If this is a response to peer review, itemize the changes made to the review suggestions
	SECONDARY search strategy— First time submitting a strategy for search question and database
	SECONDARY search strategy — NOT the first time submitting a strategy for search question and database. If this is a response to peer review, itemize the changes made to the review suggestions

Database

(i.e., MEDLINE,CINAHL…): *[mandatory]*

MEDLINE

Interface

(i.e., Ovid, EBSCO…): *[mandatory]*

PubMed

Research Question

(Describe the purpose of the search) *[mandatory]*

The aim of this review is to determine factors related to the successful implementation of e-mental health interventions for caregivers of adults with chronic diseases. Two approaches will be used to investigate this. First, studies with more pragmatic designs will be used exclusively to determine which combinations of intervention or implementation characteristics are associated with effective interventions using a qualitative comparative analysis. Second, reports regarding the implementation of e-mental health interventions will be thematically synthesized to establish the common barriers and facilitators to e-mental health implementation.

For peer review only - http://bmjopen.bmj.com/site/about/guidelines.xhtml

PICO Format

(Outline the PICOs for your question — i.e., Patient, Intervention, Comparison, Outcome, and Study Design — as applicable)

**P**	Unpaid adult caregivers of adults with either heart disease, stroke, cancer, diabetes, dementia or chronic obstructive pulmonary disease.
**I**	Any e-mental health intervention targeting the treatment of common mental health difficulties (e.g. depression, anxiety) in caregivers
**C**	**For the qualitative comparative analysis:** Studies must use a non-active control (no treatment, wait-list control, treatment as usual, non-specific treatment component control or education on the care recipient's condition). **For the thematic synthesis:** No restriction based on presence/absence of control group or control type.
**O**	**For the qualitative comparative analysis:** Depression, anxiety, stress or distress measured using an instrument with at least acceptable reliability (Cronbach's alpha ≥ 0.7). **For the thematic synthesis:** Barriers and/or facilitators to implementation.
**S**	**For the qualitative comparative analysis:** Studies must be randomized controlled effectiveness studies. **For the thematic synthesis:** No restriction based on study design.

Inclusion Criteria *[optional]*

P: Caregivers and care recipients must be aged 18 years or older. Chronic health conditions eligible for inclusion are listed in the PICOS above.

I: Interventions must be primarily delivered using Internet technology and target the caregiver's mental health. Interventions may be supplemented by additional forms of support (e.g. telephone call, face-to-face session). Any type of therapy will be included.

O: For the thematic synthesis: Barriers and facilitators can include factors related to any aspect of the Consolidated Framework for Implementation Research or the implementation outcome framework developed by Proctor and colleagues.

S: For the qualitative comparative analysis: Effectiveness will be defined as any study with a mean score of 3 or more using the PRECIS-2 tool.

For peer review only - http://bmjopen.bmj.com/site/about/guidelines.xhtml

Exclusion Criteria *[optional]*

**P:** Studies that focus on caregivers with severe mental health conditions, caregivers providing care to non-community dwelling care recipients or care recipients at the palliative phase of disease will be excluded.<br><br>**I:** Interventions delivered using the telephone, CD-ROM or video (including Skype) alone will be excluded.<br><br>**C: For the qualitative comparative analysis:** Studies using psychoeducation, education on mental health or active controls will be excluded.<br><br>**Date limit:** Studies published prior to 2007 will be excluded. Technologies from work published prior to 2007 may be outdated and other reviews have shown that production of publications involving eHealth began to rise from 2007 onwards<br><br>**Language restriction:** Studies written in languages other than English or Swedish will be excluded.

Was a search filter applied?

Yes ■ No ☐

If YES, which one(s) (e.g., Cochrane RCT filter, PubMed Clinical Queries filter)? Provide the source if this is a published filter. *[mandatory if YES to previous question — textbox]*

Date limit was applied as part of the search.

Other notes or comments you feel would be useful for the peer reviewer? ***[optional]***

There are no terms related to implementation as (1) this is not the only area we are interested in (also need effectiveness studies) and (2) implementation can be referred to in many different ways and is sometimes not mentioned at all in the title or abstract of studies, so trying to create terms about this topic may lead to poor retrieval of relevant work

Please copy and paste your search strategy here, exactly as run, including the number of hits per line. ***[mandatory]***

For peer review only - http://bmjopen.bmj.com/site/about/guidelines.xhtml

Database: PubMed. Final number of results from the full search 3265
Date Restriction: January 1, 2007 to present

1: Caregivers	2: Chronic Health Conditions	3: eHealth/ Technology	4: Mental Health	5: Therapy
**Title/Abstract**	**Title/Abstract**	**Title/Abstract**	**Title/Abstract**	**Title/Abstract**
Caregiver 19465	Cancer 1019721	eHealth 2990	Mental health 92203	Mental health service 3222
Caregivers 35586	Tumor 615354	e-health 2140	Mood 45028	Mental health services 10159
Care-giver 301	Tumors 279910	e-mental health 213	Mood disorder 3595	Mental healthcare 1741
Care-givers 1034	Tumours 49415	emental health 30	Depression 185606	Therapy 923016
Carer 3436	Tumour 95450	mhealth 3596	Depressive 72042	Therapies 201143
Carers 8055	Neoplasm 34474	m-health 462	Depressed 30180	Treatment 2180941
Informal carer 94	Neoplasms 51500	Smartphone 7429	Affective disorder 2287	Treatments 283397
Informal carers 575	Oncology 65177	Cell phone 1702	Affect 366439	Interventions 307575
Informal caregiver 460	Carcinoma 275810	Cellular phone 362	Negative affect 7192	Intervention 397118
Informal caregivers 1917	Malignan* 271316	Mobile phone 5430	Dysthymia 886	Program 229608
Family 412900	Melanoma 56126	Mobile app 1046	Dysphoria 1871	Programs 158202
Families 124835	Metastasis 147467	Mobile apps 991	Melancholic 594	Programmes 36810
Spouse 4404	Lymphoma 76891	Mobile application 1252	Anxiety 117199	Programme 52551
Spousal 2026	Leukemia 86466	Mobile applications 1283	Burden 141973	Psychological 117677
Spouses 4578	Diabetes 309446	App 16442	Distress 63488	Psychologist 2715
Sibling 10277	Diabetes mellitus 108204	Apps 4615	Stress 465139	Psychoeducation 2202
Siblings 15168	Type 1 diabetes 27354	Application 442621	Well being 49008	Psycho-education 518
Husband 2380	Type 2 diabetes 95548	Applications 318200	Emotion 29552	CBT 8475
Husbands 2371	COPD 32037	iPad 1114	Emotional 95270	Cognitive behavioural therapy 3103
Wife 1919	Chronic obstructive pulmonary	Computer 94973		Cognitive therapy 1960
Wives 1829	disease 32548	Tablet 14085	**MeSH**	Health education 13665
Partner 47483	COAD 147	Computer based 6611	Mood disorders 55994	Problem solving 9444
Partners 51514	Chronic obstructive airway disease	Computer assistive	Depression 108610	Problem solving therapy 335
Parents 92138	80	technology 421	Anxiety 42892	PST 1976
Parent 69723	Chronic obstructive lung disease	Technology 205306	Anxiety disorders 28551	ACT 140661
Friend 6741	1842	Technologies 99160	Stress, psychological 66704	Commitment therapy 809
Friends 16333	Chronic airflow obstruction 102	Electronic communication	Emotions 119893	Behaviour 108763
Relatives 28499	Stroke 150135	757	Mental health 21517	Behavioural 45351
Relative 436237	Cerebrovascular accident 2137	Email 4949		Therapist 9305
Couple 18462	CVA 1540	E-mail 4654		Support 563829
Couples 20787	Cerebral stroke 676	Text messaging 1952		Mindful 2490
Mother 51594	Acute stroke 9906	Text message 1338		Mindfulness 6250
Mothers 64116	Acute cerebrovascular accident 58	Internet 36172		Dialectic behaviour therapy 5
Fathers 9825	Cerebrovascular stroke 133	Internet based 6623		Psychotherapy 13408
Father 10914	Brain vascular accident 2	Wireless 10928		Relaxation 53119
Support person 216	Cerebrovascular apoplexy 2	Digital 71968		Meditation 3440

For peer review only

For peer review only - http://bmjopen.bmj.com/site/about/guidelines.xhtml

Support persons 226<br>Next of kin 770<br>Significant other 583<br>Significant others 1502<br><br>**MeSH**<br>Caregivers 23052<br>Family 134295<br>Spouses 5686<br>Siblings 7235<br>Parents 57525<br>Friends 3754<br>Mothers 23221<br>Fathers 4249	Apoplexy 938<br>Heart disease 71238<br>Cardiovascular disease 89039<br>CVD 27460<br>Ischemic heart disease 10129<br>IHD 2703<br>Coronary artery disease 45519<br>CAD 25678<br>Angina 16189<br>Myocardial infarction 85190<br>MI 28920<br>Heart attack 2262<br>Cardiac event 2336<br>Cardiac disease 7437<br>Dementia 64525<br>Alzheimer* 90032<br>Alzheimer's disease 73741<br>Vascular dementia 3528<br>Frontotemporal dementia 4920<br>FTD 2656<br>Lewy bodies 4361<br>Lewy body 2324<br>Neurocognitive disorder 739<br>Neurocognitive disorders 1551<br>Mild cognitive impairment 13740<br>Memory impair* 8743<br>Cognitive decline 16542<br>Chronic illness 7067<br>Chronic illnesses 2903<br>Chronic diseases 23041<br>Chronic disease 23460<br>Chronic condition 3482<br>Chronic conditions 10263<br>Multi-morbidity 476<br>Multimorbidity 3160<br>Multimorbidities 165<br>Multi-morbidities 66<br>Long-term health condition 39	Online 91273<br>On-line 10976<br>Virtual 42066<br>ICT 3885<br>Communication technology 1639<br>Web 82786<br>Web based 23890<br>Website 13756<br>Web page 528<br>Multimedia 2881<br>e-learning 2196<br>Online social network 154<br>e-therapy 146<br>e-therapies 23<br>etherapies 1<br>etherapy 10<br>iCBT 589<br>cCBT 158<br><br>**MeSH**<br>Cell phone 8834<br>Mobile application 4432<br>Computers 14176<br>Therapy, computer assisted 44315<br>Wireless technology 3136<br>Information technology 237<br>Technology 210207<br>Electronic mail 1850<br>Internet 51939<br>Online social networking 57<br>Virtual reality 1079<br>Web browser 962<br>Multimedia 951		Behavior 363942<br>Behavioral 167313<br>Cognitive behavioral therapy 7242<br>Dialectic behavior therapy 10<br>Behavioural activation 328<br>Behavioral activation 862<br>Counseling 38031<br>Counselling 14505<br>Cognitive reframing 59<br>Cognitive restructuring 506<br>Self-help 3173<br>Self-management 14367<br><br>**MeSH**<br>Mental health services 37274<br>Health education 103830<br>Problem solving 9350<br>Psychotherapy 69239<br>Relaxation 8308<br>Self-management 1348<br>Self-help groups 3265

For peer review only

	**MeSH**<br>Neoplasms 1299462<br>Diabetes mellitus 192881<br>Pulmonary disease, chronic obstructive 31584<br>Stroke 83194<br>Cardiovascular diseases 805860<br>Dementia 81697<br>Cognition disorders 56940<br>Chronic disease 85925<br>Multimorbidity 503			
**Total for concept 1:**<br>**1 278 504**	**Total for concept 2:**<br>**3 399 238**	**Total for concept 3:**<br>**1 504 756**	**Total for concept 4:**<br>**1 400 395**	**Total for concept 5:**<br>**4 478 921**

Note: All MeSH terms are exploded

Total for complete search (includes date restriction): 3265

Date of search: September 4, 2019

For peer review only

Raw PubMed Search

(((((caregiver[Title/Abstract] OR caregivers[Title/Abstract] OR care-giver[Title/Abstract] OR care-givers[Title/Abstract] OR carer[Title/Abstract] OR carers[Title/Abstract] OR informal carer[Title/Abstract] OR informal carers[Title/Abstract] OR informal caregiver[Title/Abstract] OR informal caregivers[Title/Abstract] OR family[Title/Abstract] OR families[Title/Abstract] OR spouse[Title/Abstract] OR spousal[Title/Abstract] OR spouses[Title/Abstract] OR sibling[Title/Abstract] OR siblings[Title/Abstract] OR husband[Title/Abstract] OR husbands[Title/Abstract] OR wife[Title/Abstract] OR wives[Title/Abstract] OR partner[Title/Abstract] OR partners[Title/Abstract] OR parents[Title/Abstract] OR parent[Title/Abstract] OR friend[Title/Abstract] OR friends[Title/Abstract] OR relatives[Title/Abstract] OR relative[Title/Abstract] OR couple[Title/Abstract] OR couples[Title/Abstract] OR mother[Title/Abstract] OR mothers[Title/Abstract] OR fathers[Title/Abstract] OR father[Title/Abstract] OR support person[Title/Abstract] OR support persons[Title/Abstract] OR next of kin[Title/Abstract] OR significant other[Title/Abstract] OR significant others[Title/Abstract] OR caregivers[MeSH terms] OR family[MeSH terms] OR spouses[MeSH terms] OR siblings[MeSH terms] OR parents[MeSH terms] OR friends[MeSH terms] OR mothers[MeSH terms] OR fathers[MeSH terms]) AND (cancer[Title/Abstract] OR tumor[Title/Abstract] OR tumors[Title/Abstract] OR tumours[Title/Abstract] OR tumour[Title/Abstract] OR neoplasm[Title/Abstract] OR neoplasms[Title/Abstract] OR oncology[Title/Abstract] OR carcinoma[Title/Abstract] OR malignan*[Title/Abstract] OR melanoma[Title/Abstract] OR metastasis[Title/Abstract] OR lymphoma[Title/Abstract] OR leukemia[Title/Abstract] OR diabetes[Title/Abstract] OR diabetes mellitus[Title/Abstract] OR Type 1 diabetes[Title/Abstract] OR Type 2 diabetes[Title/Abstract] OR COPD[Title/Abstract] OR chronic obstructive pulmonary disease[Title/Abstract] OR COAD[Title/Abstract] OR chronic obstructive airway disease[Title/Abstract] OR chronic obstructive lung disease[Title/Abstract] OR chronic airflow obstruction[Title/Abstract] OR stroke[Title/Abstract] OR cerebrovascular accident[Title/Abstract] OR CVA[Title/Abstract] OR cerebral stroke[Title/Abstract] OR acute stroke[Title/Abstract] OR acute cerebrovascular accident[Title/Abstract] OR cerebrovascular stroke[Title/Abstract] OR brain vascular accident[Title/Abstract] OR cerebrovascular apoplexy[Title/Abstract] OR apoplexy[Title/Abstract] OR heart disease[Title/Abstract] OR cardiovascular disease[Title/Abstract] OR CVD[Title/Abstract] OR ischemic heart disease[Title/Abstract] OR IHD[Title/Abstract] OR coronary artery disease[Title/Abstract] OR CAD[Title/Abstract] OR angina[Title/Abstract] OR myocardial infarction[Title/Abstract] OR MI[Title/Abstract] OR heart attack[Title/Abstract] OR cardiac event[Title/Abstract] OR cardiac disease[Title/Abstract] OR dementia[Title/Abstract] OR alzheimer*[Title/Abstract] OR alzheimer's disease[Title/Abstract] OR vascular dementia[Title/Abstract] OR frontotemporal dementia[Title/Abstract] OR FTD[Title/Abstract] OR Lewy body[Title/Abstract] OR Lewy bodies[Title/Abstract] OR neurocognitive disorder[Title/Abstract] OR neurocognitive disorders[Title/Abstract] OR mild cognitive impairment[Title/Abstract] OR memory impair*[Title/Abstract] OR cognitive decline[Title/Abstract] OR chronic illness[Title/Abstract] OR chronic illnesses[Title/Abstract] OR chronic diseases[Title/Abstract] OR chronic disease[Title/Abstract] OR chronic condition[Title/Abstract] OR chronic conditions[Title/Abstract] OR multi-morbidity[Title/Abstract] OR multimorbidity[Title/Abstract] OR multimorbidities[Title/Abstract] OR multi-morbidities[Title/Abstract] OR long-term health condition[Title/Abstract] OR neoplasms[MeSH

For peer review only

terms] OR diabetes mellitus[MeSH terms] OR pulmonary disease, chronic obstructive[MeSH terms] OR stroke[MeSH terms] OR cardiovascular diseases[MeSH terms] OR dementia[MeSH terms] OR Cognition disorders[MeSH terms] OR chronic disease[MeSH terms] OR multimorbidity[MeSH terms]) AND (eHealth[Title/Abstract] OR e-health[Title/Abstract] OR e-mental health[Title/Abstract] OR emental health[Title/Abstract] OR mhealth[Title/Abstract] OR m-health[Title/Abstract] OR smartphone[Title/Abstract] OR cell phone[Title/Abstract] OR cellular phone[Title/Abstract] OR mobile phone[Title/Abstract] OR mobile app[Title/Abstract] OR mobile apps[Title/Abstract] OR mobile applications[Title/Abstract] OR mobile application[Title/Abstract] OR app[Title/Abstract] OR application[Title/Abstract] OR applications[Title/Abstract] OR apps[Title/Abstract] OR iPad[Title/Abstract] OR computer[Title/Abstract] OR tablet[Title/Abstract] OR computer based[Title/Abstract] OR computer assistive technology[Title/Abstract] OR technology[Title/Abstract] OR technologies[Title/Abstract] OR electronic communication[Title/Abstract] OR email[Title/Abstract] OR e-mail[Title/Abstract] OR text messaging[Title/Abstract] OR text message[Title/Abstract] OR internet[Title/Abstract] OR internet based[Title/Abstract] OR wireless[Title/Abstract] OR online[Title/Abstract] OR digital[Title/Abstract] OR on-line[Title/Abstract] OR virtual[Title/Abstract] OR ICT[Title/Abstract] OR communication technology[Title/Abstract] OR web[Title/Abstract] OR web based[Title/Abstract] OR website[Title/Abstract] OR web page[Title/Abstract] OR multimedia[Title/Abstract] OR e-learning[Title/Abstract] OR online social network[Title/Abstract] OR iCBT[Title/Abstract] OR cCBT[Title/Abstract] OR e-therapy[Title/Abstract] OR etherapy[Title/Abstract] OR etherapies[Title/Abstract] OR e-therapies[Title/Abstract] OR cell phone[MeSH terms] OR mobile application[MeSH terms] OR computers[MeSH terms] OR therapy, computer assisted[MeSH terms] OR wireless technology[MeSH terms] OR information technology[MeSH terms] OR technology[MeSH terms] OR electronic mail[MeSH terms] OR internet[MeSH terms] OR online social networking[MeSH terms] OR virtual reality[MeSH terms] OR web browser[MeSH terms] OR multimedia[MeSH terms]) AND (mental health[Title/Abstract] OR mood[Title/Abstract] OR mood disorder[Title/Abstract] OR depression[Title/Abstract] OR depressive[Title/Abstract] OR depressed[Title/Abstract] OR affective disorder[Title/Abstract] OR affect[Title/Abstract] OR negative affect[Title/Abstract] OR dysthymia[Title/Abstract] OR dysphoria[Title/Abstract] OR melancholic[Title/Abstract] OR anxiety[Title/Abstract] OR burden[Title/Abstract] OR distress[Title/Abstract] OR stress[Title/Abstract] OR well being[Title/Abstract] OR emotion[Title/Abstract] OR emotional[Title/Abstract] OR mood disorders[MeSH terms] OR depression[MeSH terms] OR anxiety[MeSH terms] OR anxiety disorders[MeSH terms] OR stress, psychological[MeSH terms] OR emotions[MeSH terms] OR mental health[MeSH terms]) AND (mental health service[Title/Abstract] OR mental health services[Title/Abstract] OR mental healthcare[Title/Abstract] OR therapy[Title/Abstract] OR therapies[Title/Abstract] OR treatment[Title/Abstract] OR treatments[Title/Abstract] OR interventions[Title/Abstract] OR intervention[Title/Abstract] OR program[Title/Abstract] OR programs[Title/Abstract] OR programmes[Title/Abstract] OR programme[Title/Abstract] OR psychological[Title/Abstract] OR psychologist[Title/Abstract] OR psychoeducation[Title/Abstract] OR psycho-education[Title/Abstract] OR CBT[Title/Abstract] OR cognitive behavioural therapy[Title/Abstract] OR cognitive therapy[Title/Abstract] OR health education[Title/Abstract] OR problem solving[Title/Abstract] OR problem solving therapy[Title/Abstract] OR PST[Title/Abstract] OR ACT[Title/Abstract] OR commitment therapy[Title/Abstract] OR behaviour[Title/Abstract] OR

For peer review only - http://bmjopen.bmj.com/site/about/guidelines.xhtml

behavioural[Title/Abstract] OR therapist[Title/Abstract] OR support[Title/Abstract] OR mindful[Title/Abstract] OR mindfulness[Title/Abstract] OR dialectic behaviour therapy[Title/Abstract] OR psychotherapy[Title/Abstract] OR relaxation[Title/Abstract] OR meditation[Title/Abstract] OR behavior[Title/Abstract] OR behavioral[Title/Abstract] OR cognitive behavioral therapy[Title/Abstract] OR behavioural activation[Title/Abstract] OR behavioral activation[Title/Abstract] OR dialectic behavior therapy[Title/Abstract] OR counseling[Title/Abstract] OR counselling[Title/Abstract] OR cognitive reframing[Title/Abstract] OR cognitive restructuring[Title/Abstract] OR self-help[Title/Abstract] OR self-management[Title/Abstract] OR mental health services[MeSH terms] OR health education[MeSH terms] OR problem solving[MeSH terms] OR psychotherapy[MeSH terms] OR relaxation[MeSH terms] OR self-management[MeSH terms] OR self-help groups[MeSH terms]))))) AND ("2007/01/01"[Date - Publication] : "3000"[Date - Publication])

For peer review only

PEER REVIEW ASSESSMENT #1: THIS SECTION TO BE FILLED IN BY THE REVIEWER

Reviewer: Professor Mariët Hagedoorn & Truus van Ittersum	Email: mariet.hagedoorn@umcg.nl	Date completed: 2019/10/03

1. TRANSLATION

A ---No revisions	☐
B --- Revision(s) suggested	☐
C --- Revision(s) required	X

If “B” or “C,” please provide an explanation or example:

Does the search strategy match the research question/PICO?
I’m not familiar with the PRECIS-2 tool. Do you use it to rate the effectiveness of an internet technology intervention in reducing distress in caregivers as compared to a non-active control group?
Considering your overall question, I would expect that you would assess the effectiveness/successfulness of the implementation?
Are the search concepts clear?
Are there too many or too few PICO elements included?
You could consider not applying the fifth concept, but I can see the point why you do.
Are the search concepts too broad or too narrow?
Does the search retrieve too many or too few records?
Are unconventional or complex strategies explained?
The main focus is on successful implementation, which I find a relevant and timely issue to address in a review. However, the two approaches are not clear to me. For the first approach (which combination of intervention and implementation characteristics are associated with effective interventions), you want to include RCTs. To my understanding RCTs test the effectiveness of an intervention and usually does not include the aim of implementation. Will these studies be relevant to answer your overall question “which factors are related to successful implementation”? It would be helpful to clarify this.
On a practical note, the combination of two approaches appears to make the coding rather complex. Wouldn’t it be better to do this in phases? First RCTs, than the remaining literature for approach 2?

I would advise to clarify the two approaches. As I commented above, it is not clear to me how approach one answers your overall research question. I also advise you to reconsider whether you really need two approaches, with specific inclusion criteria. In case you do need both approaches, would it be possible to use a two-step coding process and first search/code RCTs and than apply the second strategy?

<u>Author response:</u>
Thank you for your feedback. We believe that approach one (the qualitative comparative analysis) can produce results that relate to the overall research question regarding implementation of e-mental health interventions for caregivers. Approach one will only utilize pragmatic randomized controlled trials in the analysis. Pragmatic trials use conditions (e.g. the setting, follow up etc.) that more closely reflect how the interventions would be used in real-world settings. Therefore, pragmatic trials are more likely than explanatory trials to include details concerning implementation, which can be included in the qualitative comparative analysis to assess whether certain implementation factors are important for interventions effectiveness. Further, pragmatic trials examine effectiveness in near to real-world settings, and as such, provide estimates of intervention effectiveness that may better reflect estimates of effectiveness that may be found if the intervention was implemented in real-world practices. The PRECIS-2 tool evaluates how pragmatic the design of a randomized controlled trial is. By using this tool in the screening process, only trials with more pragmatic features will be included in the qualitative comparative analysis. The results of the qualitative comparative analysis can also be contrasted to the barriers and

For peer review only - http://bmjopen.bmj.com/site/about/guidelines.xhtml

facilitators identified in the thematic synthesis to determine if, for example, a factor identified as a facilitator to implementation in the thematic synthesis was also identified as important for intervention effectiveness in the qualitative comparative analysis.

We agree, the two approaches adds complexity to the literature screening process, however, if we were to only search for RCTs as a first step, we may miss many reports on implementation of interventions relevant to our research question.

2. BOOLEAN AND PROXIMITY OPERATORS

A ---No revisions	X
B --- Revision(s) suggested	☐
C --- Revision(s) required	☐

If "B" or "C," please provide an explanation or example:

Are Boolean or proximity operators used correctly?
Is the use of nesting with brackets appropriate and effective for the search?
If NOT is used, is this likely to result in any unintended exclusions?
Could precision be improved by using proximity operators (e.g. adjacent, near, within) or phrase searching instead of AND?
Is the width of proximity operators suitable (e.g. might adj5 pick up more variants than adj2)?

3. SUBJECT HEADINGS

A ---No revisions	X
B --- Revision(s) suggested	☐
C --- Revision(s) required	☐

If "B" or "C," please provide an explanation or example:

Are the subject headings relevant?
Are any relevant subject headings missing (e.g. previous index terms)?
Are any subject headings too broad or too narrow?
Are subject headings exploded where necessary and vice versa?
Are major headings ("starring" or restrict to focus) used? If so, if there adequate justification?
Are subheadings missing?
Are subheadings attached to subject headings? (Floating subheadings may be preferred)
Are floating subheadings relevant and used appropriately?
Are both subject headings and terms in free text used for each concept?

4. TEXT WORD SEARCHING

A ---No revisions	☐
B --- Revision(s)suggested	X
C --- Revision(s) required	☐

If "B" or "C," please provide an explanation or example:

General: there is an overload of terms. Some specific terms are not necessary as publications are already found by more general terms (<diabetes> also finds <diabetes mellitus>, <affect> also covers <negative affect>.

For peer review only

Does the search include all spelling variants in free text (e.g. UK versus US spelling)?
Does the search include all synonyms or antonyms (e.g. opposites)?
Does the search capture relevant truncation (e.g. is truncation at the correct place)?
Using the <*> helps narrowing down the number of terms, without loosing results. <Husband*> will also find <husbands>, <caregiver*> will also find <caregivers>. There are a lot of these terms in the list.
Are acronyms or abbreviations used appropriately? Do they capture irrelevant material? Are the full terms also included?
Are the keywords specific enough or too broad? Are too many or too few keywords used? Are stop words used?
Term “Technology”: Yields many results. Does not seem very relevant. Maybe skip this term?
Term “Web”: Yields mostly “Web of Science” OR “Web of Knowledge” hits. Maybe exclude? Reconsider?
Term “Multimorbidity”: Why multimorbidity? Populations should have a chronic illness and they are specified.
Term “long-term health condition”: To vague.
Have the appropriate fields been searched (e.g. is the choice of text world fields (.tw.) or all fields (.af.) appropriate? Are there any other fields to be included or excluded (database-specific)?
Should any long strings be broken into several shorter search statements?

Author response:
Thank you for these comments. Your feedback has been incorporated into a revised search strategy which has eliminated unnecessary terms and used more term truncations.

Although the terms “technology” and “web” are very broad, we feel they should remain in the primary search strategy as they may yield relevant results pertaining to technological or web based interventions. The terms “multimorbidity” and “long-term health condition” are included as we have come across publications which refer to the population under investigation using more generic terms, such as having multimorbidities or chronic diseases, with the specific health conditions only specified in the main text. Therefore, to ensure the search captures all publications that include caregivers of individuals with the health conditions of interest to this review, we included these more general terms.

5. SPELLING, SYNTAX, AND LINE NUMBERS

A ---No revisions	X
B --- Revision(s)suggested	☐
C --- Revision(s) required	☐

If “B” or “C,” please provide an explanation or example:

Are there any spelling errors
Are there errors in system syntax (e.g. the use of a truncation symbol from a different search interface)?
Are there incorrect line combinations or orphan lines (e.g. lines that are not referred to in the final summation that could indicate an error in an AND or OR statement)?

6. LIMITS AND FILTERS

A ---No revisions	X
B --- Revision(s) suggested	☐
C --- Revision(s) required	☐

If “B” or “C,” please provide an explanation or example:

For peer review only - http://bmjopen.bmj.com/site/about/guidelines.xhtml

Are all limits and filters used appropriately and are they relevant given the research question?
Are all limits and filters used appropriately and are they relevant for the database?
Are any potentially helpful limits or filters missing? Are the limits or filters too broad or too narrow? Can any limits or filters be added or removed?
Are sources cited for the filters used?

OVERALL EVALUATION (Note: If one or more "revision required" is noted above, the response below must be "revisions required".)

A ---No revisions	☐
B --- Revision(s) suggested	☐
C --- Revision(s) required	X

For peer review only

Additional comments: see above

For peer review only - http://bmjopen.bmj.com/site/about/guidelines.xhtml

PEER REVIEW ASSESSMENT #2: THIS SECTION TO BE FILLED IN BY THE REVIEWER

Reviewer:	Email:	Date completed: 2019/10/07
Dr. Nathan Davies	n.m.davies@ucl.ac.uk	

1. TRANSLATION

A ---No revisions	X
B --- Revision(s) suggested	☐
C --- Revision(s) required	☐

If "B" or "C," please provide an explanation or example:

Does the search strategy match the research question/PICO?
Yes
Are the search concepts clear?
Yes
Are there too many or too few PICO elements included?
This is a very broad search and large area to study, it is doable but you may consider splitting the review into two.
Are the search concepts too broad or too narrow?
No, they are broad but needed for the question
Does the search retrieve too many or too few records?
My only concern is that this is going to be a very large review and managing such a large review
Are unconventional or complex strategies explained?
N/a

2. BOOLEAN AND PROXIMITY OPERATORS

A ---No revisions	X
B --- Revision(s) suggested	☐
C --- Revision(s) required	☐

If "B" or "C," please provide an explanation or example:

Are Boolean or proximity operators used correctly?
Yes
Is the use of nesting with brackets appropriate and effective for the search?
Yes
If NOT is used, is this likely to result in any unintended exclusions?
Could precision be improved by using proximity operators (e.g. adjacent, near, within) or phrase searching instead of AND?
No
Is the width of proximity operators suitable (e.g. might adj5 pick up more variants than adj2)?
n/a

For peer review only - http://bmjopen.bmj.com/site/about/guidelines.xhtml

3. SUBJECT HEADINGS

A ---No revisions	X
B --- Revision(s) suggested	☐
C --- Revision(s) required	☐

If “B” or “C,” please provide an explanation or example:

Are the subject headings relevant?
Yes
Are any relevant subject headings missing (e.g. previous index terms)?
None obvious that I could see
Are any subject headings too broad or too narrow?
No
Are subject headings exploded where necessary and vice versa?
Yes
Are major headings (“starring” or restrict to focus) used? If so, if there adequate justification?
No
Are subheadings missing?
No
Are subheadings attached to subject headings? (Floating subheadings may be preferred)
n/a
Are floating subheadings relevant and used appropriately?
n/a
Are both subject headings and terms in free text used for each concept?
Yes

4. TEXT WORD SEARCHING

A ---No revisions	☐
B --- Revision(s)suggested	X
C --- Revision(s) required	☐

If “B” or “C,” please provide an explanation or example:

Does the search include all spelling variants in free text (e.g. UK versus US spelling)?
Add MCI for mild cognitive impairment
Look at searching the term digital health.
Look at cognitive stimulation therapy which can be used with people with dementia
Does the search include all synonyms or antonyms (e.g. opposites)?
See above
Does the search capture relevant truncation (e.g. is truncation at the correct place)?
You can add * to some words which may want to be searched with and without an s at the end for example husband and husbands could just be searched as husband*. But this would not effect the numbers you have already retrieved but may make the search easier to follow with less terms.
Are acronyms or abbreviations used appropriately? Do they capture irrelevant material? Are the full terms also included?
See above
Are the keywords specific enough or too broad? Are too many or too few keywords used? Are stop words used?
See above

For peer review only

Have the appropriate fields been searched (e.g. is the choice of text world fields (.tw.) or all fields (.af.) appropriate? Are there any other fields to be included or excluded (database-specific)?
Yes
Should any long strings be broken into several shorter search statements?
No

Author response:
Thank you for these suggestions. MCI has been added to the search strategy and more terms have been truncated using the *. Digital health and cognitive stimulation therapy are very relevant concepts, however, the were not added to the search strategy as they should be captured by the terms “digital” and “therapy”, respectively, in the existing search strategy.

5. SPELLING, SYNTAX, AND LINE NUMBERS

A ---No revisions	X
B --- Revision(s)suggested	☐
C --- Revision(s) required	☐

If “B” or “C,” please provide an explanation or example:

Are there any spelling errors
no
Are there errors in system syntax (e.g. the use of a truncation symbol from a different search interface)?
no
Are there incorrect line combinations or orphan lines (e.g. lines that are not referred to in the final summation that could indicate an error in an AND or OR statement)?
no

6. LIMITS AND FILTERS

A ---No revisions	X
B --- Revision(s) suggested	☐
C --- Revision(s) required	☐

If “B” or “C,” please provide an explanation or example:

Are all limits and filters used appropriately and are they relevant given the research question?
The year limit seems appropriate you may even be able to change this to 2009 as last 10 years is a long time in technology so anything older than 10 years is most likely to be irrelevant now.
Are all limits and filters used appropriately and are they relevant for the database?
Yes – although for non-english/sweedish language you could rapidly appraise the English abstract if available and if seems very relevant seek translation of the remainder of the article.
Are any potentially helpful limits or filters missing? Are the limits or filters too broad or too narrow? Can any limits or filters be added or removed?
no
Are sources cited for the filters used?
Don’t understand this question

For peer review only - http://bmjopen.bmj.com/site/about/guidelines.xhtml

OVERALL EVALUATION (Note: If one or more “revision required” is noted above, the response below must be “revisions required”.)

A ---No revisions	
B --- Revision(s) suggested	X
C --- Revision(s) required	

Additional comments:

For peer review only

Implementation of e-mental health interventions for informal caregivers of adults with chronic diseases: a protocol for a mixed methods systematic review with a qualitative comparative analysis

Supplementary File 3: Search Strategy

PubMed

#	
**1**	caregiver*[Title/Abstract] OR<br>care-giver*[Title/Abstract] OR<br>carer*[Title/Abstract] OR<br>family[Title/Abstract] OR<br>families[Title/Abstract] OR<br>spous*[Title/Abstract] OR<br>sibling*[Title/Abstract] OR<br>husband*[Title/Abstract] OR<br>wife[Title/Abstract] OR<br>wives[Title/Abstract] OR<br>partner[Title/Abstract] OR<br>partners[Title/Abstract] OR<br>parents[Title/Abstract] OR<br>parent[Title/Abstract] OR<br>friend[Title/Abstract] OR<br>friends[Title/Abstract] OR<br>relative[Title/Abstract] OR<br>relatives[Title/Abstract] OR<br>couple[Title/Abstract] OR<br>couples[Title/Abstract] OR<br>mother*[Title/Abstract] OR<br>father*[Title/Abstract] OR<br>support person*[Title/Abstract] OR<br>next of kin[Title/Abstract] OR<br>significant other*[Title/Abstract] OR<br>caregivers[MeSH terms] OR<br>family[MeSH terms] OR<br>spouses[MeSH terms] OR<br>siblings[MeSH terms] OR<br>parents[MeSH terms] OR<br>friends[MeSH terms] OR<br>mothers[MeSH terms] OR<br>fathers[MeSH terms]
**2**	cancer[Title/Abstract] OR<br>tumor[Title/Abstract] OR<br>tumors[Title/Abstract] OR<br>tumours[Title/Abstract] OR<br>tumour[Title/Abstract] OR<br>neoplasm*[Title/Abstract] OR<br>oncology[Title/Abstract] OR<br>carcinoma[Title/Abstract] OR<br>malignan*[Title/Abstract] OR

	melanoma[Title/Abstract] OR
	metastasis[Title/Abstract] OR
	lymphoma[Title/Abstract] OR
	leukemia[Title/Abstract] OR
	diabetes[Title/Abstract] OR
	COPD[Title/Abstract] OR
	chronic obstructive pulmonary disease[Title/Abstract] OR
	COAD[Title/Abstract] OR
	chronic obstructive airway disease[Title/Abstract] OR
	chronic obstructive lung disease[Title/Abstract] OR
	chronic airflow obstruction[Title/Abstract] OR
	stroke[Title/Abstract] OR
	cerebrovascular accident[Title/Abstract] OR
	CVA[Title/Abstract] OR
	acute cerebrovascular accident[Title/Abstract] OR
	brain vascular accident[Title/Abstract] OR
	apoplexy[Title/Abstract] OR
	heart disease[Title/Abstract] OR
	cardiovascular disease[Title/Abstract] OR
	CVD[Title/Abstract] OR
	ischemic heart disease[Title/Abstract] OR
	IHD[Title/Abstract] OR
	coronary artery disease[Title/Abstract] OR
	CAD[Title/Abstract] OR
	angina[Title/Abstract] OR
	myocardial infarction[Title/Abstract] OR
	MI[Title/Abstract] OR
	heart attack[Title/Abstract] OR
	cardiac event[Title/Abstract] OR
	cardiac disease[Title/Abstract] OR
	dementia[Title/Abstract] OR
	alzheimer*[Title/Abstract] OR
	FTD[Title/Abstract] OR
	Lewy body[Title/Abstract] OR
	Lewy bodies[Title/Abstract] OR
	neurocognitive disorder*[Title/Abstract] OR
	MCI[Title/Abstract] OR
	mild cognitive impairment[Title/Abstract] OR
	memory impair*[Title/Abstract] OR
	cognitive decline[Title/Abstract] OR
	chronic illness*[Title/Abstract] OR
	chronic disease*[Title/Abstract] OR
	chronic condition*[Title/Abstract] OR
	multi-morbidit*[Title/Abstract] OR
	multimorbidit*[Title/Abstract] OR
	long-term health condition*[Title/Abstract] OR
	neoplasms[MeSH terms] OR
	diabetes mellitus[MeSH terms] OR
	pulmonary disease, chronic obstructive[MeSH terms] OR
	stroke[MeSH terms] OR
	cardiovascular diseases[MeSH terms] OR

For peer review only

	dementia[MeSH terms] OR<br>cognition disorders[MeSH terms] OR<br>chronic disease[MeSH terms] OR<br>multimorbidity[MeSH terms]
**3**	eHealth[Title/Abstract] OR<br>e-health[Title/Abstract] OR<br>e-mental health[Title/Abstract] OR<br>emental health[Title/Abstract] OR<br>mhealth[Title/Abstract] OR<br>m-health[Title/Abstract] OR<br>smartphone[Title/Abstract] OR<br>cell phone[Title/Abstract] OR<br>cellular phone[Title/Abstract] OR<br>mobile phone[Title/Abstract] OR<br>app[Title/Abstract] OR<br>apps[Title/Abstract] OR<br>application[Title/Abstract] OR<br>applications[Title/Abstract] OR<br>iPad[Title/Abstract] OR<br>computer[Title/Abstract] OR<br>tablet[Title/Abstract] OR<br>technology[Title/Abstract] OR<br>technologies[Title/Abstract] OR<br>electronic communication[Title/Abstract] OR<br>email[Title/Abstract] OR<br>e-mail[Title/Abstract] OR<br>text messag*[Title/Abstract] OR<br>internet[Title/Abstract] OR<br>wireless[Title/Abstract] OR<br>online[Title/Abstract] OR<br>digital[Title/Abstract] OR<br>on-line[Title/Abstract] OR<br>virtual[Title/Abstract] OR<br>ICT[Title/Abstract] OR<br>web[Title/Abstract] OR<br>website[Title/Abstract] OR<br>multimedia[Title/Abstract] OR<br>e-learning[Title/Abstract] OR<br>online social network[Title/Abstract] OR<br>iCBT[Title/Abstract] OR<br>cCBT[Title/Abstract] OR<br>e-therap*[Title/Abstract] OR<br>etherap*[Title/Abstract] OR<br>cell phone[MeSH terms] OR<br>mobile application[MeSH terms] OR<br>computers[MeSH terms] OR<br>therapy, computer assisted[MeSH terms] OR<br>wireless technology[MeSH terms] OR<br>information technology[MeSH terms] OR<br>technology[MeSH terms] OR<br>electronic mail[MeSH terms] OR

For peer review only

	internet[MeSH terms] OR<br>online social networking[MeSH terms] OR<br>virtual reality[MeSH terms] OR<br>web browser[MeSH terms] OR<br>multimedia[MeSH terms]
**4**	mental health[Title/Abstract] OR<br>mood[Title/Abstract] OR<br>depress*[Title/Abstract] OR<br>affective disorder[Title/Abstract] OR<br>negative affect[Title/Abstract] OR<br>dysthymia[Title/Abstract] OR<br>dysphoria[Title/Abstract] OR<br>melancholic[Title/Abstract] OR<br>anxiety[Title/Abstract] OR<br>burden[Title/Abstract] OR<br>distress[Title/Abstract] OR<br>stress[Title/Abstract] OR<br>well being[Title/Abstract] OR<br>emotion[Title/Abstract] OR<br>emotional[Title/Abstract] OR<br>mood disorders[MeSH terms] OR<br>depression[MeSH terms] OR<br>anxiety[MeSH terms] OR<br>anxiety disorders[MeSH terms] OR<br>stress, psychological[MeSH terms] OR<br>emotions[MeSH terms] OR<br>mental health[MeSH terms]
**5**	mental health service*[Title/Abstract] OR<br>mental healthcare[Title/Abstract] OR<br>therapy[Title/Abstract] OR<br>therapies[Title/Abstract] OR<br>treatment*[Title/Abstract] OR<br>intervention*[Title/Abstract] OR<br>program*[Title/Abstract] OR<br>psychological[Title/Abstract] OR<br>psychologist[Title/Abstract] OR<br>psychoeducation[Title/Abstract] OR<br>psycho-education[Title/Abstract] OR<br>CBT[Title/Abstract] OR<br>health education[Title/Abstract] OR<br>problem solving[Title/Abstract] OR<br>PST[Title/Abstract] OR<br>ACT[Title/Abstract] OR<br>behaviour*[Title/Abstract] OR<br>therapist[Title/Abstract] OR<br>support[Title/Abstract] OR<br>mindful*[Title/Abstract] OR<br>psychotherapy[Title/Abstract] OR<br>relaxation[Title/Abstract] OR<br>meditation[Title/Abstract] OR<br>behavior*[Title/Abstract] OR

For peer review only

	counseling[Title/Abstract] OR<br>counselling[Title/Abstract] OR<br>cognitive reframing[Title/Abstract] OR<br>cognitive restructuring[Title/Abstract] OR<br>self-help[Title/Abstract] OR<br>self-management[Title/Abstract] OR<br>blended[Title/Abstract] OR<br>mental health services[MeSH terms] OR<br>health education[MeSH terms] OR<br>problem solving[MeSH terms] OR<br>psychotherapy[MeSH terms] OR<br>relaxation[MeSH terms] OR<br>self-management[MeSH terms] OR<br>self-help groups[MeSH terms]
**6**	"2007/01/01"[Date - Publication] : "3000"[Date - Publication]
**7**	#1 AND #2 AND #3 AND #4 AND #5 AND #6

CINAHL Plus with Full Text

	((TI (caregiver* OR care-giver* OR carer* OR family OR families OR spous* OR sibling* OR husband* OR wife OR wives OR partner OR partners OR parents OR parent OR friend OR friends OR relative OR relatives OR couple OR couples OR mother* OR father* OR support person* OR next of kin OR significant other*) OR AB (caregiver* OR care-giver* OR carer* OR family OR families OR spous* OR sibling* OR husband* OR wife OR wives OR partner OR partners OR parents OR parent OR friend OR friends OR relative OR relatives OR couple OR couples OR mother* OR father* OR support person* OR next of kin OR significant other*) OR MH (caregivers+ OR family+ OR spouses+ OR siblings+ OR parents+ OR friends+ OR mothers+ OR fathers+)) AND (TI (cancer OR tumor OR tumors OR tumours OR tumour OR neoplasm* OR oncology OR carcinoma OR malignan* OR melanoma OR metastasis OR lymphoma OR leukemia OR diabetes OR COPD OR chronic obstructive pulmonary disease OR COAD OR chronic obstructive airway disease OR chronic obstructive lung disease OR chronic airflow obstruction OR stroke OR cerebrovascular accident OR CVA OR acute cerebrovascular accident OR brain vascular accident OR apoplexy OR heart disease OR cardiovascular disease OR CVD OR ischemic heart disease OR IHD OR coronary artery disease OR CAD OR angina OR myocardial infarction OR MI OR heart attack OR cardiac event OR cardiac disease OR dementia OR alzheimer* OR FTD OR Lewy body OR Lewy bodies OR neurocognitive disorder* OR MCI OR mild cognitive impairment OR memory impair* OR cognitive decline OR chronic illness* OR chronic disease* OR chronic condition* OR multi-morbidit* OR multimorbidit* OR long-term health condition*) OR AB (cancer OR tumor OR tumors OR tumours OR tumour OR neoplasm* OR oncology OR carcinoma OR malignan* OR melanoma OR metastasis OR lymphoma OR leukemia OR diabetes OR COPD OR chronic obstructive pulmonary disease OR COAD OR chronic obstructive airway disease OR chronic obstructive lung disease OR chronic airflow obstruction OR stroke OR cerebrovascular accident OR CVA OR acute cerebrovascular accident OR brain vascular accident OR apoplexy OR heart disease OR cardiovascular disease OR CVD OR ischemic heart disease OR IHD OR coronary artery disease OR CAD OR angina OR myocardial infarction OR MI OR heart attack OR cardiac event OR cardiac disease OR dementia OR alzheimer* OR FTD OR Lewy body OR Lewy bodies OR neurocognitive disorder* OR MCI OR mild cognitive impairment OR memory impair* OR cognitive decline OR chronic illness* OR chronic disease* OR chronic condition* OR multi-morbidit* OR multimorbidit* OR long-term health condition*) OR MH (neoplasms+ OR diabetes mellitus+ OR pulmonary disease, chronic obstructive+ OR stroke+ OR

For peer review only

	cardiovascular diseases+ OR dementia+ OR Cognition disorders+ OR Chronic disease+ OR multimorbidity+)) AND (TI (eHealth OR e-health OR e-mental health OR emental health OR mhealth OR m-health OR smartphone OR cell phone OR cellular phone OR mobile phone OR app OR application OR applications OR apps OR iPad OR computer OR tablet OR technology OR technologies OR electronic communication OR email OR e-mail OR text messag* OR internet OR wireless OR online OR digital OR on-line OR virtual OR ICT OR web OR website OR multimedia OR e-learning OR online social network OR iCBT OR cCBT OR e-therap* OR etherap*) OR AB (eHealth OR e-health OR e-mental health OR emental health OR mhealth OR m-health OR smartphone OR cell phone OR cellular phone OR mobile phone OR app OR application OR applications OR apps OR iPad OR computer OR tablet OR technology OR technologies OR electronic communication OR email OR e-mail OR text messag* OR internet OR wireless OR online OR digital OR on-line OR virtual OR ICT OR web OR website OR multimedia OR e-learning OR online social network OR iCBT OR cCBT OR e-therap* OR etherap*) OR MH (cell phone+ OR mobile application+ OR computers+ OR therapy, computer assisted+ OR wireless technology+ OR information technology+ OR technology+ OR electronic mail+ OR internet+ OR online social networking+ OR virtual reality+ OR web browser+ OR multimedia+)) AND (TI (mental health OR mood OR depress* OR affective disorder OR negative affect OR dysthymia OR dysphoria OR melancholic OR anxiety OR burden OR distress OR stress OR well being OR emotion OR emotional) OR AB (mental health OR mood OR depress* OR affective disorder OR negative affect OR dysthymia OR dysphoria OR melancholic OR anxiety OR burden OR distress OR stress OR well being OR emotion OR emotional) OR MH (mood disorders+ OR depression+ OR anxiety+ OR anxiety disorders+ OR stress, psychological+ OR emotions+ OR mental health+)) AND (TI (mental health service* OR mental healthcare OR therapy OR therapies OR treatment* OR intervention* OR program* OR psychological OR psychologist OR psychoeducation OR psycho-education OR CBT OR health education OR problem solving OR PST OR ACT OR behaviour* OR therapist OR support OR mindful* OR psychotherapy OR relaxation OR meditation OR behavior* OR counseling OR counselling OR cognitive reframing OR cognitive restructuring OR self-help OR self-management OR blended) OR AB (mental health service* OR mental healthcare OR therapy OR therapies OR treatment* OR intervention* OR program* OR psychological OR psychologist OR psychoeducation OR psycho-education OR CBT OR health education OR problem solving OR PST OR ACT OR behaviour* OR therapist OR support OR mindful* OR psychotherapy OR relaxation OR meditation OR behavior* OR counseling OR counselling OR cognitive reframing OR cognitive restructuring OR self-help OR self-management OR blended) OR MH (mental health services+ OR health education+ OR problem solving+ OR psychotherapy+ OR relaxation+ OR self-management+ OR self-help groups+)))

The Cochrane Library

	(((caregiver*):ti,ab,kw OR (care-giver*):ti,ab,kw OR (carer*):ti,ab,kw OR (family):ti,ab,kw OR (families):ti,ab,kw OR (spous*):ti,ab,kw OR (sibling*):ti,ab,kw OR (husband*):ti,ab,kw OR (wife):ti,ab,kw OR (wives):ti,ab,kw OR (partner):ti,ab,kw OR (partners):ti,ab,kw OR (parents):ti,ab,kw OR (parent):ti,ab,kw OR (friend):ti,ab,kw OR (friends):ti,ab,kw OR (relatives):ti,ab,kw OR (relative):ti,ab,kw OR (couples):ti,ab,kw OR (couple):ti,ab,kw OR (mother*):ti,ab,kw OR (father*):ti,ab,kw OR (support NEXT person*):ti,ab,kw OR ("next of kin"):ti,ab,kw OR (significant NEXT other*):ti,ab,kw OR [mh Caregivers] OR [mh Family] OR [mh Spouses] OR [mh Siblings] OR [mh Parents] OR [mh Friends] OR [mh Mothers] OR [mh Fathers]) AND ((cancer):ti,ab,kw OR (tumor):ti,ab,kw OR (tumors):ti,ab,kw OR (tumours):ti,ab,kw OR (tumour):ti,ab,kw OR (neoplasm*):ti,ab,kw OR (oncology):ti,ab,kw OR (carcinoma):ti,ab,kw OR (malignan*):ti,ab,kw OR (melanoma):ti,ab,kw OR (metastasis):ti,ab,kw OR (lymphoma):ti,ab,kw

For peer review only

	OR (leukemia):ti,ab,kw OR (diabetes):ti,ab,kw OR (COPD):ti,ab,kw OR ("chronic obstructive pulmonary disease"):ti,ab,kw OR (COAD):ti,ab,kw OR ("chronic obstructive airway disease"):ti,ab,kw OR ("chronic obstructive lung disease"):ti,ab,kw OR ("chronic airflow obstruction"):ti,ab,kw OR (stroke):ti,ab,kw OR ("cerebrovascular accident"):ti,ab,kw OR (CVA):ti,ab,kw OR ("acute cerebrovascular accident"):ti,ab,kw OR ("brain vascular accident"):ti,ab,kw OR (apoplexy):ti,ab,kw OR ("heart disease"):ti,ab,kw OR ("cardiovascular disease"):ti,ab,kw OR (CVD):ti,ab,kw OR ("ischemic heart disease"):ti,ab,kw OR (IHD):ti,ab,kw OR ("coronary artery disease"):ti,ab,kw OR (CAD):ti,ab,kw OR (angina):ti,ab,kw OR ("myocardial infarction"):ti,ab,kw OR (MI):ti,ab,kw OR ("heart attack"):ti,ab,kw OR ("cardiac event"):ti,ab,kw OR ("cardiac disease"):ti,ab,kw OR (dementia):ti,ab,kw OR (Alzheimer*):ti,ab,kw OR (FTD):ti,ab,kw OR ("Lewy body"):ti,ab,kw OR ("Lewy bodies"):ti,ab,kw OR (neurocognitive NEXT disorder*):ti,ab,kw OR ("mild cognitive impairment"):ti,ab,kw OR (MCI):ti,ab,kw OR (memory NEXT impair*):ti,ab,kw OR ("cognitive decline"):ti,ab,kw OR (chronic NEXT illness*):ti,ab,kw OR (chronic NEXT disease*):ti,ab,kw OR (chronic NEXT condition*):ti,ab,kw OR (multi-morbidit*):ti,ab,kw OR (multimorbidit*):ti,ab,kw OR (long-term NEXT health NEXT condition*):ti,ab,kw OR [mh Neoplasms] OR [mh "Diabetes mellitus"] OR [mh "pulmonary disease, chronic obstructive"] OR [mh stroke] OR [mh "cardiovascular diseases"] OR [mh dementia] OR [mh "cognition disorders"] OR [mh "chronic disease"] OR [mh Multimorbidity]) AND ((eHealth):ti,ab,kw OR (e-health):ti,ab,kw OR ("e-mental health"):ti,ab,kw OR ("emental health"):ti,ab,kw OR (mhealth):ti,ab,kw OR (m-health):ti,ab,kw OR (smartphone):ti,ab,kw OR ("cell phone"):ti,ab,kw OR ("cellular phone"):ti,ab,kw OR ("mobile phone"):ti,ab,kw OR (app):ti,ab,kw OR (application):ti,ab,kw OR (applications):ti,ab,kw OR (apps):ti,ab,kw OR (iPad):ti,ab,kw OR (computer):ti,ab,kw OR (tablet):ti,ab,kw OR (technology):ti,ab,kw OR (technologies):ti,ab,kw OR ("electronic communication"):ti,ab,kw OR (email):ti,ab,kw OR (e-mail):ti,ab,kw OR (text NEXT messag*):ti,ab,kw OR (internet):ti,ab,kw OR (wireless):ti,ab,kw OR (online):ti,ab,kw OR (digital):ti,ab,kw OR (on-line):ti,ab,kw OR (virtual):ti,ab,kw OR (ICT):ti,ab,kw OR (web):ti,ab,kw OR (website):ti,ab,kw OR (multimedia):ti,ab,kw OR (e-learning):ti,ab,kw OR ("online social network"):ti,ab,kw OR (iCBT):ti,ab,kw OR (cCBT):ti,ab,kw OR (e-therap*):ti,ab,kw OR (etherap*):ti,ab,kw OR [mh "cell phone"] OR [mh "mobile application"] OR [mh computers] OR [mh "therapy, computer assisted"] OR [mh "wireless technology"] OR [mh "information technology"] OR [mh technology] OR [mh "electronic mail"] OR [mh internet] OR [mh "online social networking"] OR [mh "virtual reality"] OR [mh "web browser"] OR [mh multimedia]) AND (("mental health"):ti,ab,kw OR (mood):ti,ab,kw OR (depress*):ti,ab,kw OR ("affective disorder"):ti,ab,kw OR ("negative affect"):ti,ab,kw OR (dysthymia):ti,ab,kw OR (dysphoria):ti,ab,kw OR (melancholic):ti,ab,kw OR (anxiety):ti,ab,kw OR (burden):ti,ab,kw OR (distress):ti,ab,kw OR (stress):ti,ab,kw OR ("well being"):ti,ab,kw OR (emotion):ti,ab,kw OR (emotional):ti,ab,kw OR [mh "mood disorders"] OR [mh depression] OR [mh anxiety] OR [mh "anxiety disorders"] OR [mh "stress, psychological"] OR [mh emotions] OR [mh "mental health"]) AND ((mental NEXT health NEXT service*):ti,ab,kw OR ("mental healthcare"):ti,ab,kw OR (therapy):ti,ab,kw OR (therapies):ti,ab,kw OR (treatment*):ti,ab,kw OR (intervention*):ti,ab,kw OR (program*):ti,ab,kw OR (psychological):ti,ab,kw OR (psychologist):ti,ab,kw OR (psychoeducation):ti,ab,kw OR (psycho-education):ti,ab,kw OR (CBT):ti,ab,kw OR ("health education"):ti,ab,kw OR ("problem solving"):ti,ab,kw OR (PST):ti,ab,kw OR (ACT):ti,ab,kw OR (behaviour*):ti,ab,kw OR (therapist):ti,ab,kw OR (support):ti,ab,kw OR (mindful*):ti,ab,kw OR (psychotherapy):ti,ab,kw OR (relaxation):ti,ab,kw OR (meditation):ti,ab,kw OR (behavior*):ti,ab,kw OR (counseling):ti,ab,kw OR (counselling):ti,ab,kw OR ("cognitive reframing"):ti,ab,kw OR ("cognitive restructuring"):ti,ab,kw OR (self-help):ti,ab,kw OR (self-management):ti,ab,kw OR (blended):ti,ab,kw OR [mh "mental health services"] OR [mh "health education"] OR [mh "problem solving"] OR [mh psychotherapy] OR [mh relaxation] OR [mh self-management] OR [mh "self-help groups"]))

EMBASE

Note: The broad search terms used in the search strategy, resulted in the retrieval of a significant number of irrelevant publications in the EMBASE database. Therefore, the search strategy for EMBASE was modified to eliminate key broad terms and when appropriate, replace them with phrases.

#	Searches
1	(caregiver* or care-giver* or carer* or family or families or spous* or sibling* or husband* or wife or wives or partner or partners or parents or parent or friend or friends or relatives or couple or couples or mother* or father* or support person* or next of kin or significant other*).ab,ti.
2	exp caregiver/
3	exp family/
4	exp spouse/
5	exp sibling/
6	exp parent/
7	exp friend/
8	exp mother/
9	exp father/
10	1 or 2 or 3 or 4 or 5 or 6 or 7 or 8 or 9
11	(cancer or tumor or tumors or tumours or tumour or neoplasm* or oncology or carcinoma or malignan* or melanoma or metastasis or lymphoma or leukemia or diabetes or COPD or chronic obstructive pulmonary disease or COAD or chronic obstructive airway disease or chronic obstructive lung disease or chronic airflow obstruction or stroke or cerebrovascular accident or CVA or acute cerebrovascular accident or brain vascular accident or apoplexy or heart disease or cardiovascular disease or CVD or ischemic heart disease or IHD or coronary artery disease or CAD or angina or myocardial infarction or MI or heart attack or cardiac event or cardiac disease or dementia or alzheimer* or FTD or Lewy body or Lewy bodies or neurocognitive disorder* or MCI or mild cognitive impairment or memory impair* or cognitive decline or chronic illness* or chronic disease* or chronic condition* or multi-morbidit* or multimorbidit* or long-term health condition*).ab,ti.
12	exp neoplasm/
13	exp diabetes mellitus/
14	exp chronic obstructive lung disease/
15	exp cerebrovascular accident/
16	exp cardiovascular disease/
17	exp dementia/

18	exp chronic disease/
19	exp multiple chronic conditions/
20	(eHealth or e-health or e-mental health or emental health or mhealth or m-health or smartphone or cell phone or cellular phone or mobile phone or app or apps or iPad or computer or tablet or electronic communication or email or e-mail or text messag* or internet or wireless or online or digital or on-line or virtual or ICT or website or multimedia or e-learning or online social network or iCBT or cCBT or e-therap* or etherap* or web-based or (mobile adj2 technolog*) or (technolog* adj2 solution*) or (technology adj2 based)).ab,ti.
21	exp mobile phone/
22	exp mobile application/
23	exp computer/
24	exp computer assisted therapy/
25	exp wireless communication/
26	exp information technology/
27	exp technology/
28	exp e-mail/
29	exp Internet/
30	exp virtual reality/
31	exp web browser/
32	exp multimedia/
33	20 or 21 or 22 or 23 or 24 or 25 or 26 or 27 or 28 or 29 or 30 or 31 or 32
34	(mental health or mood or depress* or affective disorder or negative affect or dysthymia or dysphoria or melancholic or anxiety or burden or distress or stress or well being or emotion or emotional).ab,ti.
35	exp mood disorder/
36	exp depression/
37	exp anxiety/ or exp anxiety disorder/
38	exp mental stress/
39	exp mental health/
40	(mental health service* or mental healthcare or intervention* or program* or psychological or psychologist or psychoeducation or psycho-education or CBT or health education or problem solving or PST or ACT or behaviour* or therapist or support or mindful* or psychotherapy or relaxation or meditation or behavior* or counseling or counselling or cognitive reframing or cognitive restructuring or self-help or self-management or (group adj therapy) or (psycho* adj therapy) or (cognitive adj2 therapy) or (behavio* adj2 therapy) or (problem adj2 therapy) or (acceptance adj2 therapy) or blended).ab,ti.

41	exp mental health service/
42	exp health education/
43	exp problem solving/
44	exp psychotherapy/
45	exp self help/
46	34 or 35 or 36 or 37 or 38 or 39
47	40 or 41 or 42 or 43 or 44 or 45
48	11 or 12 or 13 or 14 or 15 or 16 or 17 or 18 or 19
49	10 and 33 and 46 and 47 and 48
50	limit 49 to yr="2007 -Current"

PsychINFO

#	Searches
1	(caregiver* or care-giver* or carer* or family or families or spous* or sibling* or husband* or wife or wives or partner or partners or parents or parent or friend or friends or relative or relatives or couple or couples or mother* or father* or support person* or next of kin or significant other*).ab,ti.
2	exp Caregivers/
3	exp Family/
4	exp Spouses/
5	exp Siblings/
6	exp Parents/
7	exp Mothers/
8	exp Fathers/
9	exp Friends/
10	(cancer or tumor or tumors or tumours or tumour or neoplasm* or oncology or carcinoma or malignan* or melanoma or metastasis or lymphoma or leukemia or diabetes or COPD or chronic obstructive pulmonary disease or COAD or chronic obstructive airway disease or chronic obstructive lung disease or chronic airflow obstruction or stroke or cerebrovascular accident or CVA or acute cerebrovascular accident or brain vascular accident or apoplexy or heart disease or cardiovascular disease or CVD or ischemic heart disease or IHD or coronary artery disease or CAD or angina or myocardial infarction or MI or heart attack or cardiac event or cardiac disease or dementia or alzheimer* or FTD or Lewy body or Lewy bodies or neurocognitive disorder* or MCI or mild cognitive impairment or memory impair* or cognitive

	decline or chronic illness* or chronic disease* or chronic condition* or multi-morbidit* or multimorbidit* or long-term health condition*).ab,ti.
11	exp Neoplasms/
12	exp Diabetes Mellitus/
13	exp Chronic Obstructive Pulmonary Disease/
14	exp Stroke/
15	exp Dementia/
16	exp Cognitive Impairment/
17	exp Chronic Illness/
18	exp Comorbidity/
19	exp Cardiovascular Disorders/
20	(eHealth or e-health or e-mental health or emental health or mhealth or m-health or smartphone or cell phone or cellular phone or mobile phone or app or application or applications or apps or iPad or computer or tablet or technology or technologies or electronic communication or email or e-mail or text messag* or internet or wireless or online or digital or on-line or virtual or ICT or web or website or multimedia or e-learning or online social network or iCBT or cCBT or e-therap* or etherap*).ab,ti.
21	exp Mobile Phones/
22	exp Mobile Applications/
23	exp Computers/
24	exp Computer Assisted Therapy/
25	exp Wireless Technologies/
26	exp "Information and Communication Technology"/
27	exp Technology/
28	exp Computer Mediated Communication/
29	exp Internet/
30	exp Online Social Networks/
31	exp Virtual Reality/
32	exp Websites/
33	exp Multimedia/
34	(mental health or mood or depress* or affective disorder or negative affect or dysthymia or dysphoria or melancholic or anxiety or burden or distress or stress or well being or emotion or emotional).ab,ti.
35	exp "Depression (Emotion)"/ or exp Major Depression/

36	exp Anxiety Disorders/ or exp Anxiety/
37	exp Physiological Stress/
38	exp Emotions/
39	exp Mental Health/
40	exp Mood Disorders/
41	(mental health service* or mental healthcare or therapy or therapies or treatment* or intervention* or program* or psychological or psychologist or psychoeducation or psycho-education or CBT or health education or problem solving or PST or ACT or behaviour* or therapist or support or mindful* or psychotherapy or relaxation or meditation or behavior* or counseling or counselling or cognitive reframing or cognitive restructuring or self-help or self-management or blended).ab,ti.
42	exp Mental Health Services/
43	exp Health Education/
44	exp Problem Solving/
45	exp Psychotherapy/
46	exp Relaxation/
47	exp Self-Management/
48	exp Support Groups/
49	1 or 2 or 3 or 4 or 5 or 6 or 7 or 8 or 9
50	10 or 11 or 12 or 13 or 14 or 15 or 16 or 17 or 18 or 19
51	20 or 21 or 22 or 23 or 24 or 25 or 26 or 27 or 28 or 29 or 30 or 31 or 32 or 33
52	34 or 35 or 36 or 37 or 38 or 39 or 40
53	41 or 42 or 43 or 44 or 45 or 46 or 47 or 48
54	49 and 50 and 51 and 52 and 53
55	limit 54 to yr="2007 -Current"

Web of Science

#	
**1**	TI=(caregiver* OR care-giver* OR carer* OR family OR families OR spous* OR sibling* OR husband* OR wife OR wives OR partner OR partners OR parents OR parent OR friend OR friends OR relative OR relatives OR couple OR couples OR mother* OR father* OR support person* OR next of kin OR significant other*)
**2**	AB=(caregiver* OR care-giver* OR carer* OR family OR families OR spous* OR sibling* OR husband* OR wife OR wives OR partner OR partners OR parents OR parent OR friend OR friends OR relative OR relatives OR couple OR couples OR mother* OR father* OR support person* OR next of kin OR significant other*)
**3**	**#1 OR #2**
**4**	TI=(cancer OR tumor OR tumors OR tumours OR tumour OR neoplasm* OR oncology OR carcinoma OR malignan* OR melanoma OR metastasis OR lymphoma OR leukemia OR diabetes OR COPD OR chronic obstructive pulmonary disease OR COAD OR chronic obstructive airway disease OR chronic obstructive lung disease OR chronic airflow obstruction OR stroke OR cerebrovascular accident OR CVA OR acute cerebrovascular accident OR brain vascular accident OR apoplexy OR heart disease OR cardiovascular disease OR CVD OR ischemic heart disease OR IHD OR coronary artery disease OR CAD OR angina OR myocardial infarction OR MI OR heart attack OR cardiac event OR cardiac disease OR dementia OR alzheimer* OR FTD OR Lewy body OR Lewy bodies OR neurocognitive disorder* OR MCI OR mild cognitive impairment OR memory impair* OR cognitive decline OR chronic illness* OR chronic disease* OR chronic condition* OR multi-morbidit* OR multimorbidit* OR long-term health condition*)
**5**	AB=(cancer OR tumor OR tumors OR tumours OR tumour OR neoplasm* OR oncology OR carcinoma OR malignan* OR melanoma OR metastasis OR lymphoma OR leukemia OR diabetes OR COPD OR chronic obstructive pulmonary disease OR COAD OR chronic obstructive airway disease OR chronic obstructive lung disease OR chronic airflow obstruction OR stroke OR cerebrovascular accident OR CVA OR acute cerebrovascular accident OR brain vascular accident OR apoplexy OR heart disease OR cardiovascular disease OR CVD OR ischemic heart disease OR IHD OR coronary artery disease OR CAD OR angina OR myocardial infarction OR MI OR heart attack OR cardiac event OR cardiac disease OR dementia OR alzheimer* OR FTD OR Lewy body OR Lewy bodies OR neurocognitive disorder* OR MCI OR mild cognitive impairment OR memory impair* OR cognitive decline OR chronic illness* OR chronic disease* OR chronic condition* OR multi-morbidit* OR multimorbidit* OR long-term health condition*)
**6**	**#4 OR #5**
**7**	TI=(eHealth OR e-health OR e-mental health OR emental health OR mhealth OR m-health OR smartphone OR cell phone OR cellular phone OR mobile phone OR app OR application OR applications OR apps OR iPad OR computer OR tablet OR technology OR technologies OR electronic communication OR email OR e-mail OR text messag* OR internet OR wireless OR online OR digital OR on-line OR virtual OR ICT OR web OR website OR multimedia OR e-learning OR online social network OR iCBT OR cCBT OR e-therap* OR etherap*)
**8**	AB=(eHealth OR e-health OR e-mental health OR emental health OR mhealth OR m-health OR smartphone OR cell phone OR cellular phone OR mobile phone OR app OR application OR applications OR apps OR iPad OR computer OR tablet OR technology OR technologies OR electronic communication OR email OR e-mail OR text messag* OR internet OR wireless OR online OR digital OR on-line OR virtual OR ICT OR web OR website OR multimedia OR e-learning OR online social network OR iCBT OR cCBT OR e-therap* OR etherap*)
**9**	**#7 OR #8**

For peer review only

**10**	TI=(mental health OR mood OR depress* OR affective disorder OR negative affect OR dysthymia OR dysphoria OR melancholic OR anxiety OR burden OR distress OR stress OR well being OR emotion OR emotional)
**11**	AB=(mental health OR mood OR depress* OR affective disorder OR negative affect OR dysthymia OR dysphoria OR melancholic OR anxiety OR burden OR distress OR stress OR well being OR emotion OR emotional)
**12**	**#10 OR #11**
**13**	TI=(mental health service* OR mental healthcare OR therapy OR therapies OR treatment* OR intervention* OR program* OR psychological OR psychologist OR psychoeducation OR psycho-education OR CBT OR health education OR problem solving OR PST OR ACT OR behaviour* OR therapist OR support OR mindful* OR psychotherapy OR relaxation OR meditation OR behavior* OR counseling OR counselling OR cognitive reframing OR cognitive restructuring OR self-help OR self-management OR blended)
**14**	AB=(mental health service* OR mental healthcare OR therapy OR therapies OR treatment* OR intervention* OR program* OR psychological OR psychologist OR psychoeducation OR psycho-education OR CBT OR health education OR problem solving OR PST OR ACT OR behaviour* OR therapist OR support OR mindful* OR psychotherapy OR relaxation OR meditation OR behavior* OR counseling OR counselling OR cognitive reframing OR cognitive restructuring OR self-help OR self-management OR blended)
**15**	**#13 OR #14**
**16**	**#3 AND #6 AND # 9 AND #12 AND #15**

For peer review only

Implementation of e-mental health interventions for informal caregivers of adults with chronic diseases: a protocol for a mixed methods systematic review with a qualitative comparative analysis

Supplementary File 4: Data Extraction Form

**Study Identification Features**		
Unique Study Identifier		
Title		
First Author		
Year of Publication		
Country of Study		
Funding Source		
Protocol Available?		
**Study Characteristics**		
Aims and Objectives		
Study Design (e.g. RCT, qualitative)		
Inclusion/Exclusion Criteria		
Recruitment Setting and Method		
Randomization	Sequence Generation	
	Type	
	Allocation Concealment	
Blinding	Data Collectors	
	Data Analysts	
**Caregiver Characteristics**		
Caregiver Sample Size (total and per arm)		
Age		
Gender		
Severity of Anxiety at Baseline		
Severity of Depression at Baseline		
Severity of Distress at Baseline		
Severity of Stress at Baseline		
Relationship to Care Recipient		
Amount of Care Provided		
Length of Time as a Caregiver		
Education Level		
Employment Status		
**Care Recipient Characteristics**		
Sample Size (total and per arm)		
Diagnosis		
Age		
Gender		
Severity Measure of Physical Health Condition of Care Recipient		
**Intervention**		

For peer review only

Summary of Intervention	
Summary of Control Condition	
Type of Therapy (e.g. psychoeducation, CBT)	
Format of Intervention (e.g. web, mobile app)	
Individual or Dyadic Intervention	
Other Support Provided (e.g. some face to face contact)	
Provider (e.g. lay worker, psychologist)	
Training provided to intervention provider (yes/no, describe if yes)	
Total Length of Treatment	
Number of Sessions	
Length of Sessions	
Adherence (did participants receive intended intervention, did they complete entire intervention)	
**Implementation Outcomes**	
Implementation Outcomes Measured or Described	
Measurement Tools Used	
Timing of Measurements	
**Mental Health Outcome Measurements**	
Mental Health Outcome Measurements (primary and secondary)	
Quality of Outcome Measurements	
Timing of Measurements	
**Statistical Techniques**	
Power Calculation	
Target Sample Size	
Method of Dealing with Missing Data	
Baseline Comparability	
**Participant Flow**	
Eligible Participants	
Randomised to Intervention	
Randomised to Control	
Lost to Follow-Up Intervention[1]	
Lost to Follow-Up Control[1]	
Analysed Intervention[2]	
Analysed Control[2]	
**Results Summary**	
Summary of Mental Health Outcome Results	
Summary of Implementation Results	
**Quantitative Results[3]**	

For peer review only

Intervention	Outcome Measurement	
	Measurement Scale Used	
	Analysis Method (ITT, per protocol)	
	Pre-Treatment Means	
	Pre-Treatment Standard Deviation	
	Pre-Treatment Number Analysed	
	Post-Treatment Means	
	Post-Treatment Standard Deviation	
	Post-Treatment Number Analysed	
Control	Outcome Measurement	
	Measurement Scale Used	
	Analysis Method (ITT, per protocol)	
	Pre-Treatment Means	
	Pre-Treatment Standard Deviation	
	Pre-Treatment Number Analysed	
	Post-Treatment Means	
	Post-Treatment Standard Deviation	
	Post-Treatment Number Analysed	
**Additional Comments**		

[1]Numbers lost to follow-up to be reported at each time point measured; [2]Numbers analysed to be reported for each time point measured; [3]Results to be extracted for outcomes of anxiety, depression, distress and/or stress at all follow-up time points

For peer review only

Implementation of e-mental health interventions for informal caregivers of adults with chronic diseases: a protocol for a mixed methods systematic review with a qualitative comparative analysis

Supplementary File 5: Sample data table

Study	Conditions[a]				Outcome	
	Lay-led	Mobile app	Supplemented with personal contact	Implementing organization involved in project	Effect size (raw Hedges' g)	Effective intervention[b]
Study A	0	0	1	1	0.25	0
Study B	1	0	1	0	0.5	1

[a]The conditions shown here are examples of conditions that could be explored in the qualitative comparative analysis. These are subject to change as the conditions selected depend on the features and heterogeneity among studies included in the review. The number 1 signifies that the study belongs to the indicated set. For example Study A was not lay led or a mobile app, but it did contain personal contact and the implementing organization was involved in the project.

[b]Assuming an effective intervention is defined as having a Hedges' g effect size ≥ 0.3. In this example Study A has an effect size below this cut-off, therefore, is considered not effective.

For peer review only

BMJ Open

Implementation of e-mental health interventions for informal caregivers of adults with chronic diseases: a protocol for a mixed methods systematic review with a qualitative comparative analysis

Journal:	*BMJ Open*
Manuscript ID	bmjopen-2019-035406.R2
Article Type:	Protocol
Date Submitted by the Author:	18-May-2020
Complete List of Authors:	Coumoundouros, Chelsea; Uppsala University, Clinical Psychology in Healthcare, Department of Women's and Children's Health<br>von Essen, Louise; Uppsala University, Clinical Psychology in Healthcare, Department of Women's and Children's Health<br>Sanderman, Robbert; University of Groningen, University Medical Center Groningen, Department of Health Psychology; University of Twente, Department of Psychology, Health and Technology<br>Woodford, Joanne; Uppsala University, Clinical Psychology in Healthcare, Department of Women's and Children's Health
**Primary Subject Heading**:	Mental health
Secondary Subject Heading:	Evidence based practice, Health services research
Keywords:	MENTAL HEALTH, World Wide Web technology < BIOTECHNOLOGY & BIOINFORMATICS, PRIMARY CARE, Depression & mood disorders < PSYCHIATRY, Anxiety disorders < PSYCHIATRY

SCHOLARONE™
Manuscripts

I, the Submitting Author has the right to grant and does grant on behalf of all authors of the Work (as defined in the below author licence), an exclusive licence and/or a non-exclusive licence for contributions from authors who are: i) UK Crown employees; ii) where BMJ has agreed a CC-BY licence shall apply, and/or iii) in accordance with the terms applicable for US Federal Government officers or employees acting as part of their official duties; on a worldwide, perpetual, irrevocable, royalty-free basis to BMJ Publishing Group Ltd ("BMJ") its licensees and where the relevant Journal is co-owned by BMJ to the co-owners of the Journal, to publish the Work in this journal and any other BMJ products and to exploit all rights, as set out in our licence.

The Submitting Author accepts and understands that any supply made under these terms is made by BMJ to the Submitting Author unless you are acting as an employee on behalf of your employer or a postgraduate student of an affiliated institution which is paying any applicable article publishing charge ("APC") for Open Access articles. Where the Submitting Author wishes to make the Work available on an Open Access basis (and intends to pay the relevant APC), the terms of reuse of such Open Access shall be governed by a Creative Commons licence – details of these licences and which Creative Commons licence will apply to this Work are set out in our licence referred to above.

Other than as permitted in any relevant BMJ Author's Self Archiving Policies, I confirm this Work has not been accepted for publication elsewhere, is not being considered for publication elsewhere and does not duplicate material already published. I confirm all authors consent to publication of this Work and authorise the granting of this licence.

For peer review only

Implementation of e-mental health interventions for informal caregivers of adults with chronic diseases: a protocol for a mixed methods systematic review with a qualitative comparative analysis

Chelsea COUMOUNDOUROS[1] (chelsea.coumoundouros@kbh.uu.se), Louise VON ESSEN[1] (louise-von.essen@kbh.uu.se), Robbert SANDERMAN[2, 3] (r.sanderman@umcg.nl), Joanne WOODFORD[1] (joanne.woodford@kbh.uu.se)

Corresponding author

Chelsea Coumoundouros, Clinical Psychology in Healthcare, Department of Women's and Children's Health, Uppsala University, 751 23 Uppsala, Sweden. E-mail: chelsea.coumoundouros@kbh.uu.se Telephone: 0046 729 999 211

Affiliations

[1]Clinical Psychology in Healthcare, Department of Women's and Children's Health, Uppsala University, 751 23 Uppsala, Sweden.

[2]Department of Health Psychology, University Medical Center Groningen, University of Groningen, 9713 AV, Groningen, the Netherlands.

[3]Department of Psychology, Health and Technology, University of Twente, Enschede, the Netherlands

Word count: 4123

For peer review only

ABSTRACT

Introduction. Informal caregivers provide the majority of care to individuals with chronic health conditions, benefiting the care recipient and reducing use of formal care services. However, providing informal care negatively impacts the mental health of many caregivers. E-mental health interventions have emerged as a way to provide accessible mental healthcare to caregivers. Much attention has been given to reviewing the effectiveness and efficacy of such interventions, however, factors related to implementation have received less consideration. Therefore, this mixed methods systematic review will aim to examine factors associated with the effectiveness and implementation of e-mental health interventions for caregivers.

Methods and analysis. Eligible studies published since January 1st, 2007 will be searched for in several electronic databases (CINAHL Plus with Full Text, the Cochrane Library, EMBASE, PsychINFO, PubMed, and Web of Science), clinical trial registries and OpenGrey, with all screening steps conducted by two independent reviewers. Studies will be included if they focus on the implementation or effectiveness of e-mental health interventions designed for informal adult caregivers of adults with cancer, heart disease, stroke, diabetes, dementia or chronic obstructive pulmonary disease. Pragmatic randomized controlled trials quantitatively reporting on caregiver anxiety, depression, psychological distress or stress will be used for a qualitative comparative analysis to identify combinations of conditions that result in effective interventions. Qualitative and quantitative data on implementation of e-mental health interventions for caregivers will be integrated in a thematic synthesis to identify barriers and facilitators to implementation. These results will inform future development and implementation planning of e-mental health interventions for caregivers.

Ethics and dissemination. Ethical approval is not required for this study as no primary data will be collected. Results will be disseminated in the form of a scientific publication and presentations at academic conferences and plain language summaries for various stakeholders.

PROSPERO registration number: CRD42020155727

For peer review only

STRENGTHS AND LIMITATIONS OF THIS STUDY

- The mixed method design of this review will ensure a wide variety of data on implementation is captured and interpretations account for both qualitative and quantitative research findings
- The peer-reviewed, comprehensive search strategy with all selection steps completed by two independent reviewers will ensure a thorough search of the literature and reduce bias in study selection
- High heterogeneity across studies in terms of implementation or intervention features is easily accommodated in a qualitative comparative analysis
- Crisp set qualitative comparative analysis produces concrete results, increasing the usability of findings for healthcare professionals and decision-makers
- However, crisp set qualitative comparative analysis dichotomizes all variables including the outcome, therefore, a more detailed understanding of the strength of the effect size may be lost in this process

INTRODUCTION

Informal caregivers (hereafter referred to as caregivers) are family members or friends who provide unpaid support and care to individuals with healthcare needs. Caregivers play a vital societal role in healthcare systems worldwide, providing up to 80% of care to individuals with long-term care needs.[1] Informal care provision can include emotional support, assistance with household tasks (e.g. cooking, cleaning), medical care, transportation, managing finances and advocacy on behalf of the care recipient.[2,3] Demand for caregivers is expected to increase in the future as the proportion of older adults in populations around the world increases and healthcare policies favour deinstitutionalization and outpatient care.[4,5] As societal dependence on informal care continues to grow, it is becoming increasingly important to implement programs and policies to support individuals who become caregivers.[2,6]

Caregivers can experience both positive and negative outcomes over the course of their time as a caregiver.[3,7,8] Caregiving can lead to an improved relationship between caregiver and care recipient, feelings of personal development and a sense of accomplishment related to obtaining skills and recognizing the impact of the care they provide.[8] However, caregivers may also experience negative outcomes related to the caregiving role, such as financial strain and poor physical and mental health.[3,7,9] Indeed, the rate of depression and anxiety among caregivers exceeds that of the general population.[10] The prevalence of depressive symptoms in cancer and stroke caregivers is often above 40% and the prevalence of anxiety ranges from 21% to over 40%.[11,12] Mental health problems can result in large personal and societal costs related to increased morbidity and reduced productivity.[13–15] Additionally, poor caregiver mental health negatively impacts distress levels in the care recipient[16,17] and the quality of care provided by the caregiver.[18–21]

There is a clear need to develop effective interventions and resources to prevent or reduce the mental health burden experienced by caregivers. However, caregivers have reported various barriers to accessing mental health services such as lack of knowledge regarding available

For peer review only

services, financial barriers, stigma and prioritization of the caregiving role over self-care.[22] E-health technologies have emerged as an accessible way to provide support and information to caregivers[23–25] and can be designed to achieve various goals such as, improving communication, teaching skills or reducing depression.[26,27] Numerous systematic reviews and meta-analyses have examined e-health interventions for caregivers of adults with chronic health conditions, showing the potential for e-health interventions to improve caregiver well-being.[23,25,28–40]

E-mental health interventions, that is, mental health interventions delivered via the internet or using mobile technologies,[27,41,42] represent a subset of e-health interventions. E-mental health solutions offer a means to improve mental health service access globally[43,44] by eliminating many barriers to mental health service access (e.g. transportation, stigma, time)[25,42,45] and are often more cost-effective than traditional therapies.[42,45] Meta-analyses show reductions in caregiver's depression and anxiety in response to e-mental health interventions.[37,39] However, as many reviews focus on intervention efficacy and effectiveness,[23,28–32,34,36–40,46] gaps remain in our understanding of factors related to the intervention and the implementation context that make e-mental health interventions effective among caregivers.

Wider literature suggests that the implementation of e-health programs in real-world settings often encounters many barriers,[24,33,47,48] preventing effective interventions from being made available to those who need them. Few reviews have examined factors related to implementation of e-health interventions for caregivers,[33,35] with no current reviews, to the best of our knowledge, focusing on implementation of e-mental health interventions for caregivers exclusively. Evaluating the implementation of an intervention is essential to gain insights into why interventions succeed or fail when put into practice. Factors influencing implementation can be related to the intervention itself, the participants, the implementation setting and wider societal factors (e.g. regional policies).[49] Trials with a more pragmatic design may be better suited to investigating factors potentially associated with implementation given real-world conditions are more closely reflected in pragmatic trials.[50,51] However, systematic reviews and meta-analyses do not often distinguish between pragmatic and explanatory (also referred to as efficacy) trials despite the different conditions (e.g. setting, recruitment methods, eligibility criteria, control of adherence to and delivery of the intervention) under which interventions are evaluated.[50,52] Identifying trials with a pragmatic design may be a valuable factor to consider when interpreting results of reviews to inform implementation.

The aim of this review is to examine factors related to the effectiveness and implementation of e-mental health interventions for caregivers of adults with chronic diseases. Two approaches will be used to investigate this. First, studies with more pragmatic designs will be used exclusively to determine which combinations of intervention or implementation characteristics are associated with effectiveness using a qualitative comparative analysis. Second, reports regarding the implementation of e-mental health interventions will be thematically synthesized to establish the common barriers and facilitators to e-mental health implementation. Findings from this review can be used to guide the development of effective e-mental health interventions to support caregivers and ensure the successful implementation of these interventions within real-world healthcare settings.

For peer review only

METHODS AND ANALYSIS

This mixed method review protocol has been registered with PROSPERO with registration number CRD42020155727 and any protocol amendments will be recorded in PROSPERO. The Preferred Reporting Items for Systematic Reviews and Meta-Analysis Protocols (PRISMA-P) checklist[53] (online supplementary appendix 1) and the Joanna Briggs methodology for mixed methods systematic reviews[54] were used to guide the development of this protocol.

Study eligibility criteria

The eligibility criteria used to inform study inclusion and exclusion are outlined using PICOS (population, interventions, comparators, outcomes and study design).[55,56]

Population

Unpaid adult caregivers (aged 18 years or older) of adults with either cancer, chronic obstructive pulmonary disease (COPD), dementia, diabetes, heart disease or stroke. Care recipient's chronic health conditions eligible for inclusion were selected as, globally, they are the largest sources of disability adjusted life years due to physical chronic diseases in adults[57] and often require informal care.[58] No restrictions will be placed on the frequency or amount of care provided for someone to be considered a caregiver. Studies exclusively focusing on caregivers with severe mental health conditions (e.g. psychosis or bipolar disorder) will be excluded, as the focus of this review is on e-mental health interventions targeting psychological health difficulties associated with the provision of informal care, for example anxiety or depression, as opposed to targeting severe mental health conditions. Studies with interventions that solely focus on caregivers providing care to non-community dwelling care recipients will be excluded, given caregivers of individuals who do not live in the community may spend less time providing informal care[59] and generally experience lower levels of depression.[60,61] Additionally, studies of interventions designed specifically for caregivers of individuals at the end-of-life (e.g. within a few months of death) will be excluded, as end-of-life caregiving is associated with additional needs and burdens, for example difficulties related to grief and bereavement.[62]

Interventions

Interventions will utilise internet technology, such as web-based platforms or mobile-based applications, to deliver a mental health intervention to caregivers.[27,41] E-mental health interventions can encompass many types of mental health support such as screening, prevention, treatment or service delivery.[41] This review will focus on interventions targeting the treatment of common caregiver psychological health difficulties (anxiety, depression, psychological distress or stress). This can include any type of mental health treatment, including psychoeducation. Psychoeducation is defined as the provision of information regarding common psychological health difficulties and can be delivered passively (e.g. an information website) or actively (e.g. an information website with therapist support, homework or exercises).[63] The majority of therapeutic materials within the e-mental health intervention must be internet based, however, this may be supplemented with additional forms of support (such as telephone contact, face-to-face support or video-conferencing). There are no restrictions on the amount of support

For peer review only

provided within the e-mental health intervention. Interventions delivered via telephone, CD-ROM or video (including Skype) alone will be excluded.

Comparators

As it is necessary to determine effect sizes for the qualitative comparative analysis,[64] only studies of pragmatic randomized controlled trials with non-active controls will be included in this analysis. Non-active controls include: no treatment, wait-list control, treatment as usual, non-specific treatment component control (e.g. control for attention) or education on the care recipient's condition.[65] Studies using psychoeducation or active controls (e.g. controls using specific treatment components or studies comparing two therapies) will be excluded.

For thematic synthesis of barriers and facilitators to implementation, studies of any design (e.g. randomized controlled trials, process evaluations, focus groups) will be included in the analysis, regardless of the presence or absence of a control.

Outcomes

For the qualitative comparative analysis, studies must report on caregiver mental health outcomes, specifically anxiety, depression, psychological distress or stress, measured using an instrument with at least acceptable reliability (Cronbach's alpha ≥ 0.7).[66] Reliability of outcome measures will be assessed based on the main validation paper of the relevant measurement instrument, as this review will likely include studies with different caregiver populations, ages, genders and languages, the combination of which may not have been validated. Examples of eligible measurement instruments include the Center for Epidemiologic Studies – Depression Scale,[67] the Hospital Anxiety and Depression Scale[68] or the Perceived Stress Scale.[69]

For the thematic synthesis, studies will report on barriers and/or facilitators to intervention implementation. This may include qualitative (e.g. interviews or focus groups) or quantitative (e.g. Normalization Measure Development questionnaire[70]) data. Barriers or facilitators can include factors related to any aspect of the Consolidated Framework for Implementation Research[49] or the implementation outcome framework developed by Proctor and colleagues.[71] The Consolidated Framework for Implementation Research consists of 5 domains related to implementation, namely (1) intervention characteristics (e.g. adaptability, complexity); (2) outer setting (e.g. external policies, patient needs and resources); (3) inner/implementation setting (e.g. culture within the organization, readiness for implementation); (4) characteristics of individuals (e.g. self-efficacy, individual stage of change); and (5) process (e.g. planning, engaging).[49] The implementation outcome framework broadly classifies measurable implementation outcomes which includes acceptability, adoption, feasibility, fidelity, reach, appropriateness, implementation cost and sustainability.[71]

Study designs

Studies included for the qualitative comparative analysis must be pragmatic randomized controlled trials (also referred to as effectiveness trials). Pragmatic trials will be identified using the validated PRagmatic Explanatory Continuum Indicator Summary 2 (PRECIS-2) tool.[50]

For peer review only

PRECIS-2 was developed with input from clinicians, researchers and policymakers to allow trialists to assess how pragmatic or explanatory their trial design is across nine domains: eligibility criteria, recruitment, setting, organization, flexibility (delivery), flexibility (adherence), follow-up, primary outcome and primary analysis.[50] Trials with a pragmatic design will be defined as any trial with a mean score of 3 or higher using the PRECIS-2 tool.[50] PRECIS-2 has been used with this cut-off score to categorize studies in another systematic review,[72] although to our knowledge it has not previously been used to exclude studies from a systematic review. Using a cut-off score of 3 should ensure generous inclusion of trials containing at least a mixture of pragmatic and explanatory design features.[50]

To assess barriers and facilitators to implementation, any study type with quantitative and/or qualitative data will be eligible for inclusion.

Search strategy

Comprehensive literature searches will be conducted in multiple electronic databases (CINAHL Plus with Full Text, the Cochrane Library, EMBASE, PsychINFO, PubMed and Web of Science). Clinical trial registries (www.clinicaltrials.gov and www.who.int/trialsearch/) will be searched for relevant completed clinical trials and the resulting publications will be found and screened for inclusion. Searches for grey literature will be performed using OpenGrey (http://www.opengrey.eu/), a database of grey literature in Europe such as research reports and conference papers.

The search strategy was developed in consultation with Agnes Kotka, a librarian at Uppsala University and was reviewed by Professor Mariët Hagedoorn and Truus van Ittersum (University Medical Centre Groningen, University of Groningen) and Dr. Nathan Davies (University College London) following the PRESS peer review guidelines[73] (online supplementary appendix 2). The search was constructed using terms related to (1) caregivers; (2) the chronic health conditions of interest (cancer, COPD, dementia, diabetes, heart disease and stroke); (3) e-health/information and communication technology; (4) mental health; and (5) psychological therapies (see online supplementary appendix 3). Included terms were informed by existing reviews focusing on the population and/or intervention of interest to this review.[9,33,39,74–79] Search terms were refined based on feedback from the peer-review process, resulting in the addition of more truncations to search terms, elimination of repetitive search terms that did not retrieve additional records and the addition of an abbreviation missed prior to the peer-review process. The search will include relevant Medical Subject Headings (MeSH) when possible and terms will be searched for in the title/abstract of publications. Included studies will be restricted to those published in English, Dutch, German or Swedish. Literature produced from January 2007 onwards will be eligible for inclusion. Technologies from work published prior to 2007 may be outdated and other reviews have shown that production of publications involving e-health began to rise from 2007 onwards.[33,35] Electronic searches will be re-run prior to reporting of results to ensure the search is as up-to-date as possible.

Upon final inclusion of any studies, their references, results from forward citation searches and from the first three pages of the “find similar” search function in PubMed will be used to check

For peer review only

for any additional studies of interest. Experts in the field will be contacted to identify further studies for inclusion.

Study selection

Results of database searches will be imported into EndNote for deduplication following the procedures outlined by Bramer et al.[80] Remaining records will be imported into the online screening software Rayyan.[81] Titles, abstracts and full-texts will be screened independently by two reviewers. Conflicts will be discussed and a third reviewer will be consulted if consensus cannot be reached. Study selection will be based on the criteria outlined by the PICOS, with reasons for study exclusion being recorded at the full-text screening stage. Full-texts will be checked against each sub-section of the PICOS, recording which sub-sections are or are not met by each study, with an overall reason for exclusion being reported in the PRISMA flow-diagram. This will facilitate detailed discussions regarding study exclusion when conflicts arise. If studies do not contain enough information to decide upon inclusion, the original authors will be contacted at most twice over a one month period to obtain information to determine study eligibility. If the original authors do not respond, the study will be excluded. Abstracts, theses, books, commentaries, editorials and letters to the editor will be excluded. Reviews and study protocols will also be excluded, however, the references of related reviews will be checked for additional studies of interest, published results of relevant study protocols will be obtained and if protocol results are unpublished, authors will be contacted to determine whether access to unpublished results is possible.

Records retrieved from searches of clinical trial registries and OpenGrey will be screened for eligibility by one reviewer. When relevant clinical trial registries are identified, any resulting publications will be retrieved and screened for inclusion, unless already captured by the electronic database searches. If results from relevant trial registries are unpublished, authors will be contacted to determine if they are able to share details of any available results. Authors of grey literature records that do not contain enough information to assess eligibility will also be contacted for additional study details.

Exclusion of studies on the basis of adopting a more explanatory, as opposed to pragmatic, trial design will be conducted as a final step during the full text screening process. This screening step will only be applied to trials eligible for the qualitative comparative analysis. Studies will be scored using the PRECIS-2 tool by two independent reviewers and studies with a mean score below 3 will be excluded.[50,72]

Assessment of methodological quality

Methodological quality of studies included in the qualitative comparative analysis will be evaluated using the Cochrane Risk of Bias 2.0 tool for randomized controlled trials[82,83]. This evaluation will facilitate the identification of selection, performance, measurement, attrition and reporting bias.[83] Authors will be contacted if more information is required to complete the quality assessment. Reporting bias will be explored by comparing outcomes measures described in study protocols to the outcome measures reported in the methods and results sections of the corresponding completed trial. In response to any identified inconsistencies, authors will be

For peer review only - http://bmjopen.bmj.com/site/about/guidelines.xhtml

contacted to determine potential causes of this. Study assessment will be conducted by two independent reviewers, followed by discussion of any discrepancies, consulting a third reviewer as needed. Studies will not be excluded based on methodological quality, however the results of the Cochrane Risk of Bias 2.0 evaluation will be reported descriptively.

Data extraction

Data from included full-texts will be extracted into Microsoft Excel (2016), using a data extraction form developed for this review based on the Centre for Reviews and Dissemination guidelines.[56] Extracted information will include data pertaining to study participants, study design, the intervention and relevant outcomes (full details in online supplementary appendix 4). Data used in the qualitative comparative analysis and thematic synthesis will be extracted independently by two reviewers, with resulting extractions compared for accuracy and completion. All other data will be extracted by one reviewer and verified by a second reviewer. If conflicts arise, the original publication will be referred to in order to resolve misunderstandings and a third reviewer will be consulted if necessary. Authors will be contacted at most twice to obtain additional data and/or clarification as needed. Qualitative results pertaining to implementation will be transferred into NVivo 10 software[84] for thematic synthesis.

Data synthesis

Data related to the characteristics of each included study, such as the sample (e.g. sample size, participant demographics) or intervention (e.g. duration, type of support provided, delivery mode) characteristics, will be reported in summary tables. Further data synthesis will involve two analysis methods. Pragmatic randomized controlled trials with quantitative mental health outcome data will be included in the qualitative comparative analysis. Publications of any study design reporting on implementation will be included in the thematic synthesis, taking an integrative approach to synthesize both qualitative and quantitative findings.

Qualitative comparative analysis

A crisp set qualitative comparative analysis will be conducted to determine sets of conditions that result in effective e-mental health interventions for caregivers.[64] Crisp set qualitative comparative analysis involves dichotomizing outcome data (e.g. effective or not effective) and conditions (e.g. present or absent) selected for inclusion in the analysis into distinct categories.[64] A crisp set analysis approach was selected over a fuzzy set analysis as the results will be more clearly interpretable and easier for decision-makers to use.[85]

The first step of a qualitative comparative analysis is to build a data table containing information regarding the effectiveness of each study and conditions related to the intervention and its implementation (see online supplementary appendix 5).[64] Conditions to include in the data table will be based on important factors related to intervention components (e.g. uses goal-setting, homework), intervention delivery methods (e.g. mobile app, computer), and implementation (e.g. acceptability, feasibility). By restricting this analysis to pragmatic trials, which are designed to more closely reflect real-world settings, implementation conditions are more likely to be reported. Conditions selected will be adjusted given the need to ensure adequate

For peer review only

heterogeneity is present.[64] Qualitative comparative analysis requires diversity among studies in terms of conditions present and intervention effectiveness in order to determine the combination of factors sufficient for interventions to be effective.[64] Therefore, adjustments to outcome classification and conditions selected for analysis will be needed after data collection is completed.

Intervention effectiveness will be measured as the standardized mean effect size between control and comparator groups' mental health outcomes, calculated using Hedges' g and the Comprehensive Meta-Analysis (version 3) software. Effect sizes will be calculated for all mental health outcomes of interest for this review (anxiety, depression, psychological distress and stress) and will be based on data collected immediately after intervention completion. If enough studies report subsequent post-intervention follow-ups, these effect sizes will be calculated to explore whether different factors contribute to sustained intervention success. Effect sizes will be used to create crisp sets to categorize studies as effective (Hedges' $g \geq 0.3$) or not effective.[86] If most interventions are effective (or not effective), a different classification system will be created to ensure adequate heterogeneity for analysis,[64] for example categorizing studies as highly effective (Hedges' $g \geq 0.5$) or not highly effective.[86] Proposed cut-offs were developed based on existing meta-analyses of e-mental health interventions.[87–90]

The main data table will use general effectiveness as the outcome measure, meaning the primary mental health outcome as identified in each study will be used to represent the effectiveness of that intervention. If studies include multiple outcomes of interest, but do not identify a primary outcome measure, the outcome most frequently measured in included studies will be used to evaluate intervention effectiveness. Secondary analyses may be conducted for anxiety, depression, psychological distress and stress separately, to explore whether different conditions are more important for different outcome measures. However, this is dependent upon identification of an adequate number of studies for each outcome of interest. After completion of the data table, truth tables will be constructed and the software fs/QCA (version 3.1b) will be used to determine the sufficient conditions for effective e-mental health interventions.[91,92]

Thematic synthesis

Data from studies addressing implementation of e-mental health interventions for caregivers will be thematically synthesized using a deductive coding approach, to identify barriers and facilitators experienced during implementation.[93,94] It will likely be necessary to integrate qualitative and quantitative data as many aspects of implementation such as acceptability, feasibility and usability, may be measured using quantitative tools.[95] First, qualitative data will be thematically analyzed using the Consolidated Framework for Implementation Research to guide coding.[49] This framework was selected a priori as it was developed by combining multiple implementation theories into a single, comprehensive theory covering all aspects related to implementation[49] and it has been used as a coding guide in other reviews on implementation.[33,96] Qualitative data will be coded based on the 39 pre-defined constructs within the Consolidated Framework for Implementation Research,[49] with the creation of additional codes if needed.

For peer review only - http://bmjopen.bmj.com/site/about/guidelines.xhtml

Quantitative data will be narratively summarized to facilitate subsequent integration of qualitative and quantitative findings. Creating narrative summaries will involve approaches such as textually describing study findings and grouping findings based on the constructs and domains of the Consolidated Framework for Implementation Research.[94] Initially, 10% of full-texts included in the thematic synthesis will be coded independently by two reviewers, followed by discussion of the coding process in consultation with a third reviewer. The remaining coding will be conducted by one reviewer with regular discussions with a second reviewer, involving a third reviewer as needed. Results of the initial coding of qualitative data and narrative summaries of quantitative data will be analyzed together to identify barriers and facilitators to implementation. Two reviewers will independently identify barriers and facilitators, followed by discussion involving a third reviewer as needed.[93] Through this discussion, more abstract, analytical themes will be developed that go beyond the initial codes and identified barriers and facilitators.[93] This process will be iterative, modifying barriers and facilitators after defining initial analytical themes, followed by further refinement of analytical themes until the analytical themes fully encompass all codes and identified barriers and facilitators.[93]

Patient and public involvement

There was no patient or public involvement in the development of this protocol. However, caregivers and healthcare professionals will be involved in the interpretation of data for the thematic synthesis.[97,98] Results of the thematic synthesis will be presented to a multidisciplinary group of stakeholders (e.g. caregivers, mental health service providers) to explore whether the identified themes resonate with their experiences or if they feel important aspects related to implementation have not been captured by the synthesis. Their perspectives will be incorporated into the interpretation of the results of the thematic synthesis.

DISCUSSION

Despite the importance of implementation planning and need for effective e-mental health interventions for caregivers in real world practice, there have been no reviews focusing on this area. Using pragmatic trials and implementation research, this review will identify both the key characteristics of effective interventions and barriers and facilitators to implementation. A qualitative comparative analysis will be employed to identify combinations of conditions resulting in effective e-mental health interventions for caregivers, a method which, to the best of our knowledge, has not yet been used in this field. The results of the qualitative comparative analysis can be used to improve the design of future e-mental health interventions by identifying intervention components and implementation factors important to intervention effectiveness in real-world settings.

Additionally, common barriers and facilitators to implementation of e-mental health interventions for caregivers identified in this review can be used to inform implementation planning for similar interventions designed to reduce the mental health burden experienced by caregivers. For example, results may highlight the importance of providing training to individuals delivering the intervention or involvement of management staff in implementation activities. Improving our understanding of factors associated with implementation will allow implementers to both account

For peer review only

for and avoid common implementation challenges, thereby potentially increasing subsequent uptake and effectiveness of e-mental health programs developed to support caregivers.

ETHICS AND DISSEMINATION

Ethical approval is not required for this review, as no raw primary level data will be collected. The results of this work will be disseminated in the form of a scientific publication in a peer-reviewed journal and as presentations at conferences. Plain language summaries will be prepared and provided to groups working with or supporting caregivers and healthcare organizations. Results will also be disseminated throughout the Marie Sklodowska-Curie Innovation Training Network, ENTWINE, which conducts research related to informal care and technological interventions to support caregivers.

AUTHORS' CONTRIBUTIONS

CC contributed to the design of the study and wrote the manuscript. JW, who acts as the reviews guarantor, conceived the study, contributed to the study design and critically revised the manuscript draft. LvE and RS critically revised the study design and manuscript. All authors approved of the final manuscript.

ACKNOWLEDGEMENTS

We would like to thank Agnes Kotka, librarian at Uppsala University, for assisting in development of the search strategy. We are also grateful to Professor Mariët Hagedoorn, Truus van Ittersum (University Medical Centre Groningen, University of Groningen) and Dr. Nathan Davies (University College London), for providing valuable feedback as the peer-reviewers of the search strategy.

FUNDING

This work was supported by the European Union's Horizon 2020 research and innovation program under the Marie-Sklodowska Curie grant agreement No 814072. Funders were not involved in the creation, development or publication of this protocol, nor will they be involved in the conduct, analysis or reporting of the resulting systematic review.

COMPETING INTERESTS

The authors declare they have no conflict of interests.

ABBREVIATIONS

CINAHL: Cumulative Index to Nursing and Allied Health Literature

COPD: Chronic Obstructive Pulmonary Disease

EMBASE: Excerpta Medica dataBASE

PICOS: Population, Interventions, Comparators, Outcomes and Study design

For peer review only

PRECIS-2: PRagmatic Explanatory Continuum Indicator Summary 2

PRISMA-P: Preferred Reporting Items for Systematic Reviews and Meta-Analysis Protocols

PROSPERO: International prospective register of systematic reviews

REFERENCES

1 Hoffmann F, Rodrigues R. *Informal carers: who takes care of them? Policy brief*. Vienna: : European Centre for Social Welfare Policy and Research 2010. https://www.euro.centre.org/publications/detail/387 (accessed 8 Jul 2019).

2 National Academies of Sciences Engineering and Medicine. Family caregiving roles and impacts. In: Committee on Family Caregiving for Older Adults, Board on Health Care Services, Health and Medicine Division, *et al.*, eds. *Families Caring for an Aging America*. Washington, DC: : The National Academies Press 2016. doi:10.17226/23606

3 Girgis A, Lambert S. Cost of informal caregiving in cancer care. *Cancer Forum* 2017;**41**:16–22.https://search.informit.com.au/documentSummary;dn=112312308779892;res=IELHEA (accessed 9 Jul 2019).

4 Spasova S, Baeten R, Coster S, *et al. Challenges in long-term care in Europe: A study of national policies*. Brussels: European Comission: : European Social Policy Network (ESPN) 2018.

5 Colombo F, Llena-Nozal A, Mercier J, *et al. Help wanted? Providing and paying for long-term care*. OECD Publishing 2011. doi:10.1787/9789264097759-en

6 Schulz R, Czaja SJ. Family caregiving: A vision for the future. *Am J Geriatr Psychiatry* 2018;**26**:358–63. doi:10.1016/J.JAGP.2017.06.023

7 Lambert SD, Levesque J V., Girgis A. The impact of cancer and chronic conditions on caregivers and family members. In: Koczwara B, ed. *Cancer and Chronic Conditions*. Singapore: : Springer 2016. 159–202. doi:10.1007/978-981-10-1844-2_6

8 Li Q, Loke AY. The positive aspects of caregiving for cancer patients: a critical review of the literature and directions for future research. *Psychooncology* 2013;**22**:2399–407. doi:10.1002/pon.3311

9 Coumoundouros C, Ould Brahim L, Lambert SD, *et al.* The direct and indirect financial costs of informal cancer care: A scoping review. *Health Soc Care Community* 2019;**27**:e622–36. doi:10.1111/hsc.12808

10 Pinquart M, Sörensen S. Differences between caregivers and noncaregivers in psychological health and physical health: a meta-analysis. *Psychol Aging* 2003;**18**:250–67.https://psycnet-apa-org.proxy3.library.mcgill.ca/fulltext/2003-05349-011.pdf (accessed 9 Jul 2019).

11 Loh AZ, Tan JS, Zhang MW, *et al.* The global prevalence of anxiety and depressive symptoms among caregivers of stroke survivors. *J Am Med Dir Assoc* 2017;**18**:111–6. doi:10.1016/j.jamda.2016.08.014

For peer review only

12 Geng H, Chuang D, Yang F, *et al.* Prevalence and determinants of depression in caregivers of cancer patients: A systematic review and meta-analysis. *Medicine (Baltimore)* 2018;**97**:e11863. doi:10.1097/MD.0000000000011863

13 Birnbaum HG, Kessler RC, Kelley D, *et al.* Employer burden of mild, moderate, and severe major depressive disorder: mental health services utilization and costs, and work performance. *Depress Anxiety* 2010;**27**:78–89. doi:10.1002/da.20580

14 Kessler RC. The costs of depression. *Psychiatr Clin North Am* 2012;**35**:1–14. doi:10.1016/j.psc.2011.11.005

15 Doran CM, Kinchin I. A review of the economic impact of mental illness. *Aust Heal Rev* 2019;**43**:43–8. doi:10.1071/AH16115

16 Hagedoorn M, Sanderman R, Bolks H, *et al.* Distress in couples coping with cancer: a meta-analysis and critical review of role and gender effects. *Psychol Bull* 2008;**134**:1–30.https://psycnet-apa-org.proxy3.library.mcgill.ca/fulltext/2007-19419-001.pdf (accessed 11 Jul 2019).

17 Hodges LJ, Humphris GM, Macfarlane G. A meta-analytic investigation of the relationship between the psychological distress of cancer patients and their carers. *Soc Sci Med* 2005;**60**:1–12. doi:10.1016/J.SOCSCIMED.2004.04.018

18 Smith GR, Williamson GM, Miller LS, *et al.* Depression and quality of informal care: a longitudinal investigation of caregiving stressors. *Psychol Aging* 2011;**26**:584–91. doi:10.1037/a0022263

19 Christie J, Smith GR, Williamson GM, *et al.* Quality of informal care is multidimensional. *Rehabil Psychol* 2009;**54**:173–81. doi:10.1037/a0015705

20 Cooney C, Howard R, Lawlor B. Abuse of vulnerable people with dementia by their carers: can we identify those most at risk? *Int J Geriatr Psychiatry* 2006;**21**:564–71. doi:10.1002/gps.1525

21 Beach SR, Schulz R, Williamson GM, *et al.* Risk Factors for Potentially Harmful Informal Caregiver Behavior. *J Am Geriatr Soc* 2005;**53**:255–61. doi:10.1111/j.1532-5415.2005.53111.x

22 Mosher CE, Given BA, Ostroff JS. Barriers to mental health service use among distressed family caregivers of lung cancer patients. *Eur J Cancer Care (Engl)* 2015;**24**:50–9. doi:10.1111/ecc.12203

23 Ploeg J, Ali MU, Markle-Reid M, *et al.* Caregiver-focused, web-based interventions: systematic review and meta-analysis (Part 2). *J Med Internet Res* 2018;**20**:e11247. doi:10.2196/11247

24 Christie HL, Martin JL, Connor J, *et al.* eHealth interventions to support caregivers of people with dementia may be proven effective, but are they implementation-ready? *Internet Interv* 2019;:100260. doi:10.1016/j.invent.2019.100260

25 Sin J, Henderson C, Spain D, *et al.* eHealth interventions for family carers of people with long term illness: A promising approach? *Clin Psychol Rev* 2018;**60**:109–25.

For peer review only

doi:10.1016/J.CPR.2018.01.008

26 Pagliari C, Sloan D, Gregor P, *et al.* What is eHealth (4): a scoping exercise to map the field. *J Med Internet Res* 2005;**7**:e9. doi:10.2196/jmir.7.1.e9

27 Eysenbach G. What is e-health? *J Med Internet Res* 2001;**3**:e20. doi:10.2196/jmir.3.2.e20

28 Aldehaim AY, Alotaibi FF, Uphold CR, *et al.* The impact of technology-based interventions on informal caregivers of stroke survivors: a systematic review. *Telemed J e-health* 2016;**22**:223–31. doi:10.1089/tmj.2015.0062

29 Tang WP, Chan CW, So WK, *et al.* Web-based interventions for caregivers of cancer patients: A review of literatures. *Asia-Pacific J Oncol Nurs* 2014;**1**:9–15. doi:10.4103/2347-5625.135811

30 Kaltenbaugh D, Klem M, Hu L, *et al.* Using web-based interventions to support caregivers of patients with cancer: a systematic review. *Oncol Nurs Forum* 2015;**42**:156–64. doi:10.1188/15.ONF.156-164

31 McKechnie V, Barker C, Stott J. Effectiveness of computer-mediated interventions for informal carers of people with dementia—a systematic review. *Int Psychogeriatrics* 2014;**26**:1619–37. doi:10.1017/S1041610214001045

32 Wasilewski MB, Stinson JN, Cameron JI. Web-based health interventions for family caregivers of elderly individuals: A scoping review. *Int J Med Inform* 2017;**103**:109–38. doi:10.1016/J.IJMEDINF.2017.04.009

33 Christie HL, Bartels SL, Boots LMM, *et al.* A systematic review on the implementation of eHealth interventions for informal caregivers of people with dementia. *Internet Interv* 2018;**13**:51–9. doi:10.1016/J.INVENT.2018.07.002

34 Guay C, Auger C, Demers L, *et al.* Components and outcomes of Internet-based interventions for caregivers of older adults: systematic review. *J Med Internet Res* 2017;**19**:e313. doi:10.2196/jmir.7896

35 Heynsbergh N, Heckel L, Botti M, *et al.* Feasibility, useability and acceptability of technology-based interventions for informal cancer carers: a systematic review. *BMC Cancer* 2018;**18**. doi:10.1186/S12885-018-4160-9

36 Hopwood J, Walker N, McDonagh L, *et al.* Internet-based interventions aimed at supporting family caregivers of people with dementia: systematic review. *J Med Internet Res* 2018;**20**:e216. doi:10.2196/jmir.9548

37 Sherifali D, Ali MU, Ploeg J, *et al.* Impact of Internet-based interventions on caregiver mental health: systematic review and meta-analysis. *J Med Internet Res* 2018;**20**:e10668. doi:10.2196/10668

38 Ploeg J, Markle-Reid M, Valaitis R, *et al.* Web-based interventions to improve mental health, general caregiving outcomes, and general health for informal caregivers of adults with chronic conditions living in the community: rapid evidence review. *J Med Internet Res* 2017;**19**:e263. doi:10.2196/jmir.7564

39 Zhao Y, Feng H, Hu M, *et al.* Web-based interventions to improve mental health in home

For peer review only

caregivers of people with dementia: meta-analysis. *J Med Internet Res* 2019;**21**:e13415. doi:10.2196/13415

40 Boots LMM, de Vugt ME, van Knippenberg RJM, *et al.* A systematic review of Internet-based supportive interventions for caregivers of patients with dementia. *Int J Geriatr Psychiatry* 2014;**29**:331–44. doi:10.1002/gps.4016

41 Riper H, Andersson G, Christensen H, *et al.* Theme issue on e-mental health: a growing field in internet research. *J Med Internet Res* 2010;**12**:e74. doi:10.2196/jmir.1713

42 Lal S. E-mental health: Promising advancements in policy, research, and practice. *Healthc Manag Forum* 2019;**32**:56–62. doi:10.1177/0840470418818583

43 Rebello TJ, Marques A, Gureje O, *et al.* Innovative strategies for closing the mental health treatment gap globally. *Curr Opin Psychiatry* 2014;**27**:308–14. doi:10.1097/YCO.0000000000000068

44 Naslund JA, Aschbrenner KA, Araya R, *et al.* Digital technology for treating and preventing mental disorders in low-income and middle-income countries: a narrative review of the literature. *The Lancet Psychiatry* 2017;**4**:486–500. doi:10.1016/S2215-0366(17)30096-2

45 Health Quality Ontario. Internet-delivered cognitive behavioural therapy for major depression and anxiety disorders: a health technology assessment. *Ont Health Technol Assess Ser* 2019;**19**:1–199.http://www.ncbi.nlm.nih.gov/pubmed/30873251 (accessed 11 Jul 2019).

46 Sin J, Norman I. Psychoeducational interventions for family members of people with schizophrenia: a mixed-method systematic review. *J Clin Psychiatry* 2013;**74**:e1145–62. doi:10.4088/JCP.12r08308

47 Folker AP, Mathiasen K, Lauridsen SM, *et al.* Implementing internet-delivered cognitive behavior therapy for common mental health disorders: A comparative case study of implementation challenges perceived by therapists and managers in five European internet services. *Internet Interv* 2018;**11**:60–70. doi:10.1016/J.INVENT.2018.02.001

48 Gitlin LN, Marx K, Stanley IH, *et al.* Translating evidence-based dementia caregiving interventions into practice: State-of-the-science and next steps. *Gerontologist* 2015;**55**:210–26. doi:10.1093/geront/gnu123

49 Damschroder LJ, Aron DC, Keith RE, *et al.* Fostering implementation of health services research findings into practice: a consolidated framework for advancing implementation science. *Implement Sci* 2009;**4**:50. doi:10.1186/1748-5908-4-50

50 Loudon K, Treweek S, Sullivan F, *et al.* The PRECIS-2 tool: designing trials that are fit for purpose. *BMJ* 2015;**350**:h2147. doi:10.1136/bmj.h2147

51 Rothwell PM. External validity of randomised controlled trials: "To whom do the results of this trial apply?" *Lancet* 2005;**365**:82–93. doi:10.1016/S0140-6736(04)17670-8

52 Aves T, Allan KS, Lawson D, *et al.* The role of pragmatism in explaining heterogeneity in meta-analyses of randomised trials: a protocol for a cross-sectional methodological review.

For peer review only

BMJ Open 2017;**7**:e017887. doi:10.1136/BMJOPEN-2017-017887

53 Shamseer L, Moher D, Clarke M, *et al.* Preferred reporting items for systematic review and meta-analysis protocols (PRISMA-P) 2015: elaboration and explanation. *BMJ* 2015;**350**:g7647. doi:10.1136/bmj.g7647

54 Lizarondo L, Stern C, Carrier J, *et al.* Chapter 8: Mixed methods systematic reviews. In: Aromataris E, Munn Z, eds. *Joanna Briggs Institute Reviewer's Manual*. The Joanna Briggs Institute 2017. https://wiki.joannabriggs.org/display/MANUAL/Chapter+8%3A+Mixed+methods+systematic+reviews (accessed 1 Jul 2019).

55 Costantino G, Montano N, Casazza G. When should we change our clinical practice based on the results of a clinical study? Searching for evidence: PICOS and PubMed. *Intern Emerg Med* 2015;**10**:525–7. doi:10.1007/s11739-015-1225-5

56 Centre for Reviews and Dissemination. *Systematic Reviews: CRD's guidance for undertaking reviews in health care*. 3rd ed. CRD, University of York 2009. www.york.ac.uk/inst/crd (accessed 10 Jul 2019).

57 Institute for Health Metrics and Evaluation. *GBD compare data visualization*. Seattle, WA: : IHME, University of Washington 2018. http://www.healthdata.org/data-visualization/gbd-compare (accessed 10 Jul 2019).

58 Sinha M. *Portrait of caregivers, 2012*. 2013. https://www150.statcan.gc.ca/n1/pub/89-652-x/89-652-x2013001-eng.htm (accessed 11 Jul 2019).

59 König HH, Leicht H, Brettschneider C, *et al.* The costs of dementia from the societal perspective: is care provided in the community really cheaper than nursing home care? *J Am Med Dir Assoc* 2014;**15**:117–26. doi:10.1016/j.jamda.2013.10.003

60 Bleijlevens MHC, Stolt M, Stephan A, *et al.* Changes in caregiver burden and health-related quality of life of informal caregivers of older people with Dementia: Evidence from the European RightTimePlaceCare prospective cohort study. *J Adv Nurs* 2015;**71**:1378–91. doi:10.1111/jan.12561

61 Gaugler JE, Mittelman MS, Hepburn K, *et al.* Clinically significant changes in burden and depression among dementia caregivers following nursing home admission. *BMC Med* 2010;**8**:85. doi:10.1186/1741-7015-8-85

62 Bernard-Dubenske LL, Wen K-Y, Gustafson DH, *et al.* Caregivers' differing needs across key experiences of the advanced cancer disease trajectory. *Palliat Support Care* 2008;**6**:265–72. doi:10.1017/S1478951508000400

63 Donker T, Griffiths KM, Cuijpers P, *et al.* Psychoeducation for depression, anxiety and psychological distress: A meta-analysis. *BMC Med* 2009;**7**:79. doi:10.1186/1741-7015-7-79

64 Thomas J, O'Mara-Eves A, Brunton G. Using qualitative comparative analysis (QCA) in systematic reviews of complex interventions: a worked example. *Syst Rev* 2014;**3**:67. doi:10.1186/2046-4053-3-67

For peer review only

65 Mohr DC, Spring B, Freedland KE, *et al.* The selection and design of control conditions for randomized controlled trials of psychological interventions. *Psychother Psychosom* 2009;**78**:275–84. doi:10.1159/000228248

66 Fitzpatrick R, Davey C, Buxton M, *et al.* Evaluating patient-based outcome measures for use in clinical trials: a review. *Health Technol Assess (Rockv)* 1998;**2**. doi:10.3310/hta2140

67 Radloff LS. The CES-D scale: a self-report depression scale for research in the general population. *Appl Psychol Meas* 1977;**1**:385–401. doi:10.1177/014662167700100306

68 Zigmond AS, Snaith RP. The hospital anxiety and depression scale. *Acta Psychiatr Scand* 1983;**67**:361–70. doi:10.1111/j.1600-0447.1983.tb09716.x

69 Cohen S, Kamarck T, Mermelstein R. A global measure of perceived stress. *J Health Soc Behav* 1983;**24**:385–96. doi:10.2307/2136404

70 Vis C, Ruwaard J, Finch T, *et al.* Toward an objective assessment of implementation processes for innovations in health care: psychometric evaluation of the Normalization Measure Development (NoMAD) questionnaire among mental health care professionals. *J Med Internet Res* 2019;**21**:e12376. doi:10.2196/12376

71 Proctor E, Silmere H, Raghavan R, *et al.* Outcomes for implementation research: conceptual distinctions, measurement challenges, and research agenda. *Adm Policy Ment Health* 2011;**38**:65–76. doi:10.1007/s10488-010-0319-7

72 Sajobi TT, Li G, Awosoga O, *et al.* A comparison of meta-analytic methods for synthesizing evidence from explanatory and pragmatic trials. *Syst Rev* 2018;**7**:19. doi:10.1186/s13643-017-0668-3

73 McGowan J, Sampson M, Salzwedel DM, *et al.* PRESS Peer Review of Electronic Search Strategies: 2015 guideline statement. *J Clin Epidemiol* 2016;**75**:40–6. doi:10.1016/J.JCLINEPI.2016.01.021

74 Lambert SD, Duncan LR, Kapellas S, *et al.* A descriptive systematic review of physical activity interventions for caregivers: effects on caregivers' and care recipients' psychosocial outcomes, physical activity levels, and physical health. *Ann Behav Med* 2016;**50**:907–19. doi:10.1007/s12160-016-9819-3

75 Woodford J, Farrand P, Richards D, *et al.* Psychological treatments for common mental health problems experienced by informal carers of adults with chronic physical health conditions (Protocol). *Syst Rev* 2013;**2**:9. doi:10.1186/2046-4053-2-9

76 Corry M, While A, Neenan K, *et al.* A systematic review of systematic reviews on interventions for caregivers of people with chronic conditions. *J Adv Nurs* 2015;**71**:718–34. doi:10.1111/jan.12523

77 Vis C, Mol M, Kleiboer A, *et al.* Improving implementation of eMental health for mood disorders in routine practice: systematic review of barriers and facilitating factors. *JMIR Ment Heal* 2018;**5**:e20. doi:10.2196/mental.9769

78 Wozney L, Mcgrath PJ, Gehring ND, *et al.* eMental healthcare technologies for anxiety and depression in childhood and adolescence: systematic review of studies reporting

For peer review only

implementation outcomes. *JMIR Ment Heal* 2018;**5**:e48. doi:10.2196/mental.9655

79 Farrand P, Matthews J, Dickens C, *et al.* Psychological interventions to improve psychological well-being in people with dementia or mild cognitive impairment: systematic review and meta-analysis protocol. *BMJ Open* 2016;**6**:e009713. doi:10.1136/bmjopen-2015-009713

80 Bramer WM, Giustini D, de Jonge GB, *et al.* De-duplication of database search results for systematic reviews in EndNote. *J Med Libr Assoc* 2016;**104**:240–3. doi:10.3163/1536-5050.104.3.014

81 Ouzzani M, Hammady H, Fedorowicz Z, *et al.* Rayyan—a web and mobile app for systematic reviews. *Syst Rev* 2016;**5**:210. doi:10.1186/s13643-016-0384-4

82 Higgins JP, Savovic J, Page M, *et al.*, editors. *Revised Cochrane risk-of-bias tool for randomized trials (RoB 2)*. 2019. https://drive.google.com/file/d/1xBzD1BeRrXH2DHg58fnTNKinTkX5SLEt/view (accessed 22 Jul 2019).

83 Higgins JP, Altman D, Gotzsche P, *et al.* The Cochrane Collaboration's tool for assessing risk of bias in randomised trials. *BMJ* 2011;**343**:d5928. doi:10.1136/bmj.d5928

84 QSR International Pty Ltd. NVivo qualitative data analysis software. 2014.

85 Hill LG, Cooper BR, Parker LA. Qualitative comparative analysis: a mixed-method tool for complex implementation questions. *J Prim Prev* 2019;**40**:69–87. doi:10.1007/s10935-019-00536-5

86 Sullivan GM, Feinn R. Using effect size-or why the p value Is not enough. *J Grad Med Educ* 2012;**4**:279–82. doi:10.4300/JGME-D-12-00156.1

87 Davies EB, Morriss R, Glazebrook C. Computer-delivered and web-based interventions to improve depression, anxiety, and psychological well-being of university students: a systematic review and meta-analysis. *J Med Internet Res* 2014;**16**:e130. doi:10.2196/jmir.3142

88 van Beugen S, Ferwerda M, Hoeve D, *et al.* Internet-based cognitive behavioral therapy for patients with chronic somatic conditions: a meta-analytic review. *J Med Internet Res* 2014;**16**:e88. doi:10.2196/jmir.2777

89 Lorenzo-Luaces L, Johns E, Keefe JR. The generalizability of randomized controlled trials of self-guided Internet-based cognitive behavioral therapy for depressive symptoms: systematic review and meta-regression analysis. *J Med Internet Res* 2018;**20**:e10113. doi:10.2196/10113

90 Linde K, Sigterman K, Kriston L, *et al.* Effectiveness of psychological treatments for depressive disorders in primary care: systematic review and meta-analysis. *Ann Fam Med* 2015;**13**:56–68. doi:10.1370/afm.1719

91 Ragin C, Davey S. *Fuzzy-Set/Qualitative Comparative Analysis 3.0*. Irvine, Californai: : Department of Sociology, University of California 2016. http://www.socsci.uci.edu/~cragin/fsQCA/software.shtml (accessed 12 Jul 2019).

For peer review only

92 Ragin CC. User's guide to fuzzy-set/qualitative comparative analysis. Irvine, California: 2017. www.fsqca.com. (accessed 12 Jul 2019).

93 Thomas J, Harden A. Methods for the thematic synthesis of qualitative research in systematic reviews. *BMC Med Res Methodol* 2008;**8**:45. doi:10.1186/1471-2288-8-45

94 Popay J, Roberts H, Sowden A, *et al.* Guidance on the conduct of narrative synthesis in systematic reviews. 2006. http://citeseerx.ist.psu.edu/viewdoc/download?doi=10.1.1.178.3100&rep=rep1&type=pdf (accessed 31 Jul 2019).

95 Lewis CC, Fischer S, Weiner BJ, *et al.* Outcomes for implementation science: an enhanced systematic review of instruments using evidence-based rating criteria. *Implement Sci* 2015;**10**:155. doi:10.1186/s13012-015-0342-x

96 Kerins C, McSharry J, Hayes C, *et al.* Barriers and facilitators to implementation of menu labelling interventions to support healthy food choices: a mixed methods systematic review protocol. *Syst Rev* 2018;**7**:88. doi:10.1186/s13643-018-0752-3

97 Pollock A, Campbell P, Struthers C, *et al.* Stakeholder involvement in systematic reviews: A scoping review. *Syst Rev* 2018;**7**:208. doi:10.1186/s13643-018-0852-0

98 Boote J, Baird W, Sutton A. Public involvement in the systematic review process in health and social care: A narrative review of case examples. *Health Policy (New York)* 2011;**102**:105–16. doi:10.1016/J.HEALTHPOL.2011.05.002

For peer review only

Implementation of e-mental health interventions for informal caregivers of adults with chronic diseases: a protocol for a mixed methods systematic review with a qualitative comparative analysis

PRISMA-P (Preferred Reporting Items for Systematic review and Meta-Analysis Protocols) 2015 checklist: recommended items to address in a systematic review protocol*

Section and topic	Item No	Checklist item	Location in text (page number)
**ADMINISTRATIVE INFORMATION**			
Title:			
Identification	1a	Identify the report as a protocol of a systematic review	1
Update	1b	If the protocol is for an update of a previous systematic review, identify as such	N/A
Registration	2	If registered, provide the name of the registry (such as PROSPERO) and registration number	2 & 5
Authors:			
Contact	3a	Provide name, institutional affiliation, e-mail address of all protocol authors; provide physical mailing address of corresponding author	1
Contributions	3b	Describe contributions of protocol authors and identify the guarantor of the review	12
Amendments	4	If the protocol represents an amendment of a previously completed or published protocol, identify as such and list changes; otherwise, state plan for documenting important protocol amendments	5
Support:			
Sources	5a	Indicate sources of financial or other support for the review	12
Sponsor	5b	Provide name for the review funder and/or sponsor	12
Role of sponsor or funder	5c	Describe roles of funder(s), sponsor(s), and/or institution(s), if any, in developing the protocol	12
**INTRODUCTION**			
Rationale	6	Describe the rationale for the review in the context of what is already known	3-4
Objectives	7	Provide an explicit statement of the question(s) the review will address with reference to participants, interventions, comparators, and outcomes (PICO)	4
**METHODS**			
Eligibility criteria	8	Specify the study characteristics (such as PICO, study design, setting, time frame) and report characteristics (such as years considered, language, publication status) to be used as criteria for eligibility for the review	5-7

For peer review only

Information sources	9	Describe all intended information sources (such as electronic databases, contact with study authors, trial registers or other grey literature sources) with planned dates of coverage	7
Search strategy	10	Present draft of search strategy to be used for at least one electronic database, including planned limits, such that it could be repeated	7 & supplementary appendix 3
Study records:			
Data management	11a	Describe the mechanism(s) that will be used to manage records and data throughout the review	8
Selection process	11b	State the process that will be used for selecting studies (such as two independent reviewers) through each phase of the review (that is, screening, eligibility and inclusion in meta-analysis)	8
Data collection process	11c	Describe planned method of extracting data from reports (such as piloting forms, done independently, in duplicate), any processes for obtaining and confirming data from investigators	9
Data items	12	List and define all variables for which data will be sought (such as PICO items, funding sources), any pre-planned data assumptions and simplifications	9 & supplementary appendix 4
Outcomes and prioritization	13	List and define all outcomes for which data will be sought, including prioritization of main and additional outcomes, with rationale	6
Risk of bias in individual studies	14	Describe anticipated methods for assessing risk of bias of individual studies, including whether this will be done at the outcome or study level, or both; state how this information will be used in data synthesis	8-9
Data synthesis	15a	Describe criteria under which study data will be quantitatively synthesised	6-7 & 9
	15b	If data are appropriate for quantitative synthesis, describe planned summary measures, methods of handling data and methods of combining data from studies, including any planned exploration of consistency (such as I^2, Kendall's τ)	9-10
	15c	Describe any proposed additional analyses (such as sensitivity or subgroup analyses, meta-regression)	9-10
	15d	If quantitative synthesis is not appropriate, describe the type of summary planned	10-11
Meta-bias(es)	16	Specify any planned assessment of meta-bias(es) (such as publication bias across studies, selective reporting within studies)	8-9
Confidence in cumulative evidence	17	Describe how the strength of the body of evidence will be assessed (such as GRADE)	N/A

N/A not applicable

*** It is strongly recommended that this checklist be read in conjunction with the PRISMA-P Explanation and Elaboration (cite when available) for important clarification on the items. Amendments to a review protocol should be tracked and dated. The copyright for PRISMA-P (including checklist) is held by the PRISMA-P Group and is distributed under a Creative Commons Attribution Licence 4.0.**

From: Shamseer L, Moher D, Clarke M, Ghersi D, Liberati A, Petticrew M, Shekelle P, Stewart L, PRISMA-P Group. Preferred reporting items for systematic review and meta-analysis protocols (PRISMA-P) 2015: elaboration and explanation. BMJ. 2015 Jan 2;349(jan02 1):g7647.

For peer review only

PRESS Guideline — Search Submission & Peer Review Assessment

SEARCH SUBMISSION: THIS SECTION TO BE FILLED IN BY THE SEARCHER

Searcher: Chelsea Coumoundouros	Email: chelsea.coumoundouros@kbh.uu.se
Date Submitted: 2019/09/18	Date requested by: 2019/10/09

Systematic Review Title:

Implementation of e-Mental Health interventions for informal caregivers of adults with chronic diseases: a protocol for a mixed methods systematic review with a qualitative comparative analysis

This search strategy is …

X	My PRIMARY (core) database strategy — First time submitting a strategy for search question and database
	My PRIMARY (core) strategy — Follow-up review NOT the first time submitting a strategy for search question and database. If this is a response to peer review, itemize the changes made to the review suggestions
	SECONDARY search strategy— First time submitting a strategy for search question and database
	SECONDARY search strategy — NOT the first time submitting a strategy for search question and database. If this is a response to peer review, itemize the changes made to the review suggestions

Database

(i.e., MEDLINE,CINAHL…): *[mandatory]*

MEDLINE

Interface

(i.e., Ovid, EBSCO…): *[mandatory]*

PubMed

Research Question

(Describe the purpose of the search) *[mandatory]*

The aim of this review is to determine factors related to the successful implementation of e-mental health interventions for caregivers of adults with chronic diseases. Two approaches will be used to investigate this. First, studies with more pragmatic designs will be used exclusively to determine which combinations of intervention or implementation characteristics are associated with effective interventions using a qualitative comparative analysis. Second, reports regarding the implementation of e-mental health interventions will be thematically synthesized to establish the common barriers and facilitators to e-mental health implementation.

For peer review only - http://bmjopen.bmj.com/site/about/guidelines.xhtml

PICO Format

(Outline the PICOs for your question — i.e., Patient, Intervention, Comparison, Outcome, and Study Design — as applicable)

**P**	Unpaid adult caregivers of adults with either heart disease, stroke, cancer, diabetes, dementia or chronic obstructive pulmonary disease.
**I**	Any e-mental health intervention targeting the treatment of common mental health difficulties (e.g. depression, anxiety) in caregivers
**C**	**For the qualitative comparative analysis:** Studies must use a non-active control (no treatment, wait-list control, treatment as usual, non-specific treatment component control or education on the care recipient's condition). **For the thematic synthesis:** No restriction based on presence/absence of control group or control type.
**O**	**For the qualitative comparative analysis:** Depression, anxiety, stress or distress measured using an instrument with at least acceptable reliability (Cronbach's alpha ≥ 0.7). **For the thematic synthesis:** Barriers and/or facilitators to implementation.
**S**	**For the qualitative comparative analysis:** Studies must be randomized controlled effectiveness studies. **For the thematic synthesis:** No restriction based on study design.

Inclusion Criteria *[optional]*

P: Caregivers and care recipients must be aged 18 years or older. Chronic health conditions eligible for inclusion are listed in the PICOS above.

I: Interventions must be primarily delivered using Internet technology and target the caregiver's mental health. Interventions may be supplemented by additional forms of support (e.g. telephone call, face-to-face session). Any type of therapy will be included.

O: For the thematic synthesis: Barriers and facilitators can include factors related to any aspect of the Consolidated Framework for Implementation Research or the implementation outcome framework developed by Proctor and colleagues.

S: For the qualitative comparative analysis: Effectiveness will be defined as any study with a mean score of 3 or more using the PRECIS-2 tool.

For peer review only - http://bmjopen.bmj.com/site/about/guidelines.xhtml

Exclusion Criteria *[optional]*

P: Studies that focus on caregivers with severe mental health conditions, caregivers providing care to non-community dwelling care recipients or care recipients at the palliative phase of disease will be excluded.

I: Interventions delivered using the telephone, CD-ROM or video (including Skype) alone will be excluded.

C: For the qualitative comparative analysis: Studies using psychoeducation, education on mental health or active controls will be excluded.

Date limit: Studies published prior to 2007 will be excluded. Technologies from work published prior to 2007 may be outdated and other reviews have shown that production of publications involving eHealth began to rise from 2007 onwards

Language restriction: Studies written in languages other than English or Swedish will be excluded.

Was a search filter applied?

Yes ■ No ☐

If YES, which one(s) (e.g., Cochrane RCT filter, PubMed Clinical Queries filter)? Provide the source if this is a published filter. *[mandatory if YES to previous question — textbox]*

Date limit was applied as part of the search.

Other notes or comments you feel would be useful for the peer reviewer? ***[optional]***

There are no terms related to implementation as (1) this is not the only area we are interested in (also need effectiveness studies) and (2) implementation can be referred to in many different ways and is sometimes not mentioned at all in the title or abstract of studies, so trying to create terms about this topic may lead to poor retrieval of relevant work

Please copy and paste your search strategy here, exactly as run, including the number of hits per line. ***[mandatory]***

For peer review only - http://bmjopen.bmj.com/site/about/guidelines.xhtml

**Database: PubMed. Final number of results from the full search 3265<br>Date Restriction: January 1, 2007 to present**				
**1: Caregivers**	**2: Chronic Health Conditions**	**3: eHealth/ Technology**	**4: Mental Health**	**5: Therapy**
**Title/Abstract**	**Title/Abstract**	**Title/Abstract**	**Title/Abstract**	**Title/Abstract**
Caregiver 19465	Cancer 1019721	eHealth 2990	Mental health 92203	Mental health service 3222
Caregivers 35586	Tumor 615354	e-health 2140	Mood 45028	Mental health services 10159
Care-giver 301	Tumors 279910	e-mental health 213	Mood disorder 3595	Mental healthcare 1741
Care-givers 1034	Tumours 49415	emental health 30	Depression 185606	Therapy 923016
Carer 3436	Tumour 95450	mhealth 3596	Depressive 72042	Therapies 201143
Carers 8055	Neoplasm 34474	m-health 462	Depressed 30180	Treatment 2180941
Informal carer 94	Neoplasms 51500	Smartphone 7429	Affective disorder 2287	Treatments 283397
Informal carers 575	Oncology 65177	Cell phone 1702	Affect 366439	Interventions 307575
Informal caregiver 460	Carcinoma 275810	Cellular phone 362	Negative affect 7192	Intervention 397118
Informal caregivers 1917	Malignan* 271316	Mobile phone 5430	Dysthymia 886	Program 229608
Family 412900	Melanoma 56126	Mobile app 1046	Dysphoria 1871	Programs 158202
Families 124835	Metastasis 147467	Mobile apps 991	Melancholic 594	Programmes 36810
Spouse 4404	Lymphoma 76891	Mobile application 1252	Anxiety 117199	Programme 52551
Spousal 2026	Leukemia 86466	Mobile applications 1283	Burden 141973	Psychological 117677
Spouses 4578	Diabetes 309446	App 16442	Distress 63488	Psychologist 2715
Sibling 10277	Diabetes mellitus 108204	Apps 4615	Stress 465139	Psychoeducation 2202
Siblings 15168	Type 1 diabetes 27354	Application 442621	Well being 49008	Psycho-education 518
Husband 2380	Type 2 diabetes 95548	Applications 318200	Emotion 29552	CBT 8475
Husbands 2371	COPD 32037	iPad 1114	Emotional 95270	Cognitive behavioural therapy 3103
Wife 1919	Chronic obstructive pulmonary	Computer 94973		Cognitive therapy 1960
Wives 1829	disease 32548	Tablet 14085	**MeSH**	Health education 13665
Partner 47483	COAD 147	Computer based 6611	Mood disorders 55994	Problem solving 9444
Partners 51514	Chronic obstructive airway disease	Computer assistive	Depression 108610	Problem solving therapy 335
Parents 92138	80	technology 421	Anxiety 42892	PST 1976
Parent 69723	Chronic obstructive lung disease	Technology 205306	Anxiety disorders 28551	ACT 140661
Friend 6741	1842	Technologies 99160	Stress, psychological 66704	Commitment therapy 809
Friends 16333	Chronic airflow obstruction 102	Electronic communication	Emotions 119893	Behaviour 108763
Relatives 28499	Stroke 150135	757	Mental health 21517	Behavioural 45351
Relative 436237	Cerebrovascular accident 2137	Email 4949		Therapist 9305
Couple 18462	CVA 1540	E-mail 4654		Support 563829
Couples 20787	Cerebral stroke 676	Text messaging 1952		Mindful 2490
Mother 51594	Acute stroke 9906	Text message 1338		Mindfulness 6250
Mothers 64116	Acute cerebrovascular accident 58	Internet 36172		Dialectic behaviour therapy 5
Fathers 9825	Cerebrovascular stroke 133	Internet based 6623		Psychotherapy 13408
Father 10914	Brain vascular accident 2	Wireless 10928		Relaxation 53119
Support person 216	Cerebrovascular apoplexy 2	Digital 71968		Meditation 3440

For peer review only

For peer review only - http://bmjopen.bmj.com/site/about/guidelines.xhtml

Support persons 226<br>Next of kin 770<br>Significant other 583<br>Significant others 1502<br><br>**MeSH**<br>Caregivers 23052<br>Family 134295<br>Spouses 5686<br>Siblings 7235<br>Parents 57525<br>Friends 3754<br>Mothers 23221<br>Fathers 4249	Apoplexy 938<br>Heart disease 71238<br>Cardiovascular disease 89039<br>CVD 27460<br>Ischemic heart disease 10129<br>IHD 2703<br>Coronary artery disease 45519<br>CAD 25678<br>Angina 16189<br>Myocardial infarction 85190<br>MI 28920<br>Heart attack 2262<br>Cardiac event 2336<br>Cardiac disease 7437<br>Dementia 64525<br>Alzheimer* 90032<br>Alzheimer's disease 73741<br>Vascular dementia 3528<br>Frontotemporal dementia 4920<br>FTD 2656<br>Lewy bodies 4361<br>Lewy body 2324<br>Neurocognitive disorder 739<br>Neurocognitive disorders 1551<br>Mild cognitive impairment 13740<br>Memory impair* 8743<br>Cognitive decline 16542<br>Chronic illness 7067<br>Chronic illnesses 2903<br>Chronic diseases 23041<br>Chronic disease 23460<br>Chronic condition 3482<br>Chronic conditions 10263<br>Multi-morbidity 476<br>Multimorbidity 3160<br>Multimorbidities 165<br>Multi-morbidities 66<br>Long-term health condition 39	Online 91273<br>On-line 10976<br>Virtual 42066<br>ICT 3885<br>Communication technology 1639<br>Web 82786<br>Web based 23890<br>Website 13756<br>Web page 528<br>Multimedia 2881<br>e-learning 2196<br>Online social network 154<br>e-therapy 146<br>e-therapies 23<br>etherapies 1<br>etherapy 10<br>iCBT 589<br>cCBT 158<br><br>**MeSH**<br>Cell phone 8834<br>Mobile application 4432<br>Computers 14176<br>Therapy, computer assisted 44315<br>Wireless technology 3136<br>Information technology 237<br>Technology 210207<br>Electronic mail 1850<br>Internet 51939<br>Online social networking 57<br>Virtual reality 1079<br>Web browser 962<br>Multimedia 951		Behavior 363942<br>Behavioral 167313<br>Cognitive behavioral therapy 7242<br>Dialectic behavior therapy 10<br>Behavioural activation 328<br>Behavioral activation 862<br>Counseling 38031<br>Counselling 14505<br>Cognitive reframing 59<br>Cognitive restructuring 506<br>Self-help 3173<br>Self-management 14367<br><br>**MeSH**<br>Mental health services 37274<br>Health education 103830<br>Problem solving 9350<br>Psychotherapy 69239<br>Relaxation 8308<br>Self-management 1348<br>Self-help groups 3265

For peer review only

	**MeSH**<br>Neoplasms 1299462<br>Diabetes mellitus 192881<br>Pulmonary disease, chronic obstructive 31584<br>Stroke 83194<br>Cardiovascular diseases 805860<br>Dementia 81697<br>Cognition disorders 56940<br>Chronic disease 85925<br>Multimorbidity 503			
**Total for concept 1:**<br>**1 278 504**	**Total for concept 2:**<br>**3 399 238**	**Total for concept 3:**<br>**1 504 756**	**Total for concept 4:**<br>**1 400 395**	**Total for concept 5:**<br>**4 478 921**

Note: All MeSH terms are exploded

Total for complete search (includes date restriction): 3265

Date of search: September 4, 2019

For peer review only

For peer review only - http://bmjopen.bmj.com/site/about/guidelines.xhtml

Raw PubMed Search

(((((caregiver[Title/Abstract] OR caregivers[Title/Abstract] OR care-giver[Title/Abstract] OR care-givers[Title/Abstract] OR carer[Title/Abstract] OR carers[Title/Abstract] OR informal carer[Title/Abstract] OR informal carers[Title/Abstract] OR informal caregiver[Title/Abstract] OR informal caregivers[Title/Abstract] OR family[Title/Abstract] OR families[Title/Abstract] OR spouse[Title/Abstract] OR spousal[Title/Abstract] OR spouses[Title/Abstract] OR sibling[Title/Abstract] OR siblings[Title/Abstract] OR husband[Title/Abstract] OR husbands[Title/Abstract] OR wife[Title/Abstract] OR wives[Title/Abstract] OR partner[Title/Abstract] OR partners[Title/Abstract] OR parents[Title/Abstract] OR parent[Title/Abstract] OR friend[Title/Abstract] OR friends[Title/Abstract] OR relatives[Title/Abstract] OR relative[Title/Abstract] OR couple[Title/Abstract] OR couples[Title/Abstract] OR mother[Title/Abstract] OR mothers[Title/Abstract] OR fathers[Title/Abstract] OR father[Title/Abstract] OR support person[Title/Abstract] OR support persons[Title/Abstract] OR next of kin[Title/Abstract] OR significant other[Title/Abstract] OR significant others[Title/Abstract] OR caregivers[MeSH terms] OR family[MeSH terms] OR spouses[MeSH terms] OR siblings[MeSH terms] OR parents[MeSH terms] OR friends[MeSH terms] OR mothers[MeSH terms] OR fathers[MeSH terms]) AND (cancer[Title/Abstract] OR tumor[Title/Abstract] OR tumors[Title/Abstract] OR tumours[Title/Abstract] OR tumour[Title/Abstract] OR neoplasm[Title/Abstract] OR neoplasms[Title/Abstract] OR oncology[Title/Abstract] OR carcinoma[Title/Abstract] OR malignan*[Title/Abstract] OR melanoma[Title/Abstract] OR metastasis[Title/Abstract] OR lymphoma[Title/Abstract] OR leukemia[Title/Abstract] OR diabetes[Title/Abstract] OR diabetes mellitus[Title/Abstract] OR Type 1 diabetes[Title/Abstract] OR Type 2 diabetes[Title/Abstract] OR COPD[Title/Abstract] OR chronic obstructive pulmonary disease[Title/Abstract] OR COAD[Title/Abstract] OR chronic obstructive airway disease[Title/Abstract] OR chronic obstructive lung disease[Title/Abstract] OR chronic airflow obstruction[Title/Abstract] OR stroke[Title/Abstract] OR cerebrovascular accident[Title/Abstract] OR CVA[Title/Abstract] OR cerebral stroke[Title/Abstract] OR acute stroke[Title/Abstract] OR acute cerebrovascular accident[Title/Abstract] OR cerebrovascular stroke[Title/Abstract] OR brain vascular accident[Title/Abstract] OR cerebrovascular apoplexy[Title/Abstract] OR apoplexy[Title/Abstract] OR heart disease[Title/Abstract] OR cardiovascular disease[Title/Abstract] OR CVD[Title/Abstract] OR ischemic heart disease[Title/Abstract] OR IHD[Title/Abstract] OR coronary artery disease[Title/Abstract] OR CAD[Title/Abstract] OR angina[Title/Abstract] OR myocardial infarction[Title/Abstract] OR MI[Title/Abstract] OR heart attack[Title/Abstract] OR cardiac event[Title/Abstract] OR cardiac disease[Title/Abstract] OR dementia[Title/Abstract] OR alzheimer*[Title/Abstract] OR alzheimer's disease[Title/Abstract] OR vascular dementia[Title/Abstract] OR frontotemporal dementia[Title/Abstract] OR FTD[Title/Abstract] OR Lewy body[Title/Abstract] OR Lewy bodies[Title/Abstract] OR neurocognitive disorder[Title/Abstract] OR neurocognitive disorders[Title/Abstract] OR mild cognitive impairment[Title/Abstract] OR memory impair*[Title/Abstract] OR cognitive decline[Title/Abstract] OR chronic illness[Title/Abstract] OR chronic illnesses[Title/Abstract] OR chronic diseases[Title/Abstract] OR chronic disease[Title/Abstract] OR chronic condition[Title/Abstract] OR chronic conditions[Title/Abstract] OR multi-morbidity[Title/Abstract] OR multimorbidity[Title/Abstract] OR multimorbidities[Title/Abstract] OR multi-morbidities[Title/Abstract] OR long-term health condition[Title/Abstract] OR neoplasms[MeSH

For peer review only - http://bmjopen.bmj.com/site/about/guidelines.xhtml

terms] OR diabetes mellitus[MeSH terms] OR pulmonary disease, chronic obstructive[MeSH terms] OR stroke[MeSH terms] OR cardiovascular diseases[MeSH terms] OR dementia[MeSH terms] OR Cognition disorders[MeSH terms] OR chronic disease[MeSH terms] OR multimorbidity[MeSH terms]) AND (eHealth[Title/Abstract] OR e-health[Title/Abstract] OR e-mental health[Title/Abstract] OR emental health[Title/Abstract] OR mhealth[Title/Abstract] OR m-health[Title/Abstract] OR smartphone[Title/Abstract] OR cell phone[Title/Abstract] OR cellular phone[Title/Abstract] OR mobile phone[Title/Abstract] OR mobile app[Title/Abstract] OR mobile apps[Title/Abstract] OR mobile applications[Title/Abstract] OR mobile application[Title/Abstract] OR app[Title/Abstract] OR application[Title/Abstract] OR applications[Title/Abstract] OR apps[Title/Abstract] OR iPad[Title/Abstract] OR computer[Title/Abstract] OR tablet[Title/Abstract] OR computer based[Title/Abstract] OR computer assistive technology[Title/Abstract] OR technology[Title/Abstract] OR technologies[Title/Abstract] OR electronic communication[Title/Abstract] OR email[Title/Abstract] OR e-mail[Title/Abstract] OR text messaging[Title/Abstract] OR text message[Title/Abstract] OR internet[Title/Abstract] OR internet based[Title/Abstract] OR wireless[Title/Abstract] OR online[Title/Abstract] OR digital[Title/Abstract] OR on-line[Title/Abstract] OR virtual[Title/Abstract] OR ICT[Title/Abstract] OR communication technology[Title/Abstract] OR web[Title/Abstract] OR web based[Title/Abstract] OR website[Title/Abstract] OR web page[Title/Abstract] OR multimedia[Title/Abstract] OR e-learning[Title/Abstract] OR online social network[Title/Abstract] OR iCBT[Title/Abstract] OR cCBT[Title/Abstract] OR e-therapy[Title/Abstract] OR etherapy[Title/Abstract] OR etherapies[Title/Abstract] OR e-therapies[Title/Abstract] OR cell phone[MeSH terms] OR mobile application[MeSH terms] OR computers[MeSH terms] OR therapy, computer assisted[MeSH terms] OR wireless technology[MeSH terms] OR information technology[MeSH terms] OR technology[MeSH terms] OR electronic mail[MeSH terms] OR internet[MeSH terms] OR online social networking[MeSH terms] OR virtual reality[MeSH terms] OR web browser[MeSH terms] OR multimedia[MeSH terms]) AND (mental health[Title/Abstract] OR mood[Title/Abstract] OR mood disorder[Title/Abstract] OR depression[Title/Abstract] OR depressive[Title/Abstract] OR depressed[Title/Abstract] OR affective disorder[Title/Abstract] OR affect[Title/Abstract] OR negative affect[Title/Abstract] OR dysthymia[Title/Abstract] OR dysphoria[Title/Abstract] OR melancholic[Title/Abstract] OR anxiety[Title/Abstract] OR burden[Title/Abstract] OR distress[Title/Abstract] OR stress[Title/Abstract] OR well being[Title/Abstract] OR emotion[Title/Abstract] OR emotional[Title/Abstract] OR mood disorders[MeSH terms] OR depression[MeSH terms] OR anxiety[MeSH terms] OR anxiety disorders[MeSH terms] OR stress, psychological[MeSH terms] OR emotions[MeSH terms] OR mental health[MeSH terms]) AND (mental health service[Title/Abstract] OR mental health services[Title/Abstract] OR mental healthcare[Title/Abstract] OR therapy[Title/Abstract] OR therapies[Title/Abstract] OR treatment[Title/Abstract] OR treatments[Title/Abstract] OR interventions[Title/Abstract] OR intervention[Title/Abstract] OR program[Title/Abstract] OR programs[Title/Abstract] OR programmes[Title/Abstract] OR programme[Title/Abstract] OR psychological[Title/Abstract] OR psychologist[Title/Abstract] OR psychoeducation[Title/Abstract] OR psycho-education[Title/Abstract] OR CBT[Title/Abstract] OR cognitive behavioural therapy[Title/Abstract] OR cognitive therapy[Title/Abstract] OR health education[Title/Abstract] OR problem solving[Title/Abstract] OR problem solving therapy[Title/Abstract] OR PST[Title/Abstract] OR ACT[Title/Abstract] OR commitment therapy[Title/Abstract] OR behaviour[Title/Abstract] OR

For peer review only - http://bmjopen.bmj.com/site/about/guidelines.xhtml

behavioural[Title/Abstract] OR therapist[Title/Abstract] OR support[Title/Abstract] OR mindful[Title/Abstract] OR mindfulness[Title/Abstract] OR dialectic behaviour therapy[Title/Abstract] OR psychotherapy[Title/Abstract] OR relaxation[Title/Abstract] OR meditation[Title/Abstract] OR behavior[Title/Abstract] OR behavioral[Title/Abstract] OR cognitive behavioral therapy[Title/Abstract] OR behavioural activation[Title/Abstract] OR behavioral activation[Title/Abstract] OR dialectic behavior therapy[Title/Abstract] OR counseling[Title/Abstract] OR counselling[Title/Abstract] OR cognitive reframing[Title/Abstract] OR cognitive restructuring[Title/Abstract] OR self-help[Title/Abstract] OR self-management[Title/Abstract] OR mental health services[MeSH terms] OR health education[MeSH terms] OR problem solving[MeSH terms] OR psychotherapy[MeSH terms] OR relaxation[MeSH terms] OR self-management[MeSH terms] OR self-help groups[MeSH terms]))))) AND ("2007/01/01"[Date - Publication] : "3000"[Date - Publication])

For peer review only

For peer review only - http://bmjopen.bmj.com/site/about/guidelines.xhtml

PEER REVIEW ASSESSMENT #1: THIS SECTION TO BE FILLED IN BY THE REVIEWER

Reviewer: Professor Mariët Hagedoorn & Truus van Ittersum	Email: mariet.hagedoorn@umcg.nl	Date completed: 2019/10/03

1. TRANSLATION

A ---No revisions	☐
B --- Revision(s) suggested	☐
C --- Revision(s) required	X

If "B" or "C," please provide an explanation or example:

Does the search strategy match the research question/PICO?
I'm not familiar with the PRECIS-2 tool. Do you use it to rate the effectiveness of an internet technology intervention in reducing distress in caregivers as compared to a non-active control group?
Considering your overall question, I would expect that you would assess the effectiveness/successfulness of the implementation?
Are the search concepts clear?
Are there too many or too few PICO elements included?
You could consider not applying the fifth concept, but I can see the point why you do.
Are the search concepts too broad or too narrow?
Does the search retrieve too many or too few records?
Are unconventional or complex strategies explained?
The main focus is on successful implementation, which I find a relevant and timely issue to address in a review. However, the two approaches are not clear to me. For the first approach (which combination of intervention and implementation characteristics are associated with effective interventions), you want to include RCTs. To my understanding RCTs test the effectiveness of an intervention and usually does not include the aim of implementation. Will these studies be relevant to answer your overall question "which factors are related to successful implementation"? It would be helpful to clarify this.
On a practical note, the combination of two approaches appears to make the coding rather complex. Wouldn't it be better to do this in phases? First RCTs, than the remaining literature for approach 2?

I would advise to clarify the two approaches. As I commented above, it is not clear to me how approach one answers your overall research question. I also advise you to reconsider whether you really need two approaches, with specific inclusion criteria. In case you do need both approaches, would it be possible to use a two-step coding process and first search/code RCTs and than apply the second strategy?

<u>Author response:</u>
Thank you for your feedback. We believe that approach one (the qualitative comparative analysis) can produce results that relate to the overall research question regarding implementation of e-mental health interventions for caregivers. Approach one will only utilize pragmatic randomized controlled trials in the analysis. Pragmatic trials use conditions (e.g. the setting, follow up etc.) that more closely reflect how the interventions would be used in real-world settings. Therefore, pragmatic trials are more likely than explanatory trials to include details concerning implementation, which can be included in the qualitative comparative analysis to assess whether certain implementation factors are important for interventions effectiveness. Further, pragmatic trials examine effectiveness in near to real-world settings, and as such, provide estimates of intervention effectiveness that may better reflect estimates of effectiveness that may be found if the intervention was implemented in real-world practices. The PRECIS-2 tool evaluates how pragmatic the design of a randomized controlled trial is. By using this tool in the screening process, only trials with more pragmatic features will be included in the qualitative comparative analysis. The results of the qualitative comparative analysis can also be contrasted to the barriers and

facilitators identified in the thematic synthesis to determine if, for example, a factor identified as a facilitator to implementation in the thematic synthesis was also identified as important for intervention effectiveness in the qualitative comparative analysis.

We agree, the two approaches adds complexity to the literature screening process, however, if we were to only search for RCTs as a first step, we may miss many reports on implementation of interventions relevant to our research question.

2. BOOLEAN AND PROXIMITY OPERATORS

A ---No revisions	X
B --- Revision(s) suggested	☐
C --- Revision(s) required	☐

If "B" or "C," please provide an explanation or example:

Are Boolean or proximity operators used correctly?
Is the use of nesting with brackets appropriate and effective for the search?
If NOT is used, is this likely to result in any unintended exclusions?
Could precision be improved by using proximity operators (e.g. adjacent, near, within) or phrase searching instead of AND?
Is the width of proximity operators suitable (e.g. might adj5 pick up more variants than adj2)?

3. SUBJECT HEADINGS

A ---No revisions	X
B --- Revision(s) suggested	☐
C --- Revision(s) required	☐

If "B" or "C," please provide an explanation or example:

Are the subject headings relevant?
Are any relevant subject headings missing (e.g. previous index terms)?
Are any subject headings too broad or too narrow?
Are subject headings exploded where necessary and vice versa?
Are major headings ("starring" or restrict to focus) used? If so, if there adequate justification?
Are subheadings missing?
Are subheadings attached to subject headings? (Floating subheadings may be preferred)
Are floating subheadings relevant and used appropriately?
Are both subject headings and terms in free text used for each concept?

4. TEXT WORD SEARCHING

A ---No revisions	☐
B --- Revision(s)suggested	X
C --- Revision(s) required	☐

If "B" or "C," please provide an explanation or example:

General: there is an overload of terms. Some specific terms are not necessary as publications are already found by more general terms (<diabetes> also finds <diabetes mellitus>, <affect> also covers <negative affect>.

For peer review only

Does the search include all spelling variants in free text (e.g. UK versus US spelling)?
Does the search include all synonyms or antonyms (e.g. opposites)?
Does the search capture relevant truncation (e.g. is truncation at the correct place)?
Using the <*> helps narrowing down the number of terms, without loosing results. <Husband*> will also find <husbands>, <caregiver*> will also find <caregivers>. There are a lot of these terms in the list.
Are acronyms or abbreviations used appropriately? Do they capture irrelevant material? Are the full terms also included?
Are the keywords specific enough or too broad? Are too many or too few keywords used? Are stop words used?
Term “Technology”: Yields many results. Does not seem very relevant. Maybe skip this term?
Term “Web”: Yields mostly “Web of Science” OR “Web of Knowledge” hits. Maybe exclude? Reconsider?
Term “Multimorbidity”: Why multimorbidity? Populations should have a chronic illness and they are specified.
Term “long-term health condition”: To vague.
Have the appropriate fields been searched (e.g. is the choice of text world fields (.tw.) or all fields (.af.) appropriate? Are there any other fields to be included or excluded (database-specific)?
Should any long strings be broken into several shorter search statements?

For peer review only

Author response:
Thank you for these comments. Your feedback has been incorporated into a revised search strategy which has eliminated unnecessary terms and used more term truncations.

Although the terms “technology” and “web” are very broad, we feel they should remain in the primary search strategy as they may yield relevant results pertaining to technological or web based interventions. The terms “multimorbidity” and “long-term health condition” are included as we have come across publications which refer to the population under investigation using more generic terms, such as having multimorbidities or chronic diseases, with the specific health conditions only specified in the main text. Therefore, to ensure the search captures all publications that include caregivers of individuals with the health conditions of interest to this review, we included these more general terms.

5. SPELLING, SYNTAX, AND LINE NUMBERS

A ---No revisions	X
B --- Revision(s)suggested	☐
C --- Revision(s) required	☐

If “B” or “C,” please provide an explanation or example:

Are there any spelling errors
Are there errors in system syntax (e.g. the use of a truncation symbol from a different search interface)?
Are there incorrect line combinations or orphan lines (e.g. lines that are not referred to in the final summation that could indicate an error in an AND or OR statement)?

6. LIMITS AND FILTERS

A ---No revisions	X
B --- Revision(s) suggested	☐
C --- Revision(s) required	☐

If “B” or “C,” please provide an explanation or example:

Are all limits and filters used appropriately and are they relevant given the research question?
Are all limits and filters used appropriately and are they relevant for the database?
Are any potentially helpful limits or filters missing? Are the limits or filters too broad or too narrow? Can any limits or filters be added or removed?
Are sources cited for the filters used?

OVERALL EVALUATION (Note: If one or more "revision required" is noted above, the response below must be "revisions required".)

A ---No revisions	☐
B --- Revision(s) suggested	☐
C --- Revision(s) required	X

Additional comments: see above

For peer review only

For peer review only - http://bmjopen.bmj.com/site/about/guidelines.xhtml

PEER REVIEW ASSESSMENT #2: THIS SECTION TO BE FILLED IN BY THE REVIEWER

Reviewer: Dr. Nathan Davies	Email: n.m.davies@ucl.ac.uk	Date completed: 2019/10/07

1. TRANSLATION

A ---No revisions	X
B --- Revision(s) suggested	☐
C --- Revision(s) required	☐

If "B" or "C," please provide an explanation or example:

Does the search strategy match the research question/PICO?
Yes
Are the search concepts clear?
Yes
Are there too many or too few PICO elements included?
This is a very broad search and large area to study, it is doable but you may consider splitting the review into two.
Are the search concepts too broad or too narrow?
No, they are broad but needed for the question
Does the search retrieve too many or too few records?
My only concern is that this is going to be a very large review and managing such a large review
Are unconventional or complex strategies explained?
N/a

2. BOOLEAN AND PROXIMITY OPERATORS

A ---No revisions	X
B --- Revision(s) suggested	☐
C --- Revision(s) required	☐

If "B" or "C," please provide an explanation or example:

Are Boolean or proximity operators used correctly?
Yes
Is the use of nesting with brackets appropriate and effective for the search?
Yes
If NOT is used, is this likely to result in any unintended exclusions?
Could precision be improved by using proximity operators (e.g. adjacent, near, within) or phrase searching instead of AND?
No
Is the width of proximity operators suitable (e.g. might adj5 pick up more variants than adj2)?
n/a

For peer review only

3. SUBJECT HEADINGS

A ---No revisions	X
B --- Revision(s) suggested	☐
C --- Revision(s) required	☐

If "B" or "C," please provide an explanation or example:

Are the subject headings relevant?
Yes
Are any relevant subject headings missing (e.g. previous index terms)?
None obvious that I could see
Are any subject headings too broad or too narrow?
No
Are subject headings exploded where necessary and vice versa?
Yes
Are major headings ("starring" or restrict to focus) used? If so, if there adequate justification?
No
Are subheadings missing?
No
Are subheadings attached to subject headings? (Floating subheadings may be preferred)
n/a
Are floating subheadings relevant and used appropriately?
n/a
Are both subject headings and terms in free text used for each concept?
Yes

4. TEXT WORD SEARCHING

A ---No revisions	☐
B --- Revision(s)suggested	X
C --- Revision(s) required	☐

If "B" or "C," please provide an explanation or example:

Does the search include all spelling variants in free text (e.g. UK versus US spelling)?
Add MCI for mild cognitive impairment
Look at searching the term digital health.
Look at cognitive stimulation therapy which can be used with people with dementia
Does the search include all synonyms or antonyms (e.g. opposites)?
See above
Does the search capture relevant truncation (e.g. is truncation at the correct place)?
You can add * to some words which may want to be searched with and without an s at the end for example husband and husbands could just be searched as husband*. But this would not effect the numbers you have already retrieved but may make the search easier to follow with less terms.
Are acronyms or abbreviations used appropriately? Do they capture irrelevant material? Are the full terms also included?
See above
Are the keywords specific enough or too broad? Are too many or too few keywords used? Are stop words used?
See above

For peer review only - http://bmjopen.bmj.com/site/about/guidelines.xhtml

Have the appropriate fields been searched (e.g. is the choice of text world fields (.tw.) or all fields (.af.) appropriate? Are there any other fields to be included or excluded (database-specific)?
Yes
Should any long strings be broken into several shorter search statements?
No

Author response:
Thank you for these suggestions. MCI has been added to the search strategy and more terms have been truncated using the *. Digital health and cognitive stimulation therapy are very relevant concepts, however, the were not added to the search strategy as they should be captured by the terms "digital" and "therapy", respectively, in the existing search strategy.

5. SPELLING, SYNTAX, AND LINE NUMBERS

A ---No revisions	X
B --- Revision(s)suggested	☐
C --- Revision(s) required	☐

If "B" or "C," please provide an explanation or example:

Are there any spelling errors
no
Are there errors in system syntax (e.g. the use of a truncation symbol from a different search interface)?
no
Are there incorrect line combinations or orphan lines (e.g. lines that are not referred to in the final summation that could indicate an error in an AND or OR statement)?
no

6. LIMITS AND FILTERS

A ---No revisions	X
B --- Revision(s) suggested	☐
C --- Revision(s) required	☐

If "B" or "C," please provide an explanation or example:

Are all limits and filters used appropriately and are they relevant given the research question?
The year limit seems appropriate you may even be able to change this to 2009 as last 10 years is a long time in technology so anything older than 10 years is most likely to be irrelevant now.
Are all limits and filters used appropriately and are they relevant for the database?
Yes – although for non-english/sweedish language you could rapidly appraise the English abstract if available and if seems very relevant seek translation of the remainder of the article.
Are any potentially helpful limits or filters missing? Are the limits or filters too broad or too narrow? Can any limits or filters be added or removed?
no
Are sources cited for the filters used?
Don't understand this question

For peer review only - http://bmjopen.bmj.com/site/about/guidelines.xhtml

OVERALL EVALUATION (Note: If one or more "revision required" is noted above, the response below must be "revisions required".)

A ---No revisions	☐
B --- Revision(s) suggested	X
C --- Revision(s) required	☐

Additional comments:

For peer review only

For peer review only - http://bmjopen.bmj.com/site/about/guidelines.xhtml

Implementation of e-mental health interventions for informal caregivers of adults with chronic diseases: a protocol for a mixed methods systematic review with a qualitative comparative analysis

Supplementary File 3: Search Strategy

PubMed

#	
**1**	caregiver*[Title/Abstract] OR<br>care-giver*[Title/Abstract] OR<br>carer*[Title/Abstract] OR<br>family[Title/Abstract] OR<br>families[Title/Abstract] OR<br>spous*[Title/Abstract] OR<br>sibling*[Title/Abstract] OR<br>husband*[Title/Abstract] OR<br>wife[Title/Abstract] OR<br>wives[Title/Abstract] OR<br>partner[Title/Abstract] OR<br>partners[Title/Abstract] OR<br>parents[Title/Abstract] OR<br>parent[Title/Abstract] OR<br>friend[Title/Abstract] OR<br>friends[Title/Abstract] OR<br>relative[Title/Abstract] OR<br>relatives[Title/Abstract] OR<br>couple[Title/Abstract] OR<br>couples[Title/Abstract] OR<br>mother*[Title/Abstract] OR<br>father*[Title/Abstract] OR<br>support person*[Title/Abstract] OR<br>next of kin[Title/Abstract] OR<br>significant other*[Title/Abstract] OR<br>caregivers[MeSH terms] OR<br>family[MeSH terms] OR<br>spouses[MeSH terms] OR<br>siblings[MeSH terms] OR<br>parents[MeSH terms] OR<br>friends[MeSH terms] OR<br>mothers[MeSH terms] OR<br>fathers[MeSH terms]
**2**	cancer[Title/Abstract] OR<br>tumor[Title/Abstract] OR<br>tumors[Title/Abstract] OR<br>tumours[Title/Abstract] OR<br>tumour[Title/Abstract] OR<br>neoplasm*[Title/Abstract] OR<br>oncology[Title/Abstract] OR<br>carcinoma[Title/Abstract] OR<br>malignan*[Title/Abstract] OR

For peer review only

For peer review only - http://bmjopen.bmj.com/site/about/guidelines.xhtml

	melanoma[Title/Abstract] OR<br>metastasis[Title/Abstract] OR<br>lymphoma[Title/Abstract] OR<br>leukemia[Title/Abstract] OR<br>diabetes[Title/Abstract] OR<br>COPD[Title/Abstract] OR<br>chronic obstructive pulmonary disease[Title/Abstract] OR<br>COAD[Title/Abstract] OR<br>chronic obstructive airway disease[Title/Abstract] OR<br>chronic obstructive lung disease[Title/Abstract] OR<br>chronic airflow obstruction[Title/Abstract] OR<br>stroke[Title/Abstract] OR<br>cerebrovascular accident[Title/Abstract] OR<br>CVA[Title/Abstract] OR<br>acute cerebrovascular accident[Title/Abstract] OR<br>brain vascular accident[Title/Abstract] OR<br>apoplexy[Title/Abstract] OR<br>heart disease[Title/Abstract] OR<br>cardiovascular disease[Title/Abstract] OR<br>CVD[Title/Abstract] OR<br>ischemic heart disease[Title/Abstract] OR<br>IHD[Title/Abstract] OR<br>coronary artery disease[Title/Abstract] OR<br>CAD[Title/Abstract] OR<br>angina[Title/Abstract] OR<br>myocardial infarction[Title/Abstract] OR<br>MI[Title/Abstract] OR<br>heart attack[Title/Abstract] OR<br>cardiac event[Title/Abstract] OR<br>cardiac disease[Title/Abstract] OR<br>dementia[Title/Abstract] OR<br>alzheimer*[Title/Abstract] OR<br>FTD[Title/Abstract] OR<br>Lewy body[Title/Abstract] OR<br>Lewy bodies[Title/Abstract] OR<br>neurocognitive disorder*[Title/Abstract] OR<br>MCI[Title/Abstract] OR<br>mild cognitive impairment[Title/Abstract] OR<br>memory impair*[Title/Abstract] OR<br>cognitive decline[Title/Abstract] OR<br>chronic illness*[Title/Abstract] OR<br>chronic disease*[Title/Abstract] OR<br>chronic condition*[Title/Abstract] OR<br>multi-morbidit*[Title/Abstract] OR<br>multimorbidit*[Title/Abstract] OR<br>long-term health condition*[Title/Abstract] OR<br>neoplasms[MeSH terms] OR<br>diabetes mellitus[MeSH terms] OR<br>pulmonary disease, chronic obstructive[MeSH terms] OR<br>stroke[MeSH terms] OR<br>cardiovascular diseases[MeSH terms] OR

For peer review only

	dementia[MeSH terms] OR<br>cognition disorders[MeSH terms] OR<br>chronic disease[MeSH terms] OR<br>multimorbidity[MeSH terms]
**3**	eHealth[Title/Abstract] OR<br>e-health[Title/Abstract] OR<br>e-mental health[Title/Abstract] OR<br>emental health[Title/Abstract] OR<br>mhealth[Title/Abstract] OR<br>m-health[Title/Abstract] OR<br>smartphone[Title/Abstract] OR<br>cell phone[Title/Abstract] OR<br>cellular phone[Title/Abstract] OR<br>mobile phone[Title/Abstract] OR<br>app[Title/Abstract] OR<br>apps[Title/Abstract] OR<br>application[Title/Abstract] OR<br>applications[Title/Abstract] OR<br>iPad[Title/Abstract] OR<br>computer[Title/Abstract] OR<br>tablet[Title/Abstract] OR<br>technology[Title/Abstract] OR<br>technologies[Title/Abstract] OR<br>electronic communication[Title/Abstract] OR<br>email[Title/Abstract] OR<br>e-mail[Title/Abstract] OR<br>text messag*[Title/Abstract] OR<br>internet[Title/Abstract] OR<br>wireless[Title/Abstract] OR<br>online[Title/Abstract] OR<br>digital[Title/Abstract] OR<br>on-line[Title/Abstract] OR<br>virtual[Title/Abstract] OR<br>ICT[Title/Abstract] OR<br>web[Title/Abstract] OR<br>website[Title/Abstract] OR<br>multimedia[Title/Abstract] OR<br>e-learning[Title/Abstract] OR<br>online social network[Title/Abstract] OR<br>iCBT[Title/Abstract] OR<br>cCBT[Title/Abstract] OR<br>e-therap*[Title/Abstract] OR<br>etherap*[Title/Abstract] OR<br>cell phone[MeSH terms] OR<br>mobile application[MeSH terms] OR<br>computers[MeSH terms] OR<br>therapy, computer assisted[MeSH terms] OR<br>wireless technology[MeSH terms] OR<br>information technology[MeSH terms] OR<br>technology[MeSH terms] OR<br>electronic mail[MeSH terms] OR

For peer review only

	internet[MeSH terms] OR<br>online social networking[MeSH terms] OR<br>virtual reality[MeSH terms] OR<br>web browser[MeSH terms] OR<br>multimedia[MeSH terms]
**4**	mental health[Title/Abstract] OR<br>mood[Title/Abstract] OR<br>depress*[Title/Abstract] OR<br>affective disorder[Title/Abstract] OR<br>negative affect[Title/Abstract] OR<br>dysthymia[Title/Abstract] OR<br>dysphoria[Title/Abstract] OR<br>melancholic[Title/Abstract] OR<br>anxiety[Title/Abstract] OR<br>burden[Title/Abstract] OR<br>distress[Title/Abstract] OR<br>stress[Title/Abstract] OR<br>well being[Title/Abstract] OR<br>emotion[Title/Abstract] OR<br>emotional[Title/Abstract] OR<br>mood disorders[MeSH terms] OR<br>depression[MeSH terms] OR<br>anxiety[MeSH terms] OR<br>anxiety disorders[MeSH terms] OR<br>stress, psychological[MeSH terms] OR<br>emotions[MeSH terms] OR<br>mental health[MeSH terms]
**5**	mental health service*[Title/Abstract] OR<br>mental healthcare[Title/Abstract] OR<br>therapy[Title/Abstract] OR<br>therapies[Title/Abstract] OR<br>treatment*[Title/Abstract] OR<br>intervention*[Title/Abstract] OR<br>program*[Title/Abstract] OR<br>psychological[Title/Abstract] OR<br>psychologist[Title/Abstract] OR<br>psychoeducation[Title/Abstract] OR<br>psycho-education[Title/Abstract] OR<br>CBT[Title/Abstract] OR<br>health education[Title/Abstract] OR<br>problem solving[Title/Abstract] OR<br>PST[Title/Abstract] OR<br>ACT[Title/Abstract] OR<br>behaviour*[Title/Abstract] OR<br>therapist[Title/Abstract] OR<br>support[Title/Abstract] OR<br>mindful*[Title/Abstract] OR<br>psychotherapy[Title/Abstract] OR<br>relaxation[Title/Abstract] OR<br>meditation[Title/Abstract] OR<br>behavior*[Title/Abstract] OR

For peer review only

	counseling[Title/Abstract] OR<br>counselling[Title/Abstract] OR<br>cognitive reframing[Title/Abstract] OR<br>cognitive restructuring[Title/Abstract] OR<br>self-help[Title/Abstract] OR<br>self-management[Title/Abstract] OR<br>blended[Title/Abstract] OR<br>mental health services[MeSH terms] OR<br>health education[MeSH terms] OR<br>problem solving[MeSH terms] OR<br>psychotherapy[MeSH terms] OR<br>relaxation[MeSH terms] OR<br>self-management[MeSH terms] OR<br>self-help groups[MeSH terms]
**6**	"2007/01/01"[Date - Publication] : "3000"[Date - Publication]
**7**	#1 AND #2 AND #3 AND #4 AND #5 AND #6

CINAHL Plus with Full Text

	((TI (caregiver* OR care-giver* OR carer* OR family OR families OR spous* OR sibling* OR husband* OR wife OR wives OR partner OR partners OR parents OR parent OR friend OR friends OR relative OR relatives OR couple OR couples OR mother* OR father* OR support person* OR next of kin OR significant other*) OR AB (caregiver* OR care-giver* OR carer* OR family OR families OR spous* OR sibling* OR husband* OR wife OR wives OR partner OR partners OR parents OR parent OR friend OR friends OR relative OR relatives OR couple OR couples OR mother* OR father* OR support person* OR next of kin OR significant other*) OR MH (caregivers+ OR family+ OR spouses+ OR siblings+ OR parents+ OR friends+ OR mothers+ OR fathers+)) AND (TI (cancer OR tumor OR tumors OR tumours OR tumour OR neoplasm* OR oncology OR carcinoma OR malignan* OR melanoma OR metastasis OR lymphoma OR leukemia OR diabetes OR COPD OR chronic obstructive pulmonary disease OR COAD OR chronic obstructive airway disease OR chronic obstructive lung disease OR chronic airflow obstruction OR stroke OR cerebrovascular accident OR CVA OR acute cerebrovascular accident OR brain vascular accident OR apoplexy OR heart disease OR cardiovascular disease OR CVD OR ischemic heart disease OR IHD OR coronary artery disease OR CAD OR angina OR myocardial infarction OR MI OR heart attack OR cardiac event OR cardiac disease OR dementia OR alzheimer* OR FTD OR Lewy body OR Lewy bodies OR neurocognitive disorder* OR MCI OR mild cognitive impairment OR memory impair* OR cognitive decline OR chronic illness* OR chronic disease* OR chronic condition* OR multi-morbidit* OR multimorbidit* OR long-term health condition*) OR AB (cancer OR tumor OR tumors OR tumours OR tumour OR neoplasm* OR oncology OR carcinoma OR malignan* OR melanoma OR metastasis OR lymphoma OR leukemia OR diabetes OR COPD OR chronic obstructive pulmonary disease OR COAD OR chronic obstructive airway disease OR chronic obstructive lung disease OR chronic airflow obstruction OR stroke OR cerebrovascular accident OR CVA OR acute cerebrovascular accident OR brain vascular accident OR apoplexy OR heart disease OR cardiovascular disease OR CVD OR ischemic heart disease OR IHD OR coronary artery disease OR CAD OR angina OR myocardial infarction OR MI OR heart attack OR cardiac event OR cardiac disease OR dementia OR alzheimer* OR FTD OR Lewy body OR Lewy bodies OR neurocognitive disorder* OR MCI OR mild cognitive impairment OR memory impair* OR cognitive decline OR chronic illness* OR chronic disease* OR chronic condition* OR multi-morbidit* OR multimorbidit* OR long-term health condition*) OR MH (neoplasms+ OR diabetes mellitus+ OR pulmonary disease, chronic obstructive+ OR stroke+ OR

For peer review only

	cardiovascular diseases+ OR dementia+ OR Cognition disorders+ OR Chronic disease+ OR multimorbidity+)) AND (TI (eHealth OR e-health OR e-mental health OR emental health OR mhealth OR m-health OR smartphone OR cell phone OR cellular phone OR mobile phone OR app OR application OR applications OR apps OR iPad OR computer OR tablet OR technology OR technologies OR electronic communication OR email OR e-mail OR text messag* OR internet OR wireless OR online OR digital OR on-line OR virtual OR ICT OR web OR website OR multimedia OR e-learning OR online social network OR iCBT OR cCBT OR e-therap* OR etherap*) OR AB (eHealth OR e-health OR e-mental health OR emental health OR mhealth OR m-health OR smartphone OR cell phone OR cellular phone OR mobile phone OR app OR application OR applications OR apps OR iPad OR computer OR tablet OR technology OR technologies OR electronic communication OR email OR e-mail OR text messag* OR internet OR wireless OR online OR digital OR on-line OR virtual OR ICT OR web OR website OR multimedia OR e-learning OR online social network OR iCBT OR cCBT OR e-therap* OR etherap*) OR MH (cell phone+ OR mobile application+ OR computers+ OR therapy, computer assisted+ OR wireless technology+ OR information technology+ OR technology+ OR electronic mail+ OR internet+ OR online social networking+ OR virtual reality+ OR web browser+ OR multimedia+)) AND (TI (mental health OR mood OR depress* OR affective disorder OR negative affect OR dysthymia OR dysphoria OR melancholic OR anxiety OR burden OR distress OR stress OR well being OR emotion OR emotional) OR AB (mental health OR mood OR depress* OR affective disorder OR negative affect OR dysthymia OR dysphoria OR melancholic OR anxiety OR burden OR distress OR stress OR well being OR emotion OR emotional) OR MH (mood disorders+ OR depression+ OR anxiety+ OR anxiety disorders+ OR stress, psychological+ OR emotions+ OR mental health+)) AND (TI (mental health service* OR mental healthcare OR therapy OR therapies OR treatment* OR intervention* OR program* OR psychological OR psychologist OR psychoeducation OR psycho-education OR CBT OR health education OR problem solving OR PST OR ACT OR behaviour* OR therapist OR support OR mindful* OR psychotherapy OR relaxation OR meditation OR behavior* OR counseling OR counselling OR cognitive reframing OR cognitive restructuring OR self-help OR self-management OR blended) OR AB (mental health service* OR mental healthcare OR therapy OR therapies OR treatment* OR intervention* OR program* OR psychological OR psychologist OR psychoeducation OR psycho-education OR CBT OR health education OR problem solving OR PST OR ACT OR behaviour* OR therapist OR support OR mindful* OR psychotherapy OR relaxation OR meditation OR behavior* OR counseling OR counselling OR cognitive reframing OR cognitive restructuring OR self-help OR self-management OR blended) OR MH (mental health services+ OR health education+ OR problem solving+ OR psychotherapy+ OR relaxation+ OR self-management+ OR self-help groups+)))

The Cochrane Library

	(((caregiver*):ti,ab,kw OR (care-giver*):ti,ab,kw OR (carer*):ti,ab,kw OR (family):ti,ab,kw OR (families):ti,ab,kw OR (spous*):ti,ab,kw OR (sibling*):ti,ab,kw OR (husband*):ti,ab,kw OR (wife):ti,ab,kw OR (wives):ti,ab,kw OR (partner):ti,ab,kw OR (partners):ti,ab,kw OR (parents):ti,ab,kw OR (parent):ti,ab,kw OR (friend):ti,ab,kw OR (friends):ti,ab,kw OR (relatives):ti,ab,kw OR (relative):ti,ab,kw OR (couples):ti,ab,kw OR (couple):ti,ab,kw OR (mother*):ti,ab,kw OR (father*):ti,ab,kw OR (support NEXT person*):ti,ab,kw OR ("next of kin"):ti,ab,kw OR (significant NEXT other*):ti,ab,kw OR [mh Caregivers] OR [mh Family] OR [mh Spouses] OR [mh Siblings] OR [mh Parents] OR [mh Friends] OR [mh Mothers] OR [mh Fathers]) AND ((cancer):ti,ab,kw OR (tumor):ti,ab,kw OR (tumors):ti,ab,kw OR (tumours):ti,ab,kw OR (tumour):ti,ab,kw OR (neoplasm*):ti,ab,kw OR (oncology):ti,ab,kw OR (carcinoma):ti,ab,kw OR (malignan*):ti,ab,kw OR (melanoma):ti,ab,kw OR (metastasis):ti,ab,kw OR (lymphoma):ti,ab,kw

For peer review only

	OR (leukemia):ti,ab,kw OR (diabetes):ti,ab,kw OR (COPD):ti,ab,kw OR ("chronic obstructive pulmonary disease"):ti,ab,kw OR (COAD):ti,ab,kw OR ("chronic obstructive airway disease"):ti,ab,kw OR ("chronic obstructive lung disease"):ti,ab,kw OR ("chronic airflow obstruction"):ti,ab,kw OR (stroke):ti,ab,kw OR ("cerebrovascular accident"):ti,ab,kw OR (CVA):ti,ab,kw OR ("acute cerebrovascular accident"):ti,ab,kw OR ("brain vascular accident"):ti,ab,kw OR (apoplexy):ti,ab,kw OR ("heart disease"):ti,ab,kw OR ("cardiovascular disease"):ti,ab,kw OR (CVD):ti,ab,kw OR ("ischemic heart disease"):ti,ab,kw OR (IHD):ti,ab,kw OR ("coronary artery disease"):ti,ab,kw OR (CAD):ti,ab,kw OR (angina):ti,ab,kw OR ("myocardial infarction"):ti,ab,kw OR (MI):ti,ab,kw OR ("heart attack"):ti,ab,kw OR ("cardiac event"):ti,ab,kw OR ("cardiac disease"):ti,ab,kw OR (dementia):ti,ab,kw OR (Alzheimer*):ti,ab,kw OR (FTD):ti,ab,kw OR ("Lewy body"):ti,ab,kw OR ("Lewy bodies"):ti,ab,kw OR (neurocognitive NEXT disorder*):ti,ab,kw OR ("mild cognitive impairment"):ti,ab,kw OR (MCI):ti,ab,kw OR (memory NEXT impair*):ti,ab,kw OR ("cognitive decline"):ti,ab,kw OR (chronic NEXT illness*):ti,ab,kw OR (chronic NEXT disease*):ti,ab,kw OR (chronic NEXT condition*):ti,ab,kw OR (multi-morbidit*):ti,ab,kw OR (multimorbidit*):ti,ab,kw OR (long-term NEXT health NEXT condition*):ti,ab,kw OR [mh Neoplasms] OR [mh "Diabetes mellitus"] OR [mh "pulmonary disease, chronic obstructive"] OR [mh stroke] OR [mh "cardiovascular diseases"] OR [mh dementia] OR [mh "cognition disorders"] OR [mh "chronic disease"] OR [mh Multimorbidity]) AND ((eHealth):ti,ab,kw OR (e-health):ti,ab,kw OR ("e-mental health"):ti,ab,kw OR ("emental health"):ti,ab,kw OR (mhealth):ti,ab,kw OR (m-health):ti,ab,kw OR (smartphone):ti,ab,kw OR ("cell phone"):ti,ab,kw OR ("cellular phone"):ti,ab,kw OR ("mobile phone"):ti,ab,kw OR (app):ti,ab,kw OR (application):ti,ab,kw OR (applications):ti,ab,kw OR (apps):ti,ab,kw OR (iPad):ti,ab,kw OR (computer):ti,ab,kw OR (tablet):ti,ab,kw OR (technology):ti,ab,kw OR (technologies):ti,ab,kw OR ("electronic communication"):ti,ab,kw OR (email):ti,ab,kw OR (e-mail):ti,ab,kw OR (text NEXT messag*):ti,ab,kw OR (internet):ti,ab,kw OR (wireless):ti,ab,kw OR (online):ti,ab,kw OR (digital):ti,ab,kw OR (on-line):ti,ab,kw OR (virtual):ti,ab,kw OR (ICT):ti,ab,kw OR (web):ti,ab,kw OR (website):ti,ab,kw OR (multimedia):ti,ab,kw OR (e-learning):ti,ab,kw OR ("online social network"):ti,ab,kw OR (iCBT):ti,ab,kw OR (cCBT):ti,ab,kw OR (e-therap*):ti,ab,kw OR (etherap*):ti,ab,kw OR [mh "cell phone"] OR [mh "mobile application"] OR [mh computers] OR [mh "therapy, computer assisted"] OR [mh "wireless technology"] OR [mh "information technology"] OR [mh technology] OR [mh "electronic mail"] OR [mh internet] OR [mh "online social networking"] OR [mh "virtual reality"] OR [mh "web browser"] OR [mh multimedia]) AND (("mental health"):ti,ab,kw OR (mood):ti,ab,kw OR (depress*):ti,ab,kw OR ("affective disorder"):ti,ab,kw OR ("negative affect"):ti,ab,kw OR (dysthymia):ti,ab,kw OR (dysphoria):ti,ab,kw OR (melancholic):ti,ab,kw OR (anxiety):ti,ab,kw OR (burden):ti,ab,kw OR (distress):ti,ab,kw OR (stress):ti,ab,kw OR ("well being"):ti,ab,kw OR (emotion):ti,ab,kw OR (emotional):ti,ab,kw OR [mh "mood disorders"] OR [mh depression] OR [mh anxiety] OR [mh "anxiety disorders"] OR [mh "stress, psychological"] OR [mh emotions] OR [mh "mental health"]) AND ((mental NEXT health NEXT service*):ti,ab,kw OR ("mental healthcare"):ti,ab,kw OR (therapy):ti,ab,kw OR (therapies):ti,ab,kw OR (treatment*):ti,ab,kw OR (intervention*):ti,ab,kw OR (program*):ti,ab,kw OR (psychological):ti,ab,kw OR (psychologist):ti,ab,kw OR (psychoeducation):ti,ab,kw OR (psycho-education):ti,ab,kw OR (CBT):ti,ab,kw OR ("health education"):ti,ab,kw OR ("problem solving"):ti,ab,kw OR (PST):ti,ab,kw OR (ACT):ti,ab,kw OR (behaviour*):ti,ab,kw OR (therapist):ti,ab,kw OR (support):ti,ab,kw OR (mindful*):ti,ab,kw OR (psychotherapy):ti,ab,kw OR (relaxation):ti,ab,kw OR (meditation):ti,ab,kw OR (behavior*):ti,ab,kw OR (counseling):ti,ab,kw OR (counselling):ti,ab,kw OR ("cognitive reframing"):ti,ab,kw OR ("cognitive restructuring"):ti,ab,kw OR (self-help):ti,ab,kw OR (self-management):ti,ab,kw OR (blended):ti,ab,kw OR [mh "mental health services"] OR [mh "health education"] OR [mh "problem solving"] OR [mh psychotherapy] OR [mh relaxation] OR [mh self-management] OR [mh "self-help groups"]))

For peer review only

EMBASE

Note: The broad search terms used in the search strategy, resulted in the retrieval of a significant number of irrelevant publications in the EMBASE database. Therefore, the search strategy for EMBASE was modified to eliminate key broad terms and when appropriate, replace them with phrases.

#	Searches
1	(caregiver* or care-giver* or carer* or family or families or spous* or sibling* or husband* or wife or wives or partner or partners or parents or parent or friend or friends or relatives or couple or couples or mother* or father* or support person* or next of kin or significant other*).ab,ti.
2	exp caregiver/
3	exp family/
4	exp spouse/
5	exp sibling/
6	exp parent/
7	exp friend/
8	exp mother/
9	exp father/
10	1 or 2 or 3 or 4 or 5 or 6 or 7 or 8 or 9
11	(cancer or tumor or tumors or tumours or tumour or neoplasm* or oncology or carcinoma or malignan* or melanoma or metastasis or lymphoma or leukemia or diabetes or COPD or chronic obstructive pulmonary disease or COAD or chronic obstructive airway disease or chronic obstructive lung disease or chronic airflow obstruction or stroke or cerebrovascular accident or CVA or acute cerebrovascular accident or brain vascular accident or apoplexy or heart disease or cardiovascular disease or CVD or ischemic heart disease or IHD or coronary artery disease or CAD or angina or myocardial infarction or MI or heart attack or cardiac event or cardiac disease or dementia or alzheimer* or FTD or Lewy body or Lewy bodies or neurocognitive disorder* or MCI or mild cognitive impairment or memory impair* or cognitive decline or chronic illness* or chronic disease* or chronic condition* or multi-morbidit* or multimorbidit* or long-term health condition*).ab,ti.
12	exp neoplasm/
13	exp diabetes mellitus/
14	exp chronic obstructive lung disease/
15	exp cerebrovascular accident/
16	exp cardiovascular disease/
17	exp dementia/

18	exp chronic disease/
19	exp multiple chronic conditions/
20	(eHealth or e-health or e-mental health or emental health or mhealth or m-health or smartphone or cell phone or cellular phone or mobile phone or app or apps or iPad or computer or tablet or electronic communication or email or e-mail or text messag* or internet or wireless or online or digital or on-line or virtual or ICT or website or multimedia or e-learning or online social network or iCBT or cCBT or e-therap* or etherap* or web-based or (mobile adj2 technolog*) or (technolog* adj2 solution*) or (technology adj2 based)).ab,ti.
21	exp mobile phone/
22	exp mobile application/
23	exp computer/
24	exp computer assisted therapy/
25	exp wireless communication/
26	exp information technology/
27	exp technology/
28	exp e-mail/
29	exp Internet/
30	exp virtual reality/
31	exp web browser/
32	exp multimedia/
33	20 or 21 or 22 or 23 or 24 or 25 or 26 or 27 or 28 or 29 or 30 or 31 or 32
34	(mental health or mood or depress* or affective disorder or negative affect or dysthymia or dysphoria or melancholic or anxiety or burden or distress or stress or well being or emotion or emotional).ab,ti.
35	exp mood disorder/
36	exp depression/
37	exp anxiety/ or exp anxiety disorder/
38	exp mental stress/
39	exp mental health/
40	(mental health service* or mental healthcare or intervention* or program* or psychological or psychologist or psychoeducation or psycho-education or CBT or health education or problem solving or PST or ACT or behaviour* or therapist or support or mindful* or psychotherapy or relaxation or meditation or behavior* or counseling or counselling or cognitive reframing or cognitive restructuring or self-help or self-management or (group adj therapy) or (psycho* adj therapy) or (cognitive adj2 therapy) or (behavio* adj2 therapy) or (problem adj2 therapy) or (acceptance adj2 therapy) or blended).ab,ti.

41	exp mental health service/
42	exp health education/
43	exp problem solving/
44	exp psychotherapy/
45	exp self help/
46	34 or 35 or 36 or 37 or 38 or 39
47	40 or 41 or 42 or 43 or 44 or 45
48	11 or 12 or 13 or 14 or 15 or 16 or 17 or 18 or 19
49	10 and 33 and 46 and 47 and 48
50	limit 49 to yr="2007 -Current"

PsychINFO

#	Searches
1	(caregiver* or care-giver* or carer* or family or families or spous* or sibling* or husband* or wife or wives or partner or partners or parents or parent or friend or friends or relative or relatives or couple or couples or mother* or father* or support person* or next of kin or significant other*).ab,ti.
2	exp Caregivers/
3	exp Family/
4	exp Spouses/
5	exp Siblings/
6	exp Parents/
7	exp Mothers/
8	exp Fathers/
9	exp Friends/
10	(cancer or tumor or tumors or tumours or tumour or neoplasm* or oncology or carcinoma or malignan* or melanoma or metastasis or lymphoma or leukemia or diabetes or COPD or chronic obstructive pulmonary disease or COAD or chronic obstructive airway disease or chronic obstructive lung disease or chronic airflow obstruction or stroke or cerebrovascular accident or CVA or acute cerebrovascular accident or brain vascular accident or apoplexy or heart disease or cardiovascular disease or CVD or ischemic heart disease or IHD or coronary artery disease or CAD or angina or myocardial infarction or MI or heart attack or cardiac event or cardiac disease or dementia or alzheimer* or FTD or Lewy body or Lewy bodies or neurocognitive disorder* or MCI or mild cognitive impairment or memory impair* or cognitive

	decline or chronic illness* or chronic disease* or chronic condition* or multi-morbidit* or multimorbidit* or long-term health condition*).ab,ti.
11	exp Neoplasms/
12	exp Diabetes Mellitus/
13	exp Chronic Obstructive Pulmonary Disease/
14	exp Stroke/
15	exp Dementia/
16	exp Cognitive Impairment/
17	exp Chronic Illness/
18	exp Comorbidity/
19	exp Cardiovascular Disorders/
20	(eHealth or e-health or e-mental health or emental health or mhealth or m-health or smartphone or cell phone or cellular phone or mobile phone or app or application or applications or apps or iPad or computer or tablet or technology or technologies or electronic communication or email or e-mail or text messag* or internet or wireless or online or digital or on-line or virtual or ICT or web or website or multimedia or e-learning or online social network or iCBT or cCBT or e-therap* or etherap*).ab,ti.
21	exp Mobile Phones/
22	exp Mobile Applications/
23	exp Computers/
24	exp Computer Assisted Therapy/
25	exp Wireless Technologies/
26	exp "Information and Communication Technology"/
27	exp Technology/
28	exp Computer Mediated Communication/
29	exp Internet/
30	exp Online Social Networks/
31	exp Virtual Reality/
32	exp Websites/
33	exp Multimedia/
34	(mental health or mood or depress* or affective disorder or negative affect or dysthymia or dysphoria or melancholic or anxiety or burden or distress or stress or well being or emotion or emotional).ab,ti.
35	exp "Depression (Emotion)"/ or exp Major Depression/

36	exp Anxiety Disorders/ or exp Anxiety/
37	exp Physiological Stress/
38	exp Emotions/
39	exp Mental Health/
40	exp Mood Disorders/
41	(mental health service* or mental healthcare or therapy or therapies or treatment* or intervention* or program* or psychological or psychologist or psychoeducation or psycho-education or CBT or health education or problem solving or PST or ACT or behaviour* or therapist or support or mindful* or psychotherapy or relaxation or meditation or behavior* or counseling or counselling or cognitive reframing or cognitive restructuring or self-help or self-management or blended).ab,ti.
42	exp Mental Health Services/
43	exp Health Education/
44	exp Problem Solving/
45	exp Psychotherapy/
46	exp Relaxation/
47	exp Self-Management/
48	exp Support Groups/
49	1 or 2 or 3 or 4 or 5 or 6 or 7 or 8 or 9
50	10 or 11 or 12 or 13 or 14 or 15 or 16 or 17 or 18 or 19
51	20 or 21 or 22 or 23 or 24 or 25 or 26 or 27 or 28 or 29 or 30 or 31 or 32 or 33
52	34 or 35 or 36 or 37 or 38 or 39 or 40
53	41 or 42 or 43 or 44 or 45 or 46 or 47 or 48
54	49 and 50 and 51 and 52 and 53
55	limit 54 to yr="2007 -Current"

Web of Science

#	
**1**	TI=(caregiver* OR care-giver* OR carer* OR family OR families OR spous* OR sibling* OR husband* OR wife OR wives OR partner OR partners OR parents OR parent OR friend OR friends OR relative OR relatives OR couple OR couples OR mother* OR father* OR support person* OR next of kin OR significant other*)
**2**	AB=(caregiver* OR care-giver* OR carer* OR family OR families OR spous* OR sibling* OR husband* OR wife OR wives OR partner OR partners OR parents OR parent OR friend OR friends OR relative OR relatives OR couple OR couples OR mother* OR father* OR support person* OR next of kin OR significant other*)
**3**	**#1 OR #2**
**4**	TI=(cancer OR tumor OR tumors OR tumours OR tumour OR neoplasm* OR oncology OR carcinoma OR malignan* OR melanoma OR metastasis OR lymphoma OR leukemia OR diabetes OR COPD OR chronic obstructive pulmonary disease OR COAD OR chronic obstructive airway disease OR chronic obstructive lung disease OR chronic airflow obstruction OR stroke OR cerebrovascular accident OR CVA OR acute cerebrovascular accident OR brain vascular accident OR apoplexy OR heart disease OR cardiovascular disease OR CVD OR ischemic heart disease OR IHD OR coronary artery disease OR CAD OR angina OR myocardial infarction OR MI OR heart attack OR cardiac event OR cardiac disease OR dementia OR alzheimer* OR FTD OR Lewy body OR Lewy bodies OR neurocognitive disorder* OR MCI OR mild cognitive impairment OR memory impair* OR cognitive decline OR chronic illness* OR chronic disease* OR chronic condition* OR multi-morbidit* OR multimorbidit* OR long-term health condition*)
**5**	AB=(cancer OR tumor OR tumors OR tumours OR tumour OR neoplasm* OR oncology OR carcinoma OR malignan* OR melanoma OR metastasis OR lymphoma OR leukemia OR diabetes OR COPD OR chronic obstructive pulmonary disease OR COAD OR chronic obstructive airway disease OR chronic obstructive lung disease OR chronic airflow obstruction OR stroke OR cerebrovascular accident OR CVA OR acute cerebrovascular accident OR brain vascular accident OR apoplexy OR heart disease OR cardiovascular disease OR CVD OR ischemic heart disease OR IHD OR coronary artery disease OR CAD OR angina OR myocardial infarction OR MI OR heart attack OR cardiac event OR cardiac disease OR dementia OR alzheimer* OR FTD OR Lewy body OR Lewy bodies OR neurocognitive disorder* OR MCI OR mild cognitive impairment OR memory impair* OR cognitive decline OR chronic illness* OR chronic disease* OR chronic condition* OR multi-morbidit* OR multimorbidit* OR long-term health condition*)
**6**	**#4 OR #5**
**7**	TI=(eHealth OR e-health OR e-mental health OR emental health OR mhealth OR m-health OR smartphone OR cell phone OR cellular phone OR mobile phone OR app OR application OR applications OR apps OR iPad OR computer OR tablet OR technology OR technologies OR electronic communication OR email OR e-mail OR text messag* OR internet OR wireless OR online OR digital OR on-line OR virtual OR ICT OR web OR website OR multimedia OR e-learning OR online social network OR iCBT OR cCBT OR e-therap* OR etherap*)
**8**	AB=(eHealth OR e-health OR e-mental health OR emental health OR mhealth OR m-health OR smartphone OR cell phone OR cellular phone OR mobile phone OR app OR application OR applications OR apps OR iPad OR computer OR tablet OR technology OR technologies OR electronic communication OR email OR e-mail OR text messag* OR internet OR wireless OR online OR digital OR on-line OR virtual OR ICT OR web OR website OR multimedia OR e-learning OR online social network OR iCBT OR cCBT OR e-therap* OR etherap*)
**9**	**#7 OR #8**

For peer review only

**10**	TI=(mental health OR mood OR depress* OR affective disorder OR negative affect OR dysthymia OR dysphoria OR melancholic OR anxiety OR burden OR distress OR stress OR well being OR emotion OR emotional)
**11**	AB=(mental health OR mood OR depress* OR affective disorder OR negative affect OR dysthymia OR dysphoria OR melancholic OR anxiety OR burden OR distress OR stress OR well being OR emotion OR emotional)
**12**	**#10 OR #11**
**13**	TI=(mental health service* OR mental healthcare OR therapy OR therapies OR treatment* OR intervention* OR program* OR psychological OR psychologist OR psychoeducation OR psycho-education OR CBT OR health education OR problem solving OR PST OR ACT OR behaviour* OR therapist OR support OR mindful* OR psychotherapy OR relaxation OR meditation OR behavior* OR counseling OR counselling OR cognitive reframing OR cognitive restructuring OR self-help OR self-management OR blended)
**14**	AB=(mental health service* OR mental healthcare OR therapy OR therapies OR treatment* OR intervention* OR program* OR psychological OR psychologist OR psychoeducation OR psycho-education OR CBT OR health education OR problem solving OR PST OR ACT OR behaviour* OR therapist OR support OR mindful* OR psychotherapy OR relaxation OR meditation OR behavior* OR counseling OR counselling OR cognitive reframing OR cognitive restructuring OR self-help OR self-management OR blended)
**15**	**#13 OR #14**
**16**	**#3 AND #6 AND # 9 AND #12 AND #15**

For peer review only

Implementation of e-mental health interventions for informal caregivers of adults with chronic diseases: a protocol for a mixed methods systematic review with a qualitative comparative analysis

Supplementary File 4: Data Extraction Form

**Study Identification Features**		
Unique Study Identifier		
Title		
First Author		
Year of Publication		
Country of Study		
Funding Source		
Protocol Available?		
**Study Characteristics**		
Aims and Objectives		
Study Design (e.g. RCT, qualitative)		
Inclusion/Exclusion Criteria		
Recruitment Setting and Method		
Randomization	Sequence Generation	
	Type	
	Allocation Concealment	
Blinding	Data Collectors	
	Data Analysts	
**Caregiver Characteristics**		
Caregiver Sample Size (total and per arm)		
Age		
Gender		
Severity of Anxiety at Baseline		
Severity of Depression at Baseline		
Severity of Distress at Baseline		
Severity of Stress at Baseline		
Relationship to Care Recipient		
Amount of Care Provided		
Length of Time as a Caregiver		
Education Level		
Employment Status		
**Care Recipient Characteristics**		
Sample Size (total and per arm)		
Diagnosis		
Age		
Gender		
Severity Measure of Physical Health Condition of Care Recipient		
**Intervention**		

For peer review only

Summary of Intervention	
Summary of Control Condition	
Type of Therapy (e.g. psychoeducation, CBT)	
Format of Intervention (e.g. web, mobile app)	
Individual or Dyadic Intervention	
Other Support Provided (e.g. some face to face contact)	
Provider (e.g. lay worker, psychologist)	
Training provided to intervention provider (yes/no, describe if yes)	
Total Length of Treatment	
Number of Sessions	
Length of Sessions	
Adherence (did participants receive intended intervention, did they complete entire intervention)	
**Implementation Outcomes**	
Implementation Outcomes Measured or Described	
Measurement Tools Used	
Timing of Measurements	
**Mental Health Outcome Measurements**	
Mental Health Outcome Measurements (primary and secondary)	
Quality of Outcome Measurements	
Timing of Measurements	
**Statistical Techniques**	
Power Calculation	
Target Sample Size	
Method of Dealing with Missing Data	
Baseline Comparability	
**Participant Flow**	
Eligible Participants	
Randomised to Intervention	
Randomised to Control	
Lost to Follow-Up Intervention[1]	
Lost to Follow-Up Control[1]	
Analysed Intervention[2]	
Analysed Control[2]	
**Results Summary**	
Summary of Mental Health Outcome Results	
Summary of Implementation Results	
**Quantitative Results[3]**	

For peer review only

Intervention	Outcome Measurement	
	Measurement Scale Used	
	Analysis Method (ITT, per protocol)	
	Pre-Treatment Means	
	Pre-Treatment Standard Deviation	
	Pre-Treatment Number Analysed	
	Post-Treatment Means	
	Post-Treatment Standard Deviation	
	Post-Treatment Number Analysed	
Control	Outcome Measurement	
	Measurement Scale Used	
	Analysis Method (ITT, per protocol)	
	Pre-Treatment Means	
	Pre-Treatment Standard Deviation	
	Pre-Treatment Number Analysed	
	Post-Treatment Means	
	Post-Treatment Standard Deviation	
	Post-Treatment Number Analysed	
**Additional Comments**		

[1]Numbers lost to follow-up to be reported at each time point measured; [2]Numbers analysed to be reported for each time point measured; [3]Results to be extracted for outcomes of anxiety, depression, distress and/or stress at all follow-up time points

For peer review only

Implementation of e-mental health interventions for informal caregivers of adults with chronic diseases: a protocol for a mixed methods systematic review with a qualitative comparative analysis

Supplementary File 5: Sample data table

Study	Conditions[a]				Outcome	
	Lay-led	Mobile app	Supplemented with personal contact	Implementing organization involved in project	Effect size (raw Hedges' g)	Effective intervention[b]
Study A	0	0	1	1	0.25	0
Study B	1	0	1	0	0.5	1

[a]The conditions shown here are examples of conditions that could be explored in the qualitative comparative analysis. These are subject to change as the conditions selected depend on the features and heterogeneity among studies included in the review. The number 1 signifies that the study belongs to the indicated set. For example Study A was not lay led or a mobile app, but it did contain personal contact and the implementing organization was involved in the project.

[b]Assuming an effective intervention is defined as having a Hedges' g effect size ≥ 0.3. In this example Study A has an effect size below this cut-off, therefore, is considered not effective.

For peer review only
